# Evolution of late-stage metastatic melanoma is dominated by aneuploidy and whole genome doubling

**DOI:** 10.1038/s41467-021-21576-8

**Published:** 2021-03-04

**Authors:** Ismael A. Vergara, Christopher P. Mintoff, Shahneen Sandhu, Lachlan McIntosh, Richard J. Young, Stephen Q. Wong, Andrew Colebatch, Daniel L. Cameron, Julia Lai Kwon, Rory Wolfe, Angela Peng, Jason Ellul, Xuelin Dou, Clare Fedele, Samantha Boyle, Gisela Mir Arnau, Jeanette Raleigh, Athena Hatzimihalis, Pacman Szeto, Jennifer Mooi, Daniel S. Widmer, Phil F. Cheng, Valerie Amann, Reinhard Dummer, Nicholas Hayward, James Wilmott, Richard A. Scolyer, Raymond J. Cho, David Bowtell, Heather Thorne, Kathryn Alsop, Stephen Cordner, Noel Woodford, Jodie Leditschke, Patricia O’Brien, Sarah-Jane Dawson, Grant A. McArthur, Graham J. Mann, Mitchell P. Levesque, Anthony T. Papenfuss, Mark Shackleton

**Affiliations:** 1 Bioinformatics Division, Walter and Eliza Hall Institute of Medical Research, Melbourne, Australia; 2 Peter MacCallum Cancer Centre, Melbourne, VIC Australia; 3 Melanoma Institute of Australia, Sydney, Australia; 4 Department of Mathematics and Statistics, The University of Melbourne, Parkville, VIC Australia; 5 Department of Medical Biology, The University of Melbourne, Parkville, VIC Australia; 6 School of Public Health and Preventive Medicine, Monash University, Melbourne, Australia; 7 Central Clinical School, Faculty of Medicine, Nursing and Health Sciences, Monash University, Melbourne, Australia; 8 Department of Dermatology, University of Zürich Hospital, Zürich, Switzerland; 9 QIMR Berghofer Medical Research Institute, Brisbane, Australia; 10 Tissue Pathology and Diagnostic Oncology, Royal Prince Alfred Hospital, Sydney, Australia; 11 Sydney Medical School, The University of Sydney, Sydney, Australia; 12 Department of Dermatology, University of California, San Francisco, CA USA; 13 Sir Peter MacCallum Department of Oncology, The University of Melbourne, Parkville, VIC Australia; 14 The Victorian Institute of Forensic Medicine, Melbourne, Australia; 15 Centre of Cancer Research, The University of Melbourne, Parkville, VIC Australia; 16 Centre for Cancer Research, Westmead Institute for Medical Research, University of Sydney, Sydney, Australia; 17 Department of Oncology, Alfred Health, Melbourne, Australia

**Keywords:** Cancer, Computational biology and bioinformatics, Evolution, Oncology

## Abstract

Although melanoma is initiated by acquisition of point mutations and limited focal copy number alterations in melanocytes-of-origin, the nature of genetic changes that characterise lethal metastatic disease is poorly understood. Here, we analyze the evolution of human melanoma progressing from early to late disease in 13 patients by sampling their tumours at multiple sites and times. Whole exome and genome sequencing data from 88 tumour samples reveals only limited gain of point mutations generally, with net mutational loss in some metastases. In contrast, melanoma evolution is dominated by whole genome doubling and large-scale aneuploidy, in which widespread loss of heterozygosity sculpts the burden of point mutations, neoantigens and structural variants even in treatment-naïve and primary cutaneous melanomas in some patients. These results imply that dysregulation of genomic integrity is a key driver of selective clonal advantage during melanoma progression.

## Abstract (web summary)

The genetic changes that occur in late stage metastatic melanoma are not well delineated. Here, the authors use rapid autopsy samples from metastatic melanoma patients and show that the late stage in the disease is characterised by whole genome doubling and aneuploidy.

## Introduction

Malignant transformation in cutaneous melanocytes is typically initiated by acquisition of single nucleotide variants (SNVs) resulting from ultra-violet (UV) light-induced DNA damage. The most common SNVs, which are even found in pre-neoplastic nevi^1–3^, constitutively activate the BRAF kinase or NRAS GTPase, although deleterious SNVs that impair tumour suppression by NF1 are also frequently detected^4,5^. For each of these major genotypic variants, concurrent point mutation affecting other genes is often thought to be required for the full invasive phenotype of melanoma^6–11^.

The role of structural variants (SVs; see Methods for definition) in melanoma is less well defined, although a subset of melanomas appears driven by oncogenic fusions^12,13^. Copy number alterations (CNAs), including deletions of the *CDKN2A* tumour suppressor and amplifications of *BRAF*, arise infrequently in nevi but are more abundant in invasive melanoma^6,14^. This suggests that the development of genomic instability is a key event in melanomagenesis, potentially facilitating allelic imbalances that support maintenance and progression of disease. However, understanding is limited of the mutational events that facilitate metastasis and the evolution of typically fatal late disease.

Previous studies have identified continued acquisition of both SNVs and SVs during cancer progression^15–18^. Consistent with this, in relatively limited sampling, an array of genetic changes was observed to develop over time within treatment-naïve melanomas in individual patients^6,14,19^, as well as during evolution of therapy resistance^20,21^. These included new SNVs as well as focal allelic amplifications and deletions, suggesting that melanoma progression is fuelled by mutations other than those classically induced by UV mutagenesis.

To address this and to overcome the challenges of obtaining multiple metastatic tissues from the same patients, the CASCADE (CAncer tiSsue aCquisition After Death) rapid autopsy program^22^ was established, enabling sampling of end-stage cancers. In this study, using tumour and blood samples from 7 melanoma patients enroled in CASCADE, as well as data from 6 patients from previous studies^19,21^, we characterised somatic mutational processes linked to end-stage melanoma. We found that although gain of SNVs is generally limited in melanoma progression, disease evolution is dominated by large-scale copy number changes, including universal tetraploidization and the acquisition of remarkable degrees of allelic imbalance highlighted by extensive loss of heterozygosity (LOH) in some patients. These findings implicate dysregulation of genomic integrity as a key driver of biological advantage during competitive subclonal growth in human melanoma.

## Results

### Patient enrolment and sequencing

**Fig. 1:**
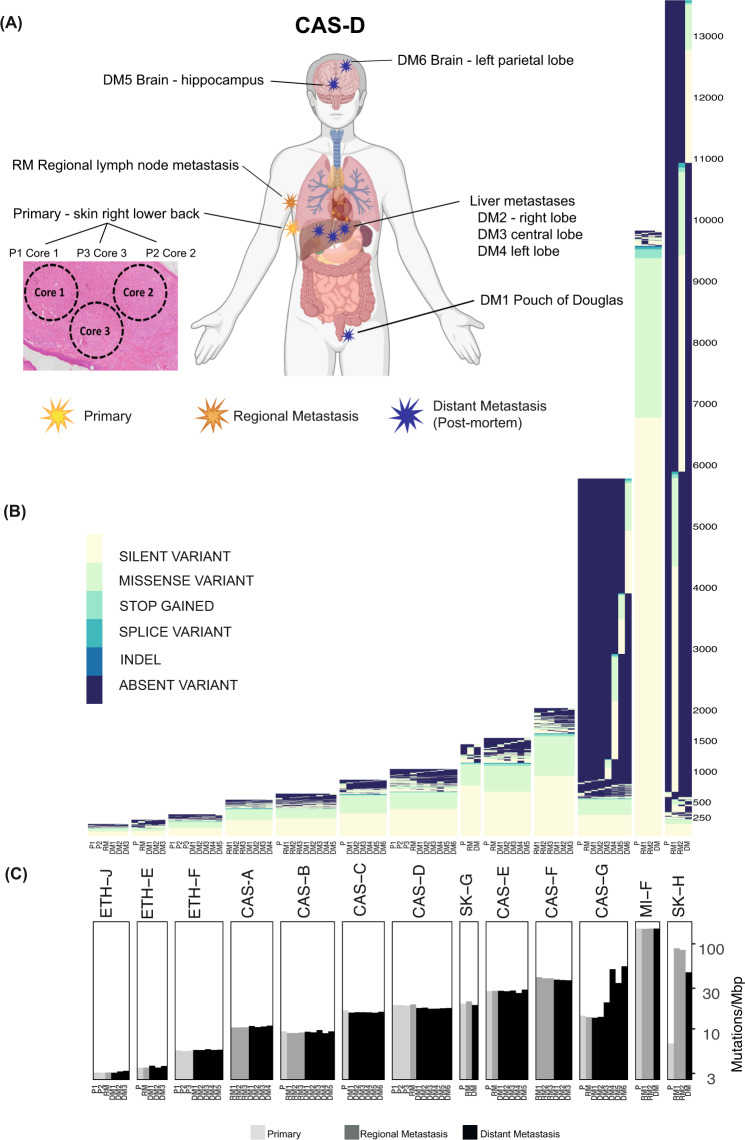
Temporal and multi-regional spatial sampling reveals limited acquisition of point mutations during melanoma progression to end-stage disease. **A** Spatial distribution of pre-mortem and autopsy samples for CASCADE patient CAS-D. Created with BioRender.com. **B** Unique SNVs and small indels detected across tumours from each patient. The identifier for each sample is described in Tables S1 and S2. *Y*-axis indicates the number of mutations in each patient. **C** Mutational load of SNVs and small indels across tumours from each patient. *Y*-axis is shown in a logarithmic scale.

Tumour samples from seven CASCADE melanoma patients with DNA available from normal tissues, matched primary melanomas and/or regional metastases, were chosen for genomic sequencing (Fig. 1A, Supplementary Figs. 1, 2A; EGA accession: EGAS00001004950). Survival times from melanoma diagnosis to death ranged from 8 months to 12 years (Supplementary Fig. 3). All CASCADE patients had BRAF mutant melanomas and received BRAF and/or MEK inhibitors during their disease course, with some also receiving immunotherapy (Supplementary Figs. 2A, 3). For each patient, 3–6 end-stage tumours (Supplementary Table 1) underwent whole exome sequencing (WES) (5 patients; mean coverage 156×, range 108–256×) or whole-genome sequencing (WGS) (2 patients, mean coverage 67×, range 40–81×). Multi-regional sampling of primary and/or regionally metastatic disease was performed in 4 patients to assess intra-tumoural heterogeneity (Fig. 1A, Supplementary Fig. 1). WES was also performed on plasma obtained prior to death from 4 patients (Supplementary Fig. 3).

With permission, published WES data from matched primary and metastatic disease from 6 additional patients were added to the cohort (Figs. S2B and S3, Supplementary Table 2)^19,21^. Of these, one had a BRAF mutant melanoma (ETH-E), two had NRAS mutant melanomas (SK-G & ETH-J), and three were BRAF/NRAS/NF1 wild-type (MI-F, SK-H, ETH-F; Supplementary Fig. 2B).

In total, 88 tumour samples from 13 patients were analysed. These spanned primaries, regional metastases, distant metastases and circulating tumour DNA and included multiple samples from individual tumours in some cases. We defined early mutations as those detected in the earliest available tumour tissue of each patient. This constituted primary melanomas for 11 patients and regional metastases for two patients. Mutations first observed in tumours recurring subsequent to this were termed late events. End-stage mutational events were those first observed in metastases obtained at autopsy. Patients were first diagnosed with disease at different stages: CAS-A, B and C presented with Stage I disease; CAS-F, CAS-G, ETH-E, ETH-F, ETH-J and MI-F had Stage III disease at the time of diagnosis; and CAS-E and D had Stage IV disease at diagnosis.

Sequencing data were first surveyed for SNVs and small insertion/deletions (indels) using an ensemble variant calling pipeline designed to take advantage of the multiple samples per patient (see “Methods”). Validation by targeted amplicon sequencing of a subset of predicted SNVs and small indels showed an accuracy of 90% (sensitivity 86%, specificity 98%) (Supplementary Notes, Supplementary Tables 3 and 4). Moreover, highly concordant variant calling was noted from DNA extracted from fresh frozen or formalin-fixed paraffin-embedded (FFPE) tissue from a visceral lung metastasis from patient CAS-G (Supplementary Notes, Supplementary Figs. 4, 5, Supplementary Table 5). This suggested only minimal FFPE artefacts in variant calling.

### Gain and loss of somatic SNVs and small indels during melanoma progression

Cutaneous melanomas typically have high numbers of SNVs^23^. Consistent with this, mutational loads from 3.3 (ETH-J) to 150.1 (MI-F) mutations/Mbp were observed in early melanomas in our cohort (Fig. 1B, C). Metastatic disease was usually dominated by these early mutations (Fig. 1B). In each late metastasis, loss of early mutations was apparent (median 2 mutations/Mbp) along with the emergence of new SNVs and indels (median 2.2 mutations/Mbp). Some of the new mutations were shared amongst other sites of late disease and some were unique to specific sites. Shared mutational losses and gains suggested evolutionary relationships between tumours.

**Fig. 2:**
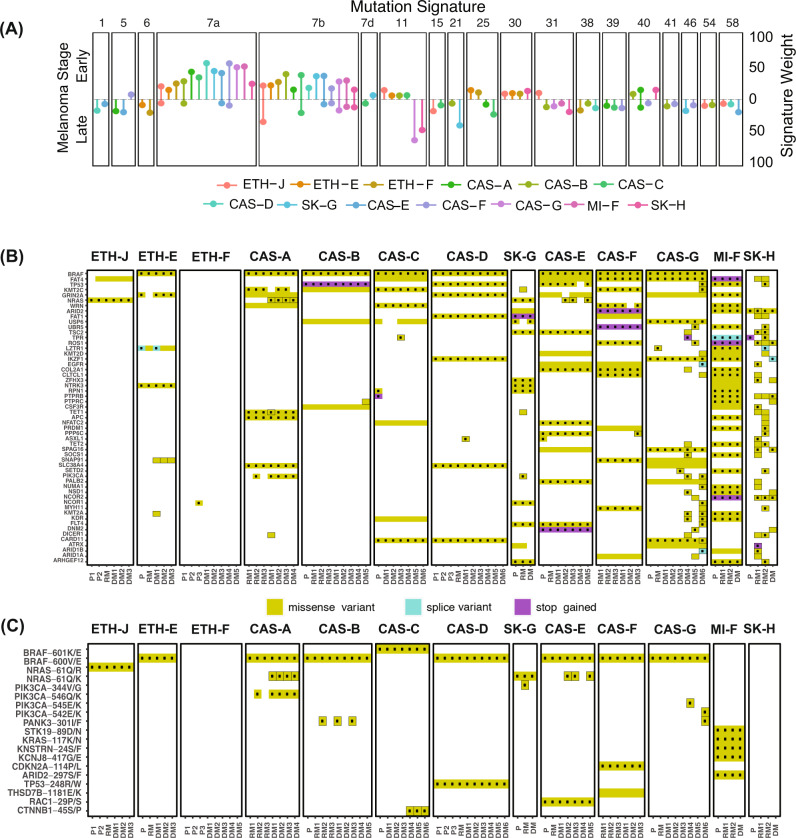
Timing of mutational processes and candidate drivers shows UV damage and DNA repair dysfunction dominate in chronologically distinct phases. **A** Mutational signatures in early and late disease. Shown signatures, represented by COSMIC identifiers, were identified in at least 2 patients. Vertical lines indicate the normalised inferred weight per patient with the indicated signature in early (upper) and late (lower) disease. **B** Recurrently mutated melanoma or COSMIC cancer genes in early or late disease. Shown genes were mutated in at least 2 patients and are presented in decreasing number of patients. Dotted mutations indicate predicted deleterious or COSMIC mutations. **C** Distribution of hotspot mutations^27^ across the cohort. Mutations surrounded by black indicate those occurring in late disease.

In order to understand mutational processes in melanoma evolution, we estimated mutational signatures in early and late disease in each patient (see “Methods”). Early mutations were predictably dominated by UV signatures (signatures 7a and 7b) (Fig. 2A). In contrast, mutations first observed in late disease mostly possessed non-UV signatures, with signatures 31, 38, 39 and 58 each apparent in at least 3 patients. While the aetiology of signature 38 is unknown, it is described as found exclusively in UV-associated melanomas. UV signature mutations also emerged in late disease in 8 patients, likely derived from low abundance subpopulations in early disease that seeded distant metastases.

In two patients (CAS-G and SK-H), metastatic disease was dominated by signature 11 (sometimes referred to as the temozolomide signature). Interestingly, although patient CAS-G had previously been treated with temozolomide, signature 11 mutations were evident in this patient’s lung and bowel but not liver metastases. Patient SK-H did not receive systemic therapy prior to tumour sampling^19^. In these patients, the signature 11 mutation loads in metastatic disease were coincident with predicted deleterious mutations in DNA-mismatch repair genes, including *MSH6* (CAS-G and SK-H), *MLH1* (CAS-G), *MLH3* (CAS-G and SK-H) and *MSH3* (SK-H); mutations in CAS-G were validated by targeted amplicon sequencing (Supplementary Table 3). Signatures 6, 15 and 21—all associated with DNA mismatch repair deficiency—were each present in two patients (signature 6: patients ETH-E and ETH-F; signature 15: patients ETH-J, CAS-C; signature 21: patients SK-G, CAS-B). These findings implicate DNA repair deficiency as a major contributor to gain of SNVs in late melanoma.

We interrogated the data from early disease for mutations in genes recurrently mutated in melanoma^5^ or in the COSMIC database (Fig. 2B and Supplementary Table 6), identifying *BRAF* (*n* = 8 patients), *FAT4* (*n* = 6), *TP53* (*n* = 4), *KMT2C* (*n* = 4), *GRIN2A* (*n* = 6), *NRAS* (*n* = 2), *WRN* (*n* = 4) and *ARID2* (*n* = 4) amongst the genes most commonly affected (Fig. 2B). The vast majority of SNVs in early disease were present in all metastatic sites.

We also identified cancer-associated genes that were mutated only in late disease (Fig. 2B), particularly in the two patients with impaired DNA repair, CAS-G and SK-H, whose rates of gained mutation across all metastases were 108 and 202 mutations/Mbp respectively. These included two independent mutations in *PIK3CA*^24^ in the lung (DM6, E542K mutation) and small bowel (DM4, E545K mutation) metastases of patient CAS-G (Fig. 2C). In patient CAS-C, mutations in *CTNNB1*, which is linked to melanoma metastasis^19,25^, were found exclusively in brain metastases at residues P44 and S45, both of which regulate beta-catenin^26^. Residues E542 and E545 in *PIK3CA*, and S45 in *CTNNB1*, are recognised mutational hotspots in cancer^27^. *PIK3CA*, which is associated with parallel subclonal evolution in melanoma^14^ and centrosome amplification^28^, was the gene that most commonly (n = 3 patients) acquired a hotspot mutation in late disease (Fig. 2C, Supplementary Table 7).

Apart from these examples, SNVs in cancer-associated genes appeared de novo in metastases only infrequently. Indeed, loss in late disease was seen in all patients of SNVs and indels present in early disease, such that no consistent net acquisition of these mutations was observed in the 11 patients without defective DNA mismatch repair.

### Acquisition of allelic imbalance and loss of heterozygosity

Apparent loss of SNVs and indels can be caused by a number of processes: deletion of one or more copies of an allele including LOH, reversion of mutations, dominance in metastases by sub-clones without mutations, and genetic heterogeneity in antecedent tumours not inherited by later metastases or acquired at the site of origin after dissemination of metastatic cells^29^. To better discern processes underlying loss of mutations, allele-specific copy number (CN) was estimated in our dataset.

**Fig. 3:**
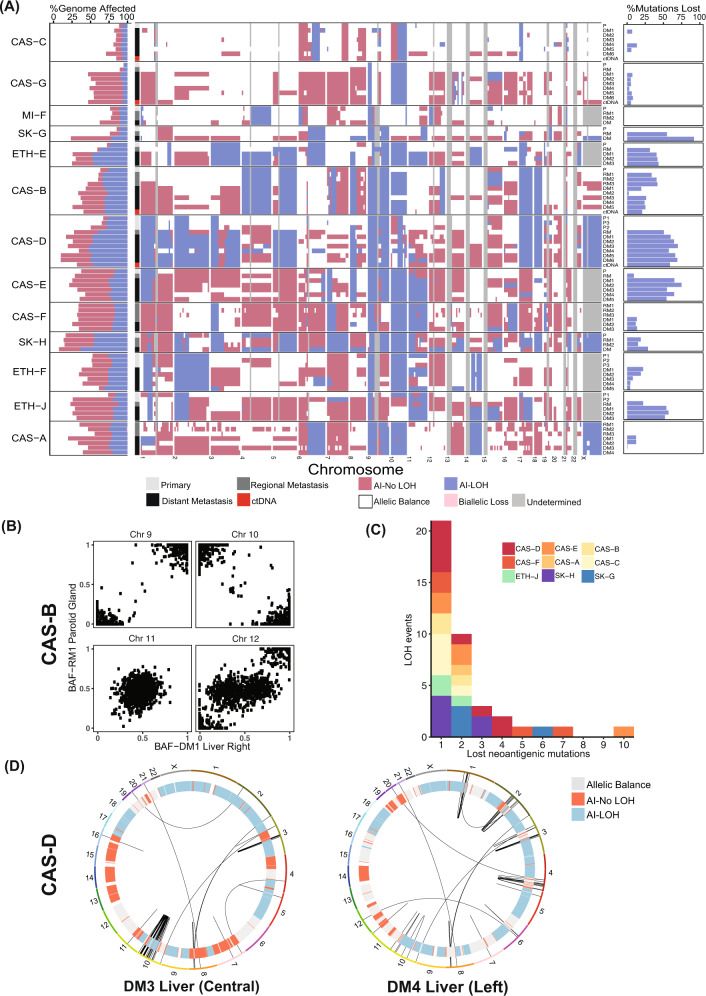
Widespread allelic imbalance (AI) and loss of heterozygosity (LOH) in metastatic melanoma. **A** Left: genomic proportions of each tissue sample affected by allelic imbalance. Horizontal bars indicate the fraction of each genome with the indicated allelic state. **Middle:** allelic imbalances in each sample at the cytogenetic band level. Regions of allelic balance have the same paternal and maternal allele copy number; regions of AI have different allele-specific copy numbers. AIs including an LOH event are shown separately. Undetermined regions are indicated in grey. **Right:** relationship between LOH and mutations lost during disease progression. Shown are proportions of all lost mutations that co-occur with acquisition of LOH events. **B** B-allele frequencies of single nucleotide polymorphisms in regional and distant metastases in patient CAS-B. A mirrored imbalance of heterozygous SNPs on chromosome 10 was evident between these lesions. **C** The impact of LOH events on neoantigenic mutations. Analysis of predicted neoantigenic mutations lost via LOH events across the cohort. *X*-axis indicates the number of neoantigenic mutations lost per LOH event. **D** Impact of LOH events on structural variants (SVs). Shown are Circos plots of large SVs detected in two liver metastases in patient CAS-D. Inner track indicates the allelic state for each chromosome.

Acquisition of allelic imbalance (AI), including LOH, was evident in all sites of disease in all patients (Fig. 3A, left and middle panels) and typically involved whole chromosomes or chromosomal arms. In early disease, the extent of AI varied widely from 5.4% (patient CAS-G) to 83.6% (SK-H) of the genome (Fig. 3A, left panel). In all patients, melanoma evolution was characterised by increasing AI either subclonally in early disease (e.g. multiple primary cores in patients CAS-D, ETH-J) or in subsets of metastatic samples (e.g. lesions in CAS-C, ETH-F). In some patients (CAS-D, ETH-E and SK-H), about 50% of the genome in late disease was affected by LOH.

Co-occurrence of SNV/indel loss and deletions was identified between early to late disease. LOH explained a significant proportion of lost SNVs and indels in late disease (Fig. 3A, right panel). Mutations lost in late disease and located in regions of retained heterozygosity had lower cancer cell fractions (in early disease) compared to those lost in regions of acquired LOH (Supplementary Fig. 6). This supports the hypothesis that these mutations were not present in the subclone that seeded the metastasis. This group could also have included mutations acquired in regions of copy number gain, where the mutated allele was subsequently lost. While mutations lost in regions of acquired LOH were mostly associated with the UV-induced mutational process with no other recurrent signatures (Supplementary Fig. 7), those lost in other regions were less well explained by this mutagen. Instead, multiple other mutational processes were observed in this group. For example, the presence of signatures 6 and 11 in patient CAS-G, signature 11 in MI-F and signature 14 in CAS-E suggested DNA-mismatch repair defects may have also occurred in early disease in these patients (Supplementary Fig. 8). This suggests that acquisition of somatic mutations via DNA repair deficiency may not only be common in late disease (Fig. 2A) but also occur subclonally in early melanoma.

Within each patient, allele-specific copy number alterations (CNAs) typically involved the same alleles, suggesting common origins of these clones. However, akin to observations in lung cancer^30^, different alleles in distinct metastases were occasionally involved. In two liver metastases from patient CAS-G, chromosome 15 acquired LOH by independent deletion events affecting different alleles (Supplementary Fig. 9). Similarly, in patient CAS-B, chromosome 10 acquired LOH by deletion of different alleles in regional and visceral metastases (Fig. 3B). Mirrored AIs such as these might reflect convergent evolution driven by continued chromosomal instability and selection for gene dosage^30^, effects of varied selective pressures in distinct environments, or selective deletion of different subclonal mutations.

As LOH events accounted for a major proportion of lost mutations in late disease (Fig. 3A, right panel), we wondered whether deletion of disadvantageous non-synonymous mutations or neoantigens might favour selection of LOH. Indeed, LOH might provide an efficient mechanism to improve the fitness of melanoma cells through the removal of multiple deleterious mutations and/or by eliminating multiple neoantigenic SNVs in single mutational events.

Of the 506 LOH segments acquired across all patients, 157 (31%) deleted at least one non-synonymous mutation (Supplementary Fig. 10A). As LOH segments that did not delete non-synonymous variants tended to be smaller (Supplementary Fig. 10B), possibly resulting from over-segmentation in copy number estimation (Supplementary Fig. 10C), we considered only segments greater than 10 Mb (*n* = 194).

We reasoned that if LOH provides a fitness advantage by selective deletion of disadvantageous non-synonymous mutations, we might observe the preferential loss of regions with a higher than the expected number of non-synonymous mutations, which can be estimated from the number of synonymous mutations and human codon usage frequencies (see Supplementary Material). Across all patients, only 67/194 large acquired LOH segments deleted more non-synonymous mutations than expected, which is not more frequent than expected by chance (*p* = 0.8, one-sided binomial test; see Supplementary Methods). This does not support a dominant role for removal of highly mutated regions, but does not rule out selective loss of individual mutations impacting fitness.

We next considered the potential role of neoantigens resulting from SNVs in early disease by predicting neoantigens for patient-specific HLA-A, -B and -C alleles using NetMHC4.0^31,32^ (see Supplementary Methods) and examining the loss of these predicted neoantigens due to LOH in late disease. We found between 43 and 2109 predicted neoepitopes with weak or strong HLA class I binding affinity in each patient (median 217) in early disease. Of these, 18–985 (median 107) occurred in genes commonly expressed in melanoma (see Supplementary Methods). To evaluate the extent to which acquired LOH events delete predicted expressed neoantigens, we collected overlapping LOH segments into events by comparing late disease samples within each patient. Patients lost from 2 (CAS-A) to 18 (CAS-D) predicted expressed neoantigens due to LOH. Thirty-nine LOH events removed from 1 to 10 (CAS-D) predicted expressed neoantigens, with 19 removing from 2 or more (Fig. 3C, Supplementary Table 8). Patients lost from 2 (CAS-A) to 18 (CAS-D) predicted expressed neoantigens due to LOH. Thirty-nine LOH events removed from 1 to 10 (CAS-D) predicted expressed neoantigens, with 19 removing from 2 or more (Fig. 3C, Supplementary Table 8). This highlights that large-scale LOH may provide an efficient mechanism to remove expressed neoantigens, or regions with high neo-antigenicity, in single mutational events. However, it is important to note that acquired LOH may reduce only a small proportion of the overall neoantigen load, as we observed.

These observations led us to consider if some regions in individual patients might demonstrate a preference for deletion of neoantigens over non-neoantigenic non-synonymous mutations, or vice versa. To test this, we looked for regions of LOH where high predicted neo-antigenicity and high rates of non-synonymous mutations were mutually exclusive, finding no such inverse association in 7 of 8 patients tested (Supplementary Table 9), although several patients could not be tested due to low numbers of mutations. Of these, 2 (CAS-F and ETH-J) only lost regions with high predicted neo-antigenicity. Interestingly, one patient, CAS-D, showed strong mutual exclusivity (p = 0.02, two-sided Barnard’s test) with 22 LOH regions with high predicted neoantigenity and low rates of non-synonymous mutations, and 13 LOH regions with low predicted neo-antigenicity and high numbers of non-synonymous mutations.

These results fall short of demonstrating a dominant role for the sculpting by LOH of non-synonymous mutations or neoantigens, but leave open the possibility that selection for deletion of specific mutations may account for large-scale LOH in some patients. Certainly, other factors may influence the selection of LOH events, such as consequent changes in the dosage of affected transcripts and the presence of advantageous mutations whose deletion may be selected against. Additionally, reduction of the allelic load of non-synonymous or neoantigenic mutations through the acquisition of allelic imbalance was not considered.

Despite the above, mutations lost in regions of acquired LOH possessed other properties that might suggest enhanced neo-antigenicity. In some patients, deleted predicted strong binding neoantigenic mutations occurred in genes with higher expression (Supplementary Fig. 11). Also, predicted neoepitopes lost due to LOH frequently had higher estimated cancer cell fraction in early disease than those lost through other mechanisms (Supplementary Fig. 12: CAS-A, CAS-B, CAS-C, CAS-D, CAS-E, CAS-F, SK-G, SK-H) and possessed stronger predicted binding affinity to specific HLA alleles in some cases (Supplementary Fig. 13: CAS-D HLA-C:06:02, CAS-F HLA-B:44:03, SK-G HLA-B:07:02 SK-G). Although neoantigen prediction has poor specificity for predicting immunogenicity^33,34^, these data support the idea that LOH events might provide a more efficient means than individual SNV reversions for clonal immuno-editing^35^.

As structural variants (SVs), such as gene fusions are increasingly recognised oncogenic drivers in melanoma^12,13^, we also examined the impact of LOH on SVs by analysing WGS data obtained from patient CAS-D. Several SVs observed in chromosomes 2 and 4 in the left liver metastasis were absent from the central liver lesion with coincident LOH. Similarly, the extension of LOH in chromosome 10 of the central liver lesion coincided with loss of SVs (Fig. 3D). This was not detectable in ctDNA, which nevertheless represented the most abundant SVs across late disease sites (Supplementary Fig. 14). LOH can thus shape not only the burden of SNVs and indels, but also SVs. While the selective subclonal advantage of individual LOH events is hard to predict, these observations highlight the possibility that such changes sculpt the broad mutational landscape of cancer genomes.

### Increased ploidy dominates the landscape of end-stage melanoma

**Fig. 4:**
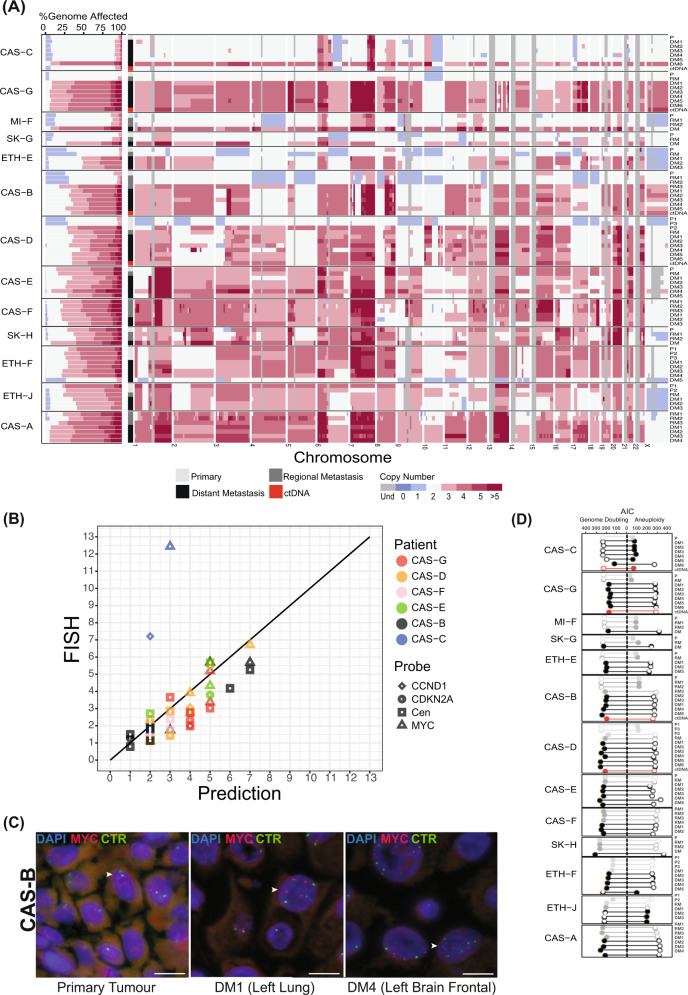
Ubiquitous hyperploidy in metastatic melanoma. **A** **Left:** genomic proportions of each tissue sample affected by copy number alterations (CNAs). Horizontal bars indicate the fraction of each genome with the indicated allelic copy number. Regions with total CN > 5 are represented by the same colour. Grey regions indicate undetermined CN. Right: CNAs in each sample at the cytogenetic band-level. **B** Validation of CN in 6 patients using Fluorescence In Situ Hybridisation (FISH). X-axis corresponds to the total CN of the segment overlapping the FISH probe predicted from sequencing data. Y-axis corresponds to the average CN across cells in each sample, determined by manual counting, for the indicated FISH probe. Centromeric probes corresponded to chromosomes 8, 9 and 11 as controls for probes for *MYC*, *CDKN2A* and *CCND1*, respectively, in those chromosomes. Cen: Centromeric probe. **C** FISH showing *MYC* amplification in two metastases from patient CAS-B, compared to the primary tumour (left). Arrow heads indicate cells with red (Myc) and green (centromere) signals. Scale bar = 10um. CTR: Centromere probes for chromosome 8. **D** Widespread genome doubling in melanoma. For each sample, the bar length indicates the Akaike Information Criteria (AIC) score obtained via branching process analyses of aneuploidy with genome doubling (left) versus aneuploidy alone (right). Solid circles indicate the most likely scenario for each sample.

We next considered total copy number (Fig. 4A). Consistent with observations of LOH and AI, CNAs in late disease were often large-scale and generally detectable in ctDNA. They were also frequently manifest as amplifications (Fig. 4A, left panel), such that the mean ploidy across late disease was 2.9 (range 1.6–4.2).

Largely diploid (mean 2, range 1.6 – 3.3) early disease was observed in patients CAS-B, CAS-C, CAS-D, CAS-G, MI-F, SK-G and ETH-E. Across these cases, increased ploidy was not usually seen until the development of distant metastases. However, in patients CAS-B and CAS-D, increased ploidy occurred subclonally in regional metastases and primary sites of earlier disease, respectively. Increased ploidy (mean 3.4, range 2.7–3.8) was observed in the early disease of all other patients, in which it was at least maintained in all but one metastasis (patient ETH-F, tumour DM5), which may have been seeded independently from a lower ploidy ancestor. Across this cohort, chromosomes 1q, 6p, 7, 8q, 13q, 15q, 20, 21q, and 22q were recurrently amplified (Fig. 4A, right panel), suggesting net selective advantage of increased expression of genes in those regions, including *BRAF*, *RAC1* and *MET* on chromosome 7, *AKT3* on chromosome 1q and *MYC* on chromosome 8q. The presence of large CNAs in treatment-naïve melanomas indicates that such changes can occur independently of treatment.

Predicted ploidy changes were validated in 6 patients by fluorescence in situ hybridisation (FISH) using probes against *MYC*, *CDKN2A*, and *CCND1*, and against centromeres in chromosomes 8, 9 and 11 (r = 0.76, Spearman correlation; Fig. 4B). In patient CAS-B, the increased copy number in visceral metastases of the brain and the lung was confirmed by probes for *MYC* and the chromosome 8 centromere (Fig. 4C). Further, FISH to *CDKN2A* confirmed a hemizygous deletion affecting 9p21.3 in the primary melanoma and diploidy of the same region in metastases (not shown). For patient CAS-D, the prediction of ploidy differences between spatially distinct cores of the primary melanoma was supported by FISH to *MYC* and the chromosome 8 centromere (Supplementary Fig. 15). Increased ploidy was also observed in a distant liver metastasis of CAS-D, based on FISH to *MYC* and *CCND1* (Supplementary Figs. 15, 16). Indeed, probe counts for *CCND1* suggested intra-tumoural heterogeneity in this metastasis, with a dominant clone carrying 5 copies of *CCND1* and a minor clone (10% of cells) carrying 8 copies (Supplementary Fig. 16).

A striking case of disagreement in copy number between predicted and FISH-observed copy number was noted in a brain metastasis of CAS-C (Fig. 4B and Supplementary Fig. 17), in which average probe counts for *MYC* and *CCND1* were 7.2 and 12.4 respectively. Assessment of this metastasis revealed extensive heterogeneity, with sub-clones that carried either no copies or a mode of 8 copies of *MYC*, and a separate population of very large cells (~27% of total cells) each with >30 copies of *MYC* (Supplementary Fig. 17). Amplifications of this magnitude have been reported in melanoma^36^ and may be due to extra-chromosomal DNA^37^.

### Near-ubiquitous tetraploidization in late melanoma

Tumours with increased ploidy were typically characterised by large-scale chromosomal CNAs ranging from 2–4 copies. Although this might be explained by the stochastic acquisition of multiple amplifications, we wondered whether such ploidy change more likely resulted from whole-genome doubling (WGD) events that occurred against a background of smaller-scale stochastic amplifications and deletions. To test this, we developed two branching process-based models (see Methods): one modelled aneuploidy only and the other modelled aneuploidy together with a single WGD event. We used the Akaike Information Criteria (AIC) to penalise for the extra parameter in the genome doubling model and determine which model better explained the data.

In this analysis, increased ploidy was better explained by the combination of WGD together with chromosomal or arm-level CNAs (Fig. 4D). Indeed, every patient showed evidence of WGD. Multi-regional analysis of two primary melanomas (samples P2 in CAS-D and P1 in ETH-J) indicated that WGD can be an early subclonal event in melanoma (Fig. 4A, D). In contrast, CAS-C possessed only one tumour at post-mortem, a brain metastasis, which had undergone WGD. Although WGD was reflected in ctDNA from three patients, WGD in patient CAS-C was private to the brain metastasis.

The observation of near-universal genome doubling together with aneuploidy across patients supports the hypothesis that these mechanisms provide an advantage to tumour cells. Tetraploidization may act as an efficient mechanism to achieve high levels of aneuploidy that increase the biological tolerance of deletion events^38,39^ or help reduce the probability of large-scale homozygous losses, which can lead to subpopulations with decreased fitness. Interestingly, multiple oncogenes were found amplified within LOH regions at a copy number beyond that expected given the ploidy of the lesion (Supplementary Fig. 18). These include genes that drive oncogenicity via amplification^40^ such as *LMO1* (4 patients), *CCND1* (3 patients), *MYCN* (2 patients) as well as *MYC*, *MDM2*, *MDM4* and *NKX2-1* (1 patient). Oncogenes linked to melanoma progression were also amplified, including *NRAS*, *KIT* and *RAC1*, the latter highly amplified in four patients in regions with LOH at copy numbers of 5 (patient ETH-F), 6 (patients CAS-E and SK-H) and 7 (patient CAS-F). This suggests amplification of the remaining allele in regions of LOH may confer selective advantage to the tumour via over-expression of driver genes.

### Mutations in genes that regulate genomic integrity

**Fig. 5:**
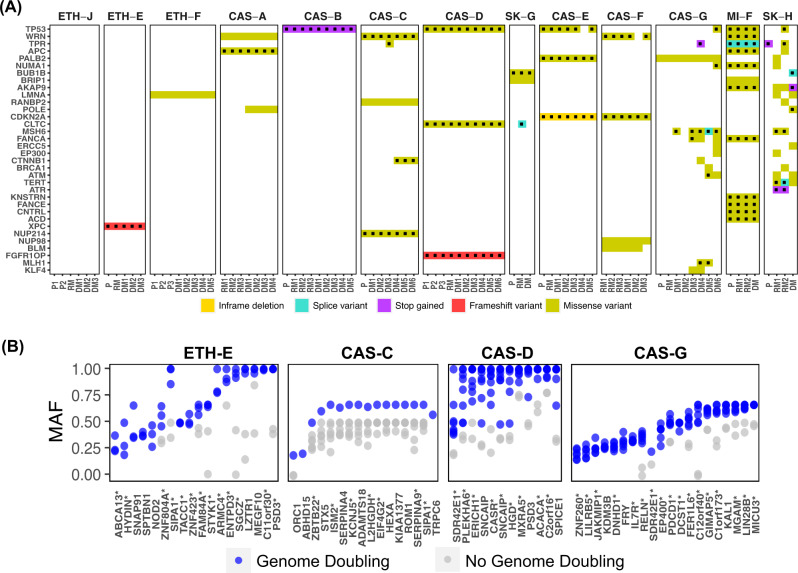
Potential drivers of aneuploidy and whole genome doubling in early-stage and metastatic melanoma. **A** Recurrent mutations across the cohort in genes that regulate chromosomal structural integrity, cell cycle and DNA repair. Dotted mutations indicate predicted deleterious or COSMIC mutations. Genes included correspond to significantly mutated genes in the TCGA cohort or COSMIC cancer census genes (see Supplementary Methods). The identifier for each sample is indicated in Tables S1 and S2. **B** Mutant allele fraction (MAF) of mutations gained or increased in allelic dose with increased ploidy, in association with genome doubling, in patients ETH-E, CAS-G, CAS-D and CAS-C. Each point per gene represents a sample. MAF: estimated fraction of mutated alleles in the tumour cells of the sample. *predicted deleterious or COSMIC mutations.

Due to the high level of CNAs in late melanoma, we also considered mutations in genes that regulate genomic integrity^41–44^, cell cycle and DNA repair (Supplementary Table 10, Fig. 5A). Deleterious TP53 mutations, which are associated with tolerance of aneuploidy^45^, were acquired early in four patients (Fig. 5A) and in late disease in two others. Interestingly, amplification of wild-type MYC was also observed in two of these patients, CAS-D and CAS-B, providing a mutational combination proposed to facilitate tetraploidization^46^. *CLTC* (mitotic spindle instability), *APC* (checkpoint defects, merotely) and *BUB1B* (checkpoint defects) were also amongst genes involved in regulation of genomic integrity recurrently affected by deleterious mutation^42,43^ (Fig. 5A). Mutations in genes that regulate the cell cycle and DNA repair further suggested mechanisms of increased CNAs: a truncating insertion in FGFR1OP, which anchors microtubules^47^ (patient CAS-D) and deleterious missense mutations in WRN, associated with homology-directed repair and stability of fragile sites^48^, in patients CAS-C, CAS-F and MI-F. Notably, CDKN2A underwent an in-frame deletion (COSMIC variant COSM24418) in patient CAS-E and a hotspot P114L substitution (COSMIC variant COSM753743) of reported functional significance^49^ in patient CAS-F.

To identify mutations associated with hyperploidy using an unbiased approach, we further evaluated patients for whom both low and high ploidy samples were available, identifying SNVs present in tetraploid but not matched diploid samples, or increased in relative dose after tetraploidization (Fig. 5B, Supplementary Table 11). The 63 genes identified by these criteria across patients CAS-C, CAS-G, CAS-D, and ETH-E regulate multiple biological processes, including signal transduction and cell differentiation (Supplementary Table 12). Mutations were also gained in genes linked to cell cycle progression and cytoskeletal and microtubule organisation, including *SIPA1*^50^ (n = 2 patients), *TACC1*^51^, *SPICE1*^52^ and *FRY*^53^, amongst others (Supplementary Table 12). While 88 samples were available, shortcomings of our study were that the number of patients was relatively low (13) and samples possessed a complex dependence structure, confounding hypothesis testing. Nevertheless, these findings suggest diverse potential drivers of disrupted genomic integrity in melanoma progression.

### Tumour phylogenies

**Fig. 6:**
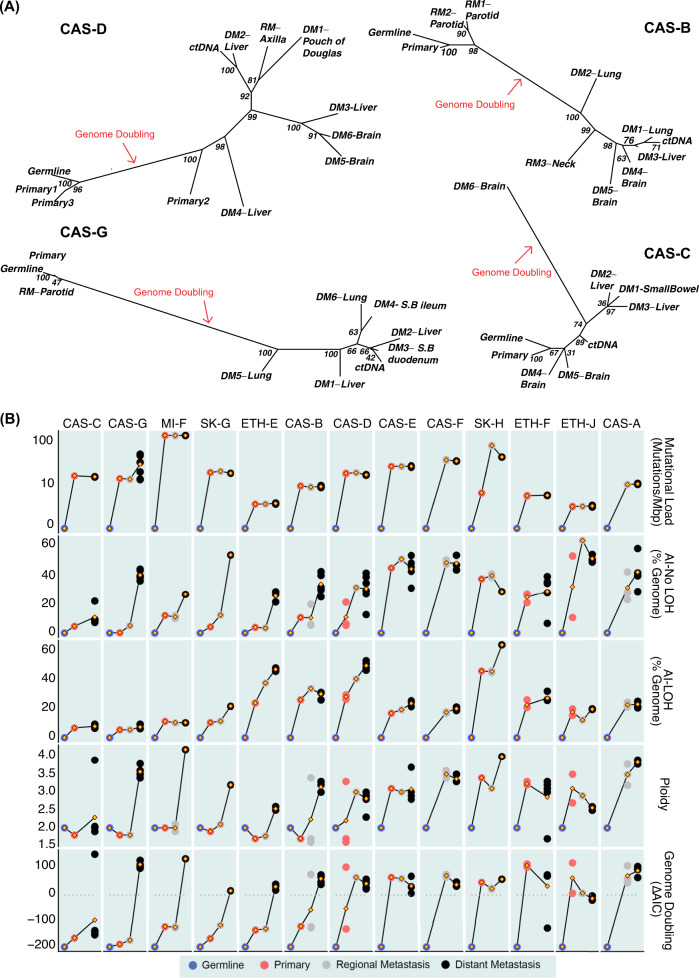
Evolution of cutaneous melanoma. **A** Copy number-based phylogenetic trees for patients CAS-D, CAS-B, CAS-G and CAS-C. Evolutionary relationships between samples were derived using MEDICC, based on allele-specific copy numbers. Support values for each node were obtained by resampling of the distance matrix. MEDICC treated genome doubling events as independent chromosomal amplifications, resulting in long branch lengths. S.B.: Small Bowel. **B** Summarised genetic changes in the progression to end-stage melanoma. From top to bottom: evolution of somatic mutational load (mutations/Mbps), allelic imbalance without LOH (% genome affected), allelic imbalance with LOH (% genome affected), average ploidy, and evidence for genome doubling (ΔAIC = AIC_non-WGD_-AIC_WGD_, where AIC_non-WGD_ is the Akaike Information Criterion (AIC) for the model with aneuploidy only, and AIC_WGD_ is the AIC for the model that includes WGD; ΔAIC > 0 supports genome doubling) across primary melanomas and regional and distant metastases for each patient. Germline estimates are depicted as comparative references. Yellow diamonds joining lines represent mean values for each time of disease. Each dot represents an individual tissue sample.

As melanoma evolution was found to be dominated by CNAs, we estimated allele-specific CNA-based phylogenetic trees of spatiotemporally separated tumours and late disease ctDNA from each of 4 patients (CAS-D, CAS-B, CAS-G, CAS-C) whose melanomas progressed from near-diploid in early disease to hyperploid in late disease (Fig. 6A) and in 2 patients (SK-H, MI-F) with previously published trees^19^ (Supplementary Figs. 19, 20). We also estimated phylogenetic trees from SNVs and jointly from SNVs and copy number (Supplementary Figs. 19–24). This provided insight to disease evolution in these cases, including evolutionary branches on which genome doubling was likely to have occurred and patterns of aneuploidy before and after genome doubling.

Based on these analyses, we observed that metastatic disease in patient CAS-D (Fig. 6A, left) most likely arose from the sub-clone in the primary melanoma that underwent genome doubling (P2, Supplementary Fig. 15). The left liver lesion (DM4) probably developed from an early seeding from this sub-clone, as it displayed similar allelic balance in chromosome 1q, which was affected by LOH in all other metastatic sites (Fig. 3, Supplementary Fig. 25). The central liver lesion (DM3) in this patient clustered closest with brain metastases, suggesting a shared subclonal origin. A SNV-based phylogeny for this patient (Supplementary Fig. 21) showed similar disease evolution patterns, but reflected that the vast majority of the SNV burden was acquired in earlier disease progression compared to CNAs. We assumed that widespread metastases would contribute to ctDNA mutations, however, the CNA profile of late disease ctDNA in patient CAS-D very closely resembled the caudate lobe liver lesion (DM2) (Fig. 6A, left). This was also observed in the SNV phylogeny, in addition to resemblance to axillary regional metastasis and the Pouch of Douglas lesion (DM1). This suggests that some more than other metastases may shed tumour-associated DNA into the circulation despite their co-location in the same organs. A joint evidence tree based on both SNVs and CNAs (Supplementary Fig. 21) confirmed patterns of progression and the similarity of ctDNA.

Although genome doubling was observed universally in almost all distant metastases (Fig. 4D and 6A), continued acquisition of aneuploidy occurred after genome doubling in patients CAS-G, CAS-D and CAS-B (Fig. 6A and Supplementary Figs. 22, 23). Additionally, the timing of genome doubling varied, occurring in the primary tumours of CAS-D (subclonally), CAS-E, SK-H, ETH-F and ETH-J (Fig. 4D), but only observed in one brain metastasis of CAS-C (Fig. 6A and Supplementary Fig. 24). Thus, genome doubling is not absolutely required to permit distant metastases, and CNAs can occur both before and after genome doubling.

The joint SNV and CNA phylogenies for these patients (Supplementary Figs. 21–24) retained the consistent clustering of early versus late mutations compared to germline, as well as the preferential grouping of ctDNA samples with specific mutations, with differences likely the result of enhanced phylogenetic signal when combining these data. For patients MI-F and SK-H, previously reported phylogenies^19^ were in agreement with those generated here with the same event types (Supplementary Figs. 19, 20, Supplementary Notes).

The phylogenetic reconstruction provided by MEDICC CNA-based trees was inspected for further instances of convergent evolution, revealing evidence of parallel evolution and reversals of mutations by LOH, as well as mirrored allelic imbalances. For example, we examined allele-specific copy number variants in patient CAS-D and sought CNAs that occurred more than once in different parts of the evolutionary tree (Supplementary Fig. 26). Chromosome 12q was initially diploid (in P1 and P3) prior to genome doubling and lost a copy to reach allele-specific copy number (2, 1) within the primary (P2). This was followed by a further gain to copy number (2, 2) and then multiple independent losses in distant metastases to (2, 1). Similarly, the region 11q23–11q25 was also initially diploid. Following WGD (P2), it underwent arm-level deletion to allele-specific copy number (2, 1), then independent gains in two distant metastases of the liver to return to allele-specific copy number (2, 2).

The evidence for convergent evolution presented here is likely an underestimate, as previous studies have illustrated parallel acquisition of mutations through disease progression in genetically identical inter-patient cancers^54^ as well as in intra-patient single-cell resolution characterisation of melanoma^29^. Phylogenies like the ones presented here will become increasingly more accurate with temporal-spatial sampling coupled with single-cell resolution data and methodologies for subclonal evolutionary history reconstruction that do not rely on the infinite sites model assumption^55^.

Comparison of the timing of CNA and SNV acquisition during melanoma development confirmed that the burden of SNVs in late disease in each patient was mostly acquired in early disease and did not change substantially during disease progression (Fig. 6B), with exceptions in the two patients who developed DNA mismatch repair defects (SK-H and CAS-G). In contrast, the kinetics of CNA acquisition varied markedly. All patients had at least low levels of aneuploidy in early disease with highly recurrent full or partial loss of chromosomes 9 and 10. While WGD occurred in at least one tumour in every patient, it was first observed in the early disease of 5 patients (CAS-E, SK-H, ETH-F, ETH-J, and subclonally in CAS-D), in the regional metastases of CAS-B (subclonally), and not until late disease in 5 patients (CAS-C, CAS-G, MI-F, SK-G, ETH-E).

Of the 11 patients for whom we analysed one or more samples of primary cutaneous melanoma, 5 showed >15% of the genome affected by allelic imbalance, 7 showed >15% of the genome affected by LOH, and 5 showed ploidy >2.5. Three patients had multi-regional sampling of their primary melanomas and all of these showed genomic instability in at least one region. It is possible that subclonal chromosomal instability in the primary melanomas we evaluated was more frequent than observed due to sampling.

Interestingly, degrees of AI and hyperploidy varied amongst distant metastases of individual patients in the latter cohort, with some metastatic sites showing decreased AI and reduced ploidy compared to early disease. In contrast, the extent of LOH increased in almost every distant metastasis (Fig. 6B), suggesting common clonal origins of cells carrying these events. This suggests that genomic sculpting by deletion events confers biological advantage in melanoma.

## Discussion

We performed multi-regional sampling of end-stage melanoma at autopsy^21,22^. This enabled us to characterise genetic changes associated with progressive disease, finding that tetraploidization and acquisition of aneuploidy are dominant mutational mechanisms across evolution to end-stage, fatal melanoma. This contrasts with the key roles of UV-induced SNVs in melanocytic transformation to early melanoma^4,6^. Although SNVs or indels affecting genes associated with cancer were abundant in the early melanomas of our cohort, their continued acquisition was minimal during progression to late disease. In contrast, acquisition of CNAs was abundant. Although at least low-level aneuploidy was consistently found in early disease, with recurrent LOH in chromosomes 9 and 10, most sites of metastatic and end-stage disease were affected by aneuploidy due to both WGD and gain and loss of individual chromosomes or chromosome arms. Our findings extend previous studies^6,14,19,20^ to illuminate the breadth of genetic changes that drive melanoma progression through treatment to end-stage disease.

Two patients (CAS-G and SK-H) were exceptions to the above pattern, displaying markedly increased SNV burdens in metastatic disease in association with acquisition of mutations in known DNA mismatch repair genes. Indeed, across all patients, mutational signatures associated with aberrant DNA repair were observed in SNVs that first appeared in sites of metastatic disease. This highlights the importance of defective DNA repair as a mechanism that permits cancer cell adaptation in the face of selective pressures such as anti-cancer therapy, and is consistent with known SNV-based mechanisms of acquired resistance in melanoma to targeted therapy^56,57^ and immunotherapy^58^.

However, loss of SNVs was also observed, often as a result of LOH. The proportions of tumour genomes affected by LOH were increased in almost all patients in late distant metastases, in some cases to >50% compared to earlier disease. One potential role of LOH may be as an efficient means for providing biological advantage by eliminating multiple deleterious mutations and reducing immunogenicity through deletion of neoantigens in single mutational events. Although only limited support was found for the possibility that LOH is a strongly selected driver of loss of such mutations, LOH could also be detrimental if it reduced critical gene products. In this sense, pervasive WGD, which we observed in the vast majority of metastases in our cohort, may protect sub-clones by providing adequate essential proteins while at the same time permitting selective advantage from focal deletion events.

Previous studies showed emergence of aneuploidy in early melanocytic neoplasia that was associated with disease evolution to the invasive and metastatic state^6,14^. Our data demonstrate that WGD can also occur in untreated primary cutaneous melanomas, in which it may develop subclonally. These findings indicate that tetraploidization and acquisition of aneuploidy are not necessarily consequences of the stresses to cancer cells imparted by anti-cancer therapy or the metastatic process. Nor are they required for metastasis and therapy resistance; one patient in our cohort (CAS-C) harboured several late metastases that had not undergone WGD. Rather, such genomic derangements appear to be widespread events in intra-tumoural evolution of melanoma that confer competitive subclonal advantage, including via increasing metastatic potential, presumably due to the changes they confer in allelic doses of wild-type (WT) genes and/or genes affected by SNVs and indels.

Pan-cancer studies^59–61^ have shown that hyperploidy is prevalent in cancer and associated with WGD in an estimated 37% of cases^61^. In in silico modelling^38^, an apparently optimal state of triploidy in cancer was achieved more efficiently by initially tetraploid cells than by diploid cells, presumably as a result of the ability of the former to buffer lethal events such as nullisomy. This is consistent with the mode of 72 chromosomes per cell observed in the Mitelman Database^38^ and the mean ploidies in our cohort of 2.9 in late metastases and 3.4 in high ploidy early primary melanomas.

While tetraploidization is a genome-wide event, AIs acquired through disease progression in our cohort targeted specific chromosomes. We hypothesise that selection of region-specific AIs is favoured because mutations in those regions modulate a competitive growth advantage. Accordingly, an allele carrying a mutation that is beneficial would be selected for amplification, and/or the corresponding WT allele might be reduced or lost (e.g. via LOH). Both events increase proportional dosage of the advantageous mutation. In contrast, mutations that are biologically deleterious and/or immunogenic would be diluted by amplification of the complementary WT allele or loss of the mutated allele.

This model also predicts that mutations enriched in tumours as a consequence of AI will support the malignant phenotype. Consistent with this, we observed gain or increased dosage of numerous mutated genes in metastatic tumours in transition from diploid to hyperploid states. These genes notably included regulators of cell cycle progression and the maintenance of genomic integrity, which are manifestly perturbed in a high proportion of progressing melanomas. It will be important to validate these genes functionally as potential regulators of aneuploidy in melanoma that might inform therapeutic opportunities. Interestingly, stochastic deregulation of wild-type regulators of genomic integrity, such as the *MYC* amplifications we observed with concurrent *TP53* mutations, might compound enrichment of mutations that fuel aneuploidy.

Our data also raise the possibility that WGD and/or the enhanced tolerance it confers to continued acquisition of AI, might have prognostic significance in melanoma. Aneuploidy has been proposed by some to increase the probability of recurrence after treatment for early melanoma^62,63^. Moreover, the rate of acquisition of aneuploidy, as represented by subclonality of CNAs, was linked to survival outcomes in non-small cell lung cancer^30^. In this case, the reliable detectability we observed by ctDNA of dominant patterns of tetraploidization and aneuploidy in metastatic melanoma could indicate a useful, minimally invasive and prognostically important means of monitoring for key stages of disease evolution in individual melanoma patients.

## Methods

### Sample collection and DNA extraction

Recruitment to the CASCADE (CAncer tiSsue aCquisition After DEath rapid autopsy programme at the Peter MacCallum Cancer Centre followed approved protocols (PMCC Human Research Ethics Committee approval number 11/102). Informed written consent was obtained from all patients and families. Rapid autopsies were performed as previously described^22^. See Supplementary Methods for further details.

Formalin-fixed paraffin-embedded (FFPE) tissues were sectioned 20 times and the 1st, 10th and 20th sections underwent haematoxylin & eosin (H&E), Melan-A and S100 staining, respectively, for identification by an expert histopathologist of intra-tumoural regions of viable melanoma. Only samples that contained >70% tumour content were used for DNA extraction and sequencing. After macro-dissection and DNA extraction (QIAamp DNA FFPE Tissue Kit, Qiagen), each sample was assessed for DNA integrity^64^. In cases where adjacent fresh frozen samples were available for DNA extraction (DNeasy blood and tissue kit, Qiagen), tumour content was verified using digital PCR for *BRAF* mutations^65^. Genomic DNA from buffy coats (used as matching germline DNA) was extracted using the DNeasy blood and tissue kit (Qiagen). Plasma DNA was extracted from 1–2 ml of plasma (QIAamp Circulating Nucleic Acid Kit, Qiagen).

### Whole exome sequencing

100 ng to 1 ug of input DNA was fragmented using a Covaris S2 focal acoustic device to an average fragment size of 180–220 bp (duty cycle: 10%; intensity: 5; cycles per burst: 200; time: 3 min). Libraries were prepared using KAPA Hyper Prep Kits for Illumina, (Kapa Biosystems) with SureSelect XT adaptors and primers (Agilent Technologies). 6 or 9 PCR cycles were used for good quality or FFPE-derived DNA, respectively. Hybridisation capture was performed with SureSelect Human All Exon V5 or V6 (Agilent). Three indexed libraries were run per lane on an Illumina HiSeq2500 platform (paired-end 100 bp).

### Whole-genome sequencing

100 ng of input DNA was fragmented as above to an average size of 350 bp (duty cycle: 5%; intensity: 5; cycles per burst: 200; time: 50 seconds). Libraries were prepared using the TruSeq Nano DNA Library Preparation Kit (Illumina). Libraries were sequenced at The Kinghorn Centre for Clinical Genomics (Sydney, Australia) on a HiSeq X Ten (Illumina). One sample was sequenced per lane or over several lanes (paired-end 150 bp) to a depth of 30X or 60X for germline or tumour, respectively.

### Calling and validation of somatic SNVs and small indels

After trimming and quality filtering, sequencing reads were aligned to the human reference genome (hg19) using bwa mem, deduplicated, realigned and scores recalibrated. Reads with a mapping quality below 30 were removed. SNVs and small insertions/deletions (indels) were called using an ensemble approach with multiSNV^66^ (minimum average read coverage across samples of 10X), VarScan^67^ (minimum allele frequency of 10%), muTect^68^ and IndelGenotyper^69^. SNVs flagged as ‘pass’ and ‘normal contamination’ by multiSNV were kept for further processing. SNVs with an allele fraction >5% in the germline DNA were discarded. Variants in regions of low coverage (<10x), repetitive/artefactual regions, off-target regions (for WES), and that overlapped with healthy population databases were filtered out. Variants detected by at least two callers and passing all filters were kept for downstream analysis.

Given that the set of variants called for each patient was usually represented in multiple samples, the following steps were applied to optimise the reliability of variant calling. First, to exclude the possibility of absent variants due to insufficient depth of sequencing, variants were filtered out if the region was regarded as low coverage (<10x) in any sample of that patient, including the germline DNA. Second, to leverage the sensitivity of muTect in samples of low cellularity, variants absent in samples based on the procedure above but called only by muTect were included. Third, to leverage the sensitivity of multiSNV as a multi-sample variant caller, the presence of variants detected as above was confirmed in other samples from the same patient via a lenient execution of VarScan (i.e. allowing variant allele frequencies <10%, minimum coverage of 10x and variant present in ≥2 reads).

Mutational signatures were estimated using deconstructSigs^70^ with the COSMIC v3 signatures set^71^. Validation of SNVs and small indels was performed using the 48.48 Access Array system (Fluidigm). See Supplementary Methods for further details.

### Somatic copy number alteration and structural variant calling

We defined structural variants (SVs) as mutations that involved a segment of DNA ( > 50nt) differing between a tumour sample and the normal sample, caused by one or more double stranded DNA breaks. These included insertions, deletions, tandem or inverted duplications, inversions, translocations or copy number alterations (CNAs). When the evidence of an SV was derived from CNA data alone, we used the term CNA.

Estimates of allele-specific copy number, tumour cellularity and ploidy within each sample were obtained using Sequenza^72^ (v2.1.0), followed by manual verification. Large SVs (>1000nt) were detected using the GRIDSS (Genomic Rearrangement Identification Software Suite; v1.3.2)^73^. See Supplementary Methods for further details.

### Phylogenic analysis

MEDICC^74^ (downloaded July 2014) was used to infer phylogenies from allele-specific copy number calls summarised at the cytogenetic band level. To infer phylogenies from SNVs, a binary matrix of mutations excluding regions of shared LOH was constructed for each patient. To infer phylogenies from all mutations, a character-weighted binary supermatrix was constructed. Maximum parsimony phylogenies were constructed using TNT (v1.5) from these matrices^75^. See Supplementary Methods for further details.

### Copy number validation

Fluorescence in situ hybridisation (FISH) was performed for six CASCADE patients on FFPE slides to validate copy number results. *Myc*/CEP8 and *CCND1*/CEP11 probes were used to validate allelic amplifications and *CDKN2A*/CEP9 probes were used to validate allelic losses. For each slide, the area to be scored was identified at low (10x) magnification using a DAPI filter. A minimum of 100 cells from at least three random fields was scored for the number of probe-specific alleles (red) and centromere (green) signals at high (100x) magnification.

### Inference of genome doubling

To test if the patterns of increased ploidy were due to stochastic aneuploidy alone (stochastic gains of individual chromosomes) or if WGD also played a role, two branching process-based models were fitted to the data. The first modelled aneuploidy as the stochastic breakage and mis-segregation of chromosomes at the arm-level. The second modelled aneuploidy in the same way, together with a single WGD event.

The evolutionary history of each tumour genome was divided into *N* arbitrary time periods. Each copy of each arm was assigned a fixed probability α of undergoing a deletion, β of not changing, and γ of being duplicated within each time period, and was subject to the constraint that α+β+γ=1. The WGD model included a single WGD event after *M* time intervals (M ≤ N). We used probability generating functions to algebraically construct the probabilities of every possible copy number state as polynomials depending on α, β, γ and *N* for the model with no WGD and α, β, γ, N and *M* for the model with WGD. The generating functions grow exponentially and are prohibitively large for *N* and *M* much greater than about 5. To construct the generating function, we developed an efficient program in the sage language (http://www.sagemath.org/) and utilised memoization to reduce computational time and memory consumption. This program only needed to be run once. We selected *N* = 6, permitting arm-level copy numbers up to 256 to be modelled.

The parameters α, β, γ, and *M* for the two models were estimated for each patient using maximum likelihood. To determine which model provided the most likely explanation of the observed data, while correcting for the additional parameter in the WGD model, we used the Akaike Information Criterion (AIC)^76^. Unlike other information criteria, AIC does not rely upon any assumptions contradicted by the model. In general, the model with WGD has 3 free parameters, while the model without WGD has 2. In this case we also imposed α = γ, leading to 2 and 1 free parameters respectively. We applied the more general formula, leading to a small constant offset of +2 to both AIC_WGD_ and AIC_non-WGD_, which does not affect the relative likelihood or choice of best-fitting model.

Notably, our approach makes several assumptions that may limit its applicability: the only events modelled are WGD and arm-level copy number change (which are treated as independent events); a whole chromosome gain or loss is modelled as a pair of independent arm-level events; focal amplification and complex events are not included in the model; and resolution of event order is limited to the N = 6 arbitrary time periods.

Code and additional details regarding the test are available at https://github.com/PapenfussLab/Genome_doubling_test.

### Reporting summary

Further information on research design is available in the Nature Research Reporting Summary linked to this article.

## Supplementary information

Supplementary Information

Reporting Summary

## Data Availability

Whole exome and genome sequencing data for CASCADE patients have been deposited in the European Genome-Phenome Archive (EGA) under the study ID EGAS00001004950. These data are available on request to the Peter MacCallum Cancer Centre Data Access Committee (dac@petermac.org). Whole exome sequencing data from patients MI-F, SK-G and SK-H^19^ are available via the National Center for Biotechnology Information database of Genotypes and Phenotypes (NCBI dbGaP accession p). Whole exome sequencing data from patients ETH-E, ETH-F and ETH-J^21^ are available on request to Mitchell Levesque (Mitchell.Levesque@usz.ch). These patients did not give their consent for the public availability of their raw sequencing data. The public release of the data was not permitted at the time of collection under Swiss law. Repeat annotations from Repeatmasker (http://www.repeatmasker.org/), known cancer genes from COSMIC (https://cancer.sanger.ac.uk/cosmic), variants from EVS (http://evs.gs.washington.edu/EVS) and 1000 Genomes (http://ftp.1000genomes.ebi.ac.uk/), and pathway information from Reactome (https://reactome.org/) were also referenced in the study. All other data are available within the Article, Supplementary Information or from the authors upon request.

## Code availability

All code developed for this in this study is publicly available: analysis pipeline (https://github.com/PapenfussLab/CascadePipe), genome doubling test (https://github.com/PapenfussLab/Genome_doubling_test), and neoantigen analysis pipeline (https://github.com/PapenfussLab/CascadeNeoPipe).
