# Supplementary material for: Evolution of late-stage metastatic melanoma is dominated by aneuploidy and whole genome doubling
Source: Nat Commun. 2021 Mar 4;12:1434. doi: 10.1038/s41467-021-21576-8 (PMC7933255; doi:10.1038/s41467-021-21576-8)
Supplement: Supplementary file 1 — Supplementary Information [file 41467_2021_21576_MOESM1_ESM.pdf]

Evolution of late-stage metastatic melanoma is dominated by aneuploidy and whole genome doubling

Ismael A Vergara, Christopher P Mintoff, Shahneen Sandhu, Lachlan McIntosh, Richard J Young, Stephen Q Wong, Andrew Colebatch, Daniel L Cameron, Julia Lai Kwon, Rory Wolfe, Angela Peng, Jason Ellul, Xuelin Dou, Clare Fedele, Samantha Boyle, Gisela Mir Arnau, Jeanette Raleigh, Athena Hatzimihalis, Pacman Szeto, Jennifer Mooi, Daniel S Widmer, Phil F Cheng, Valerie Amann, Reinhard Dummer, Nicholas Hayward, James Wilmott, Richard A Scolyer, Raymond J Cho, David Bowtell, Heather Thorne, Kathryn Alsop, Stephen Cordner, Noel Woodford, Jodie Leditschke, Patricia O'Brien, Sarah-Jane Dawson, Grant A McArthur, Graham J Mann, Mitchell P Levesque, Anthony T Papenfuss[*,†] and Mark Shackleton[*,†]

[*]Co-senior authors, contributed equally and are listed in alphabetical order.

[†]Co-corresponding authors: Anthony T Papenfuss (papenfuss@wehi.edu.au) and Mark Shackleton (mark.shackleton@monash.edu)

SUPPLEMENTARY INFORMATION

Table of contents

Supplementary Figures 3
Supplementary Notes 34
Experimental validation of somatic single nucleotide variants (SNVs) and small insertions/deletions (InDels) 34
Comparison of SNV calling from DNA extracted from FFPE vs FF tumour material... 35
Comparison of CNV-based, SNV-based and joint phylogenetic inference 36
Comparison of MEDICC, SNV-based and joint-evidence phylogenies 38
Comparison with Sanborn et al phylogenies 39
Supplementary Methods 41
Pre-autopsy tumor material 41
Autopsy procedure 41
Manual microdissection of patient tumour sections. 41
Genomic DNA Extraction 41
DNA Quantitation 42
Whole Exome Sequencing 43
Whole Genome Sequencing 43
Targeted Amplicon sequencing validation 43
Somatic Single Nucleotide Variant and small Insertion/Deletion detection 44
Mutational signatures, melanoma and cancer-associated genes, chromosomal instability and genomic integrity-associated genes 45
Neoantigen prediction and predicted expression in TCGA cohort 46
Characterisation of variants deleted due to loss of heterozygosity 47
Somatic copy number alterations detection and CNV-based phylogeny 49
Structural variant detection 49
Fluorescence In-Situ Hybridisation 50
Supplementary References 51
Supplementary Tables 54

Supplementary Figures

FIGURE S1

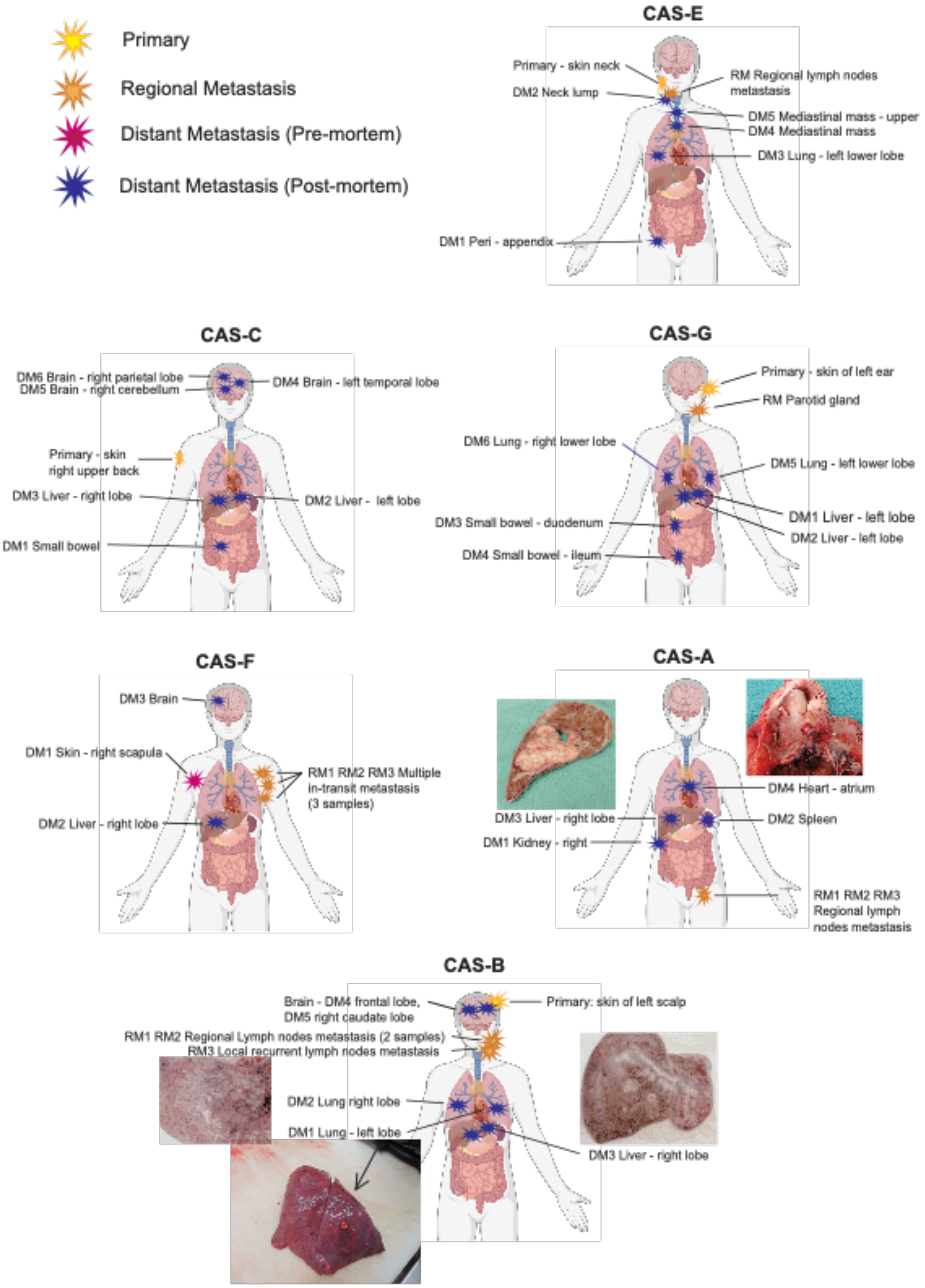

Supplementary Figure 1. Spatial distribution of pre-mortem and autopsy samples for six CASCADE patients. CASCADE patient CAS-D is shown in Figure 1A. The individual figures were created with BioRender.com. See Table S1 for sample details of samples.

FIGURE S2

(A)

Case	Mutation	Primary Site	Histo Subtype	TNM	Breslow	Mit Count	Ulceration
**CAS-D**	BRAF V600E	Back	NM	T4N3M1	4.7	2	NO
**CAS-G**	BRAF V600E	Ear	SSM	T4bN2cM0	7	5	YES
**CAS-C**	BRAF K601E	Back	SSM	T2aN0M0	1.9	<1	NO
**CAS-E**	BRAF V600E	Neck	NM	T4NxM1b	7.6	20	NO
**CAS-A**	BRAF V600E	Thigh	SSM	T1bN0M0	1	1	YES
**CAS-F**	BRAF V600E	Neck	NM	T4N3M0	15	15	YES
**CAS-B**	BRAF V600E	Scalp	Not reported	T2aN0M0	1.35	10	NO

(B)

Case	Mutation	Primary Site	Histo Subtype	TNM	Breslow	Mit Count	Ulceration
**MI-F**	BRAF/NRAS/NF1 WT	Ear	NM	T4aN3M0	12	19	NO
**SK-G**	NRAS Q61K	Forearm	NA	NA	12	11	NO
**SK-H**	BRAF/NRAS/NF1 WT	Leg	NA	NA	35	4	YES
**ETH-E**	BRAF V600E	Spine	Melanoma Ex-Naevo	T3N1aM0	3.8	NA	NO
**ETH-F**	BRAF/NRAS/NF1 WT	Scapula	SSM	T4bN3M0	17	>1	YES
**ETH-J**	NRAS Q61R	Scapula	NM	T4bN1bM0	5	NA	YES

Supplementary Figure 2. Clinical data for (A) CASCADE and (B) external cohorts patients. External cohorts correspond to those reported by Raaijmakers *et al* [1] and Sanborn *et al* [2]. See Tables S1 and S2 for sample details.

FIGURE S3

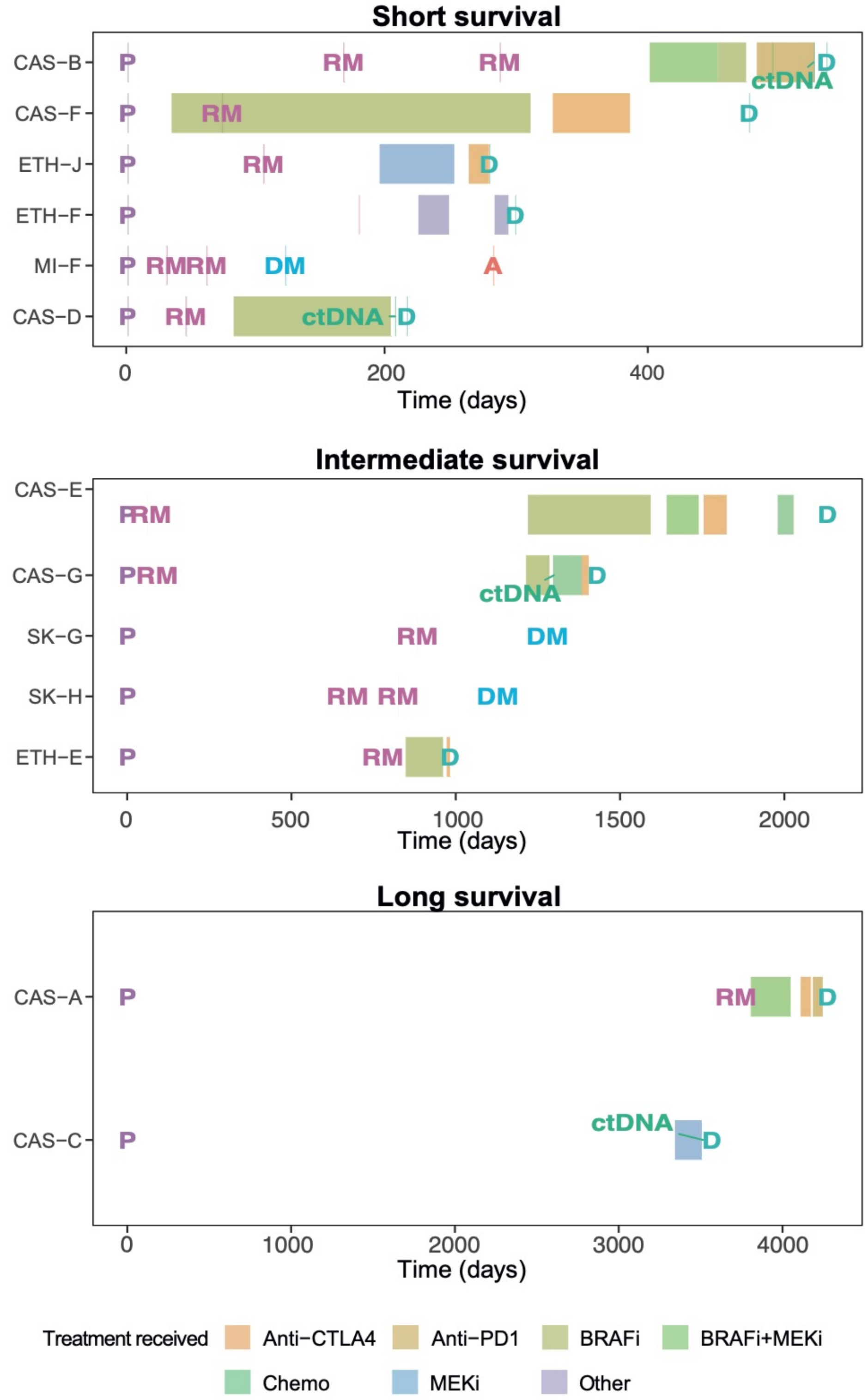

Short survival
CAS−B
CAS−F
ETH−J
ETH−F
MI−F
CAS−D
P
RM
RM
ctDNA
D
P
RM
D
P
RM
D
P
D
P
RMRM
DM
A
P
RM
ctDNA
D
0
200
400
Time (days)
Intermediate survival
CAS−E
CAS−G
SK−G
SK−H
ETH−E
PRM
D
PRM
ctDNA
D
P
RM
DM
P
RM RM
DM
P
RM
D
0
500
1000
1500
2000
Time (days)
Long survival
CAS−A
CAS−C
P
RM
D
P
ctDNA
D
0
1000
2000
3000
4000
Time (days)
Treatment received
Anti−CTLA4
Anti−PD1
BRAFi
BRAFi+MEKi
Chemo
MEKi
Other

Supplementary Figure 3. Timeline of disease progression and treatment across 13 patients in the cohort, whose survival after initial diagnosis of primary melanoma was either short, intermediate or long. Treatments received are colour coded. P: Primary Tumour RM: Regional Metastasis. DM: Distant Metastasis. ctDNA: circulating tumour DNA from plasma sample. A: Alive. D: Deceased. Status at last follow up not available for patients SK-G and SK-H.

FIGURE S4

CAS-G DM6-FF	Ubiquitous (FFPE)	Shared (FFPE)	Private (FFPE)	Not Called (FFPE)
**Ubiquitous (FF)**	601	0	0	7
**Shared (FF)**	0	96	20	18
**Private (FF)**	0	7	1303	**569**
**Not Called (FF)**	4	37	**987**	0

Columns: CAS-G DM6-FFPE

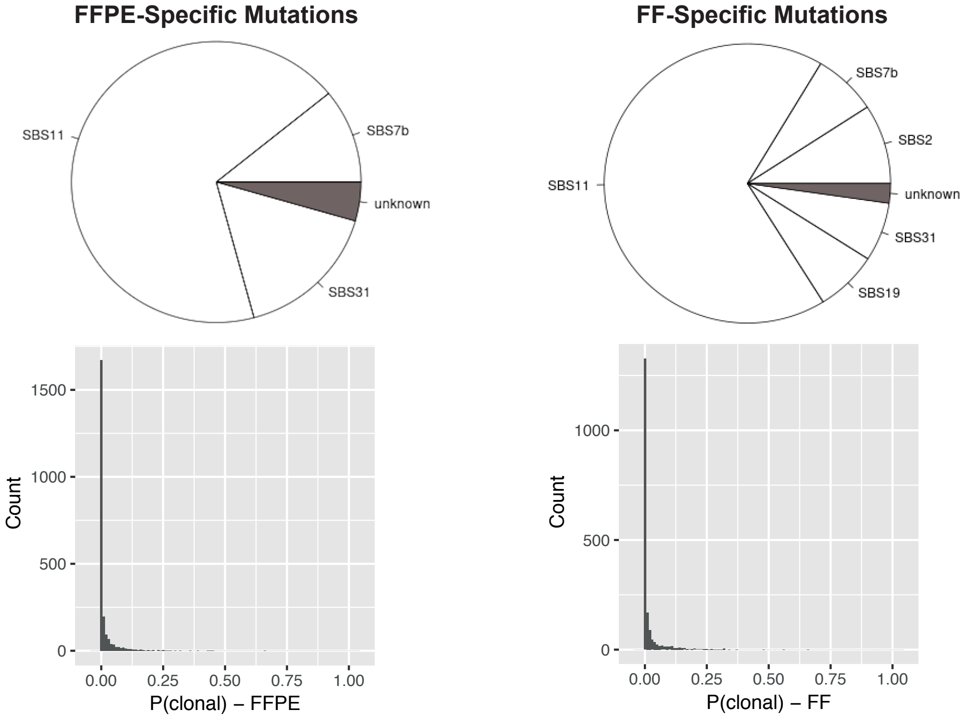

Supplementary Figure 4. Comparison of single nucleotide variant (SNV) and small insertions/deletions (indels) calling by analysis of ubiquitous, shared and private mutations in a matched fresh frozen (FF) tissues sample and its adjacent formalin-fixed paraffin embedded (FFPE) counterpart from the same metastasis (DM6-lung) from patient CAS-G. Contingency table analysis (upper) of distribution of SNV and small indel calls from FF vs FFPE samples. Mutational signatures associated with private mutations specific to each sample (middle pie charts) were assessed with deconstructSigs[3] using the COSMIC set of signatures. The probability of clonality of private mutations in each sample (bottom panels) was calculated as reported[4].

FIGURE S5

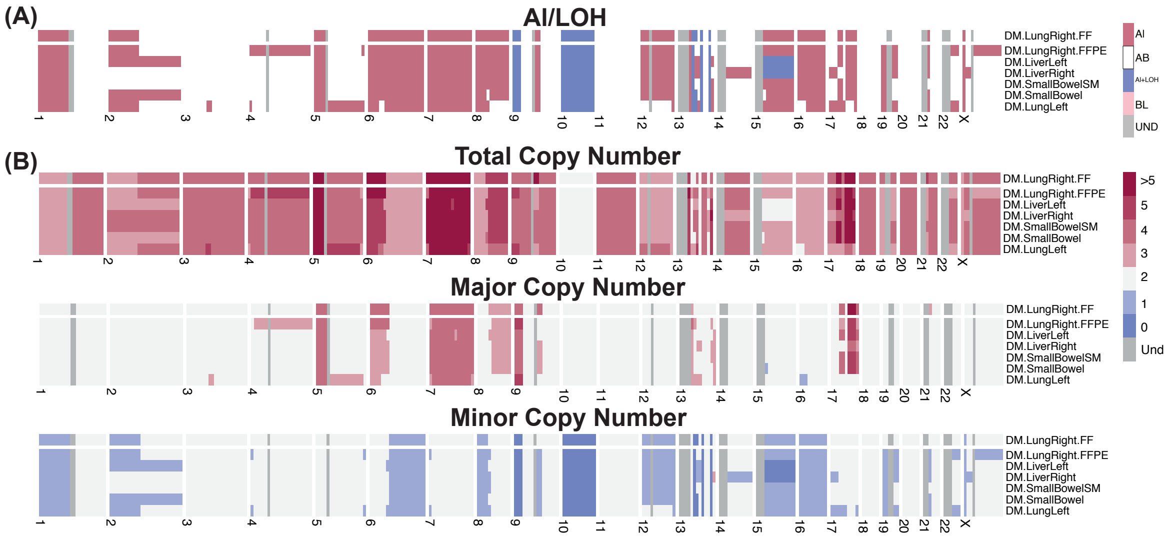

Supplementary Figure 5. Comparison of cytogenetic band-level allelic state calling (A) and total, major and minor copy number (CN) profile calling (B) in a matched fresh frozen (FF) tissues sample and its adjacent formalin-fixed paraffin embedded (FFPE) counterpart from the same metastasis (DM6-lung) from patient CAS-G. Regions of allelic balance (AB) have the same paternal and maternal allelic CN. Regions of allelic imbalance (AI) have different allelic CN. Undetermined (UND) regions are indicated in grey. The calculated CN is indicated in legend to right; regions with total CN >5 share the same colour. LOH: loss of heterozygosity, BL: biallelic loss. DM: distant metastasis.

FIGURE S6

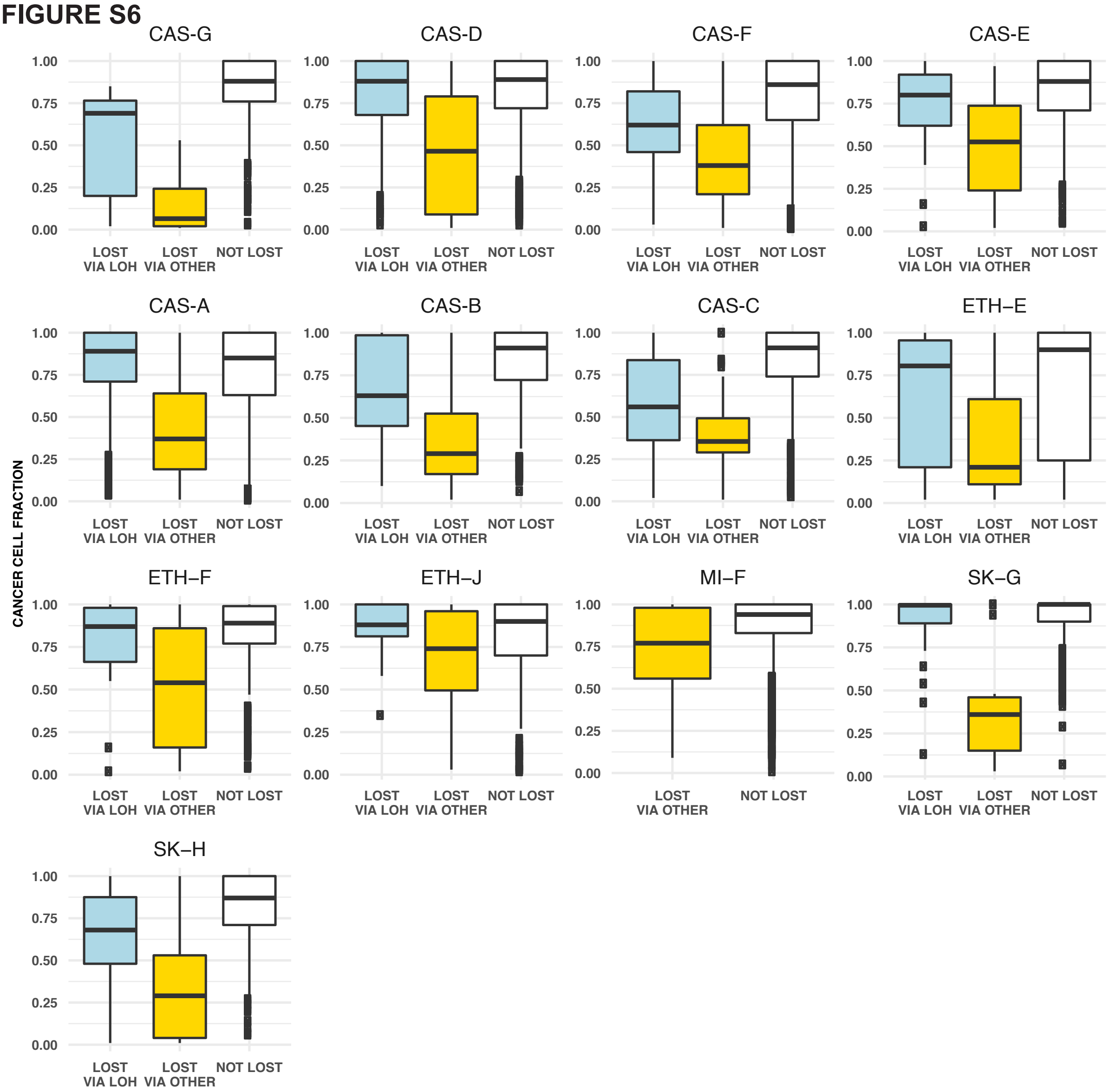

Supplementary Figure 6. Distribution of the cancer cell fractions (CCF) in the early stage disease of each patient. The box (hinges) shows the first and third quartiles (Q_1 *and* Q_3 respectively). The whiskers extend from the hinges $\pm 1.5\ (Q_3 - Q_1)$. Outliers are drawn as square dots. The three groups are:

Not Lost: mutations not lost in any late stage lesion. *Lost via LOH*: mutations lost in at least one late stage lesion, co-occurring with the acquisition of an LOH event. *Lost via Other*: mutations lost in at least one late stage lesion, with no LOH acquisition. CCF was defined for each somatic mutation as the fraction of tumour cells in the sample that carry the mutation and estimated as described in Supplementary Methods.

FIGURE S7

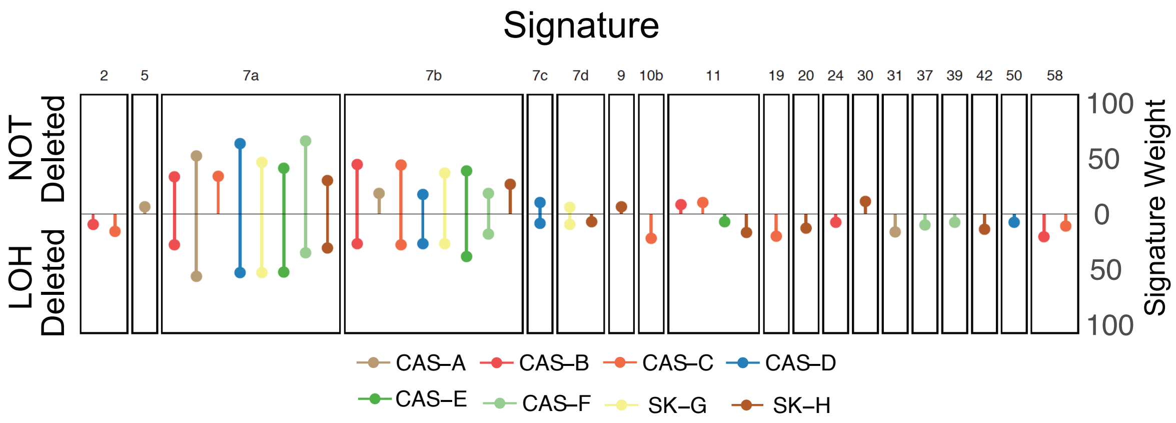

Supplementary Figure 7. Mutational signature distribution of *Lost via LOH* mutations in the early stage disease of each patient. *Not Lost*: mutations not lost in any late stage lesion. *Lost via LOH*: mutations lost in at least one late stage lesion, co-occurring with the acquisition of an LOH event. Patients CAS-G, ETH-E, ETH-F, ETH-J and MI-F were excluded due to low number of mutations in the *Lost via LOH* group.

FIGURE S8

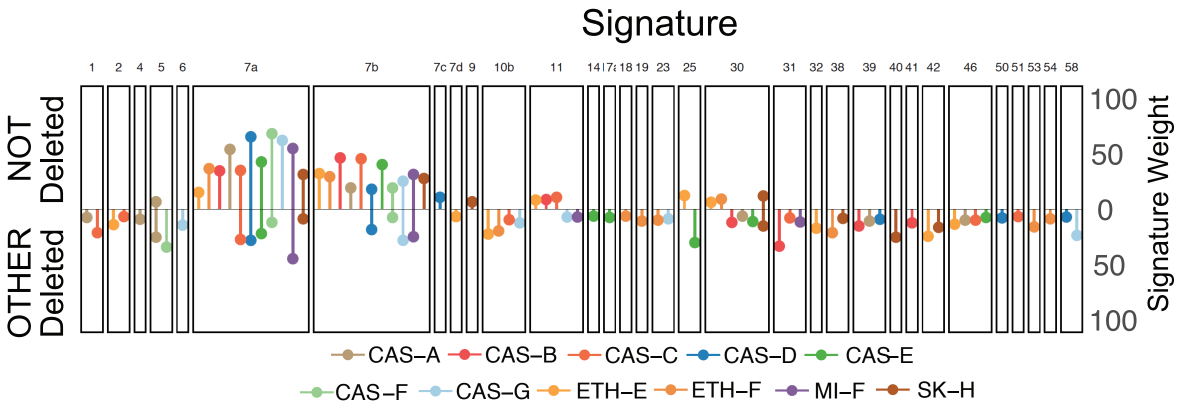

Supplementary Figure 8. Mutational signature distribution of *Lost via Other* mutations in the early stage disease of each patient. *Not Lost*: mutations not lost in any late stage lesion. *Lost via Other*: mutations lost in at least one late stage lesion that can't be explained due to LOH. Patients SK-G and ETH-J were excluded due to low number of mutations in the *Lost via Other* group.

FIGURE S9

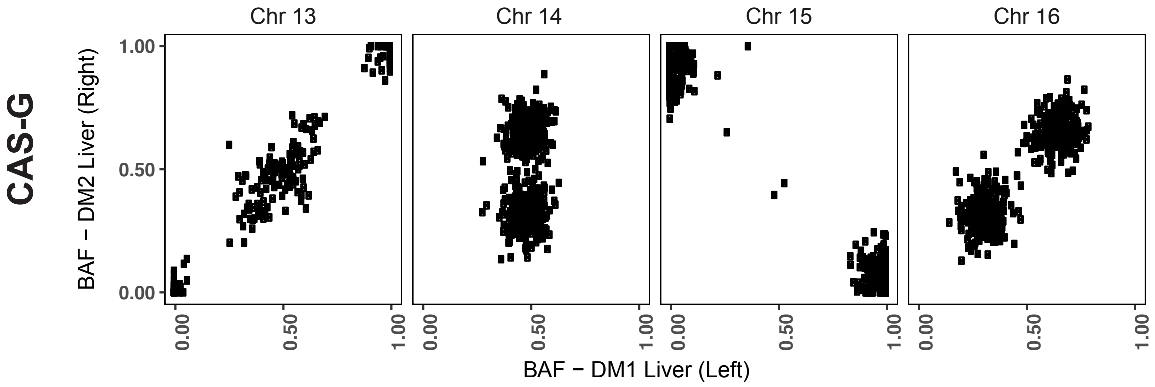

Supplementary Figure 9. B-allele frequencies of single nucleotide polymorphisms in two distant liver metastases in patient CAS-G. A mirrored imbalance of heterozygous SNPs on chromosome 15 was evident between these lesions.

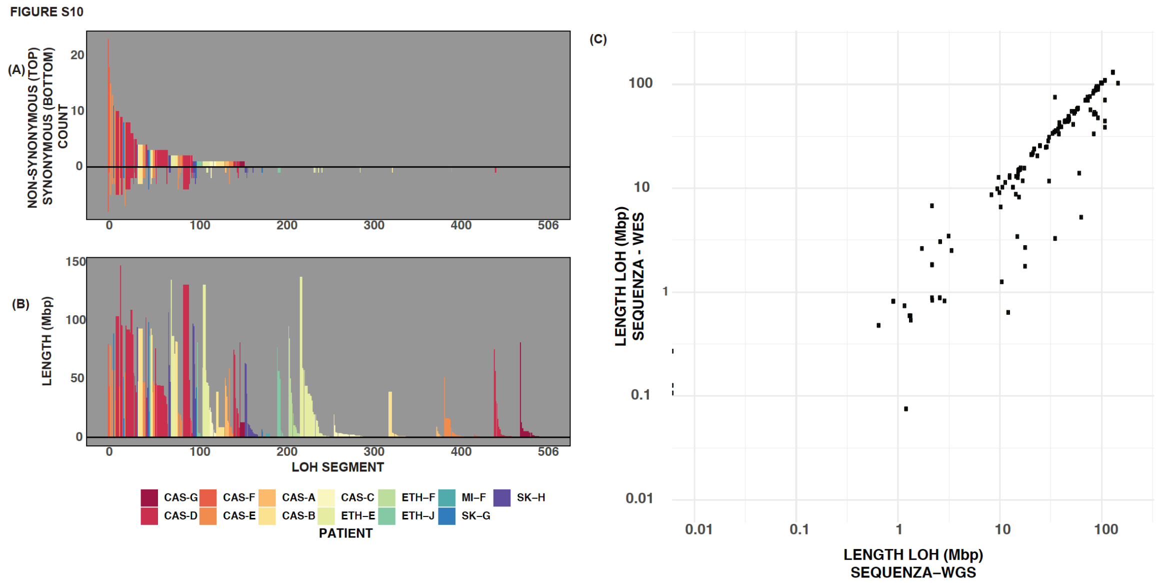

Supplementary Figure 10. Deletion of non-synonymous in late-stage melanoma by loss of heterozygosity. (A) Number of non-synonymous and synonymous variants lost due to each LOH segment. Color denotes patient. (B) Length of each LOH segment. (C) Lengths of corresponding LOH segments in WES and WGS data from patient CAS-D.

FIGURE S11

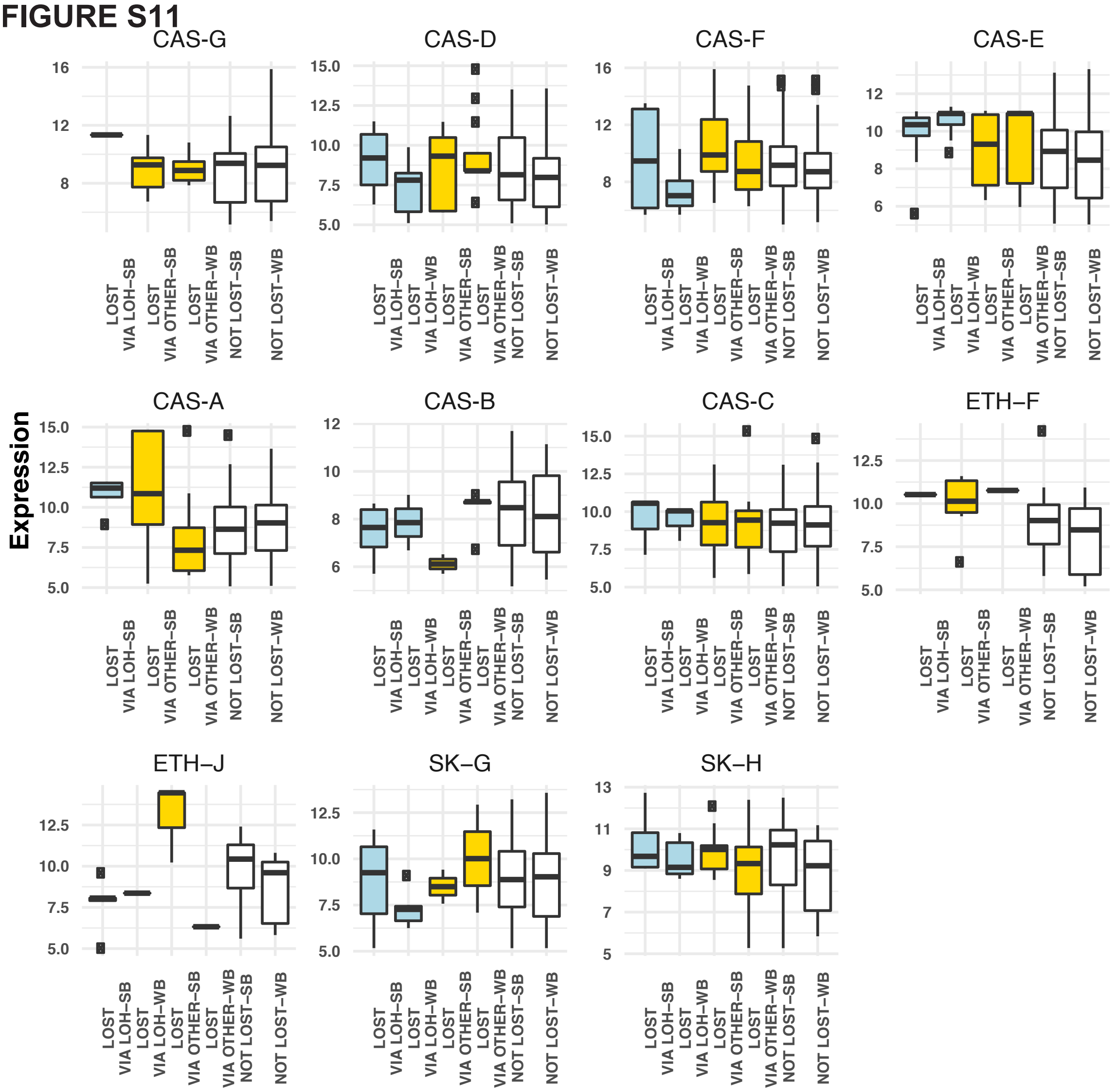

Supplementary Figure 11. Distribution of gene expression based on TCGA SKCM cohort (see Supplementary Methods) for predicted neoantigenic mutations. The box (hinges) shows the first and third quartiles (Q_1 *and* Q_3 respectively). The whiskers extend from the hinges $\pm 1.5\ (Q_3 - Q_1)$. Outliers are drawn as square dots. Groups are further separated into mutations with estimated strong binding affinity (SB) and weak binding affinity (WB).

FIGURE S12

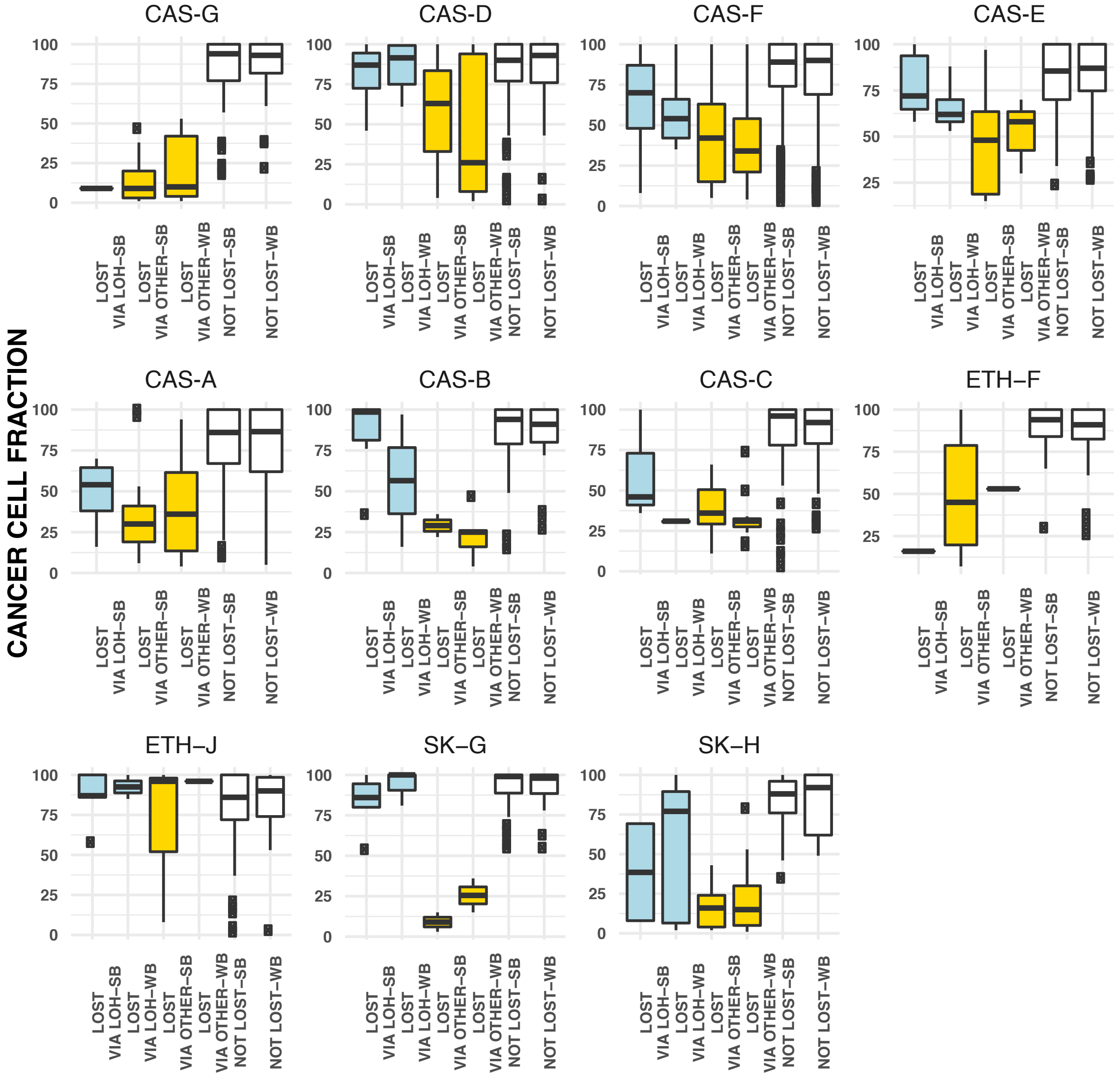

Supplementary Figure 12. Distribution of neoantigen cancer cell fractions. The box (hinges) shows the first and third quartiles (Q_1 and Q_3 respectively). The whiskers extend from the hinges $\pm 1.5\ (Q_3 - Q_1)$. Outliers are drawn as square dots. Groups are further separated into mutations with estimated strong binding affinity (SB) and weak binding affinity (WB).

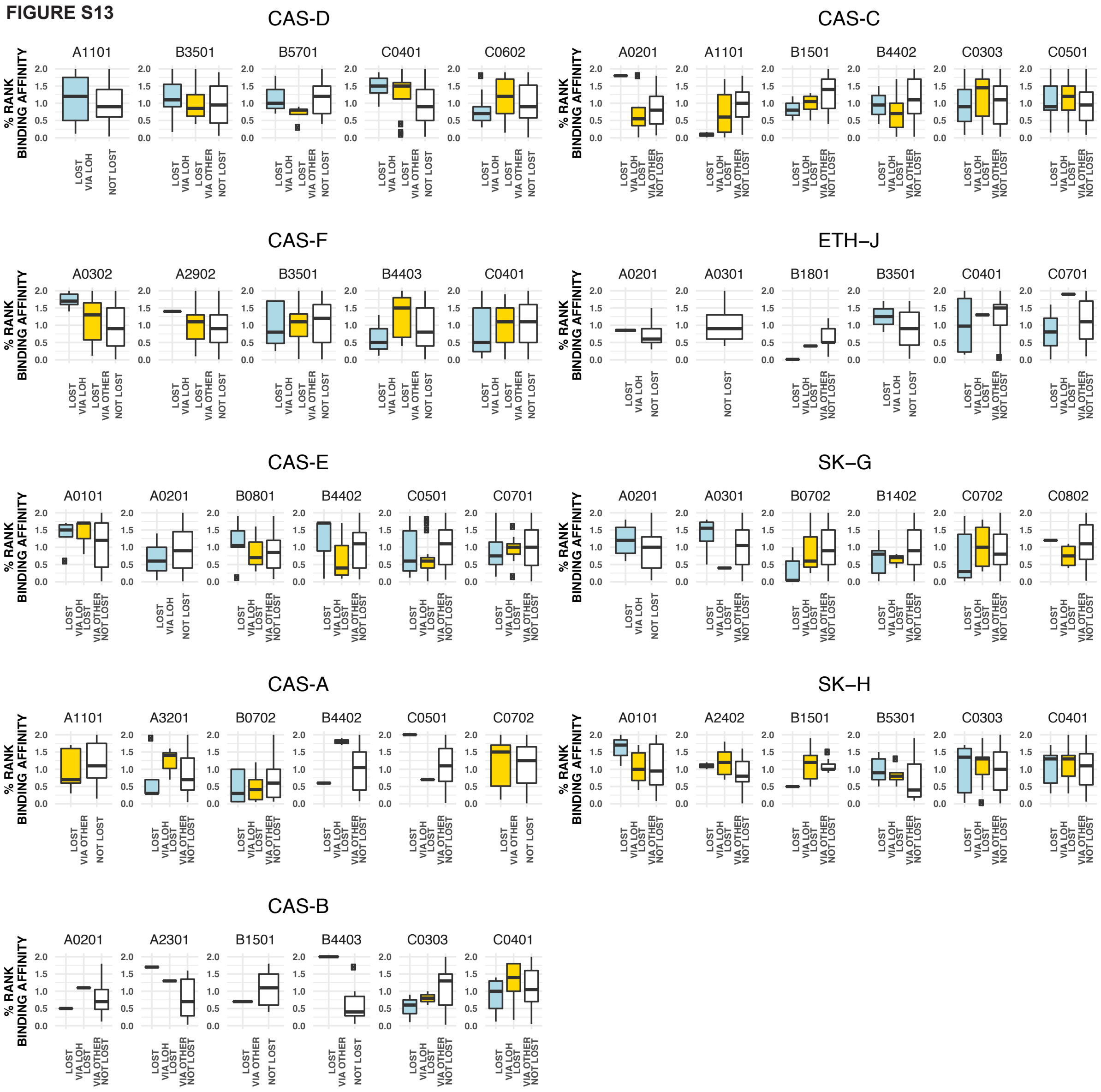

Supplementary Figure 13. Binding affinity rank distribution among groups of neoantigenic mutations. Each plot represents the distribution for each HLA-allele in a given patient. A lower %Rank Binding Affinity indicates increased binding affinity. The box (hinges) shows the first and third quartiles (Q_1 *and* Q_3 respectively). The whiskers extend from the hinges $\pm 1.5\ (Q_3 - Q_1)$. Outliers are drawn as square dots.

FIGURE S14

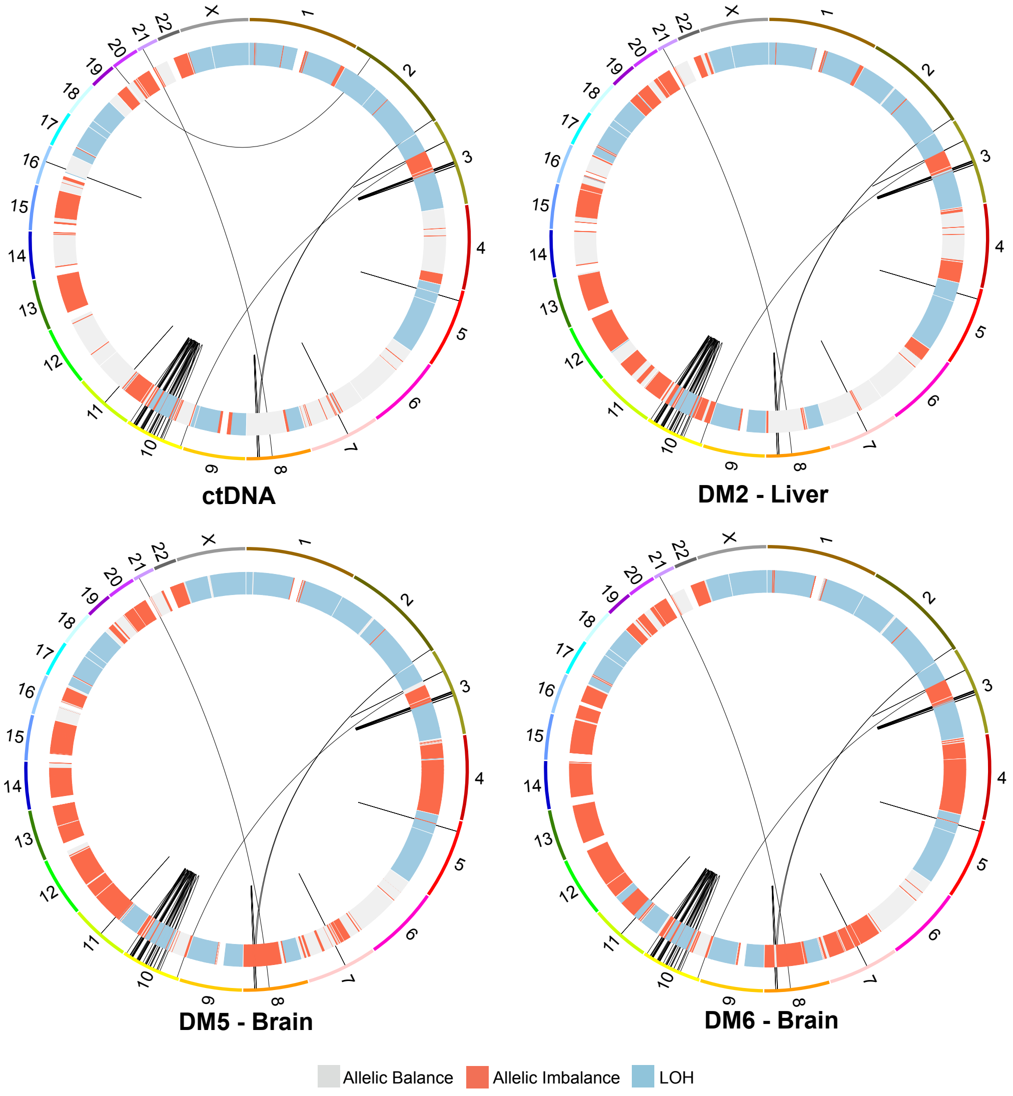

Supplementary Figure 14. Circos plots of structural variants (SVs) detected in distant metastases and plasma ctDNA for patient CAS-D. Inner track indicates the allelic state for each chromosome.

FIGURE S15

CAS-D

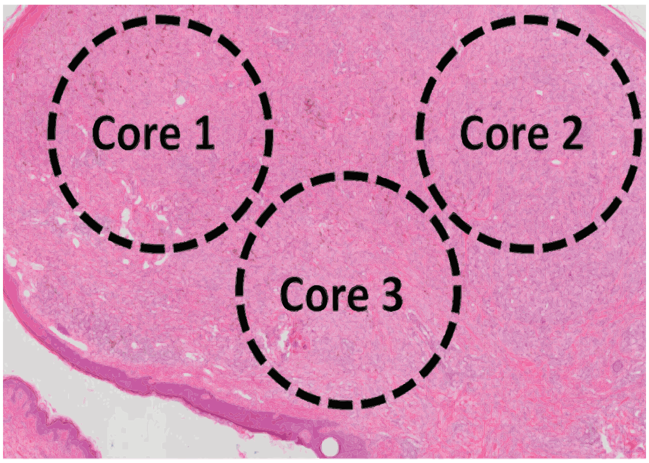

Primary Tumour

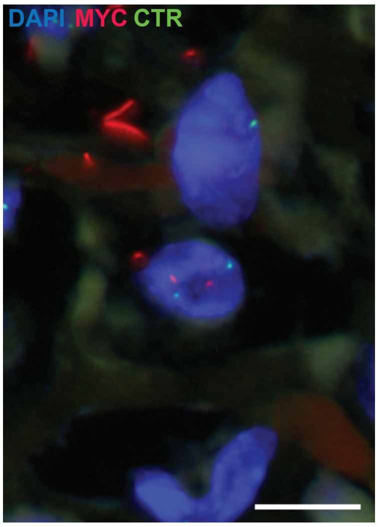

Core 1
MYC Average Count: 2.3
MYC Prediction: 2
CTR Average Count: 1.36
CTR Prediction: 2

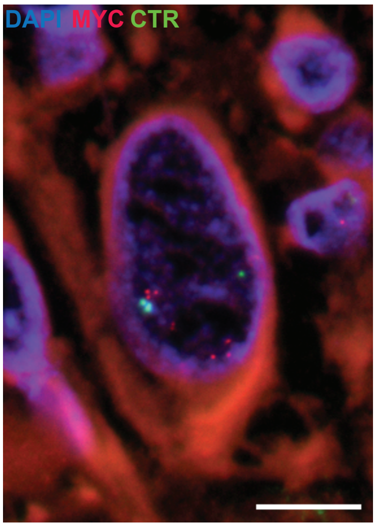

Core 2
MYC Average Count: 3.92
MYC Prediction: 4
CTR Average Count: 2.86
CTR Prediction: 3

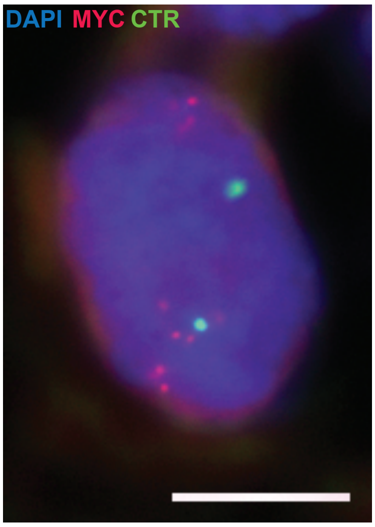

Distant Metastasis Central Liver
MYC Average Count: 6.71
MYC Prediction: 7
CTR Average Count: 2.81
CTR Prediction: 4

Supplementary Figure 15. Comparison of methods of copy number (CN) estimation in two primary melanoma cores and a liver metastasis from patient CAS-D. Average FISH counts and predicted CNs for *MYC* and centromere (CTR; chromosome 8) are provided. Counts for each primary core were calculated over 50 randomly chosen cells. Counts for the liver metastasis (DM3) were calculated over 100 cells. Scale bar = 10um.

FIGURE S16

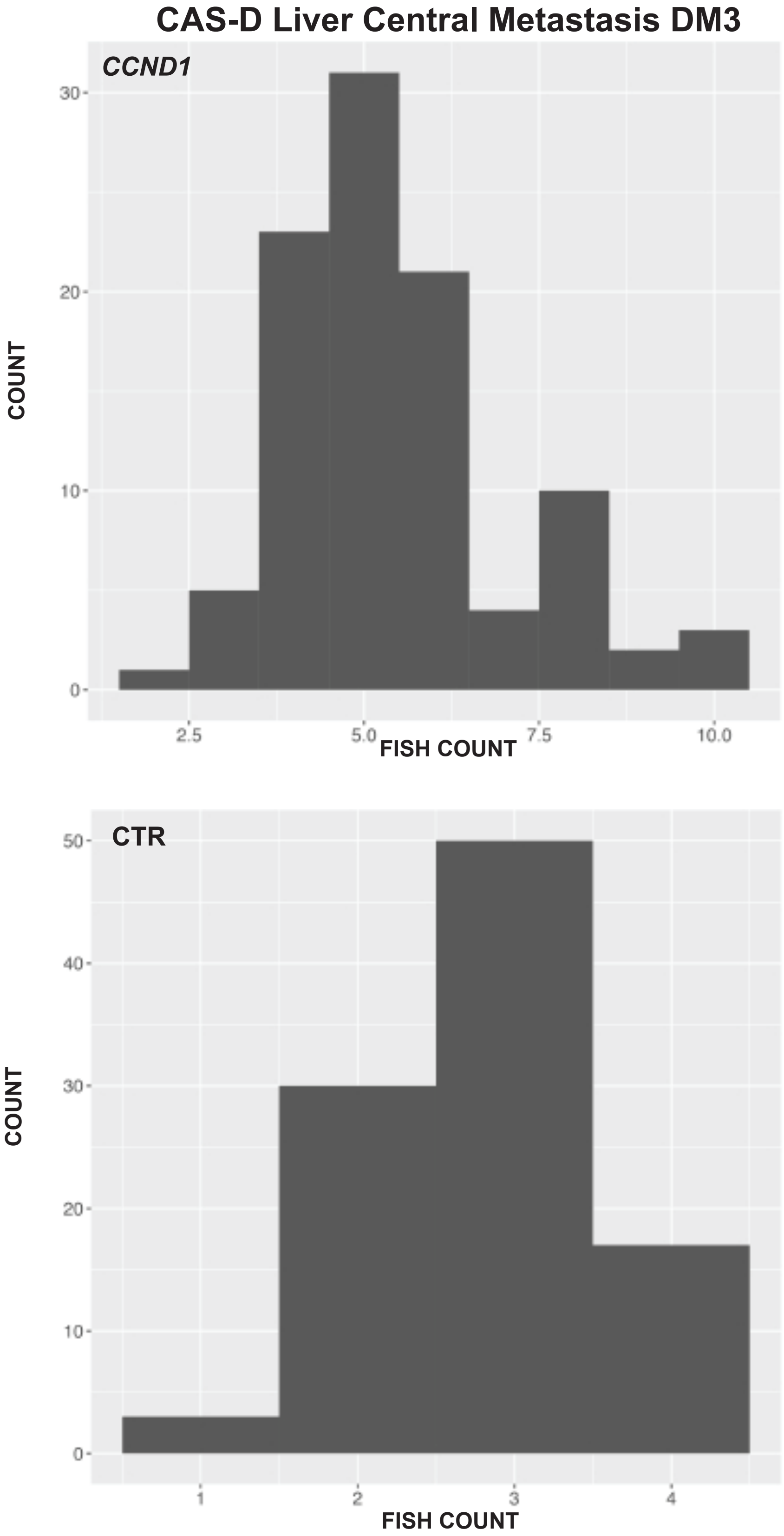
CAS-D Liver Central Metastasis DM3
CCND1
COUNT
FISH COUNT
0
10
20
30
2.5
5.0
7.5
10.0
CTR
COUNT
FISH COUNT
0
10
20
30
40
50
1
2
3
4

Supplementary Figure 16. Distribution of counts of FISH probes against *CCND1* (top) and the chromosome 11 centromere (CTR, bottom) in a liver metastasis of patient CAS-D. Y axis indicates number of cells (of a total of 100) with the indicated number of copies of these probes.

FIGURE S17

CAS-C

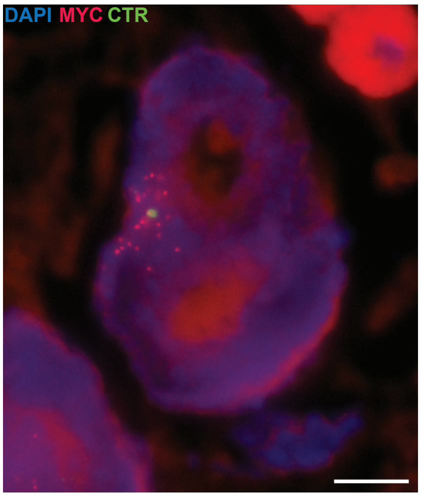

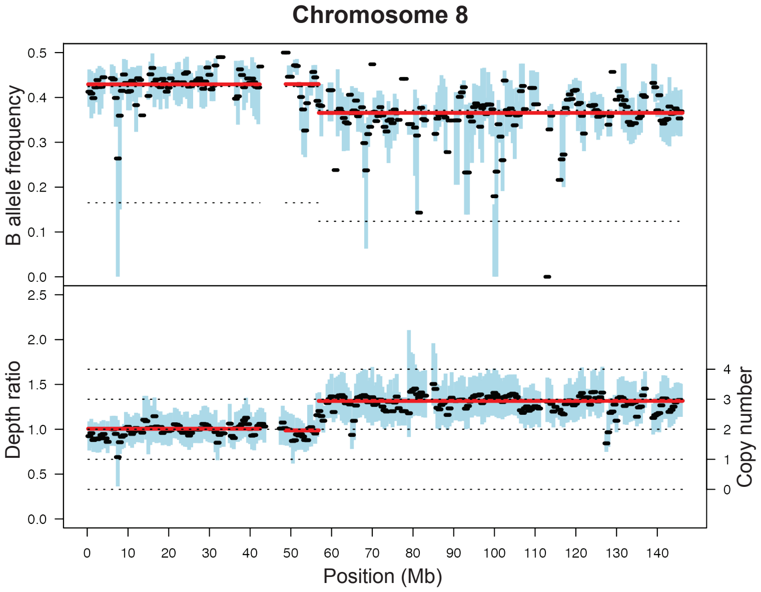

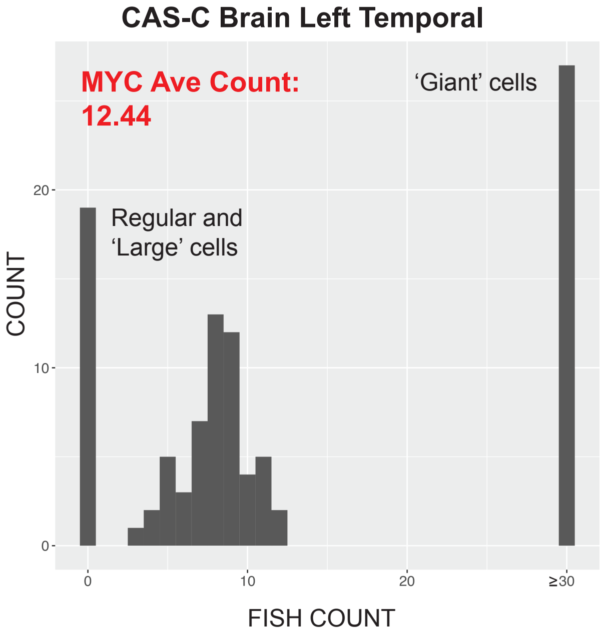

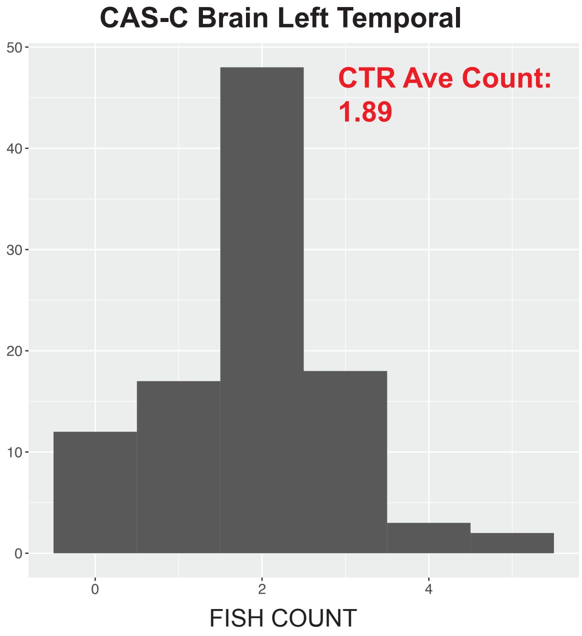

Supplementary Figure 17. Subclonality, *MYC* amplification and giant cells in a brain metastasis (DM4) from patient CAS-C. Upper left: Identification by FISH in a giant cell of *MYC* amplification using probes for *MYC* and the chromosome 8 centromere (CTR). **Upper right:** B-allele frequencies and depth ratio for chromosome 8 as generated by Sequenza. **Lower:** Distributions show the number of cells (of a total of 100) with the indicated number of copies of probes for *MYC* (left) and the chromosome 8 centromere (CTR, right). Scale bar = 10um.

FIGURE S18

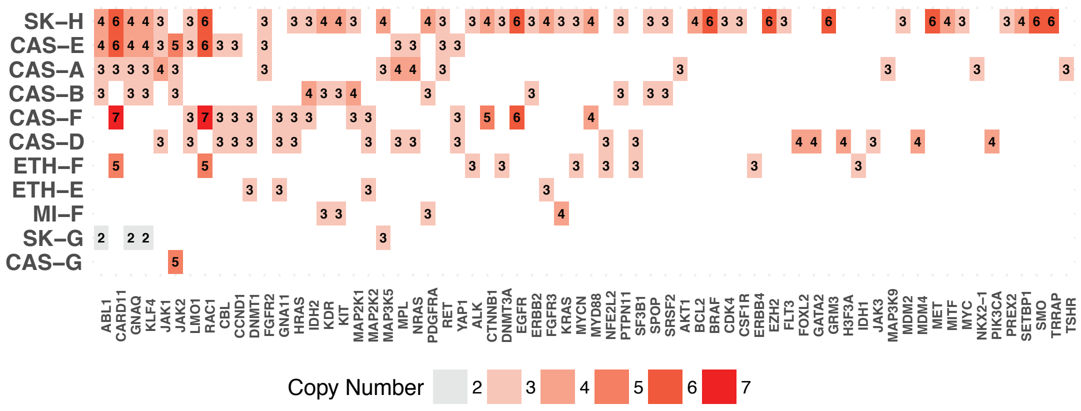

Supplementary Figure 18. Oncogene amplification in regions of LOH. Systematic identification of regions with LOH and amplification of the alternative allele not due to genome doubling alone. Regions have an allele-specific copy number of (n, 0), n≥2 in the absence of genome doubling, and n≥3 following genome doubling. For each patient, if an oncogene was found amplified in two or more samples, the maximum copy number value among those samples is displayed.

FIGURE S19

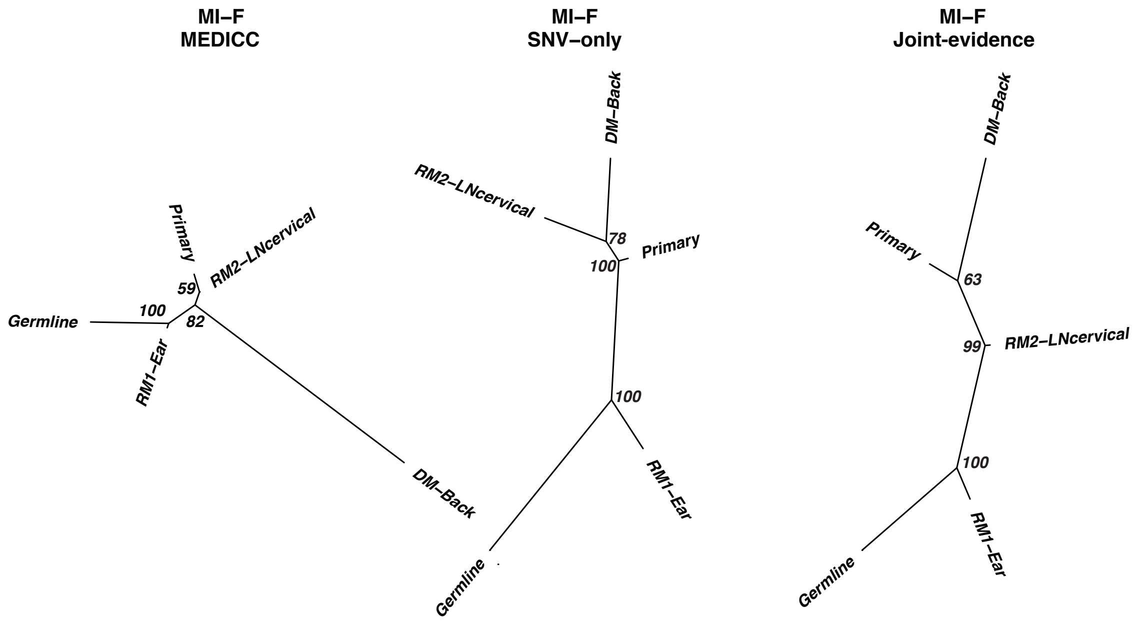

Supplementary Figure 19. MEDICC, SNV-only and joint-evidence phylogenies for patient MI-F. Germline branch length for the SNV-only and equally-weighted trees was truncated to 100 for better representation.

FIGURE S20

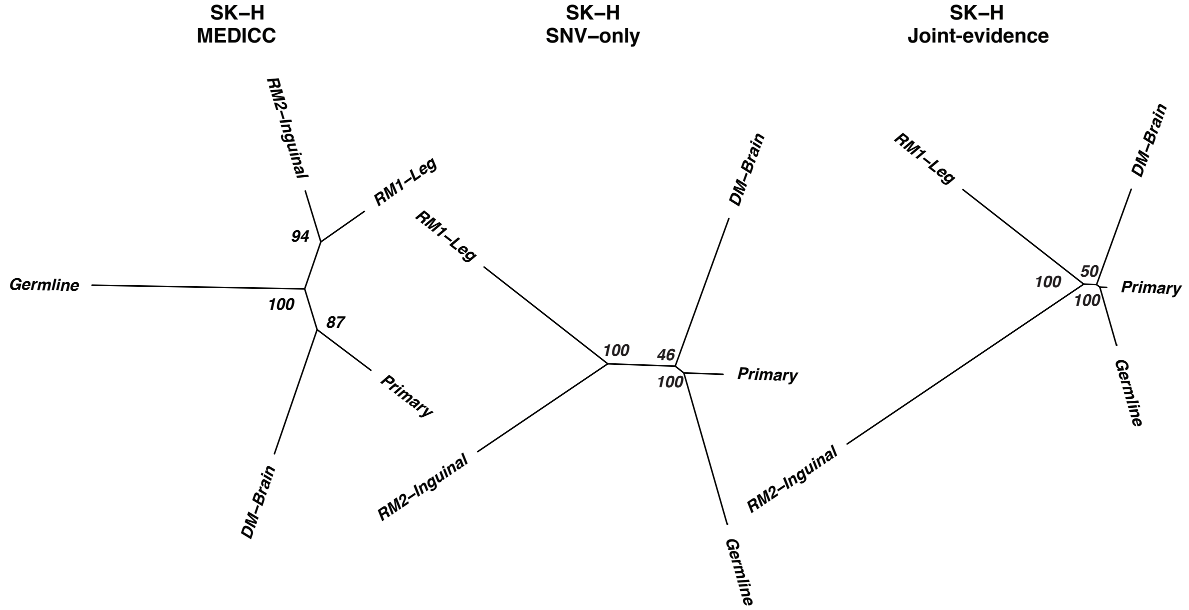

Supplementary Figure 20. MEDICC, SNV-only and joint-evidence phylogenies for patient SK-H. Germline branch length for the SNV-only tree was truncated to 100 for better representation.

FIGURE S21

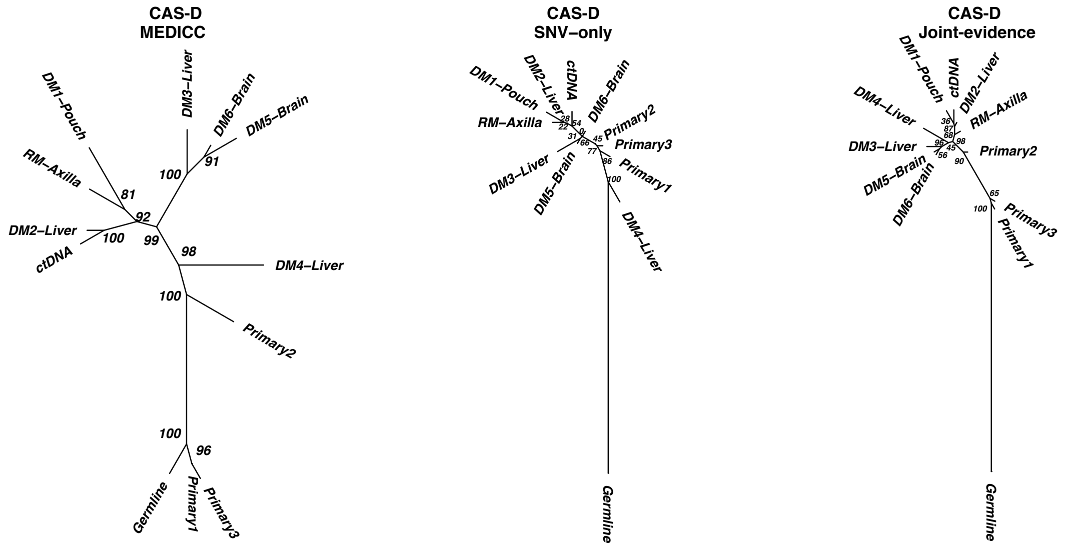

Supplementary Figure 21. MEDICC, SNV-only and joint-evidence phylogenies for patient CAS-D. Germline branch length for the SNV-only tree was truncated to 100 for better representation.

FIGURE S22

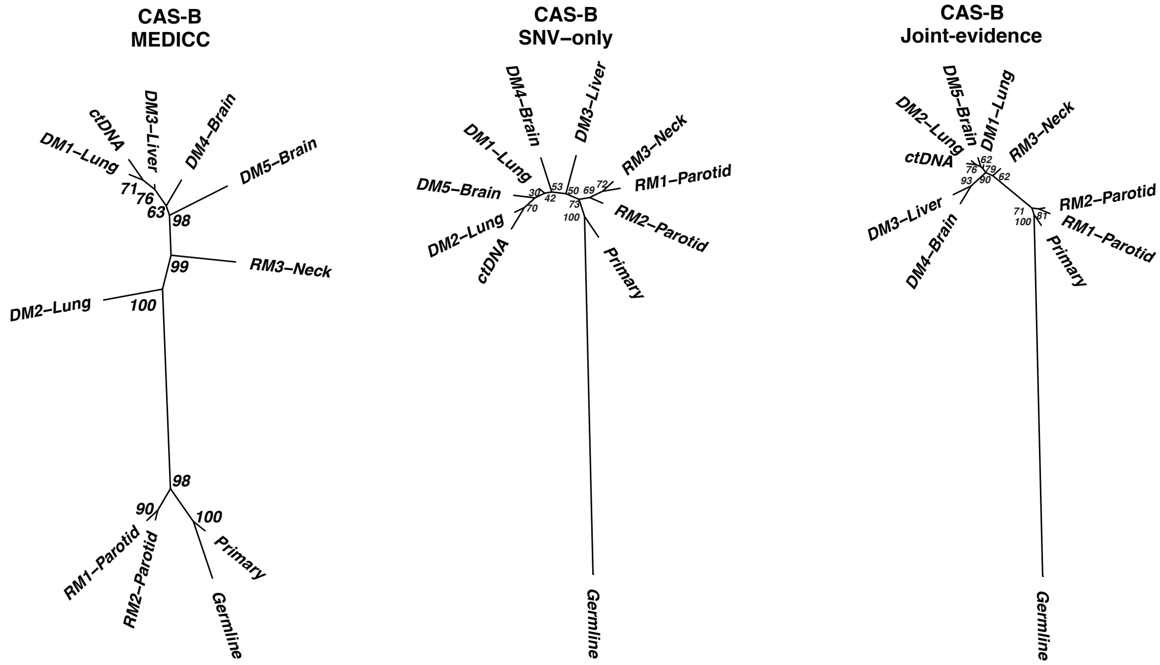

Supplementary Figure 22. MEDICC, SNV-only and joint-evidence phylogenies for patient CAS-B. Germline branch length for the SNV-only tree was truncated to 100 for better representation.

FIGURE S23

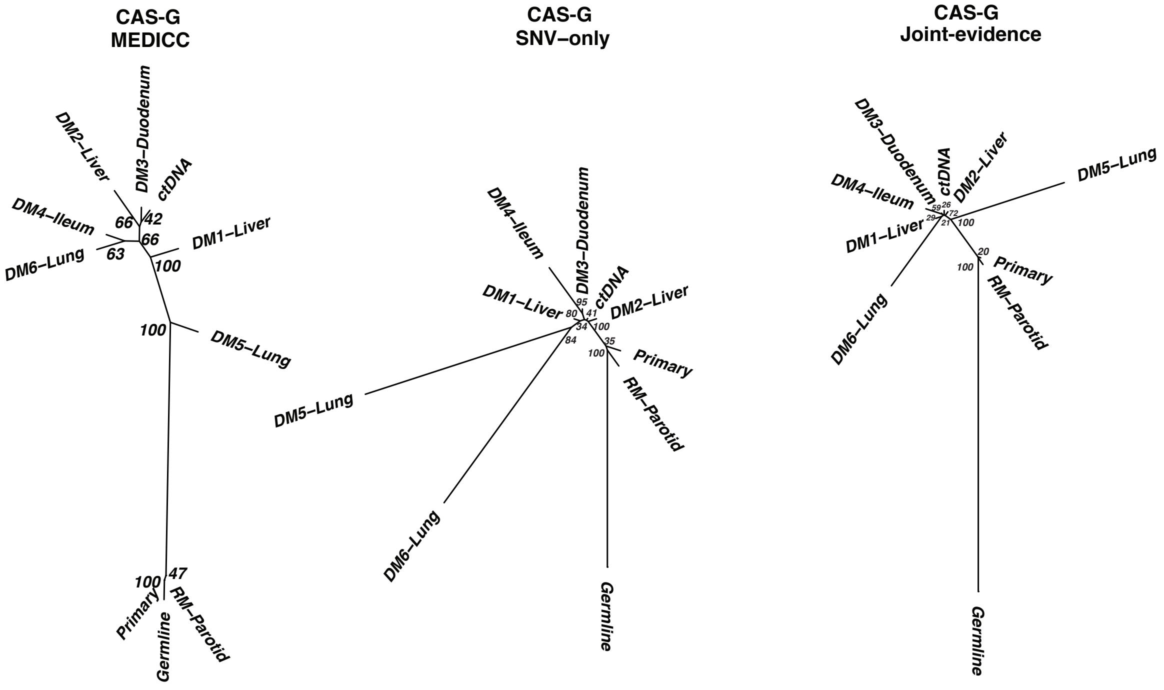

Supplementary Figure 23. MEDICC, SNV-only and joint-evidence phylogenies for patient CAS-G. Germline branch length for the SNV-only tree was truncated to 100 for better representation.

FIGURE S24

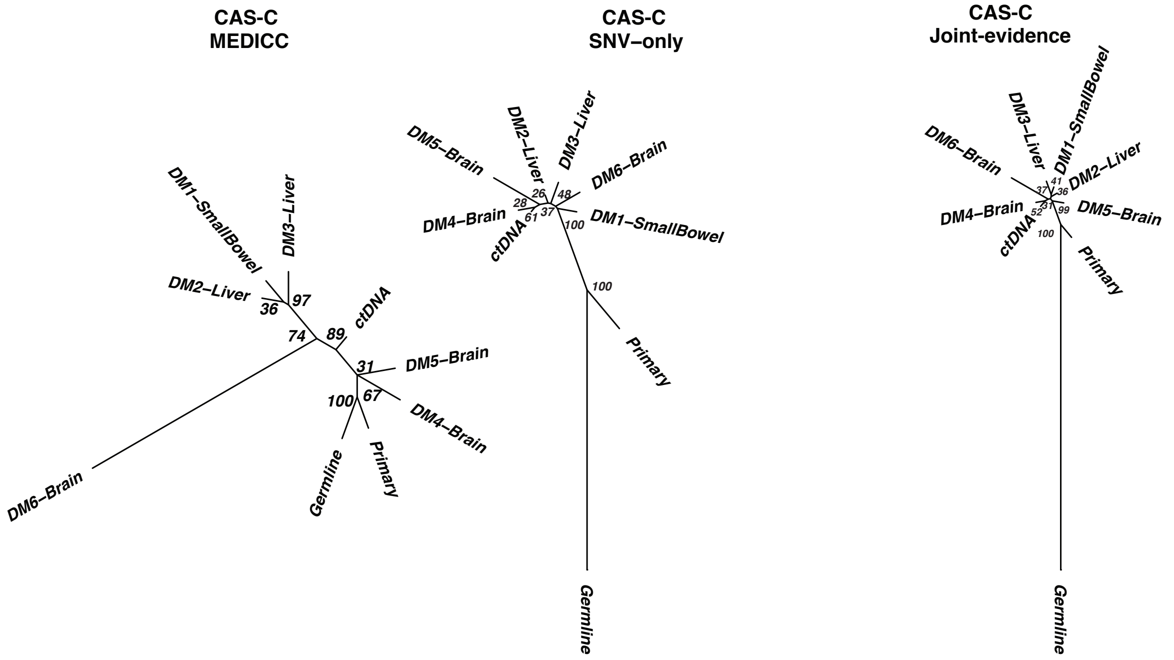

Supplementary Figure 24. MEDICC, SNV-only and joint-evidence phylogenies for patient CAS-C. Germline branch length for the SNV-only tree was truncated to 100 for better representation.

FIGURE S25

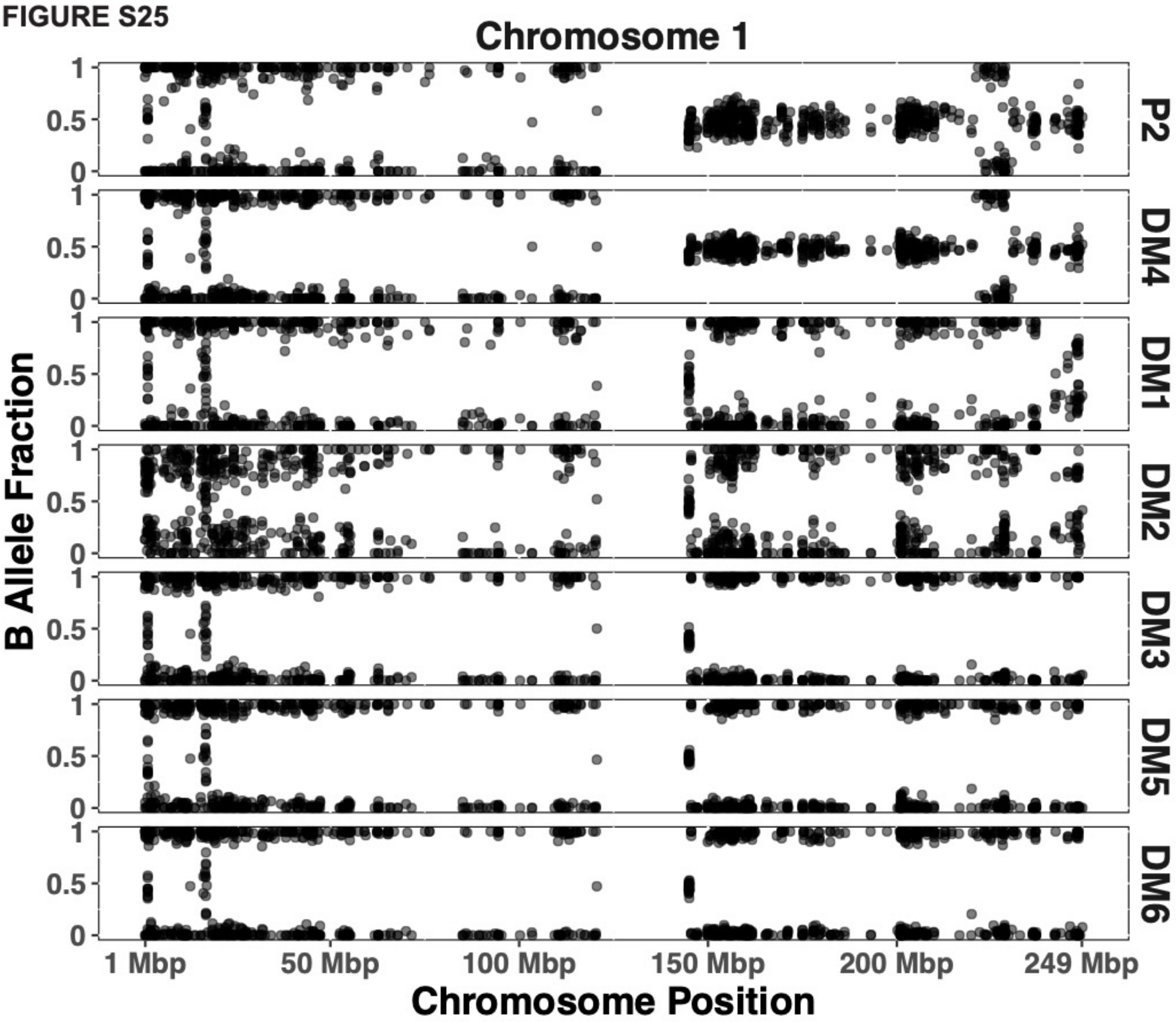

Supplementary Figure 25. B-allele frequencies of heterozygous germline SNPs in chromosome 1 for a primary core (P2) and distant metastases (DM) from patient CAS-D.

FIGURE S26

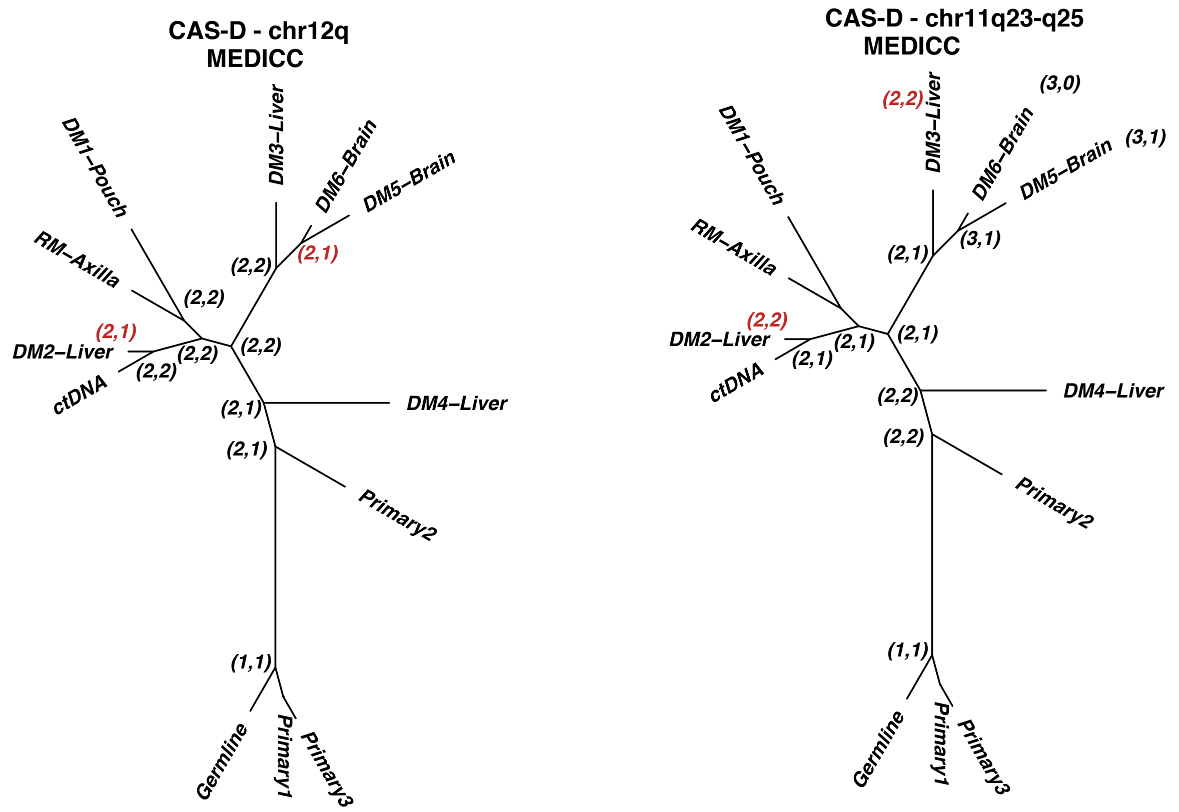

Supplementary Figure 26. Evidence of convergent evolution in CAS-D. Numbers in parenthesis indicate major and minor allele-specific copy numbers inferred by MEDICC at each node of the evolutionary tree. Convergent evolution of specific chromosomal regions of chromosomes 11 and 12 in CAS-D were obtained from least common ancestor reconstruction as reported by the MEDICC algorithm. Evolutionary steps for each region are highlighted on the same phylogenetic tree generated for this patient with genome-wide allele-specific copy number.

Supplementary Notes

Experimental validation of somatic single nucleotide variants (SNVs) and small insertions/deletions (InDels)

Validation of SNVs and small InDels detected with the pipeline used in this study was undertaken on a set of 59 high confidence and 19 low confidence mutations on 46 samples across 7 CASCADE patients (Supplementary Table 3, Supplementary Table 4). These mutations impact genes involved in DNA repair, cell cycle or are genes experimentally determined to be associated with chromosomal instability by previous studies. To determine accuracy, sensitivity and specificity, variants were experimentally validated using targeted amplicon sequencing under the following definitions: positives are high confidence calls that passed all filters for variant calling (see methods). Negatives were either (i) low confidence calls that were filtered out at some point in the variant calling, or (ii) absent calls on samples within a patient for which at least one other sample had a call on that particular variant. Using these definitions, targeted amplicon sequencing validation identified 301 true positives, 174 true negatives, 3 false positives and 51 false negatives. This resulted in an accuracy of 90%, a sensitivity of 86% and a specificity of 98%. This sensitivity was expected given the stringency of the variant calling, as demonstrated by several genes that had low confidence calls that were still validated by targeted amplicon sequencing (e.g. RAD18 in patient CAS-C and REST in patient CAS-E).

Comparison of SNV calling from DNA extracted from FFPE vs FF tumour material

To understand how formalin-fixed paraffin-embedding (FFPE) of tumours might have impacted variant calling, DNA was sequenced from adjacent fresh frozen (FF) and FFPE tumor tissue in a right lung melanoma metastasis from patient CAS-G. This sample was chosen due to the large number of mutations observed, including a high proportion of mutations characteristic of the effect of DNA degradation by FFPE[5]. Summary statistics (Supplementary Table 5) for the library preparation and sequencing showed similar mean coverage (157x FF vs 137x FFPE) and duplicate rates (FF: 5.24%, FFPE: 5.93%). As our SNV calling procedure included multiSNV[6], which simultaneously uses information from all per patient samples, the same pipeline for calling SNVs on CAS-G was executed separately for FF and FFPE samples (see methods). While 99.3% of ubiquitous FFPE mutations (those found across all tumour sites) and 73.9% of shared FFPE mutations (those found in at least two tumour sites and absent in at least one) were also found in FF material, 57.3% of private FFPE mutations were found in the FF counterpart. These corresponded to 601, 96 and 1,303 ubiquitous, shared and private mutations, respectively. Thus, most mutations in FFPE were also found in FF material and were private to this tumour site (Supplementary Figure 4).

The 987 private mutations found only in FFPE and the 569 private mutations found only in FF could be explained by (i) differences in filtration of variant calling pipeline, such as one variant caller supporting the variant (minimum required is two), or insufficient coverage, (ii) heterogeneity of the samples, and (iii) FFPE-induced artefacts. Of the 987 FFPE-only private mutations, 422 (42.7%) were either called by one variant caller or found in alignment in the FF data. Similarly, of the 569 FF-only private mutations, 307 (53.9%) were found in less stringent conditions in the FFPE counterpart.

Deconvolution of mutational signatures with deconstructSigs[3], using version 3 of the COSMIC set of signatures (http://cancer.sanger.ac.uk/cosmic/signatures) on the remainder of sample-specific private mutations, showed the majority of these associated with signature 11 (FF: 67.4%, FFPE: 68.3%), described as resembling effects of alkylating agents and likely resulting from defective DNA mismatch repair (Supplementary Figure 4). In contrast, a signature of spontaneous deamination by FFPE-based degradation was absent from the deconvolved set of FFPE-only private mutations. Thus, differences in private mutations based on FF and FFPE material were mostly explained by intratumoural heterogeneity as opposed to FFPE artefacts.

In agreement with this, the vast majority of private mutations in FF and FFPE material have a low probability of the mutation being clonal (Supplementary Figure 4).

Comparison of somatic copy number alterations (CNAs) between the same adjacent FFPE and FF samples showed 81.3% agreement for the total CNA calls, 88.2% for the major allele copy number and 91.2% for the minor allele copy number (Supplementary Figure 5). Regions with different copy number calls, such as those in chromosomes 4 and X, agreed with the type of event (i.e. amplifications were called in both chromosomes from both FFPE and FF samples). Differences appeared to be specific to FFPE tissue for this sample, as the patterns for FF tissue agreed with most distant metastases samples for this patient, making it a likely descendant from a common ancestor that carried particular CNAs.

Comparison of CNV-based, SNV-based and joint phylogenetic inference

Since the dominant mode of evolution of the advanced melanomas in our cohort was copy number change, and mutational losses due to LOH and subclonal are not incorporated into most phylogenetic methods of inference, our focus was initially on copy number-based estimation of phylogenies. However, despite the shortcomings of general phylogenetic approaches for cancer phylogenies [7], it is useful and interesting to consider evolutionary models based on other mutation types. Indeed, it may be that by taking advantage of more information from other mutation types, alternative phylogenies may be more accurate for some patients. Thus, we set out to infer phylogenies using SNVs only, as well as SNVs and CNAs jointly, using a balanced super-matrix approach.

To estimate trees jointly, we applied the following methodology on the four patients for which CNA-based phylogenies were originally reconstructed. For each patient, a binary supermatrix of SNVs, CNAs and whole genome doubling (GD) event across samples was constructed from the following information sources:

- All called SNVs with an estimated cancer cell fraction (CCF) of 0.5 or higher in at least one sample within the patient. The CCF threshold is applied to be consistent with the fact that the CNA-based phylogeny is constructed using clonal variants. Mutations within regions of shared LOH –*i.e.* regions where one or more, but not all samples carry LOH - were excluded.

- All CNAs that result in (1) LOH or (2) an estimated amplification or deletion with respect to the average ploidy of the sample, as reported by Murugaesu *et al* [8]. The purpose of this approach is to include regions of partial or total aneuploidy independently of the GD.
- The presence or absence of a GD event across samples.

This binary supermatrix captures (1) single point mutations due to varying mutational processes, (2) partial and total aneuploidy events such as deletions and amplifications specific to individual chromosomes, and (3) a genome doubling event.

Because the relative contribution of SNVs, CNAs and GD to the phylogeny reconstruction process is impacted by the number of variants (*e.g.* GD is represented by only one row in the supermatrix), we assigned different weights to SNVs, CNAs and the GD event in the supermatrix. To achieve this, the maximum count – *C* - between the number of informative (*i.e.* excluding autapomorphies) CNAs (*n_cnas*) and SNVs (*n_snvs*) was used to distribute weights as follows: (1) GD is assigned a weight equal to *C*, (2) each SNV is assigned a weight equal to *round(C/n_snvs)*, and (3) each CNA is assigned a weight equal to *round(C/ n_cnas)*. For the purpose of accurate branch length reconstruction, and independently of the aforementioned weight distribution, all autapomorphies were included with a weight of *1*. We refer to the phylogeny reconstruction based on this input supermatrix as the ***joint-evidence analysis***.

The scenario of a SNV-only phylogeny reconstruction, where SNVs represent 100% of the contribution, was pursued in a separate matrix with each SNV assigned a weight of 1. We refer to the phylogeny reconstruction based on this input matrix as the ***SNV-only analysis***.

The implicit enumeration algorithm (*ienum;* command, which guarantees an optimal solution) implemented in TNT 1.5[9] was used to generate the most parsimonious phylogenies using the equally-weighted supermatrix and SNV-only matrix.

For LOH events, a step matrix was created (*smatrix =0 1>0 i;* command) to force irreversibility of this type of events. All other variants were treated with the same cost for 0>1 and 1>0 transformation (default).

The topologies of the most parsimonious phylogenies were compared against the topology of the CNA-based tree (reported in Figure 6, hereinafter referred to as the *MEDICC analysis*) using the Robinson-Foulds (RF) distance (*tcomp <;* command), a measure of distance between two phylogenies based on the number of internal splits shared by the two phylogenies [10]. The RF distance varies between 0 (identical topologies) and 1 (no shared internal splits).

In the case of several parsimonious solutions for a given analysis, the one with the closest RF distance to the MEDICC tree was used for further analysis. If two or more solutions were closest to the MEDICC tree, the solution with lowest tree index as outputted by TNT was selected.

Support values for internal nodes within phylogenies were obtained via 1000 iterations of bootstrap resampling on the corresponding matrix (*resample boot frequency replic 1000;* command).

Comparison of MEDICC, SNV-based and joint-evidence phylogenies

Based on the above methodology, we have made the following observations between trees for each patient:

- CAS-D (Supplementary Figure 21)

  Clades or subtrees are generally in good agreement between the three methods with the exception of the position of DM4. In particular, the germline (G) & primaries (P) cluster in all trees. The RM consistently clusters with DM2 (liver), DM1 (Pouch of Douglas) and ctDNA, and it is positioned intermediate between P2 and these DMs in the joint evidence tree. Either the RM did not seed DM3, 5 & 6 or acquired substantial additional mutations after seeding. Position of DM4 is inconsistent between trees. DM4 remains heterozygous on 1q, while RM & all other DMs have an LOH event in that region. There are two possible evolutionary histories suggested by these trees: (i) either LOH occurs twice (joint evidence tree), or (ii) DM4 is seeded from the primary or an unsequenced regional met (MEDICC tree).

  The position of DM4, clustered with G, in the SNV-only tree is likely incorrect. Inspection of SNV coordinates for this patient shows eight of the eleven absent variants in lesion DM4

are located in chromosome 10 with coordinates in close proximity to the breakpoints of the intrachromosomal SVs (Figure 3D, Supplementary Figure 14). This suggests the presence/absence of these complex rearrangements play a role in the presence/absence pattern of these mutations on this patient, causing the effect observed in the SNV-only phylogeny.

- CAS-B (Supplementary Figure 22)
  P & RM samples are consistently closer to germline than DMs with the exception of DM2 in MEDICC tree. Joint evidence tree captures both GD & truncal UV mutations. The position of RM3 in SNV-only tree implies 2 independent genome doubling events. ctDNA consistently clusters in the clade with DM1 (lung).

- CAS-G (Supplementary Figure 23)
  The G, P & RM samples cluster in in all trees, but the order of branching is different in the MEDICC tree compared to the SNV-only and joint evidence trees. ctDNA consistently close to DM3 (duodenum). The SNV-based trees suggest the primary acquired additional mutations after the seeding of the regional met. Long branches in the SNV-only tree highlight the Signature 11 mutations.

- CAS-C (Supplementary Figure 24)
  G & P cluster consistently. There is no regional met in this patient. DM2 & 3 also cluster consistently in all trees with proximity to DM1. ctDNA appears close to DM4 (brain) across trees. Late whole genome doubling event in DM5 (brain) indicated by a long branch to this sample in the MEDICC tree and to a lesser extent on the joint evidence tree.

Comparison with Sanborn et al phylogenies

The phylogenies for patients MI-F and SK-H reported in Sanborn *et al* [2] were generated via inspection of groups of mutations partially shared among lesions and, based on this, estimation of the presence of subclones within lesions. For patient MI-F the phylogeny is deduced based on SNVs and reports a closer proximity of the LN cervical metastasis (RM2) to the skin distant metastasis (DM) compared to the Locoregional ear metastasis (RM1). The SNV-only tree generated with the methodology described above for this patient (Supplementary Figure 19) in this work also captures the closer proximity of RM2 to DM. For patient SK-H, the phylogeny

is by Sanborn *et al* is based on point mutations deduced to stem from different clones in the primary as well as a shared loss of heterozygosity event in chromosome 4 between the primary and the distant metastasis as well as; we find that the CNA+GD phylogeny captures the proximity of the primary tumour to the brain distant metastasis (Supplementary Figure 20), which includes the shared LOH event in chromosome 4 also called by this study (Figure 3A). These results support the validity of the trees published by Sanborn *et al*.

Supplementary Methods

Pre-autopsy tumor material

Pre-autopsy archival tissues obtained via routine diagnostic procedures were collected from pathology laboratories across Melbourne for each patient, in addition to on treatment tumor biopsies as part of the Melanoma Biomarkers Study (Peter MacCallum Cancer Centre Human Research Ethics Committee approval number: 11/105).

Autopsy procedure

Rapid autopsies were carried out at the Victorian Institute of Forensic Medicine (VIFM, Victoria, Australia) by qualified pathologists, mortuary technical staff and attending researchers to guide tumour sampling and photography. During autopsy, organs were removed from the body and reviewed by a pathologist for the presence of melanoma tissue Each organ was sectioned every 1-2cm to allow a thorough examination. Metastatic tissues were collected, annotated, photographed and checked macroscopically to minimize the collection of necrotic material. In the laboratory, fresh tumours were photographed and pieces from each placed in 10% NBF and liquid N2 (snap frozen).

Manual microdissection of patient tumour sections.

Patient formalin-fixed paraffin embedded (FFPE) tissues were sectioned using a microtome. Briefly, 20 sections per patient were cut. The 1st, 10th and 20th sections were cut at a thickness of 3μm for haematoxylin & eosin (H&E), Melan-A and S100 staining to enable localization of viable tumor by a qualified histopathologist. Only samples that contained >70% tumor were used for DNA extraction. The remaining 17 sections were cut at a thickness of 10μm and used for manual microdissection of regions of viable tumor using a fine scalpel under a light microscope (Leica M165FC, Germany) at 0.5x magnification. In cases where adjacent fresh frozen samples were available for DNA extraction (DNeasy blood and tissue kit, Qiagen) but macrodissection was not possible, tumor content was verified using digital PCR for *BRAF* mutations.

Genomic DNA Extraction

Genomic DNA from fresh frozen tissue and buffy coats (used as matching germline DNA) were extracted using the DNeasy blood and tissue kit (Qiagen) according to the manufacturer's protocol. Genomic DNA was extracted from FFPE tissue sections using the QIAamp DNA FFPE Tissue Kit (cat #56404). Briefly, 1ml Xylene was added to each sample and vortexed

vigorously for 10 secs followed by centrifugation for 2 mins at 12,000 RPM. The supernatant was aspirated without disturbing the pelleted material. A further 750µl of Xylene was added, vortexed vigorously for 10secs and centrifuged at 12,000 RMP for 2 mins. The supernatant was aspirated and 1ml of absolute ethanol was added to each sample, vortexed and centrifuged at 12,000 RPM for 2 mins. The supernatant was aspirated and the tube lids were left open at room temperature for 10 mins to ensure complete evaporation of ethanol. Each sample was re-suspended in 540ul Buffer ATL and 60ul proteinase K, vortexed and incubated at 56°C on a heat-block until the following day, with a top-up of 180µl Buffer ATL and 20µl Proteinase K at the end of Day 1. On Day 2, samples were incubated on a heat-block at 90°C for 1 hr and transferred into a clean 2ml micro-centrifuge tube, following brief centrifugation. 400ul Buffer AL was added to each sample and vortexed thoroughly to mix, followed by 400ul of absolute Ethanol (96-100%) and centrifuged at full speed (14,000rpm) for 30 secs. 600µl of each was transferred to a corresponding QIAamp MinElute column and centrifuged at 8000rpm for 1 min. The next 600µl of each lysate was transferred to the same corresponding QIAamp MinElute column and centrifuged at 8000rpm for 1 min. 500ul Buffer AW1 was added to each sample and centrifuged at 8000rpm for 1 min, followed by the addition of 500ul Buffer AW2 and centrifugation at 8000rpm for 1 min. Each sample was centrifuged at full speed for 3 min to dry the membrane completely and 50ul Buffer ATE was added to each sample to the centre of the membrane and incubated at room temperature for 10 min. Finally, each sample was centrifuged at full speed for 1 min to retain the flow-through. After DNA extraction, DNA samples were assessed for integrity [11].

For plasma DNA extraction, blood was collected in EDTA tubes and processed within two hours of collection. Whole blood was first centrifuged at 1600g for 10 minutes to separate plasma from peripheral blood cells, followed by a further centrifugation at 20,000g for 10 minutes to pellet remaining cells and/or debris. The plasma was then stored at -80°C until DNA extraction. DNA was extracted from 1-2ml aliquots of plasma using the QIAamp Circulating Nucleic Acid Kit (Qiagen, #55114). DNA was eluted into 50µl buffer AVE (Qiagen) and stored at -20°C.

DNA Quantitation

DNA was quantified using a Qubit fluorometer (Invitrogen) and a Quant-iT dsDNA HS kit (Invitrogen).

Whole Exome Sequencing

Total DNA was measured using Qubit dsDNA HS Assay (Thermo Fisher Scientific). Between 100 ng and 1 ug of input DNA was fragmented using a Covaris S2 focal acoustic device (Covaris) to an average fragment size range of 180-220 bp (duty cycle: 10%; intensity: 5; cycles per burst: 200; time: 3 minutes). Libraries were prepared using the KAPA Hyper Prep Kit for Illumina, (Kapa Biosystems) with SureSelect XT adaptors and primers (Agilent Technologies); 6 or 9 PCR cycles were used for good quality or FFPE-derived DNA, respectively. Hybridization capture was performed with SureSelect Human All Exon V5 or V6 (Agilent Technologies) according to manufacturer's protocols. Three indexed libraries were run per lane on an Illumina HiSeq2500 platform (paired-end 100 bp) according to standard protocols (Illumina).

Whole Genome Sequencing

100 ng of input DNA was fragmented as above to an average fragment size of 350 bp (duty cycle: 5%; intensity: 5; cycles per burst: 200; time: 50 seconds). Libraries were prepared using the TruSeq Nano DNA Library Preparation Kit (Illumina) using standard protocols. Libraries were sent to the Ramaciotti Centre for Genomics (Sydney, Australia) for QC, and sequencing was done at The Kinghorn Centre for Clinical Genomics (Sydney, Australia) on a HiSeq X Ten system (Illumina). One sample was sequenced per lane or over several lanes (paired-end 150bp) to a depth of 30X or 60X for germline or tumour, respectively.

Targeted Amplicon sequencing validation

Targeted amplicon (TA) sequencing was performed using the 48.48 Access Array™ system (Fluidigm), as described previously[12]. Missense variants predicted to be probably damaging by Polyphen2[13] or deleterious by SIFT[14], splice donor/acceptor variants, small indels as well as COSMIC variants occurring on the set of cell cycle, DNA repair and CIN-associated genes (see below) were used for validation. Primer sequences used are shown in Table S4. Samples were amplified with tagged target-specific primers in a microfluidic platform, which allowed 48 samples to be analyzed simultaneously with multiplexed assays in 2,448 reaction chambers. Following amplification, products were harvested, tagged with sample-specific barcodes, pooled together and purified using AMPure XP beads. All samples were analyzed in duplicate to control for PCR artefacts. The purified libraries were then sequenced with the MiSeq system (Illumina) using paired end sequencing with v2 150-bp kits.

Bcl2fastq was used to perform sample de-multiplexing and to convert BCL files generated from the MiSeq instrument into FASTQ files containing short-read data. Reads were assembled, aligned and variant called using two methods (i) a non-global alignment method developed in-house (known as *Primal*), which is based on a modified Smith-Waterman algorithm and (ii) *Canary*[15].

Somatic Single Nucleotide Variant and small Insertion/Deletion detection

Reads were quality-checked using FastQC v0.11.2 (http://www.bioinformatics.babraham.ac.uk/projects/fastqc/) and detected adapters removed using cutadapt[16] (v1.7.1) with a required minimum length (-m=35) and quality cutoff (-q=15). Reads were aligned against the hg19 version of the human genome using bwa[17] mem (v0.7.12). Aligned reads were further processed for marking of duplicates with picard v1.128 (http://picard.sourceforge.net/), and local indel realignment and base recalibration with the Genome Analysis Toolkit[18] (GATK v3.4.0). Reads marked as duplicates or with a mapping quality < 30 were excluded from further analysis.

Somatic Single Nucleotide Variants (SNVs) and small Insertions/Deletions (InDels) were detected for each tumour sample separately using the intersection of at least two variant callers of multiSNV[6] (v2.3), VarScan2[19] (v.2.3), muTect[20] (v3.1.0) and IndelGenotyper[18] (v1.04905). Variants (i) with total coverage below 10X in any of the tumour samples or the germline DNA, (ii) overlapping with annotated repetitive sequences by RepeatMasker[21], (iii) overlapping with low complexity sequences[22], (iv) present on the Exome Variant Server vESP6500 (http://evs.gs.washington.edu/EVS) or the 1000 genome database[23], and (v) overlapping with artifact-generating and other regions prone to false positives such as CDC27, mucins, ryanodine receptors and olfactory receptors[24,25] were filtered. Mutations/Mbp were computed based on the total number of base pairs targeted, using a haploid genome as reference as follows: (i) The number of total Mbps in the denominator was calculated by subtracting the non-overlapping sum of centromeric, telomeric, repetitive and artifactual regions from the total number of base pairs in the Whole Exome Sequencing (WES) platform as represented in the targets bed file. This was done since variants called within those regions are filtered out during the variant calling process. In the case of WES, this results in 47.5 (Agilent v5) to 64.9 (Agilent v4+UTR) Mbps targeted in a haploid genome. In the case of WGS, this results in 1,360 Mbps of reference haploid genome from an original 3,100 Mbps. These values present slight differences depending on the gender of the patient, which was also accounted for in the

calculation. (ii) The total number of mutations was divided by the estimate in (i). Since patients CAS-A and CAS-F were sequenced at the whole genome level, in order to make them comparable to those sequenced at the exome level in Figures 1B and 1C, mutations (Figure 1B) and mutational load (Figure 1C) were calculated at the whole exome level using Agilent v5 regions.

The fraction of mutated alleles in the tumour cells (MAFs) for each variant was estimated based on the procedure described by Anagnostou *et al*[26] as follows. Given r reads supporting a variant from a total of N, the likelihood of observing r reads is assumed to follow a binomial distribution with probability V_{exp} [26]. Based on this assumption, a likelihood was calculated over a grid of CCF values $c \in [0.01\text{-}1]$ and multiplicity $m \in (CN_{MIN}, CN_{MAJ})$ - if $CN_{MIN}>0$ - or $m=CN_{MAJ}$ otherwise, where CN_{MIN} and CN_{MAJ} are the minor and major allele copy number estimates for the genomic region that contains the variant. Likelihoods were normalized to add up to 1. The combination of multiplicity and CCF values with highest likelihood for the variant was used to estimate the MAF as $m \cdot c/CN_{TOT}$, where CN_{TOT} is the total copy number estimate for the genomic region that contains the variant. If two or more solutions of $m \cdot c$ maximized likelihood for a given variant, the solution with the highest CCF was used.

Germline SNPs used for assessment of allelic imbalances among samples of the same patient were obtained using the HaplotypeCaller function of GATK. Briefly, patterns of corrected BAF[27] of mutations in the tumour corresponding to heterozygote calls in the germline DNA ($0.4 \leq AF \leq 0.6$) were inspected across chromosomes between tumour samples to detect regions were opposite alleles had undergone an allelic imbalance event.

Mutational signatures, melanoma and cancer-associated genes, chromosomal instability and genomic integrity-associated genes

Mutational signatures were assessed using the deconstructSigs package (v1.9.0) with the cosmic signatures set V3[3]. To avoid spurious results due to low number of mutations in the assessment of mutational signatures involved in disease progression, mutations from all late-stage disease sites were pooled into one input dataset for each patient separately.

Significantly mutated genes in the melanoma TCGA cohort as calculated by the MutSig method (Q-value < 0.1) were retrieved from the cBioPortal for Cancer Genomics

(http://www.cbioportal.org/ - accessed February 2015). COSMIC cancer census genes[28] were retrieved from the COSMIC database v78 (http://cancer.sanger.ac.uk/census, accessed October 2016). Residues identified as statistically significant mutational hotspots (Q-value<0.01) were obtained as provided in the pan-cancer study by Chang *et al*[29].

Mutations with the potential to explain the observed patterns of aneuploidy and genome doubling events were detected by collating sets of genes into the following sets: (1) Genes associated with cell cycle and DNA repair pathways - as reported in reactome database [30] - were retrieved using the reactome.db R package[31]. (2) Genes previously associated with chromosomal instability (CIN) reported/reviewed by several studies[32-35] were collated to generate a list of CIN-associated genes. (3) Genes carrying mutations with increased dosage in tumour sites carrying the genome doubling event (GD group) compared to sites without the event (no-GD group) such as gained mutations, mutations associated with LOH events and mutations with increased multiplicity in patients CAS-G, CAS-D, CAS-B, CAS-C and ETH-E. The average estimated fraction of mutated alleles in the tumour cells (MAF) was set to 16% or greater in the GD group to represent expected changes in MAF due to increased multiplicity, with no less than 10% difference between the maximum MAF of the no-GD group and the minimum MAF of the GD group. GO slim terms associated to these genes were annotated using the biomart data mining tool available at http://grch37.ensembl.org/.

Neoantigen prediction and predicted expression in TCGA cohort

For each patient, germline DNA was used to predict the 4-digit types of HLA-A, HLA-B and HLA-C alleles with OptiType v1.0[36]. Experimental molecular HLA typing of the germline DNA done at the Australian Red Cross Blood Service for CAS-D validates OptiType 4-digit prediction for the three alleles. The impact of variants on protein-coding ENSEMBL GRCh37.75 transcripts was assessed simultaneously to ensure proper evaluation of adjacent mutations on same residues[37]. Mutated 17-mers centered around each amino acid change for each transcript and the predicted HLA class I alleles were provided as input to NetMHC v4.0[38,39] with default settings.

CooVar-v0.07[37] was used to simultaneously evaluate the impact of SNVs on protein-coding ENSEMBL GRCh37.75 transcripts for each tumour lesion in each patient; this ensures proper evaluation of adjacent mutations on same residues. The 'categorized-gvs.gvf' and 'variant_peptides.fasta' files generated by CooVar for each tumour lesion were used to extract

the mutated 17-mers centered around each amino acid change for each transcript in fasta format. This was done using a script generate-epitope-sequence.pl (available at https://github.com/PapenfussLab/CascadeNeoPipe).

The extracted 17-mers and the predicted HLA class I alleles obtained with OptiType 1.0 were provided as input to netMHC v4.0 with default settings and a peptide length (-l parameter) of 9 for MHC class I affinity prediction as trained by this tool from quantitative peptide-MHC class I affinity measurements from the IEDB (PMID: 26515819). For each patient, a matrix of mutations with its coordinates versus samples and associated expression of the gene as obtained from the TCGA cohort was generated where each mutation in each sample was labeled as strong binder (2), weak binder (1), no binder (0) or not mutated (-1).

Overlap of mutation coordinates (including neoantigenic mutations) with other chromosomal regions including regions of LOH, amplification and deletion was obtained with the *findOverlaps* function of the GenomicRanges R package.

Expression of predicted neoantigens was estimated using the median of log2-transformed normalised count data from the TCGA SKCM cohort generated using RSEM[40] as provided in the legacy archive of GDC (https://portal.gdc.cancer.gov/legacy-archive/ (previously https://tcga-data.nci.nih.gov/tcgafiles/ftp_auth/distro_ftpusers/anonymous/tumor/skcm/cgcc/unc.edu/illuminahiseq_rnaseqv2/rnaseqv2/unc.edu_SKCM.IlluminaHiSeq_RNASeqV2.Level_3.1.14.0/, accessed September 2015) by selecting only those with suffix .rsem.genes.normalized_results. Neoantigens in genes with median log2 normalized counts greater than or equal to 5 were considered to be predicted expressed neoantigens. This threshold was selected empirically by inspection of the distribution of log2 normalised expected counts.

Characterisation of variants deleted due to loss of heterozygosity

For each LOH event acquired in late-stage disease, four quantities were recorded: (i) number of synonymous mutations, (ii) number of non-synonymous mutations, (iii) number of neoantigenic mutations, and (iv) number of non-neoantigenic mutations lost in each late-stage lesion. Neoantigenic mutations were those resulting in neoepitopes of predicted weak or strong binding affinity to any of the HLA class I alleles of the patient. Non-neoantigenic mutations

were synonymous mutations, as well as non-synonymous mutations not predicted to have binding affinity to the HLA alleles of the patient.

Over-segmentation of LOH regions could generate short artifactual events, which may not include the variants that are under selection, particularly in WES data. To explore this, we compared the LOH segment lengths from WGS of five fresh samples from patient CAS-D and WES of matched FFPE samples from the same tumours (Supplementary Figure 10C). This shows that for segments longer than 10Mb, there was good agreement between WES and WGS LOH calls, but there was substantial disagreement for smaller segments, supporting the idea of over-segmentation.

Based on published codon usage bias frequencies (https://hive.biochemistry.gwu.edu), the expected ratio of the number of non-synonymous mutations to the number of synonymous (NS:S) mutations was estimated to be 3.39. Using this threshold, two groups of large acquired LOH segments (>10Mb) were defined: non-synonymous above expected (NS>3.39S) and non-synonymous less than expected (NS<3.39S).

Across all patients, there were 194 large acquired LOH segments. Of these, 67 segments deleted more non-synonymous mutations than expected; 80 deleted fewer non-synonymous mutations than expected; and 47 deleted zero mutations. Under the null model of uniform distribution and no selection, we expect equal probability of being above or below the threshold, however, need to deal with LOH regions with no mutations deleted. We modelled this using the Binomial distribution, treating regions that deleted no mutations or fewer non-synonymous mutations than expected with probability $p_{failure} = (1+p_{zero})/2$, where p_{zero} is the probability that an LOH event does not delete any mutations and was estimated from the data; regions that deleted more non-synonymous mutations than the expected was treated as a success with $p_{success} = 1-p_{failure}$.

Additionally, we classified each segment as high neoantigenic load if the proportion for that acquired LOH segment is greater than the observed proportion across the entire tumour genome, including no LOH and other AI regions, and low neoantigenic load otherwise. Based on the NS:S>3.39, NS:S<3.39, high neoantigenic load and low neoantigenic load groups, we created a 2x2 table for each patient and tested for mutual exclusivity using one-sided Barnard's exact tests with the Exact R package.

Somatic copy number alterations detection and CNV-based phylogeny

Estimates of allele-specific copy number, tumour purity and ploidy within each sample were obtained using Sequenza[41] with a median normalization method and with appropriate gender setting per patient. In order to avoid spurious segmentation of small regions, the gamma and kmin parameters for the aspcf function were set to 500 and 100 resp. for the WGS samples. Manual inspection of Sequenza results prompted the utilization of the second optimal ploidy and purity solution for copy number calling in samples CAS-G-76, CAS-D-ctDNA, CAS-D-62, ETH-F-P4 and CAS-B-15B08317-3A. For samples ETH-E-E1 and CAS-C-44, Sequenza's two optimal solutions resulted in spurious copy number profiles; for these two samples, facets[42] with default settings was used instead, with bam files pre-processed using the snp-pileup command with –q 30, -Q 30 and –r 10,10. MEDICC[43] was used for CNV-based phylogeny, using allele-specific copy number calls summarized at the cytogenetic band level. Support values for each node were obtained by resampling of the distance matrix for each patient using the *medicc.resample.distance.matrices* function from the MEDICCquant R package. These matrices were used as input for the *fitch* function of the phylip-3.695 package[44] using the Fitch-Margoliash method to generate trees followed by the *consense* function using the majority rule (extended) method to generate consensus trees. Visualization of phylogenies were generated using the *plot.phylo* command of the ape R package[45] providing as input the newick representation of each tree.

Structural variant detection

Large (>1000 bps) somatic SVs were detected based on whole-genome sequencing using the Genomic Rearrangement Identification Software Suite (GRIDSS) excluding blacklisted regions identified by ENCODE[46]. Briefly, GRIDSS performs genome-wide break-end assembly and combines assembly, read pair, and split read evidence in a probabilistic structural variant scoring model. In order to avoid spurious calls within breakpoints due to micro-homology, the ability of GRIDSS to process multiple samples simultaneously was used, which resolves breakpoints jointly for a given SV across samples. SVs were kept if they were absent in the germline DNA sample and if the quality score was in the top 10 percentile for at least one of the samples.

Fluorescence In-Situ Hybridisation

Fluorescence *in-situ* hybridisation (FISH) was performed for several cases to validate copy number results. Several probes were chosen for their relevance to the required validation, namely *Myc*/CEP8 and *CCND1*/CEP11 probes for gene amplification and *CDKN2A*/CEP9 probes to identify gene loss. Four micron FFPE tissue sections were incubated at 60^0C for 1 hour to melt the paraffin, then further deparaffinised through several xylene baths, rehydrated through graded alcohols and washed in water. Target retrieval was performed in Heat Pretreatment Solution (Invitrogen) in a pressure cooker at 125^0C for 2½ min followed by washing in water. Sections were then treated with Enzyme Pretreatment Reagent (Invitrogen) for 15 min at 37^0C, washed in water, dehydrated through 2 min washes in 70%, 80%, 90% and 100% alcohol solutions and air dried. Probe mix was added to a small area of the tissue section and the slide was coverslipped and sealed with rubber cement to prevent evaporation during hybridisation. Slides were placed on a StatSpin Hybridiser (Dako) for denaturation for 5 min at 80^0C followed by hybridisation for 18 hours at 37^0C. After hybridisation coverslips were removed and slides washed in two solutions of a 0.5x SSC stringent wash buffer; for 2 min at room temperature followed by 5 min at 75^0C. Slides were then washed in water, mounted in Vectashield mounting medium with Dapi (Vector), coverslipped and stored at 4^0C in the dark prior to scoring.

FISH slides were scored on an Olympus BX51 fluorescence microscope. For each slide the area to be scored was identified at low (10x) magnification using the DAPI filter. A minimum of 100 cells, typically 30-40 from at least three fields, were scored for the number of gene (red) and chromosome (green) signals at high (100x) magnification. Raw data were entered into a Microsoft Excel spreadsheet for calculation of gene and chromosome copy number status.

Supplementary References

1 Raaijmakers, M. I. *et al.* Co-existence of BRAF and NRAS driver mutations in the same melanoma cells results in heterogeneity of targeted therapy resistance. *Oncotarget* **7**, 77163-77174, doi:10.18632/oncotarget.12848 (2016).

2 Sanborn, J. Z. *et al.* Phylogenetic analyses of melanoma reveal complex patterns of metastatic dissemination. *Proc Natl Acad Sci U S A* **112**, 10995-11000, doi:10.1073/pnas.1508074112 (2015).

3 Rosenthal, R., McGranahan, N., Herrero, J., Taylor, B. S. & Swanton, C. DeconstructSigs: delineating mutational processes in single tumors distinguishes DNA repair deficiencies and patterns of carcinoma evolution. *Genome Biol* **17**, 31, doi:10.1186/s13059-016-0893-4 (2016).

4 Landau, D. A. *et al.* Evolution and impact of subclonal mutations in chronic lymphocytic leukemia. *Cell* **152**, 714-726, doi:10.1016/j.cell.2013.01.019 (2013).

5 Do, H. & Dobrovic, A. Dramatic reduction of sequence artefacts from DNA isolated from formalin-fixed cancer biopsies by treatment with uracil-DNA glycosylase. *Oncotarget* **3**, 546-558 (2012).

6 Josephidou, M., Lynch, A. G. & Tavare, S. multiSNV: a probabilistic approach for improving detection of somatic point mutations from multiple related tumour samples. *Nucleic Acids Res* **43**, e61, doi:10.1093/nar/gkv135 (2015).

7 Beerenwinkel, N., Schwarz, R. F., Gerstung, M. & Markowetz, F. Cancer evolution: mathematical models and computational inference. *Syst Biol* **64**, e1-25, doi:10.1093/sysbio/syu081 (2015).

8 Murugaesu, N. *et al.* Tracking the genomic evolution of esophageal adenocarcinoma through neoadjuvant chemotherapy. *Cancer Discov* **5**, 821-831, doi:10.1158/2159-8290.CD-15-0412 (2015).

9 Goloboff, P. A., Farris, J. S. & Nixon, K. C. TNT, a free program for phylogenetic analysis. *Cladistics* **24**, 774-786, doi:doi:10.1111/j.1096-0031.2008.00217.x (2008).

10 Robinson, D. F. & Foulds, L. R. Comparison of phylogenetic trees. *Mathematical Biosciences* **53**, 131-147, doi:https://doi.org/10.1016/0025-5564(81)90043-2 (1981).

11 Wong, S. Q. *et al.* Targeted-capture massively-parallel sequencing enables robust detection of clinically informative mutations from formalin-fixed tumours. *Sci Rep* **3**, 3494, doi:10.1038/srep03494 (2013).

12 Wong, S. Q. *et al.* Circulating Tumor DNA Analysis and Functional Imaging Provide Complementary Approaches for Comprehensive Disease Monitoring in Metastatic Melanoma. *JCO Precision Oncology*, 1-14, doi:10.1200/po.16.00009 (2017).

13 Adzhubei, I. A. *et al.* A method and server for predicting damaging missense mutations. *Nat Methods* **7**, 248-249, doi:10.1038/nmeth0410-248 (2010).

14 Ng, P. C. & Henikoff, S. Predicting deleterious amino acid substitutions. *Genome Res* **11**, 863-874, doi:10.1101/gr.176601 (2001).

15 Doig, K. D. *et al.* Canary: an atomic pipeline for clinical amplicon assays. *BMC Bioinformatics* **18**, 555, doi:10.1186/s12859-017-1950-z (2017).

16 Martin, M. Cutadapt removes adapter sequences from high-throughput sequencing reads. *EMBnet. J.* **17**, 10-12 (2011).

17 Li, H. & Durbin, R. Fast and accurate short read alignment with Burrows-Wheeler transform. *Bioinformatics* **25**, 1754-1760, doi:10.1093/bioinformatics/btp324 (2009).

18 McKenna, A. *et al.* The Genome Analysis Toolkit: a MapReduce framework for analyzing next-generation DNA sequencing data. *Genome Res* **20**, 1297-1303, doi:10.1101/gr.107524.110 (2010).
19 Koboldt, D. C. *et al.* VarScan 2: somatic mutation and copy number alteration discovery in cancer by exome sequencing. *Genome Res* **22**, 568-576, doi:10.1101/gr.129684.111 (2012).
20 Cibulskis, K. *et al.* Sensitive detection of somatic point mutations in impure and heterogeneous cancer samples. *Nat Biotechnol* **31**, 213-219, doi:10.1038/nbt.2514 (2013).
21 Smit, A. F. A., Hubley, R. & Green, P. *RepeatMasker Open-4.0*, <http://www.repeatmasker.org> (2013-2015).
22 Li, H. Toward better understanding of artifacts in variant calling from high-coverage samples. *Bioinformatics* **30**, 2843-2851, doi:10.1093/bioinformatics/btu356 (2014).
23 Genomes Project, C. *et al.* An integrated map of genetic variation from 1,092 human genomes. *Nature* **491**, 56-65, doi:10.1038/nature11632 (2012).
24 Li, M. X. *et al.* Predicting mendelian disease-causing non-synonymous single nucleotide variants in exome sequencing studies. *PLoS Genet* **9**, e1003143, doi:10.1371/journal.pgen.1003143 (2013).
25 Lawrence, M. S. *et al.* Mutational heterogeneity in cancer and the search for new cancer-associated genes. *Nature* **499**, 214-218, doi:10.1038/nature12213 (2013).
26 Anagnostou, V. *et al.* Evolution of Neoantigen Landscape During Immune Checkpoint Blockade in Non-Small Cell Lung Cancer. *Cancer Discovery*, doi:10.1158/2159-8290.cd-16-0828 (2017).
27 Li, Y. & Xie, X. Deconvolving tumor purity and ploidy by integrating copy number alterations and loss of heterozygosity. *Bioinformatics* **30**, 2121-2129, doi:10.1093/bioinformatics/btu174 (2014).
28 Futreal, P. A. *et al.* A census of human cancer genes. *Nat Rev Cancer* **4**, 177-183, doi:10.1038/nrc1299 (2004).
29 Chang, M. T. *et al.* Identifying recurrent mutations in cancer reveals widespread lineage diversity and mutational specificity. *Nat Biotechnol* **34**, 155-163, doi:10.1038/nbt.3391 (2016).
30 Joshi-Tope, G. *et al.* Reactome: a knowledgebase of biological pathways. *Nucleic Acids Res* **33**, D428-432, doi:10.1093/nar/gki072 (2005).
31 Ligtenberg, W. reactome.db: A set of annotation maps for reactome. (2017).
32 Maiato, H. & Logarinho, E. Mitotic spindle multipolarity without centrosome amplification. *Nat Cell Biol* **16**, 386-394 (2014).
33 Thompson, S. L., Bakhoum, S. F. & Compton, D. A. Mechanisms of chromosomal instability. *Curr Biol* **20**, R285-295, doi:10.1016/j.cub.2010.01.034 (2010).
34 Nigg, E. A. Centrosome aberrations: cause or consequence of cancer progression? *Nat Rev Cancer* **2**, 815-825, doi:10.1038/nrc924 (2002).
35 Valente, D. *et al.* HIPK2 deficiency causes chromosomal instability by cytokinesis failure and increases tumorigenicity. *Oncotarget* **6**, 10320-10334, doi:10.18632/oncotarget.3583 (2015).
36 Szolek, A. *et al.* OptiType: precision HLA typing from next-generation sequencing data. *Bioinformatics* **30**, 3310-3316, doi:10.1093/bioinformatics/btu548 (2014).
37 Vergara, I. A., Frech, C. & Chen, N. CooVar: co-occurring variant analyzer. *BMC Res Notes* **5**, 615, doi:10.1186/1756-0500-5-615 (2012).

38 Nielsen, M. *et al.* Reliable prediction of T-cell epitopes using neural networks with novel sequence representations. *Protein Sci* **12**, 1007-1017, doi:10.1110/ps.0239403 (2003).
39 Andreatta, M. & Nielsen, M. Gapped sequence alignment using artificial neural networks: application to the MHC class I system. *Bioinformatics* **32**, 511-517, doi:10.1093/bioinformatics/btv639 (2016).
40 Li, B. & Dewey, C. N. RSEM: accurate transcript quantification from RNA-Seq data with or without a reference genome. *BMC Bioinformatics* **12**, 323, doi:10.1186/1471-2105-12-323 (2011).
41 Favero, F. *et al.* Sequenza: allele-specific copy number and mutation profiles from tumor sequencing data. *Ann Oncol* **26**, 64-70, doi:10.1093/annonc/mdu479 (2015).
42 Shen, R. & Seshan, V. E. FACETS: allele-specific copy number and clonal heterogeneity analysis tool for high-throughput DNA sequencing. *Nucleic Acids Res* **44**, e131, doi:10.1093/nar/gkw520 (2016).
43 Schwarz, R. F. *et al.* Phylogenetic quantification of intra-tumour heterogeneity. *PLoS Comput Biol* **10**, e1003535, doi:10.1371/journal.pcbi.1003535 (2014).
44 Felsenstein, J. *PHYLIP (Phylogeny Inference Package) version 3.6.*, 2005).
45 Paradis, E. *et al.* Package ‘ape’: Analysis of Phylogenetics and Evolution. 1-283 (2017).
46 Cameron, D. L. *et al.* GRIDSS: sensitive and specific genomic rearrangement detection using positional de Bruijn graph assembly. *Genome Res*, doi:10.1101/gr.222109.117 (2017).

Supplementary Tables

Supplementary Table 1. Sample information for the 7 CASCADE patients.

Case	Sample	Material	Type	Figure ID	Kit	Mean Coverage	Purity Estimate	Organ
CAS-G	CAS-G-71	FFPE	DM	DM3	Agilent SureSelect Human All Exome V5	150.07	0.78	small bowel duodenum
CAS-G	CAS-G-72	FFPE	DM	DM4	Agilent SureSelect Human All Exome V5	137.61	0.79	small bowel ileum
CAS-G	CAS-G-74	FFPE	DM	DM1	Agilent SureSelect Human All Exome V5	147.41	0.8	liver left lobe
CAS-G	CAS-G-75	FFPE	DM	DM2	Agilent SureSelect Human All Exome V5	132.22	0.77	liver right lobe
CAS-G	CAS-G-76	FFPE	DM	DM5	Agilent SureSelect Human All Exome V5	141.89	0.75	left lung lower lobe
CAS-G	CAS-G-77	FFPE	DM	NA	Agilent SureSelect Human All Exome V5	137.43	0.69	right lung lower lobe
CAS-G	CAS-G-77FF	FF	DM	DM6	Agilent SureSelect Human All Exome V5	156.93	0.87	right lung lower lobe
CAS-G	CAS-G-91	FFPE	P	P	Agilent SureSelect Human All Exome V5	160.14	0.76	left ear
CAS-G	CAS-G-94	FFPE	RM	RM	Agilent SureSelect Human All Exome V5	132.59	0.78	parotid gland
CAS-G	CAS-G-ctDNA	Plasma	ctDNA	ctDNA	Agilent SureSelect Human All Exome V5	236.37	0.76	NA
CAS-G	CAS-G-GL	Buffy Coat	G	NA	Agilent SureSelect Human All Exome V5	159.12	NA	NA
CAS-D	CAS-D-ctDNA	Plasma	ctDNA	ctDNA	Agilent SureSelect Human All Exome V5	161.43	0.39	NA
CAS-D	CAS-D-60	FFPE	DM	DM1	Agilent SureSelect Human All Exome V5	145.6	0.53	pouch of douglas
CAS-D	CAS-D-61-1	FFPE	P	P1	Agilent SureSelect Human All Exome V5	138.96	0.36	excision bx of lesion on back core #1
CAS-D	CAS-D-61-2	FFPE	P	P2	Agilent SureSelect Human All Exome V5	143.87	0.52	excision bx of lesion on back core #2
CAS-D	CAS-D-61-3	FFPE	P	P3	Agilent SureSelect Human All Exome V5	130.12	0.34	excision bx of lesion on back core #3
CAS-D	CAS-D-62	FFPE	RM	RM	Agilent SureSelect Human All Exome V5	182.72	0.63	right axillary dissection
CAS-D	CAS-D-64	FFPE	DM	DM2	Agilent SureSelect Human All Exome V5	148.18	0.4	liver Right lobe
CAS-D	CAS-D-65	FFPE	DM	DM3	Agilent SureSelect Human All Exome V5	107.89	0.77	liver central
CAS-D	CAS-D-66	FFPE	DM	DM4	Agilent SureSelect Human All Exome V5	135.24	0.7	left Liver
CAS-D	CAS-D-68	FFPE	DM	DM5	Agilent SureSelect Human All Exome V5	136	0.73	brain - right hippocampus
CAS-D	CAS-D-70	FFPE	DM	DM6	Agilent SureSelect Human All Exome V5	126.94	0.7	brain - left parietal
CAS-D	CAS-D-89	Buffy Coat	G	NA	Agilent SureSelect Human All Exome V5	122.37	NA	NA
CAS-E	CAS-E-1	FFPE	DM	DM1	Agilent SureSelect Human All Exome V5	200.17	0.8	peri appendix/outer app
CAS-E	CAS-E-10	FFPE	RM	RM	Agilent SureSelect Human All Exome V5	188.73	0.33	sentinel lymph node
CAS-E	CAS-E-3	FFPE	DM	DM2	Agilent SureSelect Human All Exome V5	174.85	0.53	neck r lump/medistineal skin
CAS-E	CAS-E-4	FFPE	DM	DM3	Agilent SureSelect Human All Exome V5	192.8	0.74	lung r lower lobe
CAS-E	CAS-E-5	FFPE	DM	DM4	Agilent SureSelect Human All Exome V5	170.24	0.27	mediastinal mass
CAS-E	CAS-E-6	FFPE	DM	DM5	Agilent SureSelect Human All Exome V5	204.3	0.61	upper mediastinal mass/skin
CAS-E	CAS-E-8	FFPE	P	P	Agilent SureSelect Human All Exome V5	172.81	0.29	tumour neck
CAS-E	CAS-E-83	Buffy Coat	G	NA	Agilent SureSelect Human All Exome V5	152.72	NA	NA
CAS-C	CAS-C-37	FFPE	DM	DM1	Agilent SureSelect Human All Exome V5	110.4	0.84	small bowel
CAS-C	CAS-C-40	FF	DM	DM2	Agilent SureSelect Human All Exome V5	133.73	0.81	liver left lobe
CAS-C	CAS-C-41	FFPE	DM	DM3	Agilent SureSelect Human All Exome V5	126.88	0.63	liver right lobe
CAS-C	CAS-C-42	FF	DM	DM4	Agilent SureSelect Human All Exome V5	154.38	0.67	brain - l temporal lobe
CAS-C	CAS-C-43	FF	DM	DM6	Agilent SureSelect Human All Exome V5	158.68	0.63	brain - r parietal lobe (outer s)
CAS-C	CAS-C-44	FFPE	DM	DM5	Agilent SureSelect Human All Exome V5	131.08	0.24	brain - r lobe of cerebellum

CAS-C	CAS-C-46	FFPE	P	P	Agilent SureSelect Human All Exome V5	162.45	0.7	excision of r back
CAS-C	CAS-C-ctDNA	Plasma	ctDNA	ctDNA	Agilent SureSelect Human All Exome V5	159.51	0.64	NA
CAS-C	CAS-C-86	Buffy Coat	G	NA	Agilent SureSelect Human All Exome V5	204.77	NA	NA
CAS-B	CAS-B-14B37035-1B	FFPE	RM	RM1	Agilent SureSelect Human All Exome V6	255.64	0.74	L) superficial parotid gland plus nodes
CAS-B	CAS-B-14B37035-1C	FFPE	RM	RM2	Agilent SureSelect Human All Exome V6	125	0.66	L) superficial parotid gland plus nodes
CAS-B	CAS-B-15B08317-3A	FFPE	RM	RM3	Agilent SureSelect Human All Exome V6	207.97	0.39	Neck node Bx, malignant melanoma replacing a LN
CAS-B	CAS-B-21	FFPE	DM	DM2	Agilent SureSelect Human All Exome V6	226.47	0.3	left lung
CAS-B	CAS-B-22	FF	DM	DM3	Agilent SureSelect Human All Exome V6	145.16	0.57	R lung
CAS-B	CAS-B-24	FFPE	DM	DM1	Agilent SureSelect Human All Exome V6	197.99	0.36	R lobe liver
CAS-B	CAS-B-37	FFPE	DM	DM4	Agilent SureSelect Human All Exome V6	140.65	0.23	brain-left frontal
CAS-B	CAS-B-42	FFPE	DM	DM5	Agilent SureSelect Human All Exome V6	147.38	0.34	brain-right caudate
CAS-B	CAS-B-Primary-1	FFPE	P	P	Agilent SureSelect Human All Exome V6	131.07	0.52	L) frontal scalp excision - core 1
CAS-B	CAS-B-ctDNA	Plasma	ctDNA	ctDNA	Agilent SureSelect Human All Exome V6	289.75	0.32	NA
CAS-B	CAS-B-Normal	Buffy Coat	G	NA	Agilent SureSelect Human All Exome V6	197.95	NA	NA
CAS-F	CAS-F-107/FR07935913	FF	DM	DM2	TruSeq Nano DNA Library Prep Kit	55.5	0.42	right liver
CAS-F	CAS-F-113/FR07935915	FF	DM	DM3	TruSeq Nano DNA Library Prep Kit	56.85	0.47	brain - Right temporal lobe
CAS-F	CAS-F-RS/FR07935922	FF	DM	DM1	TruSeq Nano DNA Library Prep Kit	76.55	0.3	right Scapula
CAS-F	CAS-F-T12-1/FR07935903	FFPE	RM	RM2	TruSeq Nano DNA Library Prep Kit	77.93	0.58	back - In-transit metastasis - core1
CAS-F	CAS-F-T12-2/FR07935909	FFPE	RM	RM1	TruSeq Nano DNA Library Prep Kit	39.78	0.44	back - In-transit metastasis - slide
CAS-F	CAS-F-T12-3/FR07935904	FFPE	RM	RM3	TruSeq Nano DNA Library Prep Kit	53.83	0.69	back - In-transit metastasis - core3
CAS-F	CAS-F-Normal/FR07935920	Buffy Coat	G	NA	TruSeq Nano DNA Library Prep Kit	27.07	NA	NA
CAS-A	CAS-A-13/FR07935916	FF	DM	DM2	TruSeq Nano DNA Library Prep Kit	63.43	0.88	spleen
CAS-A	CAS-A-1403318B-1/FR07935906	FFPE	RM	RM2	TruSeq Nano DNA Library Prep Kit	70.64	0.37	L) inguinal lymphadectomy - core1
CAS-A	CAS-A-1403318B-2/FR07935907	FFPE	RM	RM1	TruSeq Nano DNA Library Prep Kit	76.12	0.27	L) inguinal lymphadectomy - core2
CAS-A	CAS-A-1403318B-4/FR07935908	FFPE	RM	RM3	TruSeq Nano DNA Library Prep Kit	74.19	0.52	L) inguinal lymphadectomy - core4
CAS-A	CAS-A-16/FR07935894	FF	DM	DM3	TruSeq Nano DNA Library Prep Kit	69.28	0.43	liver-right lobe
CAS-A	CAS-A-20/FR07935918	FF	DM	DM4	TruSeq Nano DNA Library Prep Kit	71.91	0.94	heart-atrium
CAS-A	CAS-A-9/FR07935905	FF	DM	DM1	TruSeq Nano DNA Library Prep Kit	80.81	0.48	right kidney
CAS-A	CAS-A-Normal/FR07935921	Buffy Coat	G	NA	TruSeq Nano DNA Library Prep Kit	40.08	NA	NA
CAS-D	CAS-D-Normal/FR07935923	Buffy Coat	G	NA	TruSeq Nano DNA Library Prep Kit	38.87	NA	NA
CAS-D	CAS-D-ctDNA/FR07935886	ctDNA	ctDNA	ctDNA	TruSeq Nano DNA HT Sample Prep Kit (V1)	84	0.49	NA
CAS-D	CAS-D-64/FR07935878	FF	DM	DM2	TruSeq Nano DNA HT Sample Prep Kit (V1)	64.75	0.48	Liver Right lobe
CAS-D	CAS-D-65/FR07935879	FF	DM	DM3	TruSeq Nano DNA HT Sample Prep Kit (V1)	70.62	0.8	Liver central
CAS-D	CAS-D-66/FR07935880	FF	DM	DM4	TruSeq Nano DNA HT Sample Prep Kit (V1)	72.02	0.72	Left Liver
CAS-D	CAS-D-68/FR07935882	FF	DM	DM5	TruSeq Nano DNA HT Sample Prep Kit (V1)	77.64	0.76	brain - right hippocampus
CAS-D	CAS-D-70/FR07935883	FF	DM	DM6	TruSeq Nano DNA HT Sample Prep Kit (V1)	76.58	0.73	brain - left parietal

Supplementary Table 2. Tumour sample information for the 6 external cohort patients.

Case	Sample	Type	Figure ID	Organ
ETH-J	J-E1	RM	RM	Axilla left
ETH-J	J-L1	DM	DM1	Liver
ETH-J	J-L2	DM	DM2	Heart
ETH-J	J-L3	DM	DM3	Mediastinal lymph node
ETH-J	J-P1	P	P2	Cutaneous, scapula left
ETH-J	J-P2	P	P1	Cutaneous, scapula left
ETH-E	E-E1	RM	RM	Lumbar left
ETH-E	E-L1	DM	DM1	Peritoneum
ETH-E	E-L3	DM	DM2	Testis
ETH-E	E-L4	DM	DM3	Spleen
ETH-E	E-P	P	P	Cutaneous
ETH-F	F-L1	DM	DM1	Axilla lymph node
ETH-F	F-L2	DM	DM2	Subcutaneous shoulder right
ETH-F	F-L3	DM	DM5	Dermal flank
ETH-F	F-L4	DM	DM3	Liver
ETH-F	F-L5	DM	DM4	Lung
ETH-F	F-P1	P	P1	Cutaneous
ETH-F	F-P2	P	P2	Cutaneous
ETH-F	F-P4	P	P3	Cutaneous
MI-F	SRR2159457	DM	DM	Distant skin metastasis back
MI-F	SRR2159458	RM	RM1	Locoregional metastasis left ear
MI-F	SRR2159459	RM	RM2	Lymph node metastasis left cervical node
MI-F	SRR2159461	P	P	Primary left ear
SK-G	SRR2159462	RM	RM	Locoregional skin metastasis axilla
SK-G	SRR2159463	DM	DM	Lung metastasis
SK-G	SRR2159465	P	P	Primary right forearm
SK-H	SRR2159466	DM	DM	Brain metastasis
SK-H	SRR2159467	RM	RM1	Locoregional skin metastasis right leg
SK-H	SRR2159468	RM	RM2	Lymph node metastasis right inguinal node
SK-H	SRR2159470	P	P	Primary right leg

Supplementary Table 3. Mutations validated by targeted amplicon sequencing. LC: Low Confidence. HC: High Confidence. TP: True Positive. TN: True Negative. FP: False Positive. FN: False Negative.

Patient	Confidence	Sample	Gene	Mutation	Result	Result Type	Predicted VAF	Observed VAF (duplicate)
CAS-G	LC	DM2	ANAPC1	2;112625662;G;A	VAL	FN	0.38	0.0806 0.0758
CAS-G	LC	DM4	ANAPC1	2;112625662;G;A	VAL	FN	0.3	0.0736 0.0786
CAS-G	LC	DM1	ANAPC1	2;112625662;G;A	VAL	FN	0.47	0.0765 0.0864
CAS-G	LC	DM5	ANAPC1	2;112625662;G;A	VAL	FN	0.54	0.0886 0.0835
CAS-G	LC	DM3	ANAPC1	2;112625662;G;A	VAL	FN	0.31	0.0873 0.0947
CAS-G	LC	P	ANAPC1	2;112625662;G;A	NOT_PREDICTED_OBSERVED	FN	0	0.0773 0.1066
CAS-G	LC	RM	ANAPC1	2;112625662;G;A	NOT_PREDICTED_OBSERVED	FN	0	0.0998 0.1016
CAS-G	HC	DM2	CUL4B	X;119678398;T;A	VAL	TP	0.24	0.1308 0.1294
CAS-G	HC	DM3	CUL4B	X;119678398;T;A	NOT_PREDICTED_NOT_OBSERVED	TN	0	0 0
CAS-G	HC	DM4	CUL4B	X;119678398;T;A	NOT_PREDICTED_NOT_OBSERVED	TN	0	0 0
CAS-G	HC	DM1	CUL4B	X;119678398;T;A	NOT_PREDICTED_NOT_OBSERVED	TN	0	0 0
CAS-G	HC	DM5	CUL4B	X;119678398;T;A	NOT_PREDICTED_NOT_OBSERVED	TN	0	0 0
CAS-G	HC	P	CUL4B	X;119678398;T;A	NOT_PREDICTED_NOT_OBSERVED	TN	0	0 0
CAS-G	HC	RM	CUL4B	X;119678398;T;A	NOT_PREDICTED_NOT_OBSERVED	TN	0	0 0
CAS-G	LC	DM5	DYNLL1	12;120934240;G;A	NOT_VAL	TN	0.01	0 0
CAS-G	LC	DM2	DYNLL1	12;120934240;G;A	NOT_VAL	TN	0	0 0
CAS-G	LC	DM3	DYNLL1	12;120934240;G;A	NOT_PREDICTED_NOT_OBSERVED	TN	0	0 0
CAS-G	LC	DM4	DYNLL1	12;120934240;G;A	NOT_PREDICTED_NOT_OBSERVED	TN	0	0 0
CAS-G	LC	DM1	DYNLL1	12;120934240;G;A	NOT_PREDICTED_NOT_OBSERVED	TN	0	0 0
CAS-G	LC	P	DYNLL1	12;120934240;G;A	NOT_PREDICTED_NOT_OBSERVED	TN	0	0 0
CAS-G	LC	RM	DYNLL1	12;120934240;G;A	NOT_PREDICTED_NOT_OBSERVED	TN	0	0 0
CAS-G	LC	P	E2F5	8;86126052;C;A	NOT_VAL	TN	0.12	0 0
CAS-G	LC	DM3	E2F5	8;86126052;C;A	NOT_PREDICTED_NOT_OBSERVED	TN	0	0 0
CAS-G	LC	DM4	E2F5	8;86126052;C;A	NOT_PREDICTED_NOT_OBSERVED	TN	0	0 0
CAS-G	LC	DM1	E2F5	8;86126052;C;A	NOT_PREDICTED_NOT_OBSERVED	TN	0	0 0
CAS-G	LC	DM2	E2F5	8;86126052;C;A	NOT_PREDICTED_NOT_OBSERVED	TN	0	0 0
CAS-G	LC	DM5	E2F5	8;86126052;C;A	NOT_PREDICTED_NOT_OBSERVED	TN	0	0 0
CAS-G	LC	RM	E2F5	8;86126052;C;A	NOT_PREDICTED_NOT_OBSERVED	TN	0	0 0
CAS-G	HC	DM2	INO80	15;41337161;G;A	VAL	TP	0.77	0.7742 0.553
CAS-G	HC	DM4	INO80	15;41337161;G;A	VAL	TP	0.23	0.277 0.274
CAS-G	HC	P	INO80	15;41337161;G;A	VAL	TP	0.22	0.374 0.3956
CAS-G	HC	RM	INO80	15;41337161;G;A	VAL	TP	0.36	0.4137 0.4083
CAS-G	HC	DM3	INO80	15;41337161;G;A	VAL	TP	0.21	0.2914 0.3
CAS-G	HC	DM5	INO80	15;41337161;G;A	VAL	TP	0.24	0.278 0.2661
CAS-G	HC	DM1	INO80	15;41337161;G;A	VAL	TP	0.04	0.0343
CAS-G	HC	DM4	MLH1	3;37089083;G;A	VAL	TP	0.05	0.0531 0.0628
CAS-G	HC	DM3	MLH1	3;37089083;G;A	NOT_PREDICTED_NOT_OBSERVED	TN	0	0 0

CAS-G	HC	DM1	MLH1	3;37089083;G;A	NOT_PREDICTED_NOT_OBSERVED	TN	0	0 0
CAS-G	HC	DM2	MLH1	3;37089083;G;A	NOT_PREDICTED_NOT_OBSERVED	TN	0	0 0
CAS-G	HC	DM5	MLH1	3;37089083;G;A	NOT_PREDICTED_NOT_OBSERVED	TN	0	0 0
CAS-G	HC	P	MLH1	3;37089083;G;A	NOT_PREDICTED_NOT_OBSERVED	TN	0	0 0
CAS-G	HC	RM	MLH1	3;37089083;G;A	NOT_PREDICTED_NOT_OBSERVED	TN	0	0 0
CAS-G	HC	DM4	MSH6	2;48018128;G;A	VAL	TP	0.05	0.0308
CAS-G	HC	DM5	MSH6	2;48027175;G;A	VAL	TP	0.21	0.1938 0.1988
CAS-G	HC	DM4	MSH6	2;48033352;C;T	VAL	TP	0.11	0.1785 0.1821
CAS-G	HC	DM3	MSH6	2;48033352;C;T	VAL	TP	0.13	0.1458 0.1614
CAS-G	HC	DM1	MSH6	2;48033352;C;T	VAL	TP	0.1	0.2723 0.0431
CAS-G	HC	DM5	MSH6	2;48033791;G;A	VAL	TP	0.12	0.2172 0.2238
CAS-G	HC	DM3	MSH6	2;48018128;G;A	NOT_PREDICTED_NOT_OBSERVED	TN	0	0 0
CAS-G	HC	DM3	MSH6	2;48027175;G;A	NOT_PREDICTED_NOT_OBSERVED	TN	0	0 0
CAS-G	HC	DM3	MSH6	2;48033791;G;A	NOT_PREDICTED_NOT_OBSERVED	TN	0	0 0
CAS-G	HC	DM4	MSH6	2;48027175;G;A	NOT_PREDICTED_NOT_OBSERVED	TN	0	0 0
CAS-G	HC	DM4	MSH6	2;48033791;G;A	NOT_PREDICTED_NOT_OBSERVED	TN	0	0 0
CAS-G	HC	DM1	MSH6	2;48018128;G;A	NOT_PREDICTED_NOT_OBSERVED	TN	0	0 0
CAS-G	HC	DM1	MSH6	2;48027175;G;A	NOT_PREDICTED_NOT_OBSERVED	TN	0	0 0
CAS-G	HC	DM1	MSH6	2;48033791;G;A	NOT_PREDICTED_NOT_OBSERVED	TN	0	0 0
CAS-G	HC	DM2	MSH6	2;48018128;G;A	NOT_PREDICTED_NOT_OBSERVED	TN	0	0 0
CAS-G	HC	DM2	MSH6	2;48027175;G;A	NOT_PREDICTED_NOT_OBSERVED	TN	0	0 0
CAS-G	HC	DM2	MSH6	2;48033352;C;T	NOT_PREDICTED_NOT_OBSERVED	TN	0	0 0
CAS-G	HC	DM2	MSH6	2;48033791;G;A	NOT_PREDICTED_NOT_OBSERVED	TN	0	0 0
CAS-G	HC	DM5	MSH6	2;48018128;G;A	NOT_PREDICTED_NOT_OBSERVED	TN	0	0 0
CAS-G	HC	DM5	MSH6	2;48033352;C;T	NOT_PREDICTED_NOT_OBSERVED	TN	0	0 0
CAS-G	HC	P	MSH6	2;48018128;G;A	NOT_PREDICTED_NOT_OBSERVED	TN	0	0 0
CAS-G	HC	P	MSH6	2;48027175;G;A	NOT_PREDICTED_NOT_OBSERVED	TN	0	0 0
CAS-G	HC	P	MSH6	2;48033352;C;T	NOT_PREDICTED_NOT_OBSERVED	TN	0	0 0
CAS-G	HC	P	MSH6	2;48033791;G;A	NOT_PREDICTED_NOT_OBSERVED	TN	0	0 0
CAS-G	HC	RM	MSH6	2;48018128;G;A	NOT_PREDICTED_NOT_OBSERVED	TN	0	0 0
CAS-G	HC	RM	MSH6	2;48027175;G;A	NOT_PREDICTED_NOT_OBSERVED	TN	0	0 0
CAS-G	HC	RM	MSH6	2;48033352;C;T	NOT_PREDICTED_NOT_OBSERVED	TN	0	0 0
CAS-G	HC	RM	MSH6	2;48033791;G;A	NOT_PREDICTED_NOT_OBSERVED	TN	0	0 0
CAS-G	HC	DM2	POLR2A	17;7404625;G;A	VAL	TP	0.55	0.4433 0.4621
CAS-G	HC	RM	POLR2A	17;7404625;G;A	VAL	TP	0.43	0.4145 0.4203
CAS-G	HC	DM3	POLR2A	17;7404625;G;A	VAL	TP	0.38	0.459 0.4521
CAS-G	HC	P	POLR2A	17;7404625;G;A	VAL	TP	0.47	0.3776 0.3685
CAS-G	HC	DM5	POLR2A	17;7404625;G;A	VAL	TP	0.56	0.5889 0.5813
CAS-G	HC	DM4	POLR2A	17;7404625;G;A	VAL	TP	0.44	0.455 0.4497
CAS-G	HC	DM1	POLR2A	17;7404625;G;A	VAL	TP	0.42	0.4682 0.4415
CAS-G	LC	P	PRKCB	16;24185900;C;T	NOT_VAL	TN	0	0 0
CAS-G	LC	DM3	PRKCB	16;24185900;C;T	NOT_PREDICTED_NOT_OBSERVED	TN	0	0 0
CAS-G	LC	DM4	PRKCB	16;24185900;C;T	NOT_PREDICTED_NOT_OBSERVED	TN	0	0 0

CAS-G	LC	DM1	PRKCB	16;24185900;C;T	NOT_PREDICTED_NOT_OBSERVED	TN	0	0 0
CAS-G	LC	DM2	PRKCB	16;24185900;C;T	NOT_PREDICTED_NOT_OBSERVED	TN	0	0 0
CAS-G	LC	DM5	PRKCB	16;24185900;C;T	NOT_PREDICTED_NOT_OBSERVED	TN	0	0 0
CAS-G	LC	RM	PRKCB	16;24185900;C;T	NOT_PREDICTED_NOT_OBSERVED	TN	0	0 0
CAS-G	LC	DM2	RHNO1	12;2996279;T;C	VAL	FN	0.06	0.26603325 0.25462013
CAS-G	LC	P	RHNO1	12;2996279;T;C	VAL	FN	0.33	0.21978022 0.25714285
CAS-G	LC	DM3	RHNO1	12;2996279;T;C	NOT_PREDICTED_OBSERVED	FN	0	0.18574514 0.18656717
CAS-G	LC	DM4	RHNO1	12;2996279;T;C	NOT_PREDICTED_OBSERVED	FN	0	0.10558069 0.13237222
CAS-G	LC	DM1	RHNO1	12;2996279;T;C	NOT_PREDICTED_OBSERVED	FN	0	0.24878048 0.1908832
CAS-G	LC	DM5	RHNO1	12;2996279;T;C	NOT_PREDICTED_OBSERVED	FN	0	0.16303101 0.1644005
CAS-G	LC	RM	RHNO1	12;2996279;T;C	NOT_PREDICTED_OBSERVED	FN	0	0.16228748 0.17154255
CAS-G	LC	DM4	TOPBP1	3;133374283;G;A	VAL	FN	0.1	0.067 0.0691
CAS-G	LC	DM1	TOPBP1	3;133374283;G;A	NOT_VAL	TN	0.01	0 0
CAS-G	LC	DM3	TOPBP1	3;133374283;G;A	NOT_PREDICTED_NOT_OBSERVED	TN	0	0 0
CAS-G	LC	DM2	TOPBP1	3;133374283;G;A	NOT_PREDICTED_NOT_OBSERVED	TN	0	0 0
CAS-G	LC	DM5	TOPBP1	3;133374283;G;A	NOT_PREDICTED_NOT_OBSERVED	TN	0	0 0
CAS-G	LC	P	TOPBP1	3;133374283;G;A	NOT_PREDICTED_NOT_OBSERVED	TN	0	0 0
CAS-G	LC	RM	TOPBP1	3;133374283;G;A	NOT_PREDICTED_NOT_OBSERVED	TN	0	0 0
CAS-G	HC	DM2	UBE2T	1;202302637;G;A	VAL	TP	0.5	0.4164 0.4314
CAS-G	HC	RM	UBE2T	1;202302637;G;A	VAL	TP	0.35	0.4144 0.4026
CAS-G	HC	DM5	UBE2T	1;202302637;G;A	VAL	TP	0.4	0.4681 0.4681
CAS-G	HC	DM4	UBE2T	1;202302637;G;A	VAL	TP	0.3	0.4349 0.4459
CAS-G	HC	P	UBE2T	1;202302637;G;A	VAL	TP	0.39	0.3424 0.3766
CAS-G	HC	DM1	UBE2T	1;202302637;G;A	VAL	TP	0.39	0.4294 0.4349
CAS-G	HC	DM3	UBE2T	1;202302637;G;A	VAL	TP	0.37	0.4599 0.4638
CAS-D	LC	DM4	ANKLE2	12;133310637;T;C	NOT_VAL	TN	0.01	0 0
CAS-D	LC	DM2	ANKLE2	12;133310637;T;C	VAL	FN	0.02	0.05472637
CAS-D	LC	P2	ANKLE2	12;133310637;T;C	NOT_PREDICTED_NOT_OBSERVED	TN	0	0 0
CAS-D	LC	P3	ANKLE2	12;133310637;T;C	NOT_PREDICTED_NOT_OBSERVED	TN	0	0 0
CAS-D	LC	DM5	ANKLE2	12;133310637;T;C	NOT_PREDICTED_NOT_OBSERVED	TN	0	0 0
CAS-D	LC	RM	ANKLE2	12;133310637;T;C	NOT_PREDICTED_NOT_OBSERVED	TN	0	0 0
CAS-D	LC	DM6	ANKLE2	12;133310637;T;C	NOT_PREDICTED_OBSERVED	FN	0	0.06374502
CAS-D	HC	DM5	CLTC	17;57760301;C;T	VAL	TP	0.77	0.7215 0.741
CAS-D	HC	DM4	CLTC	17;57760301;C;T	VAL	TP	0.66	0.7034 0.7038
CAS-D	HC	DM6	CLTC	17;57760301;C;T	VAL	TP	0.73	0.7193 0.7147
CAS-D	HC	DM2	CLTC	17;57760301;C;T	VAL	TP	0.46	0.4874 0.488
CAS-D	HC	P3	CLTC	17;57760301;C;T	VAL	TP	0.2	0.2044 0.2254
CAS-D	HC	P2	CLTC	17;57760301;C;T	VAL	TP	0.45	0.5438 0.554
CAS-D	HC	RM	CLTC	17;57760301;C;T	VAL	TP	0.7	0.6918 0.7443
CAS-D	HC	DM6	FGFR1OP	6;167417807;C;CA	VAL	TP	0.33	0.4209 0.436
CAS-D	HC	DM2	FGFR1OP	6;167417807;C;CA	VAL	TP	0.27	0.3465 0.3441
CAS-D	HC	DM5	FGFR1OP	6;167417807;C;CA	VAL	TP	0.39	0.4597 0.4525
CAS-D	HC	P2	FGFR1OP	6;167417807;C;CA	VAL	TP	0.3	0.3561 0.3337

CAS-D	HC	P3	FGFR1OP	6;167417807;C;CA	VAL	TP	0.1	0.1941 0.1781
CAS-D	HC	DM4	FGFR1OP	6;167417807;C;CA	VAL	TP	0.78	0.7226 0.7187
CAS-D	HC	RM	FGFR1OP	6;167417807;C;CA	VAL	TP	0.5	0.4878 0.5227
CAS-D	HC	DM5	NUP155	5;37350306;G;A	VAL	TP	0.77	0.6948 0.7098
CAS-D	HC	DM4	NUP155	5;37350306;G;A	VAL	TP	0.67	0.6887 0.6853
CAS-D	HC	DM6	NUP155	5;37350306;G;A	VAL	TP	0.71	0.6979 0.6936
CAS-D	HC	P2	NUP155	5;37350306;G;A	VAL	TP	0.34	0.3565 0.3552
CAS-D	HC	DM2	NUP155	5;37350306;G;A	VAL	TP	0.52	0.4501 0.4442
CAS-D	HC	P3	NUP155	5;37350306;G;A	VAL	TP	0.12	0.1621 0.1538
CAS-D	HC	RM	NUP155	5;37350306;G;A	VAL	TP	0.74	0.7004 0.6965
CAS-D	HC	DM2	PSMB11	14;23511991;G;A	VAL	TP	0.26	0.2553 0.2699
CAS-D	HC	DM5	PSMB11	14;23511991;G;A	VAL	TP	0.27	0.2714 0.2797
CAS-D	HC	DM6	PSMB11	14;23511991;G;A	VAL	TP	0.25	0.3046 0.3019
CAS-D	HC	DM4	PSMB11	14;23511991;G;A	VAL	TP	0.25	0.2995 0.3035
CAS-D	HC	RM	PSMB11	14;23511991;G;A	VAL	TP	0.33	0.4509 0.4671
CAS-D	HC	P3	PSMB11	14;23511991;G;A	VAL	TP	0.15	0.1648 0.159
CAS-D	HC	P2	PSMB11	14;23511991;G;A	VAL	TP	0.31	0.3792 0.3773
CAS-D	LC	DM2	TACC3	4;1729764;ACAAAGCGGAGACTCCGCACGGAGCCGAGGAAGAATG;A	NOT_VAL	TN	0.17	0 0
CAS-D	LC	P2	TACC3	4;1729764;ACAAAGCGGAGACTCCGCACGGAGCCGAGGAAGAATG;A	NOT_PREDICTED_NOT_OBSERVED	TN	0	0 0
CAS-D	LC	P3	TACC3	4;1729764;ACAAAGCGGAGACTCCGCACGGAGCCGAGGAAGAATG;A	NOT_PREDICTED_NOT_OBSERVED	TN	0	0 0
CAS-D	LC	DM4	TACC3	4;1729764;ACAAAGCGGAGACTCCGCACGGAGCCGAGGAAGAATG;A	NOT_PREDICTED_NOT_OBSERVED	TN	0	0 0
CAS-D	LC	DM5	TACC3	4;1729764;ACAAAGCGGAGACTCCGCACGGAGCCGAGGAAGAATG;A	NOT_PREDICTED_NOT_OBSERVED	TN	0	0 0
CAS-D	LC	DM6	TACC3	4;1729764;ACAAAGCGGAGACTCCGCACGGAGCCGAGGAAGAATG;A	NOT_PREDICTED_NOT_OBSERVED	TN	0	0 0
CAS-D	LC	RM	TACC3	4;1729764;ACAAAGCGGAGACTCCGCACGGAGCCGAGGAAGAATG;A	NOT_PREDICTED_NOT_OBSERVED	TN	0	0 0
CAS-D	HC	DM4	TP53	17;7577539;G;A	VAL	TP	0.64	0.6723 0.677
CAS-D	HC	DM2	TP53	17;7577539;G;A	VAL	TP	0.21	0.361 0.3576
CAS-D	HC	DM5	TP53	17;7577539;G;A	VAL	TP	0.76	0.6759 0.6648
CAS-D	HC	DM6	TP53	17;7577539;G;A	VAL	TP	0.55	0.6852 0.671
CAS-D	HC	P3	TP53	17;7577539;G;A	VAL	TP	0.11	0.1922 0.1939
CAS-D	HC	RM	TP53	17;7577539;G;A	VAL	TP	0.65	0.7761 0.7381
CAS-D	HC	P2	TP53	17;7577539;G;A	VAL	TP	0.52	0.5394 0.5642
CAS-D	LC	DM2	UBC	12;125397821;G;A	NOT_VAL	TN	0.3	0 0
CAS-D	LC	DM6	UBC	12;125397821;G;A	NOT_VAL	TN	0.2	0 0
CAS-D	LC	RM	UBC	12;125397821;G;A	NOT_VAL	TN	0.22	0 0
CAS-D	LC	P2	UBC	12;125397821;G;A	NOT_VAL	TN	0.16	0 0
CAS-D	LC	DM5	UBC	12;125397821;G;A	NOT_VAL	TN	0.14	0 0
CAS-D	LC	P3	UBC	12;125397821;G;A	NOT_VAL	TN	0.18	0 0
CAS-D	LC	DM4	UBC	12;125397821;G;A	NOT_VAL	TN	0.33	0 0
CAS-F	HC	DM2	ALMS1	2;73717382;C;A	VAL	TP	0.3	0.2957 0.3144
CAS-F	HC	RM1	ALMS1	2;73717382;C;A	VAL	TP	0.32	0.3011 0.2786
CAS-F	HC	RM2	ALMS1	2;73717382;C;A	VAL	TP	0.33	0.393 0.377

CAS-F	HC	RM3	ALMS1	2;73717382;C;A	VAL	TP	0.39	0.4379 0.4261
CAS-F	HC	DM1	ALMS1	2;73717382;C;A	VAL	TP	0.14	0.2212 0.2395
CAS-F	HC	DM3	ALMS1	2;73717382;C;A	VAL	TP	0.3	0.3136 0.3157
CAS-F	HC	RM3	ANAPC1	2;112614429;G;A	VAL	TP	0.05	0.1154 0.1248
CAS-F	HC	RM2	ANAPC1	2;112614429;G;A	VAL	TP	0.11	0.106 0.1062
CAS-F	HC	DM3	ANAPC1	2;112614429;G;A	VAL	TP	0.09	0.0499 0.0409
CAS-F	HC	RM1	ANAPC1	2;112614429;G;A	VAL	TP	0.09	0.1756 0.1448
CAS-F	HC	DM2	ANAPC1	2;112614429;G;A	VAL	TP	0.05	0.1155 0.1384
CAS-F	HC	DM1	ANAPC1	2;112614429;G;A	VAL	TP	0.01	0.131 0.1199
CAS-F	LC	RM3	BRCA2	13;32970126;A;T	NOT_VAL	TN	0.21	0 0
CAS-F	LC	RM2	BRCA2	13;32970126;A;T	NOT_PREDICTED_NOT_OBSERVED	TN	0	0 0
CAS-F	LC	RM1	BRCA2	13;32970126;A;T	NOT_PREDICTED_NOT_OBSERVED	TN	0	0 0
CAS-F	LC	DM2	BRCA2	13;32970126;A;T	NOT_PREDICTED_NOT_OBSERVED	TN	0	0 0
CAS-F	LC	DM3	BRCA2	13;32970126;A;T	NOT_PREDICTED_NOT_OBSERVED	TN	0	0 0
CAS-F	LC	DM1	BRCA2	13;32970126;A;T	NOT_PREDICTED_NOT_OBSERVED	TN	0	0 0
CAS-F	HC	DM2	CDKN2A	9;21971017;G;A	VAL	TP	0.48	0.4489 0.4232
CAS-F	HC	RM2	CDKN2A	9;21971017;G;A	VAL	TP	0.64	0.854 0.8411
CAS-F	HC	DM3	CDKN2A	9;21971017;G;A	VAL	TP	0.42	0.4818 0.4577
CAS-F	HC	RM3	CDKN2A	9;21971017;G;A	VAL	TP	0.92	0.8928 0.8667
CAS-F	HC	RM1	CDKN2A	9;21971017;G;A	VAL	TP	0.59	0.5092 0.3659
CAS-F	HC	DM1	CDKN2A	9;21971017;G;A	VAL	TP	0.4	0.3806 0.4229
CAS-F	LC	RM3	ENSA	1;150573651;A;G	NOT_VAL	TN	0.06	0 0
CAS-F	LC	RM2	ENSA	1;150573651;A;G	NOT_VAL	TN	0.09	0 0
CAS-F	LC	RM1	ENSA	1;150573651;A;G	NOT_PREDICTED_NOT_OBSERVED	TN	0	0 0
CAS-F	LC	DM2	ENSA	1;150573651;A;G	NOT_PREDICTED_NOT_OBSERVED	TN	0	0 0
CAS-F	LC	DM3	ENSA	1;150573651;A;G	NOT_PREDICTED_NOT_OBSERVED	TN	0	0 0
CAS-F	LC	DM1	ENSA	1;150573651;A;G	NOT_PREDICTED_NOT_OBSERVED	TN	0	0 0
CAS-F	HC	RM3	FEN1	11;61563864;T;G	VAL	TP	0.62	0.6474 0.6054
CAS-F	HC	RM2	FEN1	11;61563864;T;G	VAL	TP	0.52	0.564 0.5383
CAS-F	HC	DM1	FEN1	11;61563864;T;G	VAL	TP	0.39	0.3835 0.404
CAS-F	HC	DM2	FEN1	11;61563864;T;G	VAL	TP	0.55	0.4862 0.4707
CAS-F	HC	RM1	FEN1	11;61563864;T;G	VAL	TP	0.5	0.3889 0.41573032
CAS-F	HC	DM3	FEN1	11;61563864;T;G	VAL	TP	0.37	0.4704 0.4769
CAS-F	HC	RM2	KIF2B	17;51900950;G;A	VAL	TP	0.5	0.4558 0.4615
CAS-F	HC	DM3	KIF2B	17;51900950;G;A	VAL	TP	0.21	0.1854 0.1843
CAS-F	HC	RM3	KIF2B	17;51900950;G;A	VAL	TP	0.47	0.5191 0.5249
CAS-F	HC	RM1	KIF2B	17;51900950;G;A	VAL	TP	0.34	0.3351 0.3499
CAS-F	HC	DM2	KIF2B	17;51900950;G;A	VAL	TP	0.28	0.3531 0.3312
CAS-F	HC	DM1	KIF2B	17;51900950;G;A	VAL	TP	0.32	0.2602 0.2672
CAS-F	HC	RM2	MDC1	6;30672477;C;T	VAL	TP	0.23	0.2654 0.2875
CAS-F	HC	RM3	MDC1	6;30672477;C;T	VAL	TP	0.24	0.3115 0.2962
CAS-F	HC	DM2	MDC1	6;30672477;C;T	VAL	TP	0.22	0.176 0.1753
CAS-F	HC	DM3	MDC1	6;30672477;C;T	VAL	TP	0.42	0.3971 0.4099

CAS-F	HC	DM1	MDC1	6;30672477;C;T	VAL	TP	0.16	0.1132 0.1407
CAS-F	HC	RM1	MDC1	6;30672477;C;T	VAL	TP	0.12	0.1362 0.1357
CAS-F	HC	DM2	MYO10	5;16701934;C;A	VAL	TP	0.4	0.4024 0.4185
CAS-F	HC	RM3	MYO10	5;16701934;C;A	VAL	TP	0.57	0.4915 0.4922
CAS-F	HC	DM3	MYO10	5;16701934;C;A	VAL	TP	0.11	0.2183 0.2216
CAS-F	HC	RM2	MYO10	5;16701934;C;A	VAL	TP	0.41	0.4777 0.4615
CAS-F	HC	DM1	MYO10	5;16701934;C;A	VAL	TP	0.26	0.3346 0.3419
CAS-F	HC	RM1	MYO10	5;16701934;C;A	VAL	TP	0.45	0.3345 0.3494
CAS-F	HC	RM2	NFRKB	11;129743677;A;G	VAL	TP	0.07	0.1261 0.1079
CAS-F	HC	RM3	NFRKB	11;129743677;A;G	VAL	TP	0.24	0.1748 0.1848
CAS-F	HC	RM1	NFRKB	11;129743677;A;G	VAL	TP	0.07	0.1424 0.0985
CAS-F	HC	DM2	NFRKB	11;129743677;A;G	NOT_PREDICTED_NOT_OBSERVED	TN	0	0 0
CAS-F	HC	DM3	NFRKB	11;129743677;A;G	NOT_PREDICTED_NOT_OBSERVED	TN	0	0 0
CAS-F	HC	DM1	NFRKB	11;129743677;A;G	NOT_PREDICTED_NOT_OBSERVED	TN	0	0 0
CAS-F	HC	DM1	POLR2A	17;7404163;C;T	VAL	TP	0.05	0.15 0.1469
CAS-F	HC	DM3	POLR2A	17;7404163;C;T	VAL	TP	0.41	0.3803 0.3862
CAS-F	HC	RM1	POLR2A	17;7404163;C;T	VAL	TP	0.08	0.2205 0.2213
CAS-F	HC	RM3	POLR2A	17;7404163;C;T	VAL	TP	0.3	0.2955 0.3192
CAS-F	HC	RM2	POLR2A	17;7404163;C;T	VAL	TP	0.3	0.262 0.2605
CAS-F	HC	DM2	POLR2A	17;7404163;C;T	VAL	TP	0.16	0.1917 0.1755
CAS-F	HC	DM1	POM121	7;72396963;C;T	VAL	TP	0.38	0.4594 0.4579
CAS-F	HC	DM2	POM121	7;72396963;C;T	VAL	TP	0.59	0.4966 0.5031
CAS-F	HC	RM2	POM121	7;72396963;C;T	VAL	TP	0.63	0.6417 0.5981
CAS-F	HC	RM3	POM121	7;72396963;C;T	VAL	TP	0.67	0.6646 0.6465
CAS-F	HC	DM3	POM121	7;72396963;C;T	VAL	TP	0.53	0.5842 0.5773
CAS-F	HC	RM1	POM121	7;72396963;C;T	VAL	TP	0.52	0.528 0.5187
CAS-F	HC	RM2	POM121	7;72396964;C;T	VAL	TP	0.63	0.645 0.6019
CAS-F	HC	DM2	POM121	7;72396964;C;T	VAL	TP	0.57	0.4968 0.5041
CAS-F	HC	RM1	POM121	7;72396964;C;T	VAL	TP	0.54	0.5315 0.5208
CAS-F	HC	RM3	POM121	7;72396964;C;T	VAL	TP	0.67	0.6625 0.6525
CAS-F	HC	DM1	POM121	7;72396964;C;T	VAL	TP	0.38	0.4602 0.4576
CAS-F	HC	DM3	POM121	7;72396964;C;T	VAL	TP	0.53	0.5863 0.5785
CAS-F	HC	RM2	RNF4	4;2515439;C;T	VAL	TP	0.57	0.4776 0.4851
CAS-F	HC	RM3	RNF4	4;2515439;C;T	VAL	TP	0.55	0.515 0.5017
CAS-F	HC	RM1	RNF4	4;2515439;C;T	VAL	TP	0.42	0.3497 0.3964
CAS-F	HC	DM2	RNF4	4;2515439;C;T	VAL	TP	0.36	0.378 0.391
CAS-F	HC	DM1	RNF4	4;2515439;C;T	VAL	TP	0.23	0.352 0.324
CAS-F	HC	DM3	RNF4	4;2515439;C;T	VAL	TP	0.23	0.3598 0.3625
CAS-F	LC	RM3	SMUG1	12;54582294;C;G	NOT_VAL	TN	0.07	0 0
CAS-F	LC	RM2	SMUG1	12;54582294;C;G	NOT_VAL	TN	0.01	0 0
CAS-F	LC	RM1	SMUG1	12;54582294;C;G	NOT_PREDICTED_NOT_OBSERVED	TN	0	0 0
CAS-F	LC	DM2	SMUG1	12;54582294;C;G	NOT_PREDICTED_NOT_OBSERVED	TN	0	0 0
CAS-F	LC	DM3	SMUG1	12;54582294;C;G	NOT_PREDICTED_NOT_OBSERVED	TN	0	0 0

CAS-F	LC	DM1	SMUG1	12;54582294;C;G	NOT_PREDICTED_NOT_OBSERVED	TN	0	0 0
CAS-F	LC	RM2	TUBB4A	19;6496379;A;G	VAL	FN	0.1	0.0935 0.1078
CAS-F	LC	RM3	TUBB4A	19;6496379;A;G	NOT_PREDICTED_OBSERVED	FN	0	0.0888 0.0742
CAS-F	LC	RM1	TUBB4A	19;6496379;A;G	NOT_PREDICTED_OBSERVED	FN	0	0.044
CAS-F	LC	DM2	TUBB4A	19;6496379;A;G	NOT_PREDICTED_OBSERVED	FN	0	0.1031 0.1029
CAS-F	LC	DM3	TUBB4A	19;6496379;A;G	NOT_PREDICTED_OBSERVED	FN	0	0.0929 0.1141
CAS-F	LC	DM1	TUBB4A	19;6496379;A;G	NOT_PREDICTED_OBSERVED	FN	0	0.0322 0.03084416
CAS-F	HC	DM3	WRN	8;30974004;G;A	VAL	TP	0.24	0.2602 0.2597
CAS-F	HC	RM1	WRN	8;30974004;G;A	VAL	TP	0.07	0.1884 0.2074
CAS-F	HC	RM3	WRN	8;30974004;G;A	VAL	TP	0.33	0.2844 0.2554
CAS-F	HC	DM1	WRN	8;30974004;G;A	VAL	TP	0.09	0.1943 0.1798
CAS-F	HC	RM2	WRN	8;30974004;G;A	VAL	TP	0.3	0.2688 0.2801
CAS-F	HC	DM2	WRN	8;30974004;G;A	NOT_PREDICTED_NOT_OBSERVED	TN	0	0 0
CAS-E	HC	DM1	ASCC2	22;30221691;C;T	VAL	TP	0.49	0.4664 0.4764
CAS-E	HC	DM3	ASCC2	22;30221691;C;T	VAL	TP	0.38	0.4505 0.4497
CAS-E	HC	P	ASCC2	22;30221691;C;T	VAL	TP	0.18	0.197 0.2092
CAS-E	HC	DM2	ASCC2	22;30221691;C;T	VAL	TP	0.28	0.3285 0.3297
CAS-E	HC	RM	ASCC2	22;30221691;C;T	VAL	TP	0.22	0.2697 0.2678
CAS-E	HC	DM5	ASCC2	22;30221691;C;T	VAL	TP	0.33	0.3858 0.3866
CAS-E	LC	DM1	BRCA2	13;32970126;A;T	NOT_VAL	TN	0.15	0 0
CAS-E	LC	RM	BRCA2	13;32970126;A;T	NOT_PREDICTED_NOT_OBSERVED	TN	0	0 0
CAS-E	LC	DM2	BRCA2	13;32970126;A;T	NOT_PREDICTED_NOT_OBSERVED	TN	0	0 0
CAS-E	LC	DM3	BRCA2	13;32970126;A;T	NOT_PREDICTED_NOT_OBSERVED	TN	0	0 0
CAS-E	LC	DM5	BRCA2	13;32970126;A;T	NOT_PREDICTED_NOT_OBSERVED	TN	0	0 0
CAS-E	LC	P	BRCA2	13;32970126;A;T	NOT_PREDICTED_NOT_OBSERVED	TN	0	0 0
CAS-E	HC	DM1	CDKN2A	9;21974714;GGCAGCGCCCCCGCCTCCA;G	VAL	TP	0.51	0.8977 0.8958
CAS-E	HC	DM2	CDKN2A	9;21974714;GGCAGCGCCCCCGCCTCCA;G	VAL	TP	0.39	0.7662 0.7872
CAS-E	HC	DM3	CDKN2A	9;21974714;GGCAGCGCCCCCGCCTCCA;G	VAL	TP	0.45	0.8901 0.8871
CAS-E	HC	DM5	CDKN2A	9;21974714;GGCAGCGCCCCCGCCTCCA;G	VAL	TP	0.4	0.8019 0.7935
CAS-E	HC	P	CDKN2A	9;21974714;GGCAGCGCCCCCGCCTCCA;G	VAL	TP	0.25	0.5726 0.6149
CAS-E	HC	RM	CDKN2A	9;21974714;GGCAGCGCCCCCGCCTCCA;G	VAL	TP	0.29	0.638 0.6444
CAS-E	HC	P	CEP290	12;88483003;G;A	VAL	TP	0.13	0.1085 0.1086
CAS-E	HC	RM	CEP290	12;88483003;G;A	VAL	TP	0.14	0.1832 0.1557
CAS-E	HC	DM1	CEP290	12;88483003;G;A	VAL	TP	0.32	0.3289 0.334
CAS-E	HC	DM5	CEP290	12;88483003;G;A	VAL	TP	0.23	0.2457 0.2408
CAS-E	HC	DM2	CEP290	12;88483003;G;A	VAL	TP	0.16	0.2195 0.2232
CAS-E	HC	DM3	CEP290	12;88483003;G;A	VAL	TP	0.25	0.2975 0.2894
CAS-E	HC	DM3	COPS6	7;99686912;G;A	VAL	TP	0.29	0.3632 0.3757
CAS-E	HC	DM2	COPS6	7;99686912;G;A	VAL	TP	0.28	0.315 0.3133
CAS-E	HC	DM5	COPS6	7;99686912;G;A	VAL	TP	0.35	0.3602 0.3938
CAS-E	HC	P	COPS6	7;99686912;G;A	VAL	TP	0.23	0.238 0.2285
CAS-E	HC	DM1	COPS6	7;99686912;G;A	VAL	TP	0.42	0.4436 0.4447
CAS-E	HC	RM	COPS6	7;99686912;G;A	VAL	TP	0.26	0.2376 0.2461

CAS-E	HC	DM2	CSNK2A1	20;478391;G;A	VAL	TP	0.26	0.3482 0.3436
CAS-E	HC	DM3	CSNK2A1	20;478391;G;A	VAL	TP	0.33	0.442 0.4195
CAS-E	HC	DM1	CSNK2A1	20;478391;G;A	VAL	TP	0.45	0.4448 0.4603
CAS-E	HC	P	CSNK2A1	20;478391;G;A	VAL	TP	0.31	0.2235 0.2262
CAS-E	HC	RM	CSNK2A1	20;478391;G;A	VAL	TP	0.14	0.2341 0.2471
CAS-E	HC	DM5	CSNK2A1	20;478391;G;A	VAL	TP	0.35	0.4002 0.3912
CAS-E	HC	DM1	DYNC1H1	14;102482274;C;T	VAL	TP	0.37	0.3971 0.3784
CAS-E	HC	DM5	DYNC1H1	14;102482274;C;T	VAL	TP	0.28	0.3288 0.3231
CAS-E	HC	RM	DYNC1H1	14;102482274;C;T	VAL	TP	0.25	0.2565 0.2608
CAS-E	HC	DM3	DYNC1H1	14;102482274;C;T	VAL	TP	0.4	0.3762 0.3841
CAS-E	HC	DM2	DYNC1H1	14;102482274;C;T	VAL	TP	0.27	0.2957 0.2935
CAS-E	HC	P	DYNC1H1	14;102482274;C;T	VAL	TP	0.21	0.2576 0.2448
CAS-E	HC	DM1	EMD	X;153609403;G;A	VAL	TP	0.88	0.8645 0.8707
CAS-E	HC	DM2	EMD	X;153609403;G;A	VAL	TP	0.69	0.7087 0.7145
CAS-E	HC	DM3	EMD	X;153609403;G;A	VAL	TP	0.84	0.8247 0.8168
CAS-E	HC	DM5	EMD	X;153609403;G;A	VAL	TP	0.78	0.7401 0.7367
CAS-E	HC	P	EMD	X;153609403;G;A	VAL	TP	0.43	0.4439 0.4084
CAS-E	HC	RM	EMD	X;153609403;G;A	VAL	TP	0.41	0.4482 0.4605
CAS-E	HC	DM5	FZR1	19;3533325;C;T	VAL	TP	0.24	0.1807 0.1922
CAS-E	HC	DM1	FZR1	19;3533325;C;T	NOT_PREDICTED_NOT_OBSERVED	TN	0	0 0
CAS-E	HC	RM	FZR1	19;3533325;C;T	NOT_PREDICTED_NOT_OBSERVED	TN	0	0 0
CAS-E	HC	DM2	FZR1	19;3533325;C;T	NOT_PREDICTED_NOT_OBSERVED	TN	0	0 0
CAS-E	HC	DM3	FZR1	19;3533325;C;T	NOT_PREDICTED_NOT_OBSERVED	TN	0	0 0
CAS-E	HC	P	FZR1	19;3533325;C;T	NOT_PREDICTED_NOT_OBSERVED	TN	0	0 0
CAS-E	HC	DM3	MYO10	5;16676209;G;A	VAL	TP	0.61	0.5566 0.555
CAS-E	HC	RM	MYO10	5;16676209;G;A	VAL	TP	0.34	0.3318 0.3485
CAS-E	HC	P	MYO10	5;16676209;G;A	VAL	TP	0.29	0.3011 0.3027
CAS-E	HC	DM2	MYO10	5;16676209;G;A	VAL	TP	0.35	0.437 0.4167
CAS-E	HC	DM5	MYO10	5;16676209;G;A	VAL	TP	0.44	0.4954 0.4761
CAS-E	HC	DM1	MYO10	5;16676209;G;A	VAL	TP	0.58	0.5807 0.6115
CAS-E	HC	RM	NEIL1	15;75647322;A;G	VAL	TP	0.28	0.2197 0.2289
CAS-E	HC	DM1	NEIL1	15;75647322;A;G	VAL	TP	0.22	0.2918 0.2771
CAS-E	HC	DM5	NEIL1	15;75647322;A;G	VAL	TP	0.35	0.3418 0.36
CAS-E	HC	P	NEIL1	15;75647322;A;G	VAL	TP	0.25	0.2096 0.2142
CAS-E	HC	DM3	NEIL1	15;75647322;A;G	VAL	TP	0.47	0.4119 0.4161
CAS-E	HC	DM2	NEIL1	15;75647322;A;G	VAL	TP	0.26	0.3034 0.2992
CAS-E	HC	DM1	PDS5B	13;33233358;G;A	VAL	TP	0.66	0.6289 0.6463
CAS-E	HC	DM3	PDS5B	13;33233358;G;A	VAL	TP	0.58	0.6172 0.6253
CAS-E	HC	P	PDS5B	13;33233358;G;A	VAL	TP	0.15	0.2643 0.2568
CAS-E	HC	DM2	PDS5B	13;33233358;G;A	VAL	TP	0.43	0.5006 0.5352
CAS-E	HC	RM	PDS5B	13;33233358;G;A	VAL	TP	0.3	0.3104 0.3049
CAS-E	HC	DM5	PDS5B	13;33233358;G;A	VAL	TP	0.57	0.5463 0.5515
CAS-E	LC	DM2	REST	4;57798316;T;C	VAL	FN	0.23	0.2281 0.2314

CAS-E	LC	DM5	REST	4;57798316;T;C	VAL	FN	0.26	0.2436 0.2361
CAS-E	LC	DM3	REST	4;57798316;T;C	VAL	FN	0.32	0.2904 0.2963
CAS-E	LC	DM1	REST	4;57798316;T;C	VAL	FN	0.14	0.1538 0.1507
CAS-E	LC	RM	REST	4;57798316;T;C	NOT_PREDICTED_NOT_OBSERVED	TN	0	0 0
CAS-E	LC	P	REST	4;57798316;T;C	NOT_PREDICTED_NOT_OBSERVED	TN	0	0 0
CAS-E	HC	P	RNF168	3;196199162;G;A	VAL	TP	0.2	0.2624 0.2513
CAS-E	HC	DM1	RNF168	3;196199162;G;A	VAL	TP	0.56	0.5839 0.5843
CAS-E	HC	DM3	RNF168	3;196199162;G;A	VAL	TP	0.46	0.5251 0.526
CAS-E	HC	DM5	RNF168	3;196199162;G;A	VAL	TP	0.4	0.4747 0.4687
CAS-E	HC	RM	RNF168	3;196199162;G;A	VAL	TP	0.26	0.3248 0.3275
CAS-E	HC	DM2	RNF168	3;196199162;G;A	VAL	TP	0.47	0.4438 0.4569
CAS-E	HC	DM5	SMC3	10;112349666;G;A	VAL	TP	0.28	0.2453 0.2079
CAS-E	HC	DM1	SMC3	10;112349666;G;A	NOT_PREDICTED_NOT_OBSERVED	TN	0	0 0
CAS-E	HC	RM	SMC3	10;112349666;G;A	NOT_PREDICTED_NOT_OBSERVED	TN	0	0 0
CAS-E	HC	DM2	SMC3	10;112349666;G;A	NOT_PREDICTED_NOT_OBSERVED	TN	0	0 0
CAS-E	HC	DM3	SMC3	10;112349666;G;A	NOT_PREDICTED_NOT_OBSERVED	TN	0	0 0
CAS-E	HC	P	SMC3	10;112349666;G;A	NOT_PREDICTED_NOT_OBSERVED	TN	0	0 0
CAS-E	HC	RM	TP53	17;7578472;G;A	VAL	TP	0.17	0.2017 0.1997
CAS-E	HC	DM5	TP53	17;7578472;G;A	VAL	TP	0.22	0.2641 0.2576
CAS-E	HC	DM3	TP53	17;7578472;G;A	VAL	TP	0.25	0.4024 0.3912
CAS-E	HC	P	TP53	17;7578472;G;A	VAL	TP	0.14	0.2049 0.2022
CAS-E	HC	DM2	TP53	17;7578472;G;A	VAL	TP	0.25	0.3241 0.3303
CAS-E	HC	DM1	TP53	17;7578472;G;A	VAL	TP	0.24	0.4166 0.4172
CAS-A	HC	RM1	APC	5;112173567;C;T	VAL	TP	0.17	0.2425 0.2539
CAS-A	HC	DM2	APC	5;112173567;C;T	VAL	TP	0.54	0.5386 0.532
CAS-A	HC	DM3	APC	5;112173567;C;T	VAL	TP	0.45	0.498 0.4863
CAS-A	HC	RM3	APC	5;112173567;C;T	VAL	TP	0.32	0.3576 0.3613
CAS-A	HC	DM1	APC	5;112173567;C;T	VAL	TP	0.32	0.3859 0.3957
CAS-A	HC	DM4	APC	5;112173567;C;T	VAL	TP	0.43	0.4858 0.4974
CAS-A	HC	RM2	APC	5;112173567;C;T	VAL	TP	0.21	0.2868 0.3255
CAS-A	LC	RM2	CEP164	11;117222647;G;GA	NOT_VAL	TN	0.09	0 0
CAS-A	LC	DM4	CEP164	11;117222647;G;GA	NOT_VAL	TN	0.15	0 0
CAS-A	LC	DM3	CEP164	11;117222647;G;GA	NOT_PREDICTED_NOT_OBSERVED	TN	0	0 0
CAS-A	LC	DM1	CEP164	11;117222647;G;GA	NOT_PREDICTED_NOT_OBSERVED	TN	0	0 0
CAS-A	LC	RM1	CEP164	11;117222647;G;GA	NOT_PREDICTED_NOT_OBSERVED	TN	0	0 0
CAS-A	LC	RM3	CEP164	11;117222647;G;GA	NOT_PREDICTED_NOT_OBSERVED	TN	0	0 0
CAS-A	LC	DM2	CEP164	11;117222647;G;GA	NOT_PREDICTED_NOT_OBSERVED	TN	0	0 0
CAS-A	HC	DM3	ENSA	1;150599971;T;A	VAL	TP	0.24	0.3013 0.2951
CAS-A	HC	DM1	ENSA	1;150599971;T;A	VAL	TP	0.46	0.4526 0.4683
CAS-A	HC	RM2	ENSA	1;150599971;T;A	VAL	TP	0.14	0.2428 0.2464
CAS-A	HC	RM3	ENSA	1;150599971;T;A	VAL	TP	0.22	0.2712 0.2634
CAS-A	HC	RM1	ENSA	1;150599971;T;A	VAL	TP	0.16	0.1955 0.185
CAS-A	HC	DM2	ENSA	1;150599971;T;A	VAL	TP	0.4	0.453 0.4526

CAS-A	HC	DM4	ENSA	1;150599971;T;A	VAL	TP	0.25	0.3367 0.3458
CAS-A	HC	DM4	MCM7	7;99696901;C;G	VAL	TP	0.17	0.1221 0.137
CAS-A	HC	DM3	MCM7	7;99696901;C;G	NOT_PREDICTED_NOT_OBSERVED	TN	0	0 0
CAS-A	HC	DM1	MCM7	7;99696901;C;G	NOT_PREDICTED_NOT_OBSERVED	TN	0	0 0
CAS-A	HC	RM2	MCM7	7;99696901;C;G	NOT_PREDICTED_NOT_OBSERVED	TN	0	0 0
CAS-A	HC	RM1	MCM7	7;99696901;C;G	NOT_PREDICTED_NOT_OBSERVED	TN	0	0 0
CAS-A	HC	RM3	MCM7	7;99696901;C;G	NOT_PREDICTED_NOT_OBSERVED	TN	0	0 0
CAS-A	HC	DM2	MCM7	7;99696901;C;G	NOT_PREDICTED_NOT_OBSERVED	TN	0	0 0
CAS-A	HC	RM2	PSMD8	19;38865332;C;T	VAL	TP	0.23	0.5206 0.5214
CAS-A	HC	RM3	PSMD8	19;38865332;C;T	VAL	TP	0.36	0.7007 0.728
CAS-A	HC	DM4	PSMD8	19;38865332;C;T	VAL	TP	0.4	0.7661 0.7439
CAS-A	HC	DM3	PSMD8	19;38865332;C;T	VAL	TP	0.32	0.5686 0.5895
CAS-A	HC	DM1	PSMD8	19;38865332;C;T	VAL	TP	0.3	0.5974 0.6057
CAS-A	HC	RM1	PSMD8	19;38865332;C;T	VAL	TP	0.22	0.4443 0.4459
CAS-A	HC	DM2	PSMD8	19;38865332;C;T	VAL	TP	0.41	0.8357 0.8566
CAS-A	HC	DM1	SDCCAG8	1;243471427;C;T	VAL	TP	0.24	0.2475 0.2471
CAS-A	HC	RM1	SDCCAG8	1;243471427;C;T	VAL	TP	0.31	0.2675 0.2826
CAS-A	HC	RM3	SDCCAG8	1;243471427;C;T	VAL	TP	0.45	0.3764 0.3668
CAS-A	HC	DM2	SDCCAG8	1;243471427;C;T	VAL	TP	0.47	0.4477 0.4465
CAS-A	HC	DM3	SDCCAG8	1;243471427;C;T	VAL	TP	0.42	0.4038 0.3943
CAS-A	HC	DM4	SDCCAG8	1;243471427;C;T	VAL	TP	0.6	0.6444 0.6424
CAS-A	HC	RM2	SDCCAG8	1;243471427;C;T	VAL	TP	0.28	0.3609 0.3454
CAS-B	LC	RM2	ANKLE2	12;133310637;T;C	NOT_VAL	TN	0.06	0 0
CAS-B	LC	RM1	ANKLE2	12;133310637;T;C	NOT_PREDICTED_NOT_OBSERVED	TN	0	0 0
CAS-B	LC	RM3	ANKLE2	12;133310637;T;C	NOT_PREDICTED_NOT_OBSERVED	TN	0	0 0
CAS-B	LC	DM2	ANKLE2	12;133310637;T;C	NOT_PREDICTED_NOT_OBSERVED	TN	0	0 0
CAS-B	LC	DM3	ANKLE2	12;133310637;T;C	NOT_PREDICTED_NOT_OBSERVED	TN	0	0 0
CAS-B	LC	P	ANKLE2	12;133310637;T;C	NOT_PREDICTED_NOT_OBSERVED	TN	0	0 0
CAS-B	LC	DM3	CDKN2A	9;21971163;GAGCAGCAGCAGCT;G	VAL	FN	0.49	0.3502 0.3909
CAS-B	LC	RM1	CDKN2A	9;21971163;GAGCAGCAGCAGCT;G	VAL	FN	0.46	0.3389 0.3616
CAS-B	LC	DM2	CDKN2A	9;21971163;GAGCAGCAGCAGCT;G	VAL	FN	0.26	0.1866 0.204
CAS-B	LC	RM3	CDKN2A	9;21971163;GAGCAGCAGCAGCT;G	VAL	FN	0.52	0.3412 0.3195
CAS-B	LC	P	CDKN2A	9;21971163;GAGCAGCAGCAGCT;G	VAL	FN	0.33	0.2197 0.1927
CAS-B	LC	RM2	CDKN2A	9;21971163;GAGCAGCAGCAGCT;G	VAL	FN	0.51	0.3163 0.4698
CAS-B	HC	P	KIF23	15;69733239;C;T	VAL	TP	0.32	0.4398 0.4017
CAS-B	HC	RM2	KIF23	15;69733239;C;T	VAL	TP	0.58	0.687 0.8373
CAS-B	HC	DM2	KIF23	15;69733239;C;T	VAL	TP	0.27	0.2326 0.2291
CAS-B	HC	RM3	KIF23	15;69733239;C;T	VAL	TP	0.59	0.5753 0.5741
CAS-B	HC	DM3	KIF23	15;69733239;C;T	VAL	TP	0.58	0.5147 0.556
CAS-B	HC	RM1	KIF23	15;69733239;C;T	VAL	TP	0.71	0.7118 0.7114
CAS-B	HC	RM2	TP53	17;7574003;G;A	VAL	TP	0.35	0.4476 0.9976
CAS-B	HC	DM2	TP53	17;7574003;G;A	VAL	TP	0.34	0.3623 0.3425
CAS-B	HC	DM3	TP53	17;7574003;G;A	VAL	TP	0.61	0.5989 0.6245

CAS-B	HC	RM3	TP53	17;7574003;G;A	VAL	TP	0.49	0.6014 0.6017
CAS-B	HC	RM1	TP53	17;7574003;G;A	VAL	TP	0.53	0.621 0.6155
CAS-B	HC	P	TP53	17;7574003;G;A	VAL	TP	0.37	0.4813 0.444
CAS-B	LC	RM1	TUBB4A	19;6496379;A;G	VAL	FN	0.01	0.1065 0.1075
CAS-B	LC	P	TUBB4A	19;6496379;A;G	VAL	FN	0.08	0.04739336 0.0569
CAS-B	LC	RM2	TUBB4A	19;6496379;A;G	NOT_PREDICTED_NOT_OBSERVED	TN	0	0 0
CAS-B	LC	RM3	TUBB4A	19;6496379;A;G	NOT_PREDICTED_OBSERVED	FN	0	0.1162 0.1099
CAS-B	LC	DM2	TUBB4A	19;6496379;A;G	NOT_PREDICTED_OBSERVED	FN	0	0.1654 0.1489
CAS-B	LC	DM3	TUBB4A	19;6496379;A;G	NOT_PREDICTED_OBSERVED	FN	0	0.0625 0.052
CAS-C	HC	P	CAMK2G	10;75608338;G;A	VAL	TP	0.28	0.2333 0.2457
CAS-C	HC	DM1	CAMK2G	10;75608338;G;A	NOT_PREDICTED_NOT_OBSERVED	TN	0	0 0
CAS-C	HC	DM3	CAMK2G	10;75608338;G;A	NOT_PREDICTED_NOT_OBSERVED	TN	0	0 0
CAS-C	HC	DM5	CAMK2G	10;75608338;G;A	NOT_PREDICTED_NOT_OBSERVED	TN	0	0 0
CAS-C	HC	DM2	CAMK2G	10;75608338;G;A	NOT_PREDICTED_NOT_OBSERVED	TN	0	0 0
CAS-C	HC	DM4	CAMK2G	10;75608338;G;A	NOT_PREDICTED_NOT_OBSERVED	TN	0	0 0
CAS-C	HC	DM6	CAMK2G	10;75608338;G;A	NOT_PREDICTED_NOT_OBSERVED	TN	0	0 0
CAS-C	HC	DM1	CENPE	4;104079820;T;C	VAL	TP	0.47	0.4229 0.4292
CAS-C	HC	P	CENPE	4;104079820;T;C	VAL	TP	0.26	0.3717 0.3951
CAS-C	HC	DM2	CENPE	4;104079820;T;C	VAL	TP	0.46	0.4212 0.4186
CAS-C	HC	DM6	CENPE	4;104079820;T;C	VAL	TP	0.41	0.4341
CAS-C	HC	DM5	CENPE	4;104079820;T;C	VAL	TP	0.35	0.3139 0.3142
CAS-C	HC	DM3	CENPE	4;104079820;T;C	VAL	TP	0.36	0.3371 0.3286
CAS-C	HC	DM4	CENPE	4;104079820;T;C	VAL	TP	0.33	0.3729 0.2781
CAS-C	HC	DM2	CEP290	12;88534987;GATAAGGAAATCAATAAATTATCTGCC;G	VAL	TP	0.24	0.83333336 0.92753624
CAS-C	HC	DM1	CEP290	12;88534987;GATAAGGAAATCAATAAATTATCTGCC;G	VAL	TP	0.16	0.712 0.6794
CAS-C	HC	DM4	CEP290	12;88534987;GATAAGGAAATCAATAAATTATCTGCC;G	NOT_VAL	FP	0.29	0 0
CAS-C	HC	DM6	CEP290	12;88534987;GATAAGGAAATCAATAAATTATCTGCC;G	NOT_VAL	FP	0.31	0 0
CAS-C	HC	DM5	CEP290	12;88534987;GATAAGGAAATCAATAAATTATCTGCC;G	NOT_VAL	FP	0.15	0 0
CAS-C	HC	P	CEP290	12;88534987;GATAAGGAAATCAATAAATTATCTGCC;G	VAL	TP	0.3	0.6145 0.6774
CAS-C	HC	DM3	CEP290	12;88534987;GATAAGGAAATCAATAAATTATCTGCC;G	VAL	TP	0.17	0.6153846 0.7123288
CAS-C	HC	DM6	CTNNB1	3;41266133;C;G	VAL	TP	0.38	0.6744 0.5159
CAS-C	HC	DM5	CTNNB1	3;41266133;C;G	VAL	TP	0.24	0.1752 0.3033
CAS-C	HC	DM4	CTNNB1	3;41266133;C;G	VAL	TP	0.31	0.3544 0.3071
CAS-C	HC	DM5	CTNNB1	3;41266136;T;C	VAL	TP	0.25	0.1773 0.3062
CAS-C	HC	DM6	CTNNB1	3;41266136;T;C	VAL	TP	0.39	0.6743 0.5159
CAS-C	HC	DM4	CTNNB1	3;41266136;T;C	VAL	TP	0.33	0.3568 0.3093
CAS-C	HC	DM1	CTNNB1	3;41266133;C;G	NOT_PREDICTED_NOT_OBSERVED	TN	0	0 0
CAS-C	HC	DM1	CTNNB1	3;41266136;T;C	NOT_PREDICTED_NOT_OBSERVED	TN	0	0 0
CAS-C	HC	DM3	CTNNB1	3;41266133;C;G	NOT_PREDICTED_NOT_OBSERVED	TN	0	0 0
CAS-C	HC	DM3	CTNNB1	3;41266136;T;C	NOT_PREDICTED_NOT_OBSERVED	TN	0	0 0
CAS-C	HC	DM2	CTNNB1	3;41266133;C;G	NOT_PREDICTED_NOT_OBSERVED	TN	0	0 0
CAS-C	HC	DM2	CTNNB1	3;41266136;T;C	NOT_PREDICTED_NOT_OBSERVED	TN	0	0 0
CAS-C	HC	P	CTNNB1	3;41266133;C;G	NOT_PREDICTED_NOT_OBSERVED	TN	0	0 0

CAS-C	HC	P	CTNNB1	3;41266136;T;C	NOT_PREDICTED_NOT_OBSERVED	TN	0	0 0
CAS-C	HC	DM1	E2F5	8;86114455;T;G	VAL	TP	0.23	0.3084 0.3138
CAS-C	HC	DM3	E2F5	8;86114455;T;G	NOT_PREDICTED_NOT_OBSERVED	TN	0	0 0
CAS-C	HC	DM5	E2F5	8;86114455;T;G	NOT_PREDICTED_NOT_OBSERVED	TN	0	0 0
CAS-C	HC	DM2	E2F5	8;86114455;T;G	NOT_PREDICTED_NOT_OBSERVED	TN	0	0 0
CAS-C	HC	DM4	E2F5	8;86114455;T;G	NOT_PREDICTED_NOT_OBSERVED	TN	0	0 0
CAS-C	HC	DM6	E2F5	8;86114455;T;G	NOT_PREDICTED_NOT_OBSERVED	TN	0	0 0
CAS-C	HC	P	E2F5	8;86114455;T;G	NOT_PREDICTED_NOT_OBSERVED	TN	0	0 0
CAS-C	LC	DM1	HERC2	15;28518046;G;A	NOT_VAL	TN	0.25	0 0
CAS-C	LC	DM6	HERC2	15;28518046;G;A	NOT_VAL	TN	0.54	0 0
CAS-C	LC	P	HERC2	15;28518046;G;A	NOT_VAL	TN	0.3	0 0
CAS-C	LC	DM4	HERC2	15;28518046;G;A	NOT_VAL	TN	0.5	0 0
CAS-C	LC	DM5	HERC2	15;28518046;G;A	NOT_VAL	TN	0.26	0 0
CAS-C	LC	DM2	HERC2	15;28518046;G;A	NOT_VAL	TN	0.14	0 0
CAS-C	LC	DM3	HERC2	15;28518046;G;A	NOT_PREDICTED_NOT_OBSERVED	TN	0	0 0
CAS-C	HC	P	MYO10	5;16673936;A;G	VAL	TP	0.18	0.1561 0.1577
CAS-C	HC	DM1	MYO10	5;16673936;A;G	NOT_PREDICTED_NOT_OBSERVED	TN	0	0 0
CAS-C	HC	DM3	MYO10	5;16673936;A;G	NOT_PREDICTED_NOT_OBSERVED	TN	0	0 0
CAS-C	HC	DM5	MYO10	5;16673936;A;G	NOT_PREDICTED_NOT_OBSERVED	TN	0	0 0
CAS-C	HC	DM2	MYO10	5;16673936;A;G	NOT_PREDICTED_NOT_OBSERVED	TN	0	0 0
CAS-C	HC	DM4	MYO10	5;16673936;A;G	NOT_PREDICTED_NOT_OBSERVED	TN	0	0 0
CAS-C	HC	DM6	MYO10	5;16673936;A;G	NOT_PREDICTED_NOT_OBSERVED	TN	0	0 0
CAS-C	HC	DM5	NUP205	7;135301921;C;T	VAL	TP	0.17	0.1556 0.1554
CAS-C	HC	DM1	NUP205	7;135301921;C;T	VAL	TP	0.31	0.3353 0.3302
CAS-C	HC	DM3	NUP205	7;135301921;C;T	VAL	TP	0.21	0.2139 0.222
CAS-C	HC	DM4	NUP205	7;135301921;C;T	VAL	TP	0.22	0.2002 0.2327
CAS-C	HC	P	NUP205	7;135301921;C;T	VAL	TP	0.24	0.24 0.2392
CAS-C	HC	DM2	NUP205	7;135301921;C;T	VAL	TP	0.24	0.2957 0.2749
CAS-C	HC	DM6	NUP205	7;135301921;C;T	VAL	TP	0.22	0.4191 0.2177
CAS-C	LC	DM2	RAD18	3;9005278;C;T	VAL	FN	0.35	0.3834 0.4065
CAS-C	LC	DM3	RAD18	3;9005278;C;T	VAL	FN	0.41	0.3528 0.3389
CAS-C	LC	P	RAD18	3;9005278;C;T	VAL	FN	0.8	0.3615 0.3463
CAS-C	LC	DM5	RAD18	3;9005278;C;T	VAL	FN	0.31	0.2561 0.3716
CAS-C	LC	DM1	RAD18	3;9005278;C;T	VAL	FN	0.18	0.4294 0.4422
CAS-C	LC	DM4	RAD18	3;9005278;C;T	VAL	FN	0.5	0.219 0.2209
CAS-C	LC	DM6	RAD18	3;9005278;C;T	VAL	FN	0.35	0.96428574
CAS-C	HC	DM2	UBC	12;125398287;T;C	VAL	TP	0.39	0.4079 0.3891
CAS-C	HC	P	UBC	12;125398287;T;C	VAL	TP	0.25	0.3345 0.3222
CAS-C	HC	DM1	UBC	12;125398287;T;C	VAL	TP	0.37	0.4112 0.4086
CAS-C	HC	DM6	UBC	12;125398287;T;C	VAL	TP	0.36	0.4855
CAS-C	HC	DM4	UBC	12;125398287;T;C	VAL	TP	0.28	0.3612 0.4084
CAS-C	HC	DM3	UBC	12;125398287;T;C	VAL	TP	0.31	0.3114 0.3167
CAS-C	HC	DM5	UBC	12;125398287;T;C	VAL	TP	0.23	0.3181 0.2259

CAS-C	LC	DM3	UNG	12;109541382;C;T	VAL	FN	0.46	0.3216 0.3217
CAS-C	LC	DM1	UNG	12;109541382;C;T	VAL	FN	0.39	0.4353 0.4294
CAS-C	LC	P	UNG	12;109541382;C;T	VAL	FN	0.19	0.3576 0.3465
CAS-C	LC	DM6	UNG	12;109541382;C;T	VAL	FN	0.34	0.2776 0.5611
CAS-C	LC	DM2	UNG	12;109541382;C;T	VAL	FN	0.3	0.3976 0.4123
CAS-C	LC	DM5	UNG	12;109541382;C;T	VAL	FN	0.1	0.2226 0.1628
CAS-C	LC	DM4	UNG	12;109541382;C;T	NOT_PREDICTED_NOT_OBSERVED	TN	0	0 0
CAS-C	HC	DM2	WRN	8;30998973;C;T	VAL	TP	0.42	0.4079 0.4089
CAS-C	HC	DM4	WRN	8;30998973;C;T	VAL	TP	0.31	0.3259 0.3555
CAS-C	HC	P	WRN	8;30998973;C;T	VAL	TP	0.35	0.349 0.3542
CAS-C	HC	DM6	WRN	8;30998973;C;T	VAL	TP	0.36	0.6082 0.3963
CAS-C	HC	DM1	WRN	8;30998973;C;T	VAL	TP	0.4	0.4328 0.4383
CAS-C	HC	DM3	WRN	8;30998973;C;T	VAL	TP	0.26	0.3642 0.3569
CAS-C	HC	DM5	WRN	8;30998973;C;T	VAL	TP	0.29	0.1404 0.3608
CAS-C	HC	DM5	ZW10	11;113604472;G;A	VAL	TP	0.33	0.3965 0.2715
CAS-C	HC	DM2	ZW10	11;113604472;G;A	VAL	TP	0.36	0.3971 0.3906
CAS-C	HC	DM4	ZW10	11;113604472;G;A	VAL	TP	0.39	0.353 0.2897
CAS-C	HC	DM1	ZW10	11;113604472;G;A	VAL	TP	0.4	0.4397 0.4374
CAS-C	HC	DM6	ZW10	11;113604472;G;A	VAL	TP	0.41	0.4443 0.268
CAS-C	HC	P	ZW10	11;113604472;G;A	VAL	TP	0.23	0.3499 0.3436
CAS-C	HC	DM3	ZW10	11;113604472;G;A	VAL	TP	0.26	0.3433 0.3282

Supplementary Table 4. Primers used for validation by targeted amplicon sequencing of SNVs and small InDels.

Patient	Gene	Mutation included in validation	Forward sequence	Reverse sequence
CAS-F	ENSA	1;150573651;A;G	AAATTAGCCGGGCGTGGTGT	GCTGGAGTGCAATGGCATGA
CAS-A	ENSA	1;150599971;T;A	TCGAAGAACCTCCTTTAGCTCCA	TGAGAGAGCTGAAGAGGCAAAGC
CAS-G	UBE2T	1;202302637;G;A	GAGTCAACCATTTACCCACAACTCA	CCCATTTGAACCTCCTCAGATCC
CAS-A	SDCCAG 8	1;243471427;C;T	CCGTGTTGGTGGTCTTTGTTTG	TCCGTGCGCGTAGAAGCATA
CAS-E	SMC3	10;112349666;G;A	TGTATACTGTTAATGCATCCTCATAT G	CTGCCCACCTTTCCTGTTGC
CAS-C	CAMK2G	10;75608338;G;A	GCTTACCGCAGGCCCAGATA	GGTTCAGTATGCCTCTGCCCTCT
CAS-C	ZW10	11;113604472;G;A	GGGCAGCTGCTCTTCTTTCTG	TTTGCAGGTGGGCAGATGG
CAS-A	CEP164	11;117222647;G;GA	GTGATCCAAGAGCGGGCAAA	CTGGGAGGAGGCATCCACTG
CAS-F	NFRKB	11;129743677;A;G	TGACCACGGCTTCCCTGTTT	CCCAGCCTAGCCTTCCTGCT
CAS-F	FEN1	11;61563864;T;G	CTGAGGAGCGAATCCGCAGT	CTTGGGTTCTGGCTCCTTGC
CAS-C	UNG	12;109541382;C;T	AGCGAGGCTGGGAGCAGTT	TGGCACTGCCCTTCTTCTGA
CAS-G	DYNLL1	12;120934240;G;A	CCAGGCCTTGCCCACTAGGT	TTTCTCCAGCGCCTGAGTAGC
CAS-D	UBC	12;125397821;G;A	TTTGCCTTGACATTCTCGATGG	CACCTGGTGCTCCGTCTCAG
CAS-C	UBC	12;125398287;T;C	GGGATGCCTTCCTTATCTTGGA	TCCGCTAAATTCTGGCCGTTT
CAS-D CAS-B	ANKLE2	12;133310637;T;C	CCCGACACACAACCCATTTAAC	CCACCACGCCTGGCTAATTT
CAS-G	RHNO1	12;2996279;T;C	GGTGTTGTGGCTTACGCCTGT	GAGTCAGAGTTTCACCGTGTTGG
CAS-F	SMUG1	12;54582294;C;G	CGAGACCAGCCTGACCAACA	CCCGGGTTCAAGCGATTCTT
CAS-E	CEP290	12;88483003;G;A	CATTTCTTGCATTATCTTAAGTTTGT C	TCTCTATTATGCTCGTTTGGAGGG AAG
CAS-C	CEP290	12;88534987;GATAAGGAAATCAATAAATTATCT GCC;G	ACACTTCCAGATTGTGACAATTATA GTTG	AAAGTTGACCCAGATGACCTGCC
CAS-F CAS-E	BRCA2	13;32970126;A;T	TTAGTTCCCATACAGCATATCTACTG	GCAGACACTGCAGTGATCCG
CAS-E	PDS5B	13;33233358;G;A	GCAGCAATGGCCACAATCAG	TCACCTTATGAGCAGGTACCAGA TTT
CAS-E	DYNC1H 1	14;102482274;C;T	TGCAGCTACGATCATGCAACC	CCTGGTGCAGCATGGAGAAG
CAS-D	PSMB11	14;23511991;G;A	TGGCCCTGAGCTCTTCTACG	GGCTTCCTGGGTGCTCATGT
CAS-C	HERC2	15;28518046;G;A	CCCAAAGCGTTCCCGTCTG	ACCAAAGGGCCAGGAAGCAT
CAS-G	INO80	15;41337161;G;A	ACAAAGGGCAGCTGCAAAGG	GGCTCCATCAGCTACGCTCCT
CAS-B	KIF23	15;69733239;C;T	CATGAGCCAGCCACAGCTACA	TCCTGGCTCCTGCTGCCTAC
CAS-E	NEIL1	15;75647322;A;G	TGACCATCTTGCCTGTTCTTCC	GGGTGAGTGCAAGCAAGGAGA
CAS-G	PRKCB	16;24185900;C;T	ACGCTGCAGAAATTGCCATC	CAGGGAGGAGCTGAGGGAGA
CAS-F	KIF2B	17;51900950;G;A	GCGCCCTCGATGTCAATACC	CGCAGATGCGATGTTCTTGC
CAS-D	CLTC	17;57760301;C;T	CATTTGAGTTCACAGAAAGGAAATG TT	TGCATGTACAACAATATGAAGTC CACA
CAS-F	POLR2A	17;7404163;C;T	CCCATCCCGATGATGAAGACA	GGACAAAGCACCCACTCAAGG
CAS-G	POLR2A	17;7404625;G;A	TCCATGTTTCTTAGAGGTCCAAAGG	ACGTCCTGCTTGGCCTTCTT
CAS-B	TP53	17;7574003;G;A	CACCTGGAGTGAGCCCTGCT	CCTCCTCTGTTGCTGCAGATCC
CAS-D	TP53	17;7577539;G;A	GGTGGCAAGTGGCTCCTGAC	GGTTGGCTCTGACTGTACCACCA
CAS-E	TP53	17;7578472;G;A	CGCCTCACAACCTCCGTCAT	TTGCCAACTGGCCAAGACCT
CAS-E	FZR1	19;3533325;C;T	ATAGCACCCGGCCTCGTTG	CAGGTACAGCACGCGGTAGG
CAS-A	PSMD8	19;38865332;C;T	CGGGCTGAGGCAGGTTGTAG	GCAGACCGCCTGATTTACGG
CAS-F CAS-B	TUBB4A	19;6496379;A;G	TCTGTCTCTCCACCACCTGGA	CTCGGCCTCCCAAAGTGCT

CAS-F	ANAPC1	2;112614429;G;A	TCCACTGGTGCTGCATCCTT	AATACCCATACAGGTTGTTGCATGT
CAS-G	ANAPC1	2;112625662;G;A	TGGAGTTATTTCATCTAGTGGGTGCAG	TTTGTGCACAGACATACTCGTATTGG
CAS-G	MSH6	2;48018128;G;A	CAACAGTTGTGACTTCTCACCAGGA	TTTCCCTTTCTCGCGGATGA
CAS-G	MSH6	2;48027175;G;A	CCCAGGTGCTTAAAGGTATGACTTC	ATTAGCCATTGATAAAAGCTCCTG
CAS-G	MSH6	2;48033352;C;T	TGTTGCTTTTCTGTCCTAGCATTTTTG	ATAATGTACGACATTTTATAGTCTCAGC
CAS-G	MSH6	2;48033791;G;A	TGCTAATCTCCCAGAGGAAGTTATTCA	TTTCATAGTGCATCATCCCTTCC
CAS-F	ALMS1	2;73717382;C;A	GCCAGTGTACTGGAGCATCTGTG	GGCTTTGCCTTCTACCTTCTGC
CAS-E	CSNK2A1	20;478391;G;A	TCAAGGTTCACTTACCTTCAGAATCTC	TGTCAGCATTATTTCATGGCTCAA
CAS-E	ASCC2	22;30221691;C;T	AAGGCCAGGAGCCAGTCGAG	CCCGAGCAGAAGGCAGACC
CAS-G	TOPBP1	3;133374283;G;A	CCTCCATGCTTAACTGTGAGTTGC	TTTAACCATTTGATTCCCACAGAAA
CAS-E	RNF168	3;196199162;G;A	CTCTCAAACAGTAGATGCTCCAAATCT	AACCAAGAATCTTCCTTTGAAGCAG
CAS-G	MLH1	3;37089083;G;A	CCAGCACCGCTCTTTGACCT	TGCAAGCATCTCAGCCTTCTTC
CAS-C	CTNNB1	3;41266133;C;G	GGCAGCAACAGTCTTACCTGGA	TCCCTGTTCCCACTCATACAGGA
CAS-C	CTNNB1	3;41266136;T;C	GGCAGCAACAGTCTTACCTGGA	TCCCTGTTCCCACTCATACAGGA
CAS-C	RAD18	3;9005278;C;T	GGAGCCTGCCGGAAGTTGTA	TGGTGAGGTCTGGATTTGGTTTG
CAS-C	CENPE	4;104079820;T;C	AAACCTTACCATGTTAACAGTATCGTG	ACACTGATTACTGAGAAACTGCAGC
CAS-D	TACC3	4;1729764;ACAAAGCGGAGACTCCGCACGGAGCCGAGGAAGAATG;A	CACCCGCCTCTGAGACCCTA	GTGCCGGCATTCCTCCTC
CAS-F	RNF4	4;2515439;C;T	CTGGGACAAGGGCAAACTCG	CAGGGAATCACGGAGGCACT
CAS-E	REST	4;57798316;T;C	AAAGATTACAGCAAACACCTCAATCG	AGCAATCTTAAAAGGTTGTCTGCTATC
CAS-A	APC	5;112173567;C;T	CCTGGCTCAAGCTTGCCATC	CGATGAGATGCCTTGGGACTT
CAS-C	MYO10	5;16673936;A;G	TTCCTGCCTGTGGATCAGAGC	TGCCTCCTAGGATACGGGAACA
CAS-E	MYO10	5;16676209;G;A	GGGTGGAGCTCTGGACTTACAGTT	CTGCCTGAACTCGGATGTGG
CAS-F	MYO10	5;16701934;C;A	TCACGGGTCAGTTCAGCTTCC	AAGCGGGAGCTATGCTTGGA
CAS-D	NUP155	5;37350306;G;A	CCATCTTCTGAGAACGTGAATTGTAGC	AAGGCTGAAGCAGGGTGGTTT
CAS-D	FGFR1OP	6;167417807;C;CA	GCTGCAAGGTCTCGAAGGTC	TGTTGACAGCGCCTGATCACT
CAS-F	MDC1	6;30672477;C;T	GGTCCGAGATGTGGGCTCAG	GCCTACGTCTCAGGCTACTAGGG
CAS-C	NUP205	7;135301921;C;T	CCCTGAGCCTTTGCAGTTGG	CAATCCTCACCTTGACATTGCAG
CAS-F	POM121	7;72396963;C;T	GGGTACTTCCCACCGTGTGC	TCACATCGCAGAACGCGAAA
CAS-F	POM121	7;72396964;C;T	GGGTACTTCCCACCGTGTGC	TCACATCGCAGAACGCGAAA
CAS-E	COPS6	7;99686912;G;A	GCCATCTCCAGGGAAGAACG	GCGACGGAAACACTCCCAGT
CAS-A	MCM7	7;99696901;C;G	AAATACAGCTCACTAAGGGGAGAA	GCATCGGCTAATGATGGAGCA
CAS-F	WRN	8;30974004;G;A	ACCATGCGGGCATGAGTTT	CCACTATGAGCAACGGAGAGCA
CAS-C	WRN	8;30998973;C;T	TTTCAGAATTCTCAGCGTCTTGC	CAGTGATCAGCTGACGGGAAA
CAS-C	E2F5	8;86114455;T;G	GGCTGCTGATACTTTGGCTGTG	GGTTTCAGAAGAGGGTGCTGGT
CAS-G	E2F5	8;86126052;C;A	TTTGTTCGCAGTGTTTCCTCTCTT	GAGGTAGATAACAGTCCCAAGTTTCCA
CAS-F	CDKN2A	9;21971017;G;A	GGCATGGTTACTGCCTCTGG	TCCTGGACACGCTGGTGGT
CAS-B	CDKN2A	9;21971163;GAGCAGCAGCAGCT;G	GCACGGGTCGGGTGAGAGT	CCACCCTGGCTCTGACCATT
CAS-E	CDKN2A	9;21974714;GGCAGCGCCCCCGCCTCCA;G	TCCCGCTGCAGACCCTCTAC	ATGGAGCCTTCGGCTGACTG
CAS-G	CUL4B	X;119678398;T;A	TGCAAATCAGACAGCATGCTTAAA	GAAAGTGCAGAATAAGACAATTGATGG
CAS-E	EMD	X;153609403;G;A	GAGCTCCCTGGACCTGTCCTATT	CGGGACCTGGCGATCCTG

Supplementary Table 5. Summary sequencing metrics of adjacent fresh frozen (FF) and formalin-fixed paraffin-embedded (FFPE) regions of a right lung metastasis from patient CAS-G.

CAS-G DM6 – Liver Right		
**Metric**	**FF**	**FFPE**
Amount of DNA used (ng)	500	1000
pre-capture PCR cycles	6	7
pre-capture library yield (ng)	>750	981
post-capture PCR cycles	12	10
post-capture PCR yield (ng)	327	46.2
library fragment length	419	323
Total reads	145687680	102417113
Mapped reads	145661234	102407653
% Reads mapped	99.98	99.99
% Mapped reads duplicates	5.24	5.93
Properly paired	136110826	95610291
% Reads OnTarget	69.26	83.96
% Target bases >=10-fold Coverage	99.35	97.1
% Target bases >=20-fold Coverage	98.36	93.81
% Target bases >=100-fold Coverage	66.05	54.73
Mean coverage for target bases	156.93	137.43
% Target bases with one fifth of mean coverage	96.13	90.67

Supplementary Table 6. Recurring SNVs and small InDels.

Patient	Sample	Variant	MAF	Mutation Type	Gene	COSMIC	Polyphen2	SIFT	AA Change	Time Variant Acquisition
CAS-G	DM5	1;27105932;G;A	0.22	missense variant	ARID1A	.	possibly damaging (0.775)	.	ENSP00000320485.7:p.Gly1848Glu	LATE
CAS-G	DM6	6;157406040;G;A	0.09	splice donor variant	ARID1B	.	.	.	.	LATE
CAS-G	DM1	X;76854915;C;T	0.44	missense variant	ATRX	.	benign (0.027)	tolerated (1)	ENSP00000362441.4:p.Gly1974Glu	EARLY
CAS-G	DM2	X;76854915;C;T	0.41	missense variant	ATRX	.	benign (0.027)	tolerated (1)	ENSP00000362441.4:p.Gly1974Glu	EARLY
CAS-G	DM3	X;76854915;C;T	0.49	missense variant	ATRX	.	benign (0.027)	tolerated (1)	ENSP00000362441.4:p.Gly1974Glu	EARLY
CAS-G	DM4	X;76854915;C;T	0.40	missense variant	ATRX	.	benign (0.027)	tolerated (1)	ENSP00000362441.4:p.Gly1974Glu	EARLY
CAS-G	DM5	X;76854915;C;T	0.42	missense variant	ATRX	.	benign (0.027)	tolerated (1)	ENSP00000362441.4:p.Gly1974Glu	EARLY
CAS-G	DM6	X;76854915;C;T	0.44	missense variant	ATRX	.	benign (0.027)	tolerated (1)	ENSP00000362441.4:p.Gly1974Glu	EARLY
CAS-G	P	X;76854915;C;T	0.38	missense variant	ATRX	.	benign (0.027)	tolerated (1)	ENSP00000362441.4:p.Gly1974Glu	EARLY
CAS-G	RM	X;76854915;C;T	0.48	missense variant	ATRX	.	benign (0.027)	tolerated (1)	ENSP00000362441.4:p.Gly1974Glu	EARLY
CAS-G	DM1	7;140453136;A;T	0.67	missense variant	BRAF	476	possibly damaging (0.792)	deleterious (0)	ENSP00000288602.6:p.Val600Glu	EARLY
CAS-G	DM2	7;140453136;A;T	0.59	missense variant	BRAF	476	possibly damaging (0.792)	deleterious (0)	ENSP00000288602.6:p.Val600Glu	EARLY
CAS-G	DM3	7;140453136;A;T	0.67	missense variant	BRAF	476	possibly damaging (0.792)	deleterious (0)	ENSP00000288602.6:p.Val600Glu	EARLY
CAS-G	DM4	7;140453136;A;T	0.67	missense variant	BRAF	476	possibly damaging (0.792)	deleterious (0)	ENSP00000288602.6:p.Val600Glu	EARLY
CAS-G	DM5	7;140453136;A;T	0.60	missense variant	BRAF	476	possibly damaging (0.792)	deleterious (0)	ENSP00000288602.6:p.Val600Glu	EARLY
CAS-G	DM6	7;140453136;A;T	0.63	missense variant	BRAF	476	possibly damaging (0.792)	deleterious (0)	ENSP00000288602.6:p.Val600Glu	EARLY
CAS-G	P	7;140453136;A;T	0.50	missense variant	BRAF	476	possibly damaging (0.792)	deleterious (0)	ENSP00000288602.6:p.Val600Glu	EARLY
CAS-G	RM	7;140453136;A;T	0.50	missense variant	BRAF	476	possibly damaging (0.792)	deleterious (0)	ENSP00000288602.6:p.Val600Glu	EARLY
CAS-G	DM1	7;2977589;C;T	0.55	missense variant	CARD11	222114	benign (0.01)	tolerated (0.41)	ENSP00000380150.4:p.Met365Ile	EARLY
CAS-G	DM2	7;2977589;C;T	0.58	missense variant	CARD11	222114	benign (0.01)	tolerated (0.41)	ENSP00000380150.4:p.Met365Ile	EARLY
CAS-G	DM3	7;2977589;C;T	0.66	missense variant	CARD11	222114	benign (0.01)	tolerated (0.41)	ENSP00000380150.4:p.Met365Ile	EARLY
CAS-G	DM4	7;2977589;C;T	0.57	missense variant	CARD11	222114	benign (0.01)	tolerated (0.41)	ENSP00000380150.4:p.Met365Ile	EARLY
CAS-G	DM5	7;2977589;C;T	0.62	missense variant	CARD11	222114	benign (0.01)	tolerated (0.41)	ENSP00000380150.4:p.Met365Ile	EARLY
CAS-G	DM6	7;2969682;C;T	0.07	missense variant	CARD11	.	benign (0.359)	deleterious (0)	ENSP00000347695.3:p.Asp5Asn	LATE
CAS-G	DM6	7;2977589;C;T	0.67	missense variant	CARD11	222114	benign (0.01)	tolerated (0.41)	ENSP00000380150.4:p.Met365Ile	EARLY
CAS-G	P	7;2977589;C;T	0.50	missense variant	CARD11	222114	benign (0.01)	tolerated (0.41)	ENSP00000380150.4:p.Met365Ile	EARLY
CAS-G	RM	7;2977589;C;T	0.50	missense variant	CARD11	222114	benign (0.01)	tolerated (0.41)	ENSP00000380150.4:p.Met365Ile	EARLY
CAS-G	DM6	22;19223266;G;A	0.16	missense variant	CLTCL1	.	benign (0.14)	tolerated (0.61)	ENSP00000439662.1:p.Pro308Ser	LATE
CAS-G	DM4	12;48374703;G;A	0.07	missense variant	COL2A1	.	unknown (0)	tolerated (0.09)	ENSP00000338213.6:p.Pro769Ser	LATE
CAS-G	DM4	14;95574322;G;A	0.11	missense variant	DICER1	.	benign (0.015)	tolerated (1)	ENSP00000376783.1:p.Leu849Phe	LATE
CAS-G	DM5	19;10940840;C;T	0.11	missense variant	DNM2	.	probably damaging (0.968)	tolerated (0.16)	ENSP00000386192.3:p.Pro773Ser	LATE
CAS-G	DM6	7;55231517;G;A	0.07	splice donor variant	EGFR	.	.	.	.	LATE
CAS-G	DM1	4;126336276;C;T	0.49	missense variant	FAT4	.	possibly damaging (0.747)	deleterious (0.04)	ENSP00000335169.5:p.Ser351Phe	EARLY
CAS-G	DM2	4;126336276;C;T	0.41	missense variant	FAT4	.	possibly damaging (0.747)	deleterious (0.04)	ENSP00000335169.5:p.Ser351Phe	EARLY
CAS-G	DM3	4;126336276;C;T	0.50	missense variant	FAT4	.	possibly damaging (0.747)	deleterious (0.04)	ENSP00000335169.5:p.Ser351Phe	EARLY

CAS-G	DM4	4;126336276;C;T	0.44	missense variant	FAT4	.	possibly damaging (0.747)	deleterious (0.04)	ENSP00000335169.5:p.Ser351Phe	EARLY
CAS-G	DM5	4;126336276;C;T	0.50	missense variant	FAT4	.	possibly damaging (0.747)	deleterious (0.04)	ENSP00000335169.5:p.Ser351Phe	EARLY
CAS-G	DM6	4;126336276;C;T	0.49	missense variant	FAT4	.	possibly damaging (0.747)	deleterious (0.04)	ENSP00000335169.5:p.Ser351Phe	EARLY
CAS-G	P	4;126336276;C;T	0.50	missense variant	FAT4	.	possibly damaging (0.747)	deleterious (0.04)	ENSP00000335169.5:p.Ser351Phe	EARLY
CAS-G	RM	4;126336276;C;T	0.50	missense variant	FAT4	.	possibly damaging (0.747)	deleterious (0.04)	ENSP00000335169.5:p.Ser351Phe	EARLY
CAS-G	DM6	5;180056265;C;T	0.29	missense variant	FLT4	.	probably damaging (0.954)	deleterious (0)	ENSP00000377016.3:p.Val327Met	LATE
CAS-G	DM1	16;9858331;C;T	0.32	missense variant	GRIN2A	.	benign (0.003)	tolerated (0.79)	ENSP00000454998.1:p.Asp1024Asn	EARLY
CAS-G	DM2	16;9858331;C;T	0.25	missense variant	GRIN2A	.	benign (0.003)	tolerated (0.79)	ENSP00000454998.1:p.Asp1024Asn	EARLY
CAS-G	DM3	16;9858331;C;T	0.49	missense variant	GRIN2A	.	benign (0.003)	tolerated (0.79)	ENSP00000454998.1:p.Asp1024Asn	EARLY
CAS-G	DM4	16;9858331;C;T	0.33	missense variant	GRIN2A	.	benign (0.003)	tolerated (0.79)	ENSP00000454998.1:p.Asp1024Asn	EARLY
CAS-G	DM5	16;9858331;C;T	0.27	missense variant	GRIN2A	.	benign (0.003)	tolerated (0.79)	ENSP00000454998.1:p.Asp1024Asn	EARLY
CAS-G	DM6	16;9858331;C;T	0.38	missense variant	GRIN2A	.	benign (0.003)	tolerated (0.79)	ENSP00000454998.1:p.Asp1024Asn	EARLY
CAS-G	P	16;9858331;C;T	0.40	missense variant	GRIN2A	.	benign (0.003)	tolerated (0.79)	ENSP00000454998.1:p.Asp1024Asn	EARLY
CAS-G	RM	16;9858331;C;T	0.48	missense variant	GRIN2A	.	benign (0.003)	tolerated (0.79)	ENSP00000454998.1:p.Asp1024Asn	EARLY
CAS-G	DM1	7;50468131;G;A	0.35	missense variant	IKZF1	.	probably damaging (1)	deleterious (0)	ENSP00000413025.1:p.Gly414Arg	EARLY
CAS-G	DM2	7;50468131;G;A	0.35	missense variant	IKZF1	.	probably damaging (1)	deleterious (0)	ENSP00000413025.1:p.Gly414Arg	EARLY
CAS-G	DM3	7;50468131;G;A	0.35	missense variant	IKZF1	.	probably damaging (1)	deleterious (0)	ENSP00000413025.1:p.Gly414Arg	EARLY
CAS-G	DM4	7;50468131;G;A	0.30	missense variant	IKZF1	.	probably damaging (1)	deleterious (0)	ENSP00000413025.1:p.Gly414Arg	EARLY
CAS-G	DM5	7;50468131;G;A	0.30	missense variant	IKZF1	.	probably damaging (1)	deleterious (0)	ENSP00000413025.1:p.Gly414Arg	EARLY
CAS-G	DM6	7;50468131;G;A	0.30	missense variant	IKZF1	.	probably damaging (1)	deleterious (0)	ENSP00000413025.1:p.Gly414Arg	EARLY
CAS-G	P	7;50468131;G;A	0.44	missense variant	IKZF1	.	probably damaging (1)	deleterious (0)	ENSP00000413025.1:p.Gly414Arg	EARLY
CAS-G	RM	7;50468131;G;A	0.46	missense variant	IKZF1	.	probably damaging (1)	deleterious (0)	ENSP00000413025.1:p.Gly414Arg	EARLY
CAS-G	DM4	4;55953837;G;A	0.03	missense variant	KDR	.	probably damaging (0.984)	deleterious (0.03)	ENSP00000263923.4:p.Ser1200Phe	LATE
CAS-G	DM6	4;55953837;G;A	0.08	missense variant	KDR	.	probably damaging (0.984)	deleterious (0.03)	ENSP00000263923.4:p.Ser1200Phe	LATE
CAS-G	DM4	11;118373167;C;T	0.09	missense variant	KMT2A	.	probably damaging (0.996)	deleterious (0)	ENSP00000436786.1:p.Ser2187Phe	LATE
CAS-G	DM6	11;118365066;C;T	0.09	missense variant	KMT2A	.	probably damaging (0.998)	deleterious (0.04)	ENSP00000436786.1:p.Pro1748Ser	LATE
CAS-G	DM6	7;151845643;C;T	0.09	missense variant	KMT2C	.	probably damaging (1)	deleterious (0.01)	ENSP00000262189.6:p.Gly4457Ser	LATE
CAS-G	DM6	12;49422948;C;T	0.09	missense variant	KMT2D	.	unknown (0)	.	ENSP00000301067.7:p.Gly4716Glu	LATE
CAS-G	DM6	12;49428241;G;A	0.26	missense variant	KMT2D	.	benign (0.158)	.	ENSP00000301067.7:p.Pro3487Ser	LATE
CAS-G	DM6	12;49444216;G;A	0.33	missense variant	KMT2D	.	possibly damaging (0.747)	.	ENSP00000301067.7:p.Pro1052Leu	LATE
CAS-G	RM	22;21345976;G;A	0.19	missense variant	LZTR1	.	probably damaging (0.998)	deleterious (0)	ENSP00000374006.3:p.Arg265His	LATE
CAS-G	DM6	16;15931998;C;T	0.04	missense variant	MYH11	.	benign (0.004)	tolerated (0.2)	ENSP00000379616.3:p.Val38Ile	LATE
CAS-G	DM6	17;15938135;C;T	0.08	missense variant	NCOR1	.	probably damaging (1)	deleterious (0)	ENSP00000268712.2:p.Gly2360Glu	LATE
CAS-G	DM5	12;124832806;G;A	0.06	missense variant	NCOR2	.	benign (0.23)	tolerated (0.08)	ENSP00000380513.1:p.Pro1291Leu	LATE
CAS-G	DM4	5;176721420;G;A	0.04	missense variant	NSD1	.	possibly damaging (0.628)	deleterious (0.01)	ENSP00000343209.4:p.Glu2082Lys	LATE
CAS-G	DM6	11;71723981;C;T	0.06	missense variant	NUMA1	.	probably damaging (0.992)	tolerated (1)	ENSP00000446396.1:p.Arg127Lys	LATE
CAS-G	DM1	16;23646286;A;C	0.67	missense variant	PALB2	.	benign (0.005)	tolerated (0.13)	ENSP00000261584.4:p.Cys527Trp	EARLY
CAS-G	DM2	16;23646286;A;C	0.52	missense variant	PALB2	.	benign (0.005)	tolerated (0.13)	ENSP00000261584.4:p.Cys527Trp	EARLY
CAS-G	DM3	16;23646286;A;C	0.51	missense variant	PALB2	.	benign (0.005)	tolerated (0.13)	ENSP00000261584.4:p.Cys527Trp	EARLY

CAS-G	DM4	16;23646286;A;C	0.66	missense variant	PALB2	.	benign (0.005)	tolerated (0.13)	ENSP00000261584.4:p.Cys527Trp	EARLY
CAS-G	DM5	16;23646286;A;C	0.50	missense variant	PALB2	.	benign (0.005)	tolerated (0.13)	ENSP00000261584.4:p.Cys527Trp	EARLY
CAS-G	DM6	16;23646286;A;C	0.51	missense variant	PALB2	.	benign (0.005)	tolerated (0.13)	ENSP00000261584.4:p.Cys527Trp	EARLY
CAS-G	P	16;23646286;A;C	0.31	missense variant	PALB2	.	benign (0.005)	tolerated (0.13)	ENSP00000261584.4:p.Cys527Trp	EARLY
CAS-G	RM	16;23646286;A;C	0.42	missense variant	PALB2	.	benign (0.005)	tolerated (0.13)	ENSP00000261584.4:p.Cys527Trp	EARLY
CAS-G	DM4	3;178936091;G;A	0.08	missense variant	PIK3CA	763,125370	probably damaging (0.959)	deleterious (0.01)	ENSP00000263967.3:p.Glu545Lys	LATE
CAS-G	DM6	3;178936082;G;A	0.21	missense variant	PIK3CA	760,125369	probably damaging (0.96)	tolerated (0.11)	ENSP00000263967.3:p.Glu542Lys	LATE
CAS-G	DM5	6;117629979;G;A	0.25	missense variant	ROS1	.	probably damaging (1)	deleterious (0)	ENSP00000357494.3:p.Pro2183Ser	LATE
CAS-G	DM3	3;47084115;G;A	0.06	missense variant	SETD2	.	probably damaging (0.939)	deleterious (0)	ENSP00000386759.3:p.Pro2392Ser	LATE
CAS-G	DM1	12;47163174;C;T	0.49	missense variant	SLC38A4	.	benign (0.097)	tolerated (0.09)	ENSP00000389843.1:p.Arg446Gln	EARLY
CAS-G	DM2	12;47163174;C;T	0.29	missense variant	SLC38A4	.	benign (0.097)	tolerated (0.09)	ENSP00000389843.1:p.Arg446Gln	EARLY
CAS-G	DM3	12;47163174;C;T	0.28	missense variant	SLC38A4	.	benign (0.097)	tolerated (0.09)	ENSP00000389843.1:p.Arg446Gln	EARLY
CAS-G	DM4	12;47163174;C;T	0.21	missense variant	SLC38A4	.	benign (0.097)	tolerated (0.09)	ENSP00000389843.1:p.Arg446Gln	EARLY
CAS-G	DM5	12;47163174;C;T	0.30	missense variant	SLC38A4	.	benign (0.097)	tolerated (0.09)	ENSP00000389843.1:p.Arg446Gln	EARLY
CAS-G	DM6	12;47163174;C;T	0.25	missense variant	SLC38A4	.	benign (0.097)	tolerated (0.09)	ENSP00000389843.1:p.Arg446Gln	EARLY
CAS-G	P	12;47163174;C;T	0.48	missense variant	SLC38A4	.	benign (0.097)	tolerated (0.09)	ENSP00000389843.1:p.Arg446Gln	EARLY
CAS-G	RM	12;47163174;C;T	0.50	missense variant	SLC38A4	.	benign (0.097)	tolerated (0.09)	ENSP00000389843.1:p.Arg446Gln	EARLY
CAS-G	DM1	6;84302943;G;A	0.38	missense variant	SNAP91	.	benign (0.161)	tolerated (0.46)	ENSP00000428215.1:p.Pro578Ser	EARLY
CAS-G	DM2	6;84302943;G;A	0.33	missense variant	SNAP91	.	benign (0.161)	tolerated (0.46)	ENSP00000428215.1:p.Pro578Ser	EARLY
CAS-G	DM3	6;84302943;G;A	0.41	missense variant	SNAP91	.	benign (0.161)	tolerated (0.46)	ENSP00000428215.1:p.Pro578Ser	EARLY
CAS-G	DM4	6;84302943;G;A	0.38	missense variant	SNAP91	.	benign (0.161)	tolerated (0.46)	ENSP00000428215.1:p.Pro578Ser	EARLY
CAS-G	DM5	6;84302943;G;A	0.33	missense variant	SNAP91	.	benign (0.161)	tolerated (0.46)	ENSP00000428215.1:p.Pro578Ser	EARLY
CAS-G	DM6	6;84302943;G;A	0.23	missense variant	SNAP91	.	benign (0.161)	tolerated (0.46)	ENSP00000428215.1:p.Pro578Ser	EARLY
CAS-G	P	6;84302943;G;A	0.44	missense variant	SNAP91	.	benign (0.161)	tolerated (0.46)	ENSP00000428215.1:p.Pro578Ser	EARLY
CAS-G	RM	6;84302943;G;A	0.45	missense variant	SNAP91	.	benign (0.161)	tolerated (0.46)	ENSP00000428215.1:p.Pro578Ser	EARLY
CAS-G	DM4	16;11348713;G;A	0.04	missense variant	SOCS1	.	probably damaging (1)	deleterious (0)	ENSP00000329418.2:p.Pro208Leu	LATE
CAS-G	DM1	2;214354699;C;T	0.61	missense variant	SPAG16	222751	possibly damaging (0.746)	deleterious (0.02)	ENSP00000332592.5:p.Pro319Ser	EARLY
CAS-G	DM2	2;214354699;C;T	0.47	missense variant	SPAG16	222751	possibly damaging (0.746)	deleterious (0.02)	ENSP00000332592.5:p.Pro319Ser	EARLY
CAS-G	DM3	2;214354699;C;T	0.43	missense variant	SPAG16	222751	possibly damaging (0.746)	deleterious (0.02)	ENSP00000332592.5:p.Pro319Ser	EARLY
CAS-G	DM4	2;214354699;C;T	0.53	missense variant	SPAG16	222751	possibly damaging (0.746)	deleterious (0.02)	ENSP00000332592.5:p.Pro319Ser	EARLY
CAS-G	DM5	2;214354699;C;T	0.47	missense variant	SPAG16	222751	possibly damaging (0.746)	deleterious (0.02)	ENSP00000332592.5:p.Pro319Ser	EARLY
CAS-G	DM6	2;214354699;C;T	0.50	missense variant	SPAG16	222751	possibly damaging (0.746)	deleterious (0.02)	ENSP00000332592.5:p.Pro319Ser	EARLY
CAS-G	P	2;214354699;C;T	0.39	missense variant	SPAG16	222751	possibly damaging (0.746)	deleterious (0.02)	ENSP00000332592.5:p.Pro319Ser	EARLY
CAS-G	RM	2;214354699;C;T	0.50	missense variant	SPAG16	222751	possibly damaging (0.746)	deleterious (0.02)	ENSP00000332592.5:p.Pro319Ser	EARLY
CAS-G	DM4	4;106157647;C;T	0.08	missense variant	TET2	.	benign (0.011)	tolerated (0.06)	ENSP00000265149.5:p.His850Tyr	LATE
CAS-G	DM6	17;7579550;G;A	0.10	missense variant	TP53	45013	benign (0.266)	tolerated (0.69)	ENSP00000398846.2:p.Ser46Phe	LATE
CAS-G	DM4	1;186291703;G;A	0.03	stop gained	TPR	.	.	.	ENSP00000356448.3:p.Arg2105Ter	LATE
CAS-G	DM4	16;2121565;C;T	0.05	missense variant	TSC2	.	benign (0.396)	tolerated (0.13)	ENSP00000384468.2:p.Pro632Ser	LATE
CAS-G	DM5	8;103307981;C;T	0.05	missense variant	UBR5	.	probably damaging (0.996)	deleterious (0)	ENSP00000427819.1:p.Cys1226Tyr	LATE

CAS-G	DM1	17;5048874;G;A	0.49	missense variant	USP6	.	probably damaging (0.991)	deleterious (0.01)	ENSP00000328010.4:p.Val723Met	EARLY
CAS-G	DM2	17;5048874;G;A	0.57	missense variant	USP6	.	probably damaging (0.991)	deleterious (0.01)	ENSP00000328010.4:p.Val723Met	EARLY
CAS-G	DM3	17;5048874;G;A	0.49	missense variant	USP6	.	probably damaging (0.991)	deleterious (0.01)	ENSP00000328010.4:p.Val723Met	EARLY
CAS-G	DM4	17;5048874;G;A	0.47	missense variant	USP6	.	probably damaging (0.991)	deleterious (0.01)	ENSP00000328010.4:p.Val723Met	EARLY
CAS-G	DM5	17;5048874;G;A	0.67	missense variant	USP6	.	probably damaging (0.991)	deleterious (0.01)	ENSP00000328010.4:p.Val723Met	EARLY
CAS-G	DM6	17;5048874;G;A	0.50	missense variant	USP6	.	probably damaging (0.991)	deleterious (0.01)	ENSP00000328010.4:p.Val723Met	EARLY
CAS-G	P	17;5048874;G;A	0.47	missense variant	USP6	.	probably damaging (0.991)	deleterious (0.01)	ENSP00000328010.4:p.Val723Met	EARLY
CAS-G	RM	17;5048874;G;A	0.50	missense variant	USP6	.	probably damaging (0.991)	deleterious (0.01)	ENSP00000328010.4:p.Val723Met	EARLY
CAS-D	DM1	20;30959968;T;C	0.17	missense variant	ASXL1	.	benign (0.302)	deleterious (0.01)	ENSP00000364841.1:p.Val81Ala	LATE
CAS-D	DM1	7;140453136;A;T	0.58	missense variant	BRAF	476	possibly damaging (0.792)	deleterious (0)	ENSP00000288602.6:p.Val600Glu	EARLY
CAS-D	DM2	7;140453136;A;T	0.50	missense variant	BRAF	476	possibly damaging (0.792)	deleterious (0)	ENSP00000288602.6:p.Val600Glu	EARLY
CAS-D	DM3	7;140453136;A;T	0.47	missense variant	BRAF	476	possibly damaging (0.792)	deleterious (0)	ENSP00000288602.6:p.Val600Glu	EARLY
CAS-D	DM4	7;140453136;A;T	0.50	missense variant	BRAF	476	possibly damaging (0.792)	deleterious (0)	ENSP00000288602.6:p.Val600Glu	EARLY
CAS-D	DM5	7;140453136;A;T	0.50	missense variant	BRAF	476	possibly damaging (0.792)	deleterious (0)	ENSP00000288602.6:p.Val600Glu	EARLY
CAS-D	DM6	7;140453136;A;T	0.49	missense variant	BRAF	476	possibly damaging (0.792)	deleterious (0)	ENSP00000288602.6:p.Val600Glu	EARLY
CAS-D	P1	7;140453136;A;T	0.45	missense variant	BRAF	476	possibly damaging (0.792)	deleterious (0)	ENSP00000288602.6:p.Val600Glu	EARLY
CAS-D	P2	7;140453136;A;T	0.44	missense variant	BRAF	476	possibly damaging (0.792)	deleterious (0)	ENSP00000288602.6:p.Val600Glu	EARLY
CAS-D	P3	7;140453136;A;T	0.50	missense variant	BRAF	476	possibly damaging (0.792)	deleterious (0)	ENSP00000288602.6:p.Val600Glu	EARLY
CAS-D	RM	7;140453136;A;T	0.50	missense variant	BRAF	476	possibly damaging (0.792)	deleterious (0)	ENSP00000288602.6:p.Val600Glu	EARLY
CAS-D	DM1	7;2985579;C;T	0.24	missense variant	CARD11	.	probably damaging (1)	deleterious (0)	ENSP00000380150.4:p.Asp78Asn	EARLY
CAS-D	DM2	7;2985579;C;T	0.40	missense variant	CARD11	.	probably damaging (1)	deleterious (0)	ENSP00000380150.4:p.Asp78Asn	EARLY
CAS-D	DM3	7;2985579;C;T	0.43	missense variant	CARD11	.	probably damaging (1)	deleterious (0)	ENSP00000380150.4:p.Asp78Asn	EARLY
CAS-D	DM4	7;2985579;C;T	0.42	missense variant	CARD11	.	probably damaging (1)	deleterious (0)	ENSP00000380150.4:p.Asp78Asn	EARLY
CAS-D	DM5	7;2985579;C;T	0.52	missense variant	CARD11	.	probably damaging (1)	deleterious (0)	ENSP00000380150.4:p.Asp78Asn	EARLY
CAS-D	DM6	7;2985579;C;T	0.53	missense variant	CARD11	.	probably damaging (1)	deleterious (0)	ENSP00000380150.4:p.Asp78Asn	EARLY
CAS-D	P1	7;2985579;C;T	0.43	missense variant	CARD11	.	probably damaging (1)	deleterious (0)	ENSP00000380150.4:p.Asp78Asn	EARLY
CAS-D	P2	7;2985579;C;T	0.50	missense variant	CARD11	.	probably damaging (1)	deleterious (0)	ENSP00000380150.4:p.Asp78Asn	EARLY
CAS-D	P3	7;2985579;C;T	0.66	missense variant	CARD11	.	probably damaging (1)	deleterious (0)	ENSP00000380150.4:p.Asp78Asn	EARLY
CAS-D	RM	7;2985579;C;T	0.41	missense variant	CARD11	.	probably damaging (1)	deleterious (0)	ENSP00000380150.4:p.Asp78Asn	EARLY
CAS-D	DM1	4;187541876;G;A	0.63	missense variant	FAT1	.	probably damaging (0.966)	tolerated (0.14)	ENSP00000406229.2:p.Ser1955Phe	EARLY
CAS-D	DM2	4;187541876;G;A	0.67	missense variant	FAT1	.	probably damaging (0.966)	tolerated (0.14)	ENSP00000406229.2:p.Ser1955Phe	EARLY
CAS-D	DM3	4;187541876;G;A	0.31	missense variant	FAT1	.	probably damaging (0.966)	tolerated (0.14)	ENSP00000406229.2:p.Ser1955Phe	EARLY
CAS-D	DM4	4;187541876;G;A	0.50	missense variant	FAT1	.	probably damaging (0.966)	tolerated (0.14)	ENSP00000406229.2:p.Ser1955Phe	EARLY
CAS-D	DM5	4;187541876;G;A	0.31	missense variant	FAT1	.	probably damaging (0.966)	tolerated (0.14)	ENSP00000406229.2:p.Ser1955Phe	EARLY
CAS-D	DM6	4;187541876;G;A	0.31	missense variant	FAT1	.	probably damaging (0.966)	tolerated (0.14)	ENSP00000406229.2:p.Ser1955Phe	EARLY
CAS-D	P1	4;187541876;G;A	0.41	missense variant	FAT1	.	probably damaging (0.966)	tolerated (0.14)	ENSP00000406229.2:p.Ser1955Phe	EARLY

CAS-D	P2	4;187541876;G;A	0.30	missense variant	FAT1	.	probably damaging (0.966)	tolerated (0.14)	ENSP00000406229.2:p.Ser1955Phe	EARLY
CAS-D	P3	4;187541876;G;A	0.50	missense variant	FAT1	.	probably damaging (0.966)	tolerated (0.14)	ENSP00000406229.2:p.Ser1955Phe	EARLY
CAS-D	RM	4;187541876;G;A	0.67	missense variant	FAT1	.	probably damaging (0.966)	tolerated (0.14)	ENSP00000406229.2:p.Ser1955Phe	EARLY
CAS-D	DM1	16;9858586;C;T	0.40	missense variant	GRIN2A	.	probably damaging (1)	tolerated (0.67)	ENSP00000454998.1:p.Gly939Arg	EARLY
CAS-D	DM2	16;9858586;C;T	0.50	missense variant	GRIN2A	.	probably damaging (1)	tolerated (0.67)	ENSP00000454998.1:p.Gly939Arg	EARLY
CAS-D	DM3	16;9858586;C;T	0.35	missense variant	GRIN2A	.	probably damaging (1)	tolerated (0.67)	ENSP00000454998.1:p.Gly939Arg	EARLY
CAS-D	DM4	16;9858586;C;T	0.49	missense variant	GRIN2A	.	probably damaging (1)	tolerated (0.67)	ENSP00000454998.1:p.Gly939Arg	EARLY
CAS-D	DM5	16;9858586;C;T	0.37	missense variant	GRIN2A	.	probably damaging (1)	tolerated (0.67)	ENSP00000454998.1:p.Gly939Arg	EARLY
CAS-D	DM6	16;9858586;C;T	0.33	missense variant	GRIN2A	.	probably damaging (1)	tolerated (0.67)	ENSP00000454998.1:p.Gly939Arg	EARLY
CAS-D	P1	16;9858586;C;T	0.33	missense variant	GRIN2A	.	probably damaging (1)	tolerated (0.67)	ENSP00000454998.1:p.Gly939Arg	EARLY
CAS-D	P2	16;9858586;C;T	0.50	missense variant	GRIN2A	.	probably damaging (1)	tolerated (0.67)	ENSP00000454998.1:p.Gly939Arg	EARLY
CAS-D	P3	16;9858586;C;T	0.43	missense variant	GRIN2A	.	probably damaging (1)	tolerated (0.67)	ENSP00000454998.1:p.Gly939Arg	EARLY
CAS-D	RM	16;9858586;C;T	0.39	missense variant	GRIN2A	.	probably damaging (1)	tolerated (0.67)	ENSP00000454998.1:p.Gly939Arg	EARLY
CAS-D	DM1	7;50467675;G;A	0.67	missense variant	IKZF1	.	possibly damaging (0.738)	deleterious (0)	ENSP00000413025.1:p.Glu262Lys	EARLY
CAS-D	DM2	7;50467675;G;A	0.35	missense variant	IKZF1	.	possibly damaging (0.738)	deleterious (0)	ENSP00000413025.1:p.Glu262Lys	EARLY
CAS-D	DM3	7;50467675;G;A	0.39	missense variant	IKZF1	.	possibly damaging (0.738)	deleterious (0)	ENSP00000413025.1:p.Glu262Lys	EARLY
CAS-D	DM4	7;50467675;G;A	0.50	missense variant	IKZF1	.	possibly damaging (0.738)	deleterious (0)	ENSP00000413025.1:p.Glu262Lys	EARLY
CAS-D	DM5	7;50467675;G;A	0.37	missense variant	IKZF1	.	possibly damaging (0.738)	deleterious (0)	ENSP00000413025.1:p.Glu262Lys	EARLY
CAS-D	DM6	7;50467675;G;A	0.38	missense variant	IKZF1	.	possibly damaging (0.738)	deleterious (0)	ENSP00000413025.1:p.Glu262Lys	EARLY
CAS-D	P1	7;50467675;G;A	0.41	missense variant	IKZF1	.	possibly damaging (0.738)	deleterious (0)	ENSP00000413025.1:p.Glu262Lys	EARLY
CAS-D	P2	7;50467675;G;A	0.50	missense variant	IKZF1	.	possibly damaging (0.738)	deleterious (0)	ENSP00000413025.1:p.Glu262Lys	EARLY
CAS-D	P3	7;50467675;G;A	0.44	missense variant	IKZF1	.	possibly damaging (0.738)	deleterious (0)	ENSP00000413025.1:p.Glu262Lys	EARLY
CAS-D	RM	7;50467675;G;A	0.45	missense variant	IKZF1	.	possibly damaging (0.738)	deleterious (0)	ENSP00000413025.1:p.Glu262Lys	EARLY
CAS-D	DM1	12;47162166;A;T	0.19	missense variant	SLC38A4	.	probably damaging (0.998)	deleterious (0)	ENSP00000389843.1:p.Ile490Asn	EARLY
CAS-D	DM2	12;47162166;A;T	0.47	missense variant	SLC38A4	.	probably damaging (0.998)	deleterious (0)	ENSP00000389843.1:p.Ile490Asn	EARLY
CAS-D	DM3	12;47162166;A;T	0.50	missense variant	SLC38A4	.	probably damaging (0.998)	deleterious (0)	ENSP00000389843.1:p.Ile490Asn	EARLY
CAS-D	DM4	12;47162166;A;T	0.66	missense variant	SLC38A4	.	probably damaging (0.998)	deleterious (0)	ENSP00000389843.1:p.Ile490Asn	EARLY
CAS-D	DM5	12;47162166;A;T	0.61	missense variant	SLC38A4	.	probably damaging (0.998)	deleterious (0)	ENSP00000389843.1:p.Ile490Asn	EARLY
CAS-D	DM6	12;47162166;A;T	0.67	missense variant	SLC38A4	.	probably damaging (0.998)	deleterious (0)	ENSP00000389843.1:p.Ile490Asn	EARLY
CAS-D	P1	12;47162166;A;T	0.42	missense variant	SLC38A4	.	probably damaging (0.998)	deleterious (0)	ENSP00000389843.1:p.Ile490Asn	EARLY
CAS-D	P2	12;47162166;A;T	0.67	missense variant	SLC38A4	.	probably damaging (0.998)	deleterious (0)	ENSP00000389843.1:p.Ile490Asn	EARLY
CAS-D	P3	12;47162166;A;T	0.50	missense variant	SLC38A4	.	probably damaging (0.998)	deleterious (0)	ENSP00000389843.1:p.Ile490Asn	EARLY
CAS-D	RM	12;47162166;A;T	0.45	missense variant	SLC38A4	.	probably damaging (0.998)	deleterious (0)	ENSP00000389843.1:p.Ile490Asn	EARLY
CAS-D	DM1	17;7577539;G;A	1.00	missense variant	TP53	10656,120007,120005, 1640831,120006	probably damaging (1)	deleterious (0)	ENSP00000398846.2:p.Arg248Trp	EARLY
CAS-D	DM2	17;7577539;G;A	0.54	missense variant	TP53	10656,120007,120005, 1640831,120006	probably damaging (1)	deleterious (0)	ENSP00000398846.2:p.Arg248Trp	EARLY
CAS-D	DM3	17;7577539;G;A	0.89	missense variant	TP53	10656,120007,120005, 1640831,120006	probably damaging (1)	deleterious (0)	ENSP00000398846.2:p.Arg248Trp	EARLY

CAS-D	DM4	17;7577539;G;A	0.91	missense variant	TP53	10656,120007,120005, 1640831,120006	probably damaging (1)	deleterious (0)	ENSP00000398846.2:p.Arg248Trp	EARLY
CAS-D	DM5	17;7577539;G;A	1.00	missense variant	TP53	10656,120007,120005, 1640831,120006	probably damaging (1)	deleterious (0)	ENSP00000398846.2:p.Arg248Trp	EARLY
CAS-D	DM6	17;7577539;G;A	0.79	missense variant	TP53	10656,120007,120005, 1640831,120006	probably damaging (1)	deleterious (0)	ENSP00000398846.2:p.Arg248Trp	EARLY
CAS-D	P1	17;7577539;G;A	0.64	missense variant	TP53	10656,120007,120005, 1640831,120006	probably damaging (1)	deleterious (0)	ENSP00000398846.2:p.Arg248Trp	EARLY
CAS-D	P2	17;7577539;G;A	1.00	missense variant	TP53	10656,120007,120005, 1640831,120006	probably damaging (1)	deleterious (0)	ENSP00000398846.2:p.Arg248Trp	EARLY
CAS-D	P3	17;7577539;G;A	0.54	missense variant	TP53	10656,120007,120005, 1640831,120006	probably damaging (1)	deleterious (0)	ENSP00000398846.2:p.Arg248Trp	EARLY
CAS-D	RM	17;7577539;G;A	0.91	missense variant	TP53	10656,120007,120005, 1640831,120006	probably damaging (1)	deleterious (0)	ENSP00000398846.2:p.Arg248Trp	EARLY
CAS-F	DM1	1;27056295;C;T	0.68	missense variant	ARID1A	.	benign (0.053)	.	ENSP00000320485.7:p.Pro431Ser	EARLY
CAS-F	DM2	1;27056295;C;T	0.72	missense variant	ARID1A	.	benign (0.053)	.	ENSP00000320485.7:p.Pro431Ser	EARLY
CAS-F	DM3	1;27056295;C;T	0.60	missense variant	ARID1A	.	benign (0.053)	.	ENSP00000320485.7:p.Pro431Ser	EARLY
CAS-F	RM1	1;27056295;C;T	0.75	missense variant	ARID1A	.	benign (0.053)	.	ENSP00000320485.7:p.Pro431Ser	EARLY
CAS-F	RM2	1;27056295;C;T	0.65	missense variant	ARID1A	.	benign (0.053)	.	ENSP00000320485.7:p.Pro431Ser	EARLY
CAS-F	RM3	1;27056295;C;T	0.57	missense variant	ARID1A	.	benign (0.053)	.	ENSP00000320485.7:p.Pro431Ser	EARLY
CAS-F	DM1	12;46246137;C;T	0.30	stop gained	ARID2	.	.	.	ENSP00000388357.1:p.Gln19Ter	EARLY
CAS-F	DM1	12;46285631;C;T	0.50	missense variant	ARID2	.	possibly damaging (0.595)	tolerated (0.31)	ENSP00000388357.1:p.Pro272Leu	EARLY
CAS-F	DM2	12;46246137;C;T	0.39	stop gained	ARID2	.	.	.	ENSP00000388357.1:p.Gln19Ter	EARLY
CAS-F	DM2	12;46285631;C;T	0.50	missense variant	ARID2	.	possibly damaging (0.595)	tolerated (0.31)	ENSP00000388357.1:p.Pro272Leu	EARLY
CAS-F	DM3	12;46246137;C;T	0.40	stop gained	ARID2	.	.	.	ENSP00000388357.1:p.Gln19Ter	EARLY
CAS-F	DM3	12;46285631;C;T	0.61	missense variant	ARID2	.	possibly damaging (0.595)	tolerated (0.31)	ENSP00000388357.1:p.Pro272Leu	EARLY
CAS-F	RM1	12;46246137;C;T	0.50	stop gained	ARID2	.	.	.	ENSP00000388357.1:p.Gln19Ter	EARLY
CAS-F	RM1	12;46285631;C;T	0.50	missense variant	ARID2	.	possibly damaging (0.595)	tolerated (0.31)	ENSP00000388357.1:p.Pro272Leu	EARLY
CAS-F	RM2	12;46246137;C;T	0.46	stop gained	ARID2	.	.	.	ENSP00000388357.1:p.Gln19Ter	EARLY
CAS-F	RM2	12;46285631;C;T	0.50	missense variant	ARID2	.	possibly damaging (0.595)	tolerated (0.31)	ENSP00000388357.1:p.Pro272Leu	EARLY
CAS-F	RM3	12;46285631;C;T	0.39	missense variant	ARID2	.	possibly damaging (0.595)	tolerated (0.31)	ENSP00000388357.1:p.Pro272Leu	EARLY
CAS-F	RM3	12;46246137;C;T	0.50	stop gained	ARID2	.	.	.	ENSP00000388357.1:p.Gln19Ter	EARLY
CAS-F	DM1	7;140453136;A;T	0.71	missense variant	BRAF	476	possibly damaging (0.792)	deleterious (0)	ENSP00000288602.6:p.Val600Glu	EARLY
CAS-F	DM2	7;140453136;A;T	0.80	missense variant	BRAF	476	possibly damaging (0.792)	deleterious (0)	ENSP00000288602.6:p.Val600Glu	EARLY
CAS-F	DM3	7;140453136;A;T	0.69	missense variant	BRAF	476	possibly damaging (0.792)	deleterious (0)	ENSP00000288602.6:p.Val600Glu	EARLY
CAS-F	RM1	7;140453136;A;T	0.72	missense variant	BRAF	476	possibly damaging (0.792)	deleterious (0)	ENSP00000288602.6:p.Val600Glu	EARLY
CAS-F	RM2	7;140453136;A;T	0.75	missense variant	BRAF	476	possibly damaging (0.792)	deleterious (0)	ENSP00000288602.6:p.Val600Glu	EARLY
CAS-F	RM3	7;140453136;A;T	0.61	missense variant	BRAF	476	possibly damaging (0.792)	deleterious (0)	ENSP00000288602.6:p.Val600Glu	EARLY
CAS-F	DM1	22;19184164;G;A	0.42	missense variant	CLTCL1	.	possibly damaging (0.789)	deleterious (0.04)	ENSP00000439662.1:p.Arg1293Cys	EARLY
CAS-F	DM2	22;19184164;G;A	0.49	missense variant	CLTCL1	.	possibly damaging (0.789)	deleterious (0.04)	ENSP00000439662.1:p.Arg1293Cys	EARLY
CAS-F	DM3	22;19184164;G;A	0.42	missense variant	CLTCL1	.	possibly damaging (0.789)	deleterious (0.04)	ENSP00000439662.1:p.Arg1293Cys	EARLY
CAS-F	RM1	22;19184164;G;A	0.56	missense variant	CLTCL1	.	possibly damaging (0.789)	deleterious (0.04)	ENSP00000439662.1:p.Arg1293Cys	EARLY
CAS-F	RM2	22;19184164;G;A	0.65	missense variant	CLTCL1	.	possibly damaging (0.789)	deleterious (0.04)	ENSP00000439662.1:p.Arg1293Cys	EARLY
CAS-F	RM3	22;19184164;G;A	0.32	missense variant	CLTCL1	.	possibly damaging (0.789)	deleterious (0.04)	ENSP00000439662.1:p.Arg1293Cys	EARLY
CAS-F	DM1	12;48378791;G;A	0.41	missense variant	COL2A1	.	probably damaging (0.995)	deleterious (0.03)	ENSP00000338213.6:p.Pro538Leu	EARLY

CAS-F	DM2	12;48378791;G;A	0.38	missense variant	COL2A1	.	probably damaging (0.995)	deleterious (0.03)	ENSP00000338213.6:p.Pro538Leu	EARLY
CAS-F	DM3	12;48378791;G;A	0.26	missense variant	COL2A1	.	probably damaging (0.995)	deleterious (0.03)	ENSP00000338213.6:p.Pro538Leu	EARLY
CAS-F	RM1	12;48378791;G;A	0.42	missense variant	COL2A1	.	probably damaging (0.995)	deleterious (0.03)	ENSP00000338213.6:p.Pro538Leu	EARLY
CAS-F	RM2	12;48378791;G;A	0.50	missense variant	COL2A1	.	probably damaging (0.995)	deleterious (0.03)	ENSP00000338213.6:p.Pro538Leu	EARLY
CAS-F	RM3	12;48378791;G;A	0.49	missense variant	COL2A1	.	probably damaging (0.995)	deleterious (0.03)	ENSP00000338213.6:p.Pro538Leu	EARLY
CAS-F	DM1	7;55272970;C;T	0.77	missense variant	EGFR	.	benign (0.14)	tolerated (0.07)	ENSP00000275493.2:p.Pro1098Leu	EARLY
CAS-F	DM2	7;55272970;C;T	0.70	missense variant	EGFR	.	benign (0.14)	tolerated (0.07)	ENSP00000275493.2:p.Pro1098Leu	EARLY
CAS-F	DM3	7;55272970;C;T	1.00	missense variant	EGFR	.	benign (0.14)	tolerated (0.07)	ENSP00000275493.2:p.Pro1098Leu	EARLY
CAS-F	RM1	7;55272970;C;T	1.00	missense variant	EGFR	.	benign (0.14)	tolerated (0.07)	ENSP00000275493.2:p.Pro1098Leu	EARLY
CAS-F	RM2	7;55272970;C;T	0.88	missense variant	EGFR	.	benign (0.14)	tolerated (0.07)	ENSP00000275493.2:p.Pro1098Leu	EARLY
CAS-F	RM3	7;55272970;C;T	1.00	missense variant	EGFR	.	benign (0.14)	tolerated (0.07)	ENSP00000275493.2:p.Pro1098Leu	EARLY
CAS-F	DM1	4;187517744;G;A	0.21	missense variant	FAT1	.	benign (0.354)	tolerated (0.32)	ENSP00000406229.2:p.Pro4317Leu	EARLY
CAS-F	DM2	4;187517744;G;A	0.39	missense variant	FAT1	.	benign (0.354)	tolerated (0.32)	ENSP00000406229.2:p.Pro4317Leu	EARLY
CAS-F	DM3	4;187517744;G;A	0.50	missense variant	FAT1	.	benign (0.354)	tolerated (0.32)	ENSP00000406229.2:p.Pro4317Leu	EARLY
CAS-F	RM1	4;187517744;G;A	0.19	missense variant	FAT1	.	benign (0.354)	tolerated (0.32)	ENSP00000406229.2:p.Pro4317Leu	EARLY
CAS-F	RM2	4;187517744;G;A	0.40	missense variant	FAT1	.	benign (0.354)	tolerated (0.32)	ENSP00000406229.2:p.Pro4317Leu	EARLY
CAS-F	RM3	4;187517744;G;A	0.41	missense variant	FAT1	.	benign (0.354)	tolerated (0.32)	ENSP00000406229.2:p.Pro4317Leu	EARLY
CAS-F	DM1	4;126398317;G;A	0.67	missense variant	FAT4	.	probably damaging (0.999)	tolerated (1)	ENSP00000335169.5:p.Asp2364Asn	EARLY
CAS-F	DM1	4;126372684;G;A	0.53	missense variant	FAT4	.	probably damaging (1)	deleterious (0)	ENSP00000335169.5:p.Asp1803Asn	EARLY
CAS-F	DM1	4;126239992;G;A	0.19	missense variant	FAT4	.	possibly damaging (0.904)	tolerated (0.17)	ENSP00000377862.3:p.Gly809Glu	EARLY
CAS-F	DM2	4;126398317;G;A	0.56	missense variant	FAT4	.	probably damaging (0.999)	tolerated (1)	ENSP00000335169.5:p.Asp2364Asn	EARLY
CAS-F	DM2	4;126239992;G;A	0.19	missense variant	FAT4	.	possibly damaging (0.904)	tolerated (0.17)	ENSP00000377862.3:p.Gly809Glu	EARLY
CAS-F	DM2	4;126372684;G;A	0.63	missense variant	FAT4	.	probably damaging (1)	deleterious (0)	ENSP00000335169.5:p.Asp1803Asn	EARLY
CAS-F	DM3	4;126239992;G;A	0.43	missense variant	FAT4	.	possibly damaging (0.904)	tolerated (0.17)	ENSP00000377862.3:p.Gly809Glu	EARLY
CAS-F	DM3	4;126372684;G;A	0.49	missense variant	FAT4	.	probably damaging (1)	deleterious (0)	ENSP00000335169.5:p.Asp1803Asn	EARLY
CAS-F	DM3	4;126398317;G;A	0.38	missense variant	FAT4	.	probably damaging (0.999)	tolerated (1)	ENSP00000335169.5:p.Asp2364Asn	EARLY
CAS-F	RM1	4;126398317;G;A	0.67	missense variant	FAT4	.	probably damaging (0.999)	tolerated (1)	ENSP00000335169.5:p.Asp2364Asn	EARLY
CAS-F	RM1	4;126372684;G;A	0.67	missense variant	FAT4	.	probably damaging (1)	deleterious (0)	ENSP00000335169.5:p.Asp1803Asn	EARLY
CAS-F	RM1	4;126239992;G;A	0.41	missense variant	FAT4	.	possibly damaging (0.904)	tolerated (0.17)	ENSP00000377862.3:p.Gly809Glu	EARLY
CAS-F	RM2	4;126372684;G;A	0.67	missense variant	FAT4	.	probably damaging (1)	deleterious (0)	ENSP00000335169.5:p.Asp1803Asn	EARLY
CAS-F	RM2	4;126239992;G;A	0.49	missense variant	FAT4	.	possibly damaging (0.904)	tolerated (0.17)	ENSP00000377862.3:p.Gly809Glu	EARLY
CAS-F	RM2	4;126398317;G;A	0.67	missense variant	FAT4	.	probably damaging (0.999)	tolerated (1)	ENSP00000335169.5:p.Asp2364Asn	EARLY
CAS-F	RM3	4;126239992;G;A	0.39	missense variant	FAT4	.	possibly damaging (0.904)	tolerated (0.17)	ENSP00000377862.3:p.Gly809Glu	EARLY
CAS-F	RM3	4;126398317;G;A	0.30	missense variant	FAT4	.	probably damaging (0.999)	tolerated (1)	ENSP00000335169.5:p.Asp2364Asn	EARLY
CAS-F	RM3	4;126372684;G;A	0.48	missense variant	FAT4	.	probably damaging (1)	deleterious (0)	ENSP00000335169.5:p.Asp1803Asn	EARLY
CAS-F	DM1	7;151970859;C;T	0.08	missense variant	KMT2C	1179669,1179668	probably damaging (1)	deleterious (0)	ENSP00000453752.1:p.Gly315Ser	EARLY
CAS-F	DM2	7;151945171;G;A	0.08	missense variant	KMT2C	.	possibly damaging (0.729)	deleterious (0)	ENSP00000453752.1:p.Ser783Phe	EARLY
CAS-F	DM2	7;151970859;C;T	0.06	missense variant	KMT2C	1179669,1179668	probably damaging (1)	deleterious (0)	ENSP00000453752.1:p.Gly315Ser	EARLY

CAS-F	DM3	7;151970859;C;T	0.07	missense variant	KMT2C	1179669,1179668	probably damaging (1)	deleterious (0)	ENSP00000453752.1:p.Gly315Ser	EARLY
CAS-F	RM1	7;151970859;C;T	0.07	missense variant	KMT2C	1179669,1179668	probably damaging (1)	deleterious (0)	ENSP00000453752.1:p.Gly315Ser	EARLY
CAS-F	RM1	7;151927025;A;G	0.14	missense variant	KMT2C	253767,253768	probably damaging (0.999)	deleterious (0)	ENSP00000262189.6:p.Tyr987His	EARLY
CAS-F	RM1	7;151945171;G;A	0.02	missense variant	KMT2C	.	possibly damaging (0.729)	deleterious (0)	ENSP00000453752.1:p.Ser783Phe	EARLY
CAS-F	RM2	7;151945171;G;A	0.10	missense variant	KMT2C	.	possibly damaging (0.729)	deleterious (0)	ENSP00000453752.1:p.Ser783Phe	EARLY
CAS-F	RM2	7;151927025;A;G	0.07	missense variant	KMT2C	253767,253768	probably damaging (0.999)	deleterious (0)	ENSP00000262189.6:p.Tyr987His	EARLY
CAS-F	RM2	7;151970859;C;T	0.10	missense variant	KMT2C	1179669,1179668	probably damaging (1)	deleterious (0)	ENSP00000453752.1:p.Gly315Ser	EARLY
CAS-F	RM3	7;151927025;A;G	0.06	missense variant	KMT2C	253767,253768	probably damaging (0.999)	deleterious (0)	ENSP00000262189.6:p.Tyr987His	EARLY
CAS-F	RM3	7;151945171;G;A	0.04	missense variant	KMT2C	.	possibly damaging (0.729)	deleterious (0)	ENSP00000453752.1:p.Ser783Phe	EARLY
CAS-F	RM3	7;151970859;C;T	0.02	missense variant	KMT2C	1179669,1179668	probably damaging (1)	deleterious (0)	ENSP00000453752.1:p.Gly315Ser	EARLY
CAS-F	DM1	16;15841910;C;T	0.50	missense variant	MYH11	.	probably damaging (0.999)	deleterious (0.02)	ENSP00000379616.3:p.Arg732His	EARLY
CAS-F	DM2	16;15841910;C;T	0.59	missense variant	MYH11	.	probably damaging (0.999)	deleterious (0.02)	ENSP00000379616.3:p.Arg732His	EARLY
CAS-F	DM3	16;15841910;C;T	0.67	missense variant	MYH11	.	probably damaging (0.999)	deleterious (0.02)	ENSP00000379616.3:p.Arg732His	EARLY
CAS-F	RM1	16;15841910;C;T	0.50	missense variant	MYH11	.	probably damaging (0.999)	deleterious (0.02)	ENSP00000379616.3:p.Arg732His	EARLY
CAS-F	RM2	16;15841910;C;T	0.33	missense variant	MYH11	.	probably damaging (0.999)	deleterious (0.02)	ENSP00000379616.3:p.Arg732His	EARLY
CAS-F	RM3	16;15841910;C;T	0.59	missense variant	MYH11	.	probably damaging (0.999)	deleterious (0.02)	ENSP00000379616.3:p.Arg732His	EARLY
CAS-F	DM3	9;127915915;C;T	0.59	missense variant	PPP6C	.	probably damaging (1)	deleterious (0)	ENSP00000392147.1:p.Gly226Glu	LATE
CAS-F	DM1	6;106534435;G;A	0.55	missense variant	PRDM1	.	benign (0.179)	tolerated (0.21)	ENSP00000358092.4:p.Asp3Asn	EARLY
CAS-F	DM2	6;106534435;G;A	0.67	missense variant	PRDM1	.	benign (0.179)	tolerated (0.21)	ENSP00000358092.4:p.Asp3Asn	EARLY
CAS-F	DM3	6;106534435;G;A	0.33	missense variant	PRDM1	.	benign (0.179)	tolerated (0.21)	ENSP00000358092.4:p.Asp3Asn	EARLY
CAS-F	RM1	6;106534435;G;A	0.67	missense variant	PRDM1	.	benign (0.179)	tolerated (0.21)	ENSP00000358092.4:p.Asp3Asn	EARLY
CAS-F	RM2	6;106534435;G;A	0.64	missense variant	PRDM1	.	benign (0.179)	tolerated (0.21)	ENSP00000358092.4:p.Asp3Asn	EARLY
CAS-F	RM3	6;106534435;G;A	0.67	missense variant	PRDM1	.	benign (0.179)	tolerated (0.21)	ENSP00000358092.4:p.Asp3Asn	EARLY
CAS-F	DM1	6;117714445;C;T	0.19	missense variant	ROS1	1697839,1697840	benign (0.169)	tolerated (0.24)	ENSP00000357494.3:p.Glu402Lys	EARLY
CAS-F	DM2	6;117714445;C;T	0.39	missense variant	ROS1	1697839,1697840	benign (0.169)	tolerated (0.24)	ENSP00000357494.3:p.Glu402Lys	EARLY
CAS-F	DM3	6;117714445;C;T	0.56	missense variant	ROS1	1697839,1697840	benign (0.169)	tolerated (0.24)	ENSP00000357494.3:p.Glu402Lys	EARLY
CAS-F	RM1	6;117714445;C;T	0.43	missense variant	ROS1	1697839,1697840	benign (0.169)	tolerated (0.24)	ENSP00000357494.3:p.Glu402Lys	EARLY
CAS-F	RM2	6;117714445;C;T	0.45	missense variant	ROS1	1697839,1697840	benign (0.169)	tolerated (0.24)	ENSP00000357494.3:p.Glu402Lys	EARLY
CAS-F	RM3	6;117714445;C;T	0.59	missense variant	ROS1	1697839,1697840	benign (0.169)	tolerated (0.24)	ENSP00000357494.3:p.Glu402Lys	EARLY
CAS-F	DM1	6;84270618;G;A	0.16	missense variant	SNAP91	.	possibly damaging (0.604)	deleterious (0.04)	ENSP00000428215.1:p.Pro831Ser	EARLY
CAS-F	DM2	6;84270618;G;A	0.24	missense variant	SNAP91	.	possibly damaging (0.604)	deleterious (0.04)	ENSP00000428215.1:p.Pro831Ser	EARLY
CAS-F	DM3	6;84270618;G;A	0.67	missense variant	SNAP91	.	possibly damaging (0.604)	deleterious (0.04)	ENSP00000428215.1:p.Pro831Ser	EARLY
CAS-F	RM1	6;84270618;G;A	0.40	missense variant	SNAP91	.	possibly damaging (0.604)	deleterious (0.04)	ENSP00000428215.1:p.Pro831Ser	EARLY
CAS-F	RM2	6;84270618;G;A	0.41	missense variant	SNAP91	.	possibly damaging (0.604)	deleterious (0.04)	ENSP00000428215.1:p.Pro831Ser	EARLY
CAS-F	RM3	6;84270618;G;A	0.27	missense variant	SNAP91	.	possibly damaging (0.604)	deleterious (0.04)	ENSP00000428215.1:p.Pro831Ser	EARLY
CAS-F	DM1	8;103291368;G;A	0.50	stop gained	UBR5	1472022	.	.	ENSP00000427819.1:p.Arg2018Ter	EARLY
CAS-F	DM2	8;103291368;G;A	0.35	stop gained	UBR5	1472022	.	.	ENSP00000427819.1:p.Arg2018Ter	EARLY
CAS-F	DM3	8;103291368;G;A	0.50	stop gained	UBR5	1472022	.	.	ENSP00000427819.1:p.Arg2018Ter	EARLY

CAS-F	RM1	8;103291368;G;A	0.56	stop gained	UBR5	1472022	.	.	ENSP00000427819.1:p.Arg2018Ter	EARLY
CAS-F	RM2	8;103291368;G;A	0.47	stop gained	UBR5	1472022	.	.	ENSP00000427819.1:p.Arg2018Ter	EARLY
CAS-F	RM3	8;103291368;G;A	0.67	stop gained	UBR5	1472022	.	.	ENSP00000427819.1:p.Arg2018Ter	EARLY
CAS-F	DM1	8;30974004;G;A	0.29	missense variant	WRN	.	probably damaging (0.999)	deleterious (0)	ENSP00000298139.5:p.Arg803Lys	EARLY
CAS-F	DM3	8;30974004;G;A	0.50	missense variant	WRN	.	probably damaging (0.999)	deleterious (0)	ENSP00000298139.5:p.Arg803Lys	EARLY
CAS-F	RM1	8;30974004;G;A	0.14	missense variant	WRN	.	probably damaging (0.999)	deleterious (0)	ENSP00000298139.5:p.Arg803Lys	EARLY
CAS-F	RM2	8;30974004;G;A	0.50	missense variant	WRN	.	probably damaging (0.999)	deleterious (0)	ENSP00000298139.5:p.Arg803Lys	EARLY
CAS-F	RM3	8;30974004;G;A	0.43	missense variant	WRN	.	probably damaging (0.999)	deleterious (0)	ENSP00000298139.5:p.Arg803Lys	EARLY
CAS-E	P	20;30959968;T;C	0.24	missense variant	ASXL1	.	benign (0.302)	deleterious (0.01)	ENSP00000364841.1:p.Val81Ala	EARLY
CAS-E	DM1	7;140453136;A;T	0.50	missense variant	BRAF	476	possibly damaging (0.792)	deleterious (0)	ENSP00000288602.6:p.Val600Glu	EARLY
CAS-E	DM2	7;140453136;A;T	0.36	missense variant	BRAF	476	possibly damaging (0.792)	deleterious (0)	ENSP00000288602.6:p.Val600Glu	EARLY
CAS-E	DM3	7;140453136;A;T	0.34	missense variant	BRAF	476	possibly damaging (0.792)	deleterious (0)	ENSP00000288602.6:p.Val600Glu	EARLY
CAS-E	DM4	7;140453136;A;T	0.44	missense variant	BRAF	476	possibly damaging (0.792)	deleterious (0)	ENSP00000288602.6:p.Val600Glu	EARLY
CAS-E	DM5	7;140453136;A;T	0.44	missense variant	BRAF	476	possibly damaging (0.792)	deleterious (0)	ENSP00000288602.6:p.Val600Glu	EARLY
CAS-E	P	7;140453136;A;T	0.41	missense variant	BRAF	476	possibly damaging (0.792)	deleterious (0)	ENSP00000288602.6:p.Val600Glu	EARLY
CAS-E	RM	7;140453136;A;T	0.50	missense variant	BRAF	476	possibly damaging (0.792)	deleterious (0)	ENSP00000288602.6:p.Val600Glu	EARLY
CAS-E	DM1	12;48376890;G;A	0.65	missense variant	COL2A1	1705526,1705527	unknown (0)	tolerated (0.1)	ENSP00000338213.6:p.Leu625Phe	EARLY
CAS-E	DM2	12;48376890;G;A	0.58	missense variant	COL2A1	1705526,1705527	unknown (0)	tolerated (0.1)	ENSP00000338213.6:p.Leu625Phe	EARLY
CAS-E	DM3	12;48376890;G;A	0.63	missense variant	COL2A1	1705526,1705527	unknown (0)	tolerated (0.1)	ENSP00000338213.6:p.Leu625Phe	EARLY
CAS-E	DM4	12;48376890;G;A	0.75	missense variant	COL2A1	1705526,1705527	unknown (0)	tolerated (0.1)	ENSP00000338213.6:p.Leu625Phe	EARLY
CAS-E	DM5	12;48376890;G;A	0.67	missense variant	COL2A1	1705526,1705527	unknown (0)	tolerated (0.1)	ENSP00000338213.6:p.Leu625Phe	EARLY
CAS-E	P	12;48376890;G;A	0.67	missense variant	COL2A1	1705526,1705527	unknown (0)	tolerated (0.1)	ENSP00000338213.6:p.Leu625Phe	EARLY
CAS-E	RM	12;48376890;G;A	0.67	missense variant	COL2A1	1705526,1705527	unknown (0)	tolerated (0.1)	ENSP00000338213.6:p.Leu625Phe	EARLY
CAS-E	DM1	19;10935754;C;T	0.35	stop gained	DNM2	.	.	.	ENSP00000373905.3:p.Gln639Ter	EARLY
CAS-E	DM2	19;10935754;C;T	0.32	stop gained	DNM2	.	.	.	ENSP00000373905.3:p.Gln639Ter	EARLY
CAS-E	DM3	19;10935754;C;T	0.45	stop gained	DNM2	.	.	.	ENSP00000373905.3:p.Gln639Ter	EARLY
CAS-E	DM4	19;10935754;C;T	0.87	stop gained	DNM2	.	.	.	ENSP00000373905.3:p.Gln639Ter	EARLY
CAS-E	DM5	19;10935754;C;T	0.50	stop gained	DNM2	.	.	.	ENSP00000373905.3:p.Gln639Ter	EARLY
CAS-E	P	19;10935754;C;T	0.39	stop gained	DNM2	.	.	.	ENSP00000373905.3:p.Gln639Ter	EARLY
CAS-E	RM	19;10935754;C;T	0.45	stop gained	DNM2	.	.	.	ENSP00000373905.3:p.Gln639Ter	EARLY
CAS-E	DM1	7;55223543;C;T	0.50	missense variant	EGFR	.	benign (0.103)	tolerated (1)	ENSP00000413843.2:p.His304Tyr	EARLY
CAS-E	DM2	7;55223543;C;T	0.41	missense variant	EGFR	.	benign (0.103)	tolerated (1)	ENSP00000413843.2:p.His304Tyr	EARLY
CAS-E	DM3	7;55223543;C;T	0.39	missense variant	EGFR	.	benign (0.103)	tolerated (1)	ENSP00000413843.2:p.His304Tyr	EARLY
CAS-E	DM4	7;55223543;C;T	0.41	missense variant	EGFR	.	benign (0.103)	tolerated (1)	ENSP00000413843.2:p.His304Tyr	EARLY
CAS-E	DM5	7;55223543;C;T	0.39	missense variant	EGFR	.	benign (0.103)	tolerated (1)	ENSP00000413843.2:p.His304Tyr	EARLY
CAS-E	P	7;55223543;C;T	0.43	missense variant	EGFR	.	benign (0.103)	tolerated (1)	ENSP00000413843.2:p.His304Tyr	EARLY
CAS-E	RM	7;55223543;C;T	0.50	missense variant	EGFR	.	benign (0.103)	tolerated (1)	ENSP00000413843.2:p.His304Tyr	EARLY
CAS-E	DM1	4;126241630;C;T	0.47	missense variant	FAT4	.	benign (0.297)	tolerated (0.19)	ENSP00000377862.3:p.Thr1355Ile	EARLY
CAS-E	DM2	4;126241630;C;T	0.33	missense variant	FAT4	.	benign (0.297)	tolerated (0.19)	ENSP00000377862.3:p.Thr1355Ile	EARLY
CAS-E	DM3	4;126241630;C;T	0.30	missense variant	FAT4	.	benign (0.297)	tolerated (0.19)	ENSP00000377862.3:p.Thr1355Ile	EARLY

CAS-E	DM4	4;126241630;C;T	0.42	missense variant	FAT4	.	benign (0.297)	tolerated (0.19)	ENSP00000377862.3:p.Thr1355Ile	EARLY
CAS-E	DM5	4;126241630;C;T	0.34	missense variant	FAT4	.	benign (0.297)	tolerated (0.19)	ENSP00000377862.3:p.Thr1355Ile	EARLY
CAS-E	P	4;126241630;C;T	0.50	missense variant	FAT4	.	benign (0.297)	tolerated (0.19)	ENSP00000377862.3:p.Thr1355Ile	EARLY
CAS-E	RM	4;126241630;C;T	0.41	missense variant	FAT4	.	benign (0.297)	tolerated (0.19)	ENSP00000377862.3:p.Thr1355Ile	EARLY
CAS-E	DM1	5;180052998;G;A	0.35	missense variant	FLT4	.	possibly damaging (0.866)	deleterious (0.04)	ENSP00000377016.3:p.Ser431Phe	EARLY
CAS-E	DM2	5;180052998;G;A	0.39	missense variant	FLT4	.	possibly damaging (0.866)	deleterious (0.04)	ENSP00000377016.3:p.Ser431Phe	EARLY
CAS-E	DM3	5;180052998;G;A	0.21	missense variant	FLT4	.	possibly damaging (0.866)	deleterious (0.04)	ENSP00000377016.3:p.Ser431Phe	EARLY
CAS-E	DM4	5;180052998;G;A	0.67	missense variant	FLT4	.	possibly damaging (0.866)	deleterious (0.04)	ENSP00000377016.3:p.Ser431Phe	EARLY
CAS-E	DM5	5;180052998;G;A	0.30	missense variant	FLT4	.	possibly damaging (0.866)	deleterious (0.04)	ENSP00000377016.3:p.Ser431Phe	EARLY
CAS-E	P	5;180052998;G;A	0.35	missense variant	FLT4	.	possibly damaging (0.866)	deleterious (0.04)	ENSP00000377016.3:p.Ser431Phe	EARLY
CAS-E	RM	5;180052998;G;A	0.17	missense variant	FLT4	.	possibly damaging (0.866)	deleterious (0.04)	ENSP00000377016.3:p.Ser431Phe	EARLY
CAS-E	DM1	16;9857436;C;T	0.25	missense variant	GRIN2A	.	benign (0.005)	tolerated (0.52)	ENSP00000379820.2:p.Gly1322Glu	EARLY
CAS-E	DM3	16;9857436;C;T	0.32	missense variant	GRIN2A	.	benign (0.005)	tolerated (0.52)	ENSP00000379820.2:p.Gly1322Glu	EARLY
CAS-E	DM4	16;9857436;C;T	0.43	missense variant	GRIN2A	.	benign (0.005)	tolerated (0.52)	ENSP00000379820.2:p.Gly1322Glu	EARLY
CAS-E	DM5	16;9857436;C;T	0.40	missense variant	GRIN2A	.	benign (0.005)	tolerated (0.52)	ENSP00000379820.2:p.Gly1322Glu	EARLY
CAS-E	P	16;9857436;C;T	0.36	missense variant	GRIN2A	.	benign (0.005)	tolerated (0.52)	ENSP00000379820.2:p.Gly1322Glu	EARLY
CAS-E	RM	16;9857436;C;T	0.41	missense variant	GRIN2A	.	benign (0.005)	tolerated (0.52)	ENSP00000379820.2:p.Gly1322Glu	EARLY
CAS-E	DM1	12;49445638;G;A	0.66	missense variant	KMT2D	.	unknown (0)	.	ENSP00000301067.7:p.Pro610Ser	EARLY
CAS-E	DM2	12;49445638;G;A	0.67	missense variant	KMT2D	.	unknown (0)	.	ENSP00000301067.7:p.Pro610Ser	EARLY
CAS-E	DM3	12;49445638;G;A	0.63	missense variant	KMT2D	.	unknown (0)	.	ENSP00000301067.7:p.Pro610Ser	EARLY
CAS-E	DM4	12;49445638;G;A	0.69	missense variant	KMT2D	.	unknown (0)	.	ENSP00000301067.7:p.Pro610Ser	EARLY
CAS-E	DM5	12;49445638;G;A	0.67	missense variant	KMT2D	.	unknown (0)	.	ENSP00000301067.7:p.Pro610Ser	EARLY
CAS-E	P	12;49445638;G;A	0.67	missense variant	KMT2D	.	unknown (0)	.	ENSP00000301067.7:p.Pro610Ser	EARLY
CAS-E	RM	12;49445638;G;A	0.67	missense variant	KMT2D	.	unknown (0)	.	ENSP00000301067.7:p.Pro610Ser	EARLY
CAS-E	DM1	20;50140259;G;A	0.50	missense variant	NFATC2	.	probably damaging (0.999)	tolerated (1)	ENSP00000396471.1:p.Ser154Phe	EARLY
CAS-E	DM2	20;50140259;G;A	0.50	missense variant	NFATC2	.	probably damaging (0.999)	tolerated (1)	ENSP00000396471.1:p.Ser154Phe	EARLY
CAS-E	DM3	20;50140259;G;A	0.49	missense variant	NFATC2	.	probably damaging (0.999)	tolerated (1)	ENSP00000396471.1:p.Ser154Phe	EARLY
CAS-E	DM4	20;50140259;G;A	0.50	missense variant	NFATC2	.	probably damaging (0.999)	tolerated (1)	ENSP00000396471.1:p.Ser154Phe	EARLY
CAS-E	DM5	20;50140259;G;A	0.49	missense variant	NFATC2	.	probably damaging (0.999)	tolerated (1)	ENSP00000396471.1:p.Ser154Phe	EARLY
CAS-E	P	20;50140259;G;A	0.50	missense variant	NFATC2	.	probably damaging (0.999)	tolerated (1)	ENSP00000396471.1:p.Ser154Phe	EARLY
CAS-E	RM	20;50140259;G;A	0.50	missense variant	NFATC2	.	probably damaging (0.999)	tolerated (1)	ENSP00000396471.1:p.Ser154Phe	EARLY
CAS-E	DM2	1;115256530;G;T	0.20	missense variant	NRAS	580	benign (0.045)	deleterious (0.01)	ENSP00000358548.4:p.Gln61Lys	LATE
CAS-E	DM3	1;115256530;G;T	0.13	missense variant	NRAS	580	benign (0.045)	deleterious (0.01)	ENSP00000358548.4:p.Gln61Lys	LATE
CAS-E	DM5	1;115256530;G;T	0.15	missense variant	NRAS	580	benign (0.045)	deleterious (0.01)	ENSP00000358548.4:p.Gln61Lys	LATE
CAS-E	DM1	16;23641109;A;G	0.66	missense variant	PALB2	.	benign (0.126)	deleterious (0.01)	ENSP00000261584.4:p.Leu789Pro	EARLY
CAS-E	DM2	16;23641109;A;G	0.98	missense variant	PALB2	.	benign (0.126)	deleterious (0.01)	ENSP00000261584.4:p.Leu789Pro	EARLY
CAS-E	DM3	16;23641109;A;G	0.67	missense variant	PALB2	.	benign (0.126)	deleterious (0.01)	ENSP00000261584.4:p.Leu789Pro	EARLY
CAS-E	DM4	16;23641109;A;G	0.50	missense variant	PALB2	.	benign (0.126)	deleterious (0.01)	ENSP00000261584.4:p.Leu789Pro	EARLY
CAS-E	DM5	16;23641109;A;G	0.50	missense variant	PALB2	.	benign (0.126)	deleterious (0.01)	ENSP00000261584.4:p.Leu789Pro	EARLY

CAS-E	P	16;23641109;A;G	0.50	missense variant	PALB2	.	benign (0.126)	deleterious (0.01)	ENSP00000261584.4:p.Leu789Pro	EARLY
CAS-E	RM	16;23641109;A;G	0.50	missense variant	PALB2	.	benign (0.126)	deleterious (0.01)	ENSP00000261584.4:p.Leu789Pro	EARLY
CAS-E	DM1	9;127920559;G;A	0.58	missense variant	PPP6C	23144,1167934	benign (0.117)	deleterious (0.02)	ENSP00000392147.1:p.His151Tyr	EARLY
CAS-E	DM2	9;127920559;G;A	0.89	missense variant	PPP6C	23144,1167934	benign (0.117)	deleterious (0.02)	ENSP00000392147.1:p.His151Tyr	EARLY
CAS-E	DM3	9;127920559;G;A	0.63	missense variant	PPP6C	23144,1167934	benign (0.117)	deleterious (0.02)	ENSP00000392147.1:p.His151Tyr	EARLY
CAS-E	DM4	9;127920559;G;A	1.00	missense variant	PPP6C	23144,1167934	benign (0.117)	deleterious (0.02)	ENSP00000392147.1:p.His151Tyr	EARLY
CAS-E	DM5	9;127920559;G;A	0.86	missense variant	PPP6C	23144,1167934	benign (0.117)	deleterious (0.02)	ENSP00000392147.1:p.His151Tyr	EARLY
CAS-E	P	9;127920559;G;A	0.56	missense variant	PPP6C	23144,1167934	benign (0.117)	deleterious (0.02)	ENSP00000392147.1:p.His151Tyr	EARLY
CAS-E	RM	9;127920559;G;A	0.65	missense variant	PPP6C	23144,1167934	benign (0.117)	deleterious (0.02)	ENSP00000392147.1:p.His151Tyr	EARLY
CAS-E	DM1	2;214239772;C;T	0.67	missense variant	SPAG16	.	benign (0.214)	tolerated (0.2)	ENSP00000332592.5:p.Pro291Ser	EARLY
CAS-E	DM2	2;214239772;C;T	0.43	missense variant	SPAG16	.	benign (0.214)	tolerated (0.2)	ENSP00000332592.5:p.Pro291Ser	EARLY
CAS-E	DM3	2;214239772;C;T	0.48	missense variant	SPAG16	.	benign (0.214)	tolerated (0.2)	ENSP00000332592.5:p.Pro291Ser	EARLY
CAS-E	DM4	2;214239772;C;T	0.50	missense variant	SPAG16	.	benign (0.214)	tolerated (0.2)	ENSP00000332592.5:p.Pro291Ser	EARLY
CAS-E	DM5	2;214239772;C;T	0.34	missense variant	SPAG16	.	benign (0.214)	tolerated (0.2)	ENSP00000332592.5:p.Pro291Ser	EARLY
CAS-E	P	2;214239772;C;T	0.24	missense variant	SPAG16	.	benign (0.214)	tolerated (0.2)	ENSP00000332592.5:p.Pro291Ser	EARLY
CAS-E	RM	2;214239772;C;T	0.67	missense variant	SPAG16	.	benign (0.214)	tolerated (0.2)	ENSP00000332592.5:p.Pro291Ser	EARLY
CAS-E	DM1	17;7578472;G;A	0.28	missense variant	TP53	44367	benign (0.014)	tolerated (0.21)	ENSP00000398846.2:p.Pro153Leu	EARLY
CAS-E	DM2	17;7578472;G;A	0.40	missense variant	TP53	44367	benign (0.014)	tolerated (0.21)	ENSP00000398846.2:p.Pro153Leu	EARLY
CAS-E	DM3	17;7578472;G;A	0.31	missense variant	TP53	44367	benign (0.014)	tolerated (0.21)	ENSP00000398846.2:p.Pro153Leu	EARLY
CAS-E	DM5	17;7578472;G;A	0.31	missense variant	TP53	44367	benign (0.014)	tolerated (0.21)	ENSP00000398846.2:p.Pro153Leu	EARLY
CAS-E	P	17;7578472;G;A	0.35	missense variant	TP53	44367	benign (0.014)	tolerated (0.21)	ENSP00000398846.2:p.Pro153Leu	EARLY
CAS-E	RM	17;7578472;G;A	0.39	missense variant	TP53	44367	benign (0.014)	tolerated (0.21)	ENSP00000398846.2:p.Pro153Leu	EARLY
CAS-E	DM1	16;2134253;G;A	0.67	missense variant	TSC2	.	probably damaging (0.977)	tolerated (0.14)	ENSP00000371978.6:p.Glu1229Lys	EARLY
CAS-E	DM2	16;2134253;G;A	1.00	missense variant	TSC2	.	probably damaging (0.977)	tolerated (0.14)	ENSP00000371978.6:p.Glu1229Lys	EARLY
CAS-E	DM3	16;2134253;G;A	0.62	missense variant	TSC2	.	probably damaging (0.977)	tolerated (0.14)	ENSP00000371978.6:p.Glu1229Lys	EARLY
CAS-E	DM4	16;2134253;G;A	1.00	missense variant	TSC2	.	probably damaging (0.977)	tolerated (0.14)	ENSP00000371978.6:p.Glu1229Lys	EARLY
CAS-E	DM5	16;2134253;G;A	0.50	missense variant	TSC2	.	probably damaging (0.977)	tolerated (0.14)	ENSP00000371978.6:p.Glu1229Lys	EARLY
CAS-E	P	16;2134253;G;A	0.50	missense variant	TSC2	.	probably damaging (0.977)	tolerated (0.14)	ENSP00000371978.6:p.Glu1229Lys	EARLY
CAS-E	RM	16;2134253;G;A	0.41	missense variant	TSC2	.	probably damaging (0.977)	tolerated (0.14)	ENSP00000371978.6:p.Glu1229Lys	EARLY
CAS-A	DM1	5;112173567;C;T	0.55	missense variant	APC	.	probably damaging (0.999)	deleterious (0)	ENSP00000423828.2:p.Ala759Val	EARLY
CAS-A	DM2	5;112173567;C;T	0.50	missense variant	APC	.	probably damaging (0.999)	deleterious (0)	ENSP00000423828.2:p.Ala759Val	EARLY
CAS-A	DM3	5;112173567;C;T	0.75	missense variant	APC	.	probably damaging (0.999)	deleterious (0)	ENSP00000423828.2:p.Ala759Val	EARLY
CAS-A	DM4	5;112173567;C;T	0.45	missense variant	APC	.	probably damaging (0.999)	deleterious (0)	ENSP00000423828.2:p.Ala759Val	EARLY
CAS-A	RM1	5;112173567;C;T	0.40	missense variant	APC	.	probably damaging (0.999)	deleterious (0)	ENSP00000423828.2:p.Ala759Val	EARLY
CAS-A	RM2	5;112173567;C;T	0.34	missense variant	APC	.	probably damaging (0.999)	deleterious (0)	ENSP00000423828.2:p.Ala759Val	EARLY
CAS-A	RM3	5;112173567;C;T	0.47	missense variant	APC	.	probably damaging (0.999)	deleterious (0)	ENSP00000423828.2:p.Ala759Val	EARLY
CAS-A	DM1	7;140453136;A;T	0.51	missense variant	BRAF	476	possibly damaging (0.792)	deleterious (0)	ENSP00000288602.6:p.Val600Glu	EARLY
CAS-A	DM2	7;140453136;A;T	0.67	missense variant	BRAF	476	possibly damaging (0.792)	deleterious (0)	ENSP00000288602.6:p.Val600Glu	EARLY

CAS-A	DM3	7;140453136;A;T	0.60	missense variant	BRAF	476	possibly damaging (0.792)	deleterious (0)	ENSP00000288602.6:p.Val600Glu	EARLY
CAS-A	DM4	7;140453136;A;T	0.67	missense variant	BRAF	476	possibly damaging (0.792)	deleterious (0)	ENSP00000288602.6:p.Val600Glu	EARLY
CAS-A	RM1	7;140453136;A;T	0.79	missense variant	BRAF	476	possibly damaging (0.792)	deleterious (0)	ENSP00000288602.6:p.Val600Glu	EARLY
CAS-A	RM2	7;140453136;A;T	0.73	missense variant	BRAF	476	possibly damaging (0.792)	deleterious (0)	ENSP00000288602.6:p.Val600Glu	EARLY
CAS-A	RM3	7;140453136;A;T	0.83	missense variant	BRAF	476	possibly damaging (0.792)	deleterious (0)	ENSP00000288602.6:p.Val600Glu	EARLY
CAS-A	DM1	14;95578562;C;A	0.36	missense variant	DICER1	.	possibly damaging (0.703)	tolerated (0.44)	ENSP00000435681.1:p.Arg688Leu	LATE
CAS-A	DM1	16;9858331;C;T	0.49	missense variant	GRIN2A	.	benign (0.003)	tolerated (0.79)	ENSP00000454998.1:p.Asp1024Asn	EARLY
CAS-A	DM2	16;9858331;C;T	0.41	missense variant	GRIN2A	.	benign (0.003)	tolerated (0.79)	ENSP00000454998.1:p.Asp1024Asn	EARLY
CAS-A	DM3	16;9858331;C;T	0.38	missense variant	GRIN2A	.	benign (0.003)	tolerated (0.79)	ENSP00000454998.1:p.Asp1024Asn	EARLY
CAS-A	DM4	16;9858331;C;T	0.50	missense variant	GRIN2A	.	benign (0.003)	tolerated (0.79)	ENSP00000454998.1:p.Asp1024Asn	EARLY
CAS-A	RM1	16;9858331;C;T	0.50	missense variant	GRIN2A	.	benign (0.003)	tolerated (0.79)	ENSP00000454998.1:p.Asp1024Asn	EARLY
CAS-A	RM2	16;9858331;C;T	0.50	missense variant	GRIN2A	.	benign (0.003)	tolerated (0.79)	ENSP00000454998.1:p.Asp1024Asn	EARLY
CAS-A	RM3	16;9858331;C;T	0.54	missense variant	GRIN2A	.	benign (0.003)	tolerated (0.79)	ENSP00000454998.1:p.Asp1024Asn	EARLY
CAS-A	RM1	7;151927021;C;A	0.40	missense variant	KMT2C	150426,150427	probably damaging (1)	deleterious (0)	ENSP00000262189.6:p.Cys988Phe	EARLY
CAS-A	RM2	7;151927021;C;A	0.30	missense variant	KMT2C	150426,150427	probably damaging (1)	deleterious (0)	ENSP00000262189.6:p.Cys988Phe	EARLY
CAS-A	RM3	7;151927021;C;A	0.21	missense variant	KMT2C	150426,150427	probably damaging (1)	deleterious (0)	ENSP00000262189.6:p.Cys988Phe	EARLY
CAS-A	DM2	7;151927021;C;A	0.11	missense variant	KMT2C	150426,150427	probably damaging (1)	deleterious (0)	ENSP00000262189.6:p.Cys988Phe	EARLY
CAS-A	DM3	7;151927021;C;A	0.07	missense variant	KMT2C	150426,150427	probably damaging (1)	deleterious (0)	ENSP00000262189.6:p.Cys988Phe	EARLY
CAS-A	DM1	1;115256530;G;T	0.81	missense variant	NRAS	580	benign (0.045)	deleterious (0.01)	ENSP00000358548.4:p.Gln61Lys	LATE
CAS-A	DM2	1;115256530;G;T	0.75	missense variant	NRAS	580	benign (0.045)	deleterious (0.01)	ENSP00000358548.4:p.Gln61Lys	LATE
CAS-A	DM3	1;115256530;G;T	0.23	missense variant	NRAS	580	benign (0.045)	deleterious (0.01)	ENSP00000358548.4:p.Gln61Lys	LATE
CAS-A	DM4	1;115256530;G;T	0.74	missense variant	NRAS	580	benign (0.045)	deleterious (0.01)	ENSP00000358548.4:p.Gln61Lys	LATE
CAS-A	DM1	3;178936094;C;A	0.52	missense variant	PIK3CA	766,255876	probably damaging (0.952)	tolerated (0.23)	ENSP00000263967.3:p.Gln546Lys	EARLY
CAS-A	DM2	3;178936094;C;A	0.19	missense variant	PIK3CA	766,255876	probably damaging (0.952)	tolerated (0.23)	ENSP00000263967.3:p.Gln546Lys	EARLY
CAS-A	DM3	3;178936094;C;A	0.16	missense variant	PIK3CA	766,255876	probably damaging (0.952)	tolerated (0.23)	ENSP00000263967.3:p.Gln546Lys	EARLY
CAS-A	DM4	3;178936094;C;A	0.44	missense variant	PIK3CA	766,255876	probably damaging (0.952)	tolerated (0.23)	ENSP00000263967.3:p.Gln546Lys	EARLY
CAS-A	RM2	3;178936094;C;A	0.19	missense variant	PIK3CA	766,255876	probably damaging (0.952)	tolerated (0.23)	ENSP00000263967.3:p.Gln546Lys	EARLY
CAS-A	DM1	12;47186852;C;T	0.45	initiator codon variant	SLC38A4	.	benign (0.104)	tolerated (0.06)	ENSP00000389843.1:p.Met1?	EARLY
CAS-A	DM2	12;47186852;C;T	0.50	initiator codon variant	SLC38A4	.	benign (0.104)	tolerated (0.06)	ENSP00000389843.1:p.Met1?	EARLY
CAS-A	DM3	12;47186852;C;T	0.50	initiator codon variant	SLC38A4	.	benign (0.104)	tolerated (0.06)	ENSP00000389843.1:p.Met1?	EARLY
CAS-A	DM4	12;47186852;C;T	0.50	initiator codon variant	SLC38A4	.	benign (0.104)	tolerated (0.06)	ENSP00000389843.1:p.Met1?	EARLY
CAS-A	RM1	12;47186852;C;T	0.36	initiator codon variant	SLC38A4	.	benign (0.104)	tolerated (0.06)	ENSP00000389843.1:p.Met1?	EARLY
CAS-A	RM2	12;47186852;C;T	0.45	initiator codon variant	SLC38A4	.	benign (0.104)	tolerated (0.06)	ENSP00000389843.1:p.Met1?	EARLY
CAS-A	RM3	12;47186852;C;T	0.50	initiator codon variant	SLC38A4	.	benign (0.104)	tolerated (0.06)	ENSP00000389843.1:p.Met1?	EARLY
CAS-A	DM1	10;70332519;T;C	0.34	missense variant	TET1	.	benign (0.003)	tolerated (0.51)	ENSP00000362748.4:p.Tyr142His	LATE
CAS-A	DM1	10;70406105;C;T	0.93	missense variant	TET1	.	probably damaging (0.931)	deleterious (0.01)	ENSP00000362748.4:p.Pro1207Ser	EARLY
CAS-A	DM2	10;70406105;C;T	1.00	missense variant	TET1	.	probably damaging (0.931)	deleterious (0.01)	ENSP00000362748.4:p.Pro1207Ser	EARLY
CAS-A	DM3	10;70406105;C;T	0.81	missense variant	TET1	.	probably damaging (0.931)	deleterious (0.01)	ENSP00000362748.4:p.Pro1207Ser	EARLY
CAS-A	DM4	10;70406105;C;T	0.91	missense variant	TET1	.	probably damaging (0.931)	deleterious (0.01)	ENSP00000362748.4:p.Pro1207Ser	EARLY

CAS-A	RM1	10;70406105;C;T	1.00	missense variant	TET1	.	probably damaging (0.931)	deleterious (0.01)	ENSP00000362748.4:p.Pro1207Ser	EARLY
CAS-A	RM2	10;70406105;C;T	0.85	missense variant	TET1	.	probably damaging (0.931)	deleterious (0.01)	ENSP00000362748.4:p.Pro1207Ser	EARLY
CAS-A	RM3	10;70406105;C;T	1.00	missense variant	TET1	.	probably damaging (0.931)	deleterious (0.01)	ENSP00000362748.4:p.Pro1207Ser	EARLY
CAS-A	DM1	8;30982117;C;T	0.50	missense variant	WRN	.	benign (0.003)	tolerated (0.17)	ENSP00000298139.5:p.His904Tyr	EARLY
CAS-A	DM2	8;30982117;C;T	0.50	missense variant	WRN	.	benign (0.003)	tolerated (0.17)	ENSP00000298139.5:p.His904Tyr	EARLY
CAS-A	DM3	8;30982117;C;T	0.65	missense variant	WRN	.	benign (0.003)	tolerated (0.17)	ENSP00000298139.5:p.His904Tyr	EARLY
CAS-A	DM4	8;30982117;C;T	0.50	missense variant	WRN	.	benign (0.003)	tolerated (0.17)	ENSP00000298139.5:p.His904Tyr	EARLY
CAS-A	RM1	8;30982117;C;T	0.44	missense variant	WRN	.	benign (0.003)	tolerated (0.17)	ENSP00000298139.5:p.His904Tyr	EARLY
CAS-A	RM2	8;30982117;C;T	0.49	missense variant	WRN	.	benign (0.003)	tolerated (0.17)	ENSP00000298139.5:p.His904Tyr	EARLY
CAS-A	RM3	8;30982117;C;T	0.36	missense variant	WRN	.	benign (0.003)	tolerated (0.17)	ENSP00000298139.5:p.His904Tyr	EARLY
CAS-B	DM1	7;140453136;A;T	0.67	missense variant	BRAF	476	possibly damaging (0.792)	deleterious (0)	ENSP00000288602.6:p.Val600Glu	EARLY
CAS-B	DM2	7;140453136;A;T	0.71	missense variant	BRAF	476	possibly damaging (0.792)	deleterious (0)	ENSP00000288602.6:p.Val600Glu	EARLY
CAS-B	DM3	7;140453136;A;T	0.61	missense variant	BRAF	476	possibly damaging (0.792)	deleterious (0)	ENSP00000288602.6:p.Val600Glu	EARLY
CAS-B	DM4	7;140453136;A;T	0.61	missense variant	BRAF	476	possibly damaging (0.792)	deleterious (0)	ENSP00000288602.6:p.Val600Glu	EARLY
CAS-B	DM5	7;140453136;A;T	0.68	missense variant	BRAF	476	possibly damaging (0.792)	deleterious (0)	ENSP00000288602.6:p.Val600Glu	EARLY
CAS-B	P	7;140453136;A;T	0.66	missense variant	BRAF	476	possibly damaging (0.792)	deleterious (0)	ENSP00000288602.6:p.Val600Glu	EARLY
CAS-B	RM1	7;140453136;A;T	0.67	missense variant	BRAF	476	possibly damaging (0.792)	deleterious (0)	ENSP00000288602.6:p.Val600Glu	EARLY
CAS-B	RM2	7;140453136;A;T	0.66	missense variant	BRAF	476	possibly damaging (0.792)	deleterious (0)	ENSP00000288602.6:p.Val600Glu	EARLY
CAS-B	RM3	7;140453136;A;T	0.66	missense variant	BRAF	476	possibly damaging (0.792)	deleterious (0)	ENSP00000288602.6:p.Val600Glu	EARLY
CAS-B	DM1	1;36933492;G;A	0.64	missense variant	CSF3R	.	benign (0.119)	tolerated (0.39)	ENSP00000355406.4:p.His599Tyr	EARLY
CAS-B	DM2	1;36933492;G;A	0.55	missense variant	CSF3R	.	benign (0.119)	tolerated (0.39)	ENSP00000355406.4:p.His599Tyr	EARLY
CAS-B	DM3	1;36933492;G;A	0.63	missense variant	CSF3R	.	benign (0.119)	tolerated (0.39)	ENSP00000355406.4:p.His599Tyr	EARLY
CAS-B	DM4	1;36933492;G;A	0.47	missense variant	CSF3R	.	benign (0.119)	tolerated (0.39)	ENSP00000355406.4:p.His599Tyr	EARLY
CAS-B	DM5	1;36933492;G;A	0.67	missense variant	CSF3R	.	benign (0.119)	tolerated (0.39)	ENSP00000355406.4:p.His599Tyr	EARLY
CAS-B	P	1;36933492;G;A	0.38	missense variant	CSF3R	.	benign (0.119)	tolerated (0.39)	ENSP00000355406.4:p.His599Tyr	EARLY
CAS-B	RM1	1;36933492;G;A	0.44	missense variant	CSF3R	.	benign (0.119)	tolerated (0.39)	ENSP00000355406.4:p.His599Tyr	EARLY
CAS-B	RM2	1;36933492;G;A	0.50	missense variant	CSF3R	.	benign (0.119)	tolerated (0.39)	ENSP00000355406.4:p.His599Tyr	EARLY
CAS-B	RM3	1;36933492;G;A	0.49	missense variant	CSF3R	.	benign (0.119)	tolerated (0.39)	ENSP00000355406.4:p.His599Tyr	EARLY
CAS-B	DM1	7;151874960;T;G	0.27	missense variant	KMT2C	.	possibly damaging (0.714)	tolerated (0.12)	ENSP00000262189.6:p.Leu2526Phe	EARLY
CAS-B	DM2	7;151874960;T;G	0.35	missense variant	KMT2C	.	possibly damaging (0.714)	tolerated (0.12)	ENSP00000262189.6:p.Leu2526Phe	EARLY
CAS-B	DM3	7;151874960;T;G	0.28	missense variant	KMT2C	.	possibly damaging (0.714)	tolerated (0.12)	ENSP00000262189.6:p.Leu2526Phe	EARLY
CAS-B	DM4	7;151874960;T;G	0.32	missense variant	KMT2C	.	possibly damaging (0.714)	tolerated (0.12)	ENSP00000262189.6:p.Leu2526Phe	EARLY
CAS-B	DM5	7;151874960;T;G	0.32	missense variant	KMT2C	.	possibly damaging (0.714)	tolerated (0.12)	ENSP00000262189.6:p.Leu2526Phe	EARLY
CAS-B	P	7;151874960;T;G	0.34	missense variant	KMT2C	.	possibly damaging (0.714)	tolerated (0.12)	ENSP00000262189.6:p.Leu2526Phe	EARLY
CAS-B	RM1	7;151874960;T;G	0.36	missense variant	KMT2C	.	possibly damaging (0.714)	tolerated (0.12)	ENSP00000262189.6:p.Leu2526Phe	EARLY
CAS-B	RM2	7;151874960;T;G	0.35	missense variant	KMT2C	.	possibly damaging (0.714)	tolerated (0.12)	ENSP00000262189.6:p.Leu2526Phe	EARLY
CAS-B	RM3	7;151874960;T;G	0.29	missense variant	KMT2C	.	possibly damaging (0.714)	tolerated (0.12)	ENSP00000262189.6:p.Leu2526Phe	EARLY

CAS-B	DM5	1;198678842;A;T	0.22	missense variant	PTPRC	.	benign (0.02)	tolerated (0.29)	ENSP00000356337.5:p.Ile288Phe	LATE
CAS-B	DM1	17;7574003;G;A	0.99	stop gained	TP53	11073,99721	.	.	ENSP00000391478.2:p.Arg342Ter	EARLY
CAS-B	DM2	17;7574003;G;A	1.00	stop gained	TP53	11073,99721	.	.	ENSP00000391478.2:p.Arg342Ter	EARLY
CAS-B	DM3	17;7574003;G;A	1.00	stop gained	TP53	11073,99721	.	.	ENSP00000391478.2:p.Arg342Ter	EARLY
CAS-B	DM4	17;7574003;G;A	0.65	stop gained	TP53	11073,99721	.	.	ENSP00000391478.2:p.Arg342Ter	EARLY
CAS-B	DM5	17;7574003;G;A	0.89	stop gained	TP53	11073,99721	.	.	ENSP00000391478.2:p.Arg342Ter	EARLY
CAS-B	P	17;7574003;G;A	1.00	stop gained	TP53	11073,99721	.	.	ENSP00000391478.2:p.Arg342Ter	EARLY
CAS-B	RM1	17;7574003;G;A	0.90	stop gained	TP53	11073,99721	.	.	ENSP00000391478.2:p.Arg342Ter	EARLY
CAS-B	RM2	17;7574003;G;A	0.71	stop gained	TP53	11073,99721	.	.	ENSP00000391478.2:p.Arg342Ter	EARLY
CAS-B	RM3	17;7574003;G;A	0.84	stop gained	TP53	11073,99721	.	.	ENSP00000391478.2:p.Arg342Ter	EARLY
CAS-B	DM1	17;5036838;G;A	0.23	missense variant	USP6	.	benign (0)	tolerated (1)	ENSP00000328010.4:p.Arg126Lys	EARLY
CAS-B	DM2	17;5036838;G;A	0.05	missense variant	USP6	.	benign (0)	tolerated (1)	ENSP00000328010.4:p.Arg126Lys	EARLY
CAS-B	DM3	17;5036838;G;A	0.14	missense variant	USP6	.	benign (0)	tolerated (1)	ENSP00000328010.4:p.Arg126Lys	EARLY
CAS-B	DM4	17;5036838;G;A	0.73	missense variant	USP6	.	benign (0)	tolerated (1)	ENSP00000328010.4:p.Arg126Lys	EARLY
CAS-B	DM5	17;5036838;G;A	0.16	missense variant	USP6	.	benign (0)	tolerated (1)	ENSP00000328010.4:p.Arg126Lys	EARLY
CAS-B	P	17;5036838;G;A	0.49	missense variant	USP6	.	benign (0)	tolerated (1)	ENSP00000328010.4:p.Arg126Lys	EARLY
CAS-B	RM1	17;5036838;G;A	0.33	missense variant	USP6	.	benign (0)	tolerated (1)	ENSP00000328010.4:p.Arg126Lys	EARLY
CAS-B	RM2	17;5036838;G;A	0.29	missense variant	USP6	.	benign (0)	tolerated (1)	ENSP00000328010.4:p.Arg126Lys	EARLY
CAS-B	RM3	17;5036838;G;A	0.22	missense variant	USP6	.	benign (0)	tolerated (1)	ENSP00000328010.4:p.Arg126Lys	EARLY
CAS-C	DM1	7;140453134;T;C	0.89	missense variant	BRAF	478	benign (0.195)	deleterious (0)	ENSP00000288602.6:p.Lys601Glu	EARLY
CAS-C	DM2	7;140453134;T;C	0.77	missense variant	BRAF	478	benign (0.195)	deleterious (0)	ENSP00000288602.6:p.Lys601Glu	EARLY
CAS-C	DM3	7;140453134;T;C	0.86	missense variant	BRAF	478	benign (0.195)	deleterious (0)	ENSP00000288602.6:p.Lys601Glu	EARLY
CAS-C	DM4	7;140453134;T;C	0.86	missense variant	BRAF	478	benign (0.195)	deleterious (0)	ENSP00000288602.6:p.Lys601Glu	EARLY
CAS-C	DM5	7;140453134;T;C	0.78	missense variant	BRAF	478	benign (0.195)	deleterious (0)	ENSP00000288602.6:p.Lys601Glu	EARLY
CAS-C	DM6	7;140453134;T;C	0.85	missense variant	BRAF	478	benign (0.195)	deleterious (0)	ENSP00000288602.6:p.Lys601Glu	EARLY
CAS-C	P	7;140453134;T;C	0.83	missense variant	BRAF	478	benign (0.195)	deleterious (0)	ENSP00000288602.6:p.Lys601Glu	EARLY
CAS-C	DM1	7;2977660;C;T	0.49	missense variant	CARD11	.	possibly damaging (0.883)	deleterious (0.04)	ENSP00000380150.4:p.Glu342Lys	EARLY
CAS-C	DM2	7;2977660;C;T	0.50	missense variant	CARD11	.	possibly damaging (0.883)	deleterious (0.04)	ENSP00000380150.4:p.Glu342Lys	EARLY
CAS-C	DM3	7;2977660;C;T	0.34	missense variant	CARD11	.	possibly damaging (0.883)	deleterious (0.04)	ENSP00000380150.4:p.Glu342Lys	EARLY
CAS-C	DM4	7;2977660;C;T	0.35	missense variant	CARD11	.	possibly damaging (0.883)	deleterious (0.04)	ENSP00000380150.4:p.Glu342Lys	EARLY
CAS-C	DM5	7;2977660;C;T	0.64	missense variant	CARD11	.	possibly damaging (0.883)	deleterious (0.04)	ENSP00000380150.4:p.Glu342Lys	EARLY
CAS-C	DM6	7;2977660;C;T	0.50	missense variant	CARD11	.	possibly damaging (0.883)	deleterious (0.04)	ENSP00000380150.4:p.Glu342Lys	EARLY
CAS-C	P	7;2977660;C;T	0.43	missense variant	CARD11	.	possibly damaging (0.883)	deleterious (0.04)	ENSP00000380150.4:p.Glu342Lys	EARLY
CAS-C	DM1	4;126328110;G;A	0.47	missense variant	FAT4	.	benign (0.346)	tolerated (0.49)	ENSP00000335169.5:p.Asp93Asn	EARLY
CAS-C	DM2	4;126328110;G;A	0.50	missense variant	FAT4	.	benign (0.346)	tolerated (0.49)	ENSP00000335169.5:p.Asp93Asn	EARLY
CAS-C	DM3	4;126328110;G;A	0.42	missense variant	FAT4	.	benign (0.346)	tolerated (0.49)	ENSP00000335169.5:p.Asp93Asn	EARLY
CAS-C	DM4	4;126328110;G;A	0.39	missense variant	FAT4	.	benign (0.346)	tolerated (0.49)	ENSP00000335169.5:p.Asp93Asn	EARLY
CAS-C	DM5	4;126328110;G;A	0.40	missense variant	FAT4	.	benign (0.346)	tolerated (0.49)	ENSP00000335169.5:p.Asp93Asn	EARLY
CAS-C	DM6	4;126328110;G;A	0.50	missense variant	FAT4	.	benign (0.346)	tolerated (0.49)	ENSP00000335169.5:p.Asp93Asn	EARLY
CAS-C	P	4;126328110;G;A	0.15	missense variant	FAT4	.	benign (0.346)	tolerated (0.49)	ENSP00000335169.5:p.Asp93Asn	EARLY
CAS-C	DM1	4;55948732;C;T	0.50	missense variant	KDR	.	possibly damaging (0.784)	tolerated (0.44)	ENSP00000263923.4:p.Glu1245Lys	EARLY

CAS-C	DM2	4;55948732;C;T	0.50	missense variant	KDR	.	possibly damaging (0.784)	tolerated (0.44)	ENSP00000263923.4:p.Glu1245Lys	EARLY
CAS-C	DM3	4;55948732;C;T	0.39	missense variant	KDR	.	possibly damaging (0.784)	tolerated (0.44)	ENSP00000263923.4:p.Glu1245Lys	EARLY
CAS-C	DM4	4;55948732;C;T	0.47	missense variant	KDR	.	possibly damaging (0.784)	tolerated (0.44)	ENSP00000263923.4:p.Glu1245Lys	EARLY
CAS-C	DM5	4;55948732;C;T	0.50	missense variant	KDR	.	possibly damaging (0.784)	tolerated (0.44)	ENSP00000263923.4:p.Glu1245Lys	EARLY
CAS-C	DM6	4;55948732;C;T	0.47	missense variant	KDR	.	possibly damaging (0.784)	tolerated (0.44)	ENSP00000263923.4:p.Glu1245Lys	EARLY
CAS-C	P	4;55948732;C;T	0.50	missense variant	KDR	.	possibly damaging (0.784)	tolerated (0.44)	ENSP00000263923.4:p.Glu1245Lys	EARLY
CAS-C	DM1	7;151848068;C;T	0.22	missense variant	KMT2C	.	benign (0.044)	deleterious (0.05)	ENSP00000262189.6:p.Val4231Ile	EARLY
CAS-C	DM2	7;151848068;C;T	0.13	missense variant	KMT2C	.	benign (0.044)	deleterious (0.05)	ENSP00000262189.6:p.Val4231Ile	EARLY
CAS-C	DM3	7;151848068;C;T	0.17	missense variant	KMT2C	.	benign (0.044)	deleterious (0.05)	ENSP00000262189.6:p.Val4231Ile	EARLY
CAS-C	DM4	7;151848068;C;T	0.16	missense variant	KMT2C	.	benign (0.044)	deleterious (0.05)	ENSP00000262189.6:p.Val4231Ile	EARLY
CAS-C	DM5	7;151848068;C;T	0.18	missense variant	KMT2C	.	benign (0.044)	deleterious (0.05)	ENSP00000262189.6:p.Val4231Ile	EARLY
CAS-C	DM6	7;151848068;C;T	0.16	missense variant	KMT2C	.	benign (0.044)	deleterious (0.05)	ENSP00000262189.6:p.Val4231Ile	EARLY
CAS-C	P	7;151848068;C;T	0.24	missense variant	KMT2C	.	benign (0.044)	deleterious (0.05)	ENSP00000262189.6:p.Val4231Ile	EARLY
CAS-C	DM1	20;50090574;G;A	0.43	missense variant	NFATC2	.	benign (0.064)	tolerated (0.11)	ENSP00000396471.1:p.Pro531Ser	EARLY
CAS-C	DM2	20;50090574;G;A	0.50	missense variant	NFATC2	.	benign (0.064)	tolerated (0.11)	ENSP00000396471.1:p.Pro531Ser	EARLY
CAS-C	DM3	20;50090574;G;A	0.50	missense variant	NFATC2	.	benign (0.064)	tolerated (0.11)	ENSP00000396471.1:p.Pro531Ser	EARLY
CAS-C	DM4	20;50090574;G;A	0.29	missense variant	NFATC2	.	benign (0.064)	tolerated (0.11)	ENSP00000396471.1:p.Pro531Ser	EARLY
CAS-C	DM5	20;50090574;G;A	0.48	missense variant	NFATC2	.	benign (0.064)	tolerated (0.11)	ENSP00000396471.1:p.Pro531Ser	EARLY
CAS-C	DM6	20;50090574;G;A	0.46	missense variant	NFATC2	.	benign (0.064)	tolerated (0.11)	ENSP00000396471.1:p.Pro531Ser	EARLY
CAS-C	P	20;50090574;G;A	0.39	missense variant	NFATC2	.	benign (0.064)	tolerated (0.11)	ENSP00000396471.1:p.Pro531Ser	EARLY
CAS-C	P	12;70933756;G;A	0.20	stop gained	PTPRB	.	.	.	ENSP00000447302.1:p.Arg1573Ter	EARLY
CAS-C	P	3;128348950;G;A	0.15	missense variant	RPN1	.	probably damaging (1)	deleterious (0)	ENSP00000417529.1:p.Arg122Trp	EARLY
CAS-C	DM3	1;186307212;G;A	0.14	missense variant	TPR	.	.	deleterious (0.01)	ENSP00000356448.3:p.Arg1439Cys	LATE
CAS-C	DM3	17;5037195;G;A	0.06	missense variant	USP6	.	benign (0)	tolerated (1)	ENSP00000328010.4:p.Arg133Lys	EARLY
CAS-C	DM4	17;5037195;G;A	0.06	missense variant	USP6	.	benign (0)	tolerated (1)	ENSP00000328010.4:p.Arg133Lys	EARLY
CAS-C	DM5	17;5037195;G;A	0.13	missense variant	USP6	.	benign (0)	tolerated (1)	ENSP00000328010.4:p.Arg133Lys	EARLY
CAS-C	DM6	17;5037195;G;A	0.02	missense variant	USP6	.	benign (0)	tolerated (1)	ENSP00000328010.4:p.Arg133Lys	EARLY
CAS-C	P	17;5037195;G;A	0.04	missense variant	USP6	.	benign (0)	tolerated (1)	ENSP00000328010.4:p.Arg133Lys	EARLY
CAS-C	DM1	8;30998973;C;T	0.48	missense variant	WRN	136658	probably damaging (0.999)	deleterious (0)	ENSP00000298139.5:p.Arg999Cys	EARLY
CAS-C	DM2	8;30998973;C;T	0.50	missense variant	WRN	136658	probably damaging (0.999)	deleterious (0)	ENSP00000298139.5:p.Arg999Cys	EARLY
CAS-C	DM3	8;30998973;C;T	0.42	missense variant	WRN	136658	probably damaging (0.999)	deleterious (0)	ENSP00000298139.5:p.Arg999Cys	EARLY
CAS-C	DM4	8;30998973;C;T	0.46	missense variant	WRN	136658	probably damaging (0.999)	deleterious (0)	ENSP00000298139.5:p.Arg999Cys	EARLY
CAS-C	DM5	8;30998973;C;T	0.50	missense variant	WRN	136658	probably damaging (0.999)	deleterious (0)	ENSP00000298139.5:p.Arg999Cys	EARLY
CAS-C	DM6	8;30998973;C;T	0.47	missense variant	WRN	136658	probably damaging (0.999)	deleterious (0)	ENSP00000298139.5:p.Arg999Cys	EARLY
CAS-C	P	8;30998973;C;T	0.50	missense variant	WRN	136658	probably damaging (0.999)	deleterious (0)	ENSP00000298139.5:p.Arg999Cys	EARLY
ETH-E	DM1	7;140453136;A;T	0.50	missense variant	BRAF	476	possibly damaging (0.792)	deleterious (0)	ENSP00000288602.6:p.Val600Glu	EARLY
ETH-E	DM2	7;140453136;A;T	0.50	missense variant	BRAF	476	possibly damaging (0.792)	deleterious (0)	ENSP00000288602.6:p.Val600Glu	EARLY
ETH-E	DM3	7;140453136;A;T	0.50	missense variant	BRAF	476	possibly damaging (0.792)	deleterious (0)	ENSP00000288602.6:p.Val600Glu	EARLY

ETH-E	P	7;140453136;A;T	0.48	missense variant	BRAF	476	possibly damaging (0.792)	deleterious (0)	ENSP00000288602.6:p.Val600Glu	EARLY
ETH-E	RM	7;140453136;A;T	0.47	missense variant	BRAF	476	possibly damaging (0.792)	deleterious (0)	ENSP00000288602.6:p.Val600Glu	EARLY
ETH-E	DM1	16;9857727;G;A	0.25	missense variant	GRIN2A	.	benign (0.174)	deleterious (0.01)	ENSP00000454998.1:p.Ser1225Leu	EARLY
ETH-E	DM2	16;9857727;G;A	0.50	missense variant	GRIN2A	.	benign (0.174)	deleterious (0.01)	ENSP00000454998.1:p.Ser1225Leu	EARLY
ETH-E	DM3	16;9857727;G;A	0.39	missense variant	GRIN2A	.	benign (0.174)	deleterious (0.01)	ENSP00000454998.1:p.Ser1225Leu	EARLY
ETH-E	P	16;9857727;G;A	0.47	missense variant	GRIN2A	.	benign (0.174)	deleterious (0.01)	ENSP00000454998.1:p.Ser1225Leu	EARLY
ETH-E	DM1	11;118379868;C;T	0.24	missense variant	KMT2A	.	benign (0.121)	tolerated (0.06)	ENSP00000436786.1:p.Pro3618Leu	LATE
ETH-E	DM1	22;21343965;GGAGGAGGTGAGGGGCGTGGGGAGCCAGGGCGCAGGTA;G	0.26	splice donor variant	LZTR1	.	.	.	.	EARLY
ETH-E	DM1	22;21348438;G;A	0.96	missense variant	LZTR1	.	benign (0.308)	tolerated (0.13)	ENSP00000374006.3:p.Val480Met	EARLY
ETH-E	DM2	22;21348438;G;A	1.00	missense variant	LZTR1	.	benign (0.308)	tolerated (0.13)	ENSP00000374006.3:p.Val480Met	EARLY
ETH-E	DM3	22;21348438;G;A	1.00	missense variant	LZTR1	.	benign (0.308)	tolerated (0.13)	ENSP00000374006.3:p.Val480Met	EARLY
ETH-E	P	22;21348438;G;A	0.16	missense variant	LZTR1	.	benign (0.308)	tolerated (0.13)	ENSP00000374006.3:p.Val480Met	EARLY
ETH-E	P	22;21343965;GGAGGAGGTGAGGGGCGTGGGGAGCCAGGGCGCAGGTA;G	0.18	splice donor variant	LZTR1	.	.	.	.	EARLY
ETH-E	RM	22;21348438;G;A	0.39	missense variant	LZTR1	.	benign (0.308)	tolerated (0.13)	ENSP00000374006.3:p.Val480Met	EARLY
ETH-E	DM1	15;88476397;G;T	0.50	missense variant	NTRK3	.	probably damaging (0.968)	tolerated (0.27)	ENSP00000354207.2:p.Leu579Met	EARLY
ETH-E	DM2	15;88476397;G;T	0.50	missense variant	NTRK3	.	probably damaging (0.968)	tolerated (0.27)	ENSP00000354207.2:p.Leu579Met	EARLY
ETH-E	DM3	15;88476397;G;T	0.50	missense variant	NTRK3	.	probably damaging (0.968)	tolerated (0.27)	ENSP00000354207.2:p.Leu579Met	EARLY
ETH-E	P	15;88476397;G;T	0.02	missense variant	NTRK3	.	probably damaging (0.968)	tolerated (0.27)	ENSP00000354207.2:p.Leu579Met	EARLY
ETH-E	RM	15;88476397;G;T	0.44	missense variant	NTRK3	.	probably damaging (0.968)	tolerated (0.27)	ENSP00000354207.2:p.Leu579Met	EARLY
ETH-E	DM1	6;84270606;G;T	0.66	missense variant	SNAP91	.	possibly damaging (0.824)	tolerated (0.33)	ENSP00000428215.1:p.Gln835Lys	LATE
ETH-E	DM2	6;84270606;G;T	0.36	missense variant	SNAP91	.	possibly damaging (0.824)	tolerated (0.33)	ENSP00000428215.1:p.Gln835Lys	LATE
ETH-E	DM3	6;84270606;G;T	0.37	missense variant	SNAP91	.	possibly damaging (0.824)	tolerated (0.33)	ENSP00000428215.1:p.Gln835Lys	LATE
ETH-F	P3	17;16089896;G;A	0.27	missense variant	NCOR1	.	probably damaging (0.997)	deleterious (0)	ENSP00000268712.2:p.Leu72Phe	EARLY
ETH-J	DM1	4;126239655;C;G	0.49	missense variant	FAT4	.	benign (0.309)	tolerated (0.35)	ENSP00000377862.3:p.His697Asp	EARLY
ETH-J	DM2	4;126239655;C;G	0.35	missense variant	FAT4	.	benign (0.309)	tolerated (0.35)	ENSP00000377862.3:p.His697Asp	EARLY
ETH-J	DM3	4;126239655;C;G	0.29	missense variant	FAT4	.	benign (0.309)	tolerated (0.35)	ENSP00000377862.3:p.His697Asp	EARLY
ETH-J	P2	4;126239655;C;G	0.01	missense variant	FAT4	.	benign (0.309)	tolerated (0.35)	ENSP00000377862.3:p.His697Asp	EARLY
ETH-J	RM	4;126239655;C;G	0.33	missense variant	FAT4	.	benign (0.309)	tolerated (0.35)	ENSP00000377862.3:p.His697Asp	EARLY
ETH-J	DM1	1;115256529;T;C	0.92	missense variant	NRAS	584	benign (0.006)	deleterious (0.03)	ENSP00000358548.4:p.Gln61Arg	EARLY
ETH-J	DM2	1;115256529;T;C	0.98	missense variant	NRAS	584	benign (0.006)	deleterious (0.03)	ENSP00000358548.4:p.Gln61Arg	EARLY
ETH-J	DM3	1;115256529;T;C	0.96	missense variant	NRAS	584	benign (0.006)	deleterious (0.03)	ENSP00000358548.4:p.Gln61Arg	EARLY
ETH-J	P1	1;115256529;T;C	0.91	missense variant	NRAS	584	benign (0.006)	deleterious (0.03)	ENSP00000358548.4:p.Gln61Arg	EARLY
ETH-J	P2	1;115256529;T;C	1.00	missense variant	NRAS	584	benign (0.006)	deleterious (0.03)	ENSP00000358548.4:p.Gln61Arg	EARLY
ETH-J	RM	1;115256529;T;C	0.98	missense variant	NRAS	584	benign (0.006)	deleterious (0.03)	ENSP00000358548.4:p.Gln61Arg	EARLY
MI-F	DM	5;112154970;G;T	0.39	missense variant	APC	.	possibly damaging (0.762)	deleterious (0.02)	ENSP00000423828.2:p.Arg414Leu	EARLY
MI-F	DM	5;112170771;C;T	0.33	missense variant	APC	.	possibly damaging (0.874)	deleterious (0.01)	ENSP00000473355.1:p.Arg186Trp	EARLY
MI-F	P	5;112154970;G;T	0.33	missense variant	APC	.	possibly damaging (0.762)	deleterious (0.02)	ENSP00000423828.2:p.Arg414Leu	EARLY

MI-F	P	5;112170771;C;T	0.50	missense variant	APC	.	possibly damaging (0.874)	deleterious (0.01)	ENSP00000473355.1:p.Arg186Trp	EARLY
MI-F	RM1	5;112170771;C;T	0.43	missense variant	APC	.	possibly damaging (0.874)	deleterious (0.01)	ENSP00000473355.1:p.Arg186Trp	EARLY
MI-F	RM1	5;112154970;G;T	0.48	missense variant	APC	.	possibly damaging (0.762)	deleterious (0.02)	ENSP00000423828.2:p.Arg414Leu	EARLY
MI-F	RM2	5;112170771;C;T	0.47	missense variant	APC	.	possibly damaging (0.874)	deleterious (0.01)	ENSP00000473355.1:p.Arg186Trp	EARLY
MI-F	RM2	5;112154970;G;T	0.35	missense variant	APC	.	possibly damaging (0.762)	deleterious (0.02)	ENSP00000423828.2:p.Arg414Leu	EARLY
MI-F	DM	11;120347394;C;T	0.65	missense variant	ARHGEF12	.	probably damaging (0.995)	tolerated (0.19)	ENSP00000349056.3:p.Ser1082Phe	EARLY
MI-F	P	11;120347394;C;T	0.65	missense variant	ARHGEF12	.	probably damaging (0.995)	tolerated (0.19)	ENSP00000349056.3:p.Ser1082Phe	EARLY
MI-F	RM1	11;120347394;C;T	0.50	missense variant	ARHGEF12	.	probably damaging (0.995)	tolerated (0.19)	ENSP00000349056.3:p.Ser1082Phe	EARLY
MI-F	RM2	11;120347394;C;T	0.54	missense variant	ARHGEF12	.	probably damaging (0.995)	tolerated (0.19)	ENSP00000349056.3:p.Ser1082Phe	EARLY
MI-F	DM	6;157495190;C;T	0.31	missense variant	ARID1B	.	benign (0.407)	tolerated (0.05)	ENSP00000275248.4:p.Pro954Leu	EARLY
MI-F	P	6;157495190;C;T	0.44	missense variant	ARID1B	.	benign (0.407)	tolerated (0.05)	ENSP00000275248.4:p.Pro954Leu	EARLY
MI-F	RM1	6;157495190;C;T	0.46	missense variant	ARID1B	.	benign (0.407)	tolerated (0.05)	ENSP00000275248.4:p.Pro954Leu	EARLY
MI-F	RM2	6;157495190;C;T	0.37	missense variant	ARID1B	.	benign (0.407)	tolerated (0.05)	ENSP00000275248.4:p.Pro954Leu	EARLY
MI-F	DM	12;46230632;G;T	0.67	missense variant	ARID2	1183572	probably damaging (0.996)	deleterious (0)	ENSP00000415650.1:p.Arg145Ile	EARLY
MI-F	DM	12;46230641;C;T	0.33	missense variant	ARID2	1167991	probably damaging (0.997)	deleterious (0)	ENSP00000335044.6:p.Ser297Phe	EARLY
MI-F	P	12;46230632;G;T	0.64	missense variant	ARID2	1183572	probably damaging (0.996)	deleterious (0)	ENSP00000415650.1:p.Arg145Ile	EARLY
MI-F	P	12;46230641;C;T	0.30	missense variant	ARID2	1167991	probably damaging (0.997)	deleterious (0)	ENSP00000335044.6:p.Ser297Phe	EARLY
MI-F	RM1	12;46230641;C;T	0.33	missense variant	ARID2	1167991	probably damaging (0.997)	deleterious (0)	ENSP00000335044.6:p.Ser297Phe	EARLY
MI-F	RM1	12;46230632;G;T	0.65	missense variant	ARID2	1183572	probably damaging (0.996)	deleterious (0)	ENSP00000415650.1:p.Arg145Ile	EARLY
MI-F	RM2	12;46230632;G;T	0.66	missense variant	ARID2	1183572	probably damaging (0.996)	deleterious (0)	ENSP00000415650.1:p.Arg145Ile	EARLY
MI-F	RM2	12;46230641;C;T	0.30	missense variant	ARID2	1167991	probably damaging (0.997)	deleterious (0)	ENSP00000335044.6:p.Ser297Phe	EARLY
MI-F	DM	22;19210287;G;A	0.44	missense variant	CLTCL1	182039	probably damaging (0.965)	deleterious (0)	ENSP00000439662.1:p.Arg780Cys	EARLY
MI-F	P	22;19210287;G;A	0.50	missense variant	CLTCL1	182039	probably damaging (0.965)	deleterious (0)	ENSP00000439662.1:p.Arg780Cys	EARLY
MI-F	RM1	22;19210287;G;A	0.43	missense variant	CLTCL1	182039	probably damaging (0.965)	deleterious (0)	ENSP00000439662.1:p.Arg780Cys	EARLY
MI-F	RM2	22;19210287;G;A	0.50	missense variant	CLTCL1	182039	probably damaging (0.965)	deleterious (0)	ENSP00000439662.1:p.Arg780Cys	EARLY
MI-F	DM	12;48391466;C;T	0.43	missense variant	COL2A1	.	unknown (0)	tolerated (0.1)	ENSP00000338213.6:p.Asp83Asn	EARLY
MI-F	DM	12;48378831;C;T	0.65	missense variant	COL2A1	.	probably damaging (1)	deleterious (0)	ENSP00000338213.6:p.Gly525Arg	EARLY
MI-F	P	12;48391466;C;T	0.26	missense variant	COL2A1	.	unknown (0)	tolerated (0.1)	ENSP00000338213.6:p.Asp83Asn	EARLY
MI-F	P	12;48378831;C;T	0.67	missense variant	COL2A1	.	probably damaging (1)	deleterious (0)	ENSP00000338213.6:p.Gly525Arg	EARLY
MI-F	RM1	12;48378831;C;T	0.67	missense variant	COL2A1	.	probably damaging (1)	deleterious (0)	ENSP00000338213.6:p.Gly525Arg	EARLY
MI-F	RM1	12;48391466;C;T	0.34	missense variant	COL2A1	.	unknown (0)	tolerated (0.1)	ENSP00000338213.6:p.Asp83Asn	EARLY
MI-F	RM2	12;48378831;C;T	0.67	missense variant	COL2A1	.	probably damaging (1)	deleterious (0)	ENSP00000338213.6:p.Gly525Arg	EARLY
MI-F	RM2	12;48391466;C;T	0.32	missense variant	COL2A1	.	unknown (0)	tolerated (0.1)	ENSP00000338213.6:p.Asp83Asn	EARLY
MI-F	DM	1;36932277;C;T	0.36	missense variant	CSF3R	.	benign (0.083)	tolerated (0.2)	ENSP00000355406.4:p.Gly731Glu	EARLY
MI-F	P	1;36932277;C;T	0.50	missense variant	CSF3R	.	benign (0.083)	tolerated (0.2)	ENSP00000355406.4:p.Gly731Glu	EARLY
MI-F	RM1	1;36932277;C;T	0.44	missense variant	CSF3R	.	benign (0.083)	tolerated (0.2)	ENSP00000355406.4:p.Gly731Glu	EARLY
MI-F	RM2	1;36932277;C;T	0.50	missense variant	CSF3R	.	benign (0.083)	tolerated (0.2)	ENSP00000355406.4:p.Gly731Glu	EARLY

MI-F	DM	4;126372579;C;T	0.88	stop gained	FAT4	.	.	.	ENSP00000335169.5:p.Arg1768Ter	EARLY
MI-F	P	4;126372579;C;T	1.00	stop gained	FAT4	.	.	.	ENSP00000335169.5:p.Arg1768Ter	EARLY
MI-F	RM1	4;126372579;C;T	0.79	stop gained	FAT4	.	.	.	ENSP00000335169.5:p.Arg1768Ter	EARLY
MI-F	RM2	4;126372579;C;T	0.95	stop gained	FAT4	.	.	.	ENSP00000335169.5:p.Arg1768Ter	EARLY
MI-F	DM	16;9857500;C;T	0.37	missense variant	GRIN2A	.	benign (0.345)	deleterious (0.01)	ENSP00000379820.2:p.Glu1301Lys	EARLY
MI-F	DM	16;9923507;G;A	0.50	missense variant	GRIN2A	.	probably damaging (0.997)	tolerated (0.12)	ENSP00000454998.1:p.Pro594Ser	EARLY
MI-F	DM	16;10273929;C;T	0.50	missense variant	GRIN2A	.	probably damaging (0.996)	deleterious (0.03)	ENSP00000454998.1:p.Asp114Asn	EARLY
MI-F	DM	16;9857502;C;T	0.47	missense variant	GRIN2A	.	benign (0.078)	tolerated (1)	ENSP00000379820.2:p.Arg1300Lys	EARLY
MI-F	P	16;9857502;C;T	0.50	missense variant	GRIN2A	.	benign (0.078)	tolerated (1)	ENSP00000379820.2:p.Arg1300Lys	EARLY
MI-F	P	16;10273929;C;T	0.45	missense variant	GRIN2A	.	probably damaging (0.996)	deleterious (0.03)	ENSP00000454998.1:p.Asp114Asn	EARLY
MI-F	P	16;9857500;C;T	0.38	missense variant	GRIN2A	.	benign (0.345)	deleterious (0.01)	ENSP00000379820.2:p.Glu1301Lys	EARLY
MI-F	P	16;9923507;G;A	0.47	missense variant	GRIN2A	.	probably damaging (0.997)	tolerated (0.12)	ENSP00000454998.1:p.Pro594Ser	EARLY
MI-F	RM1	16;9857502;C;T	0.45	missense variant	GRIN2A	.	benign (0.078)	tolerated (1)	ENSP00000379820.2:p.Arg1300Lys	EARLY
MI-F	RM1	16;9923507;G;A	0.41	missense variant	GRIN2A	.	probably damaging (0.997)	tolerated (0.12)	ENSP00000454998.1:p.Pro594Ser	EARLY
MI-F	RM1	16;10273929;C;T	0.40	missense variant	GRIN2A	.	probably damaging (0.996)	deleterious (0.03)	ENSP00000454998.1:p.Asp114Asn	EARLY
MI-F	RM1	16;9857500;C;T	0.37	missense variant	GRIN2A	.	benign (0.345)	deleterious (0.01)	ENSP00000379820.2:p.Glu1301Lys	EARLY
MI-F	RM2	16;9857502;C;T	0.37	missense variant	GRIN2A	.	benign (0.078)	tolerated (1)	ENSP00000379820.2:p.Arg1300Lys	EARLY
MI-F	RM2	16;9923507;G;A	0.48	missense variant	GRIN2A	.	probably damaging (0.997)	tolerated (0.12)	ENSP00000454998.1:p.Pro594Ser	EARLY
MI-F	RM2	16;9857500;C;T	0.50	missense variant	GRIN2A	.	benign (0.345)	deleterious (0.01)	ENSP00000379820.2:p.Glu1301Lys	EARLY
MI-F	RM2	16;10273929;C;T	0.50	missense variant	GRIN2A	.	probably damaging (0.996)	deleterious (0.03)	ENSP00000454998.1:p.Asp114Asn	EARLY
MI-F	DM	7;50367333;C;T	0.27	missense variant	IKZF1	.	benign (0.005)	deleterious (0.04)	ENSP00000413025.1:p.Ser47Phe	EARLY
MI-F	DM	7;50444407;C;T	0.39	missense variant	IKZF1	.	possibly damaging (0.905)	tolerated (0.06)	ENSP00000413025.1:p.Pro113Ser	EARLY
MI-F	DM	7;50459516;A;G	0.53	missense variant	IKZF1	.	benign (0.004)	tolerated (0.11)	ENSP00000413025.1:p.Ser227Gly	EARLY
MI-F	P	7;50459516;A;G	0.57	missense variant	IKZF1	.	benign (0.004)	tolerated (0.11)	ENSP00000413025.1:p.Ser227Gly	EARLY
MI-F	P	7;50367333;C;T	0.39	missense variant	IKZF1	.	benign (0.005)	deleterious (0.04)	ENSP00000413025.1:p.Ser47Phe	EARLY
MI-F	P	7;50444407;C;T	0.43	missense variant	IKZF1	.	possibly damaging (0.905)	tolerated (0.06)	ENSP00000413025.1:p.Pro113Ser	EARLY
MI-F	RM1	7;50367333;C;T	0.27	missense variant	IKZF1	.	benign (0.005)	deleterious (0.04)	ENSP00000413025.1:p.Ser47Phe	EARLY
MI-F	RM1	7;50444407;C;T	0.48	missense variant	IKZF1	.	possibly damaging (0.905)	tolerated (0.06)	ENSP00000413025.1:p.Pro113Ser	EARLY
MI-F	RM1	7;50459516;A;G	0.59	missense variant	IKZF1	.	benign (0.004)	tolerated (0.11)	ENSP00000413025.1:p.Ser227Gly	EARLY
MI-F	RM2	7;50444407;C;T	0.50	missense variant	IKZF1	.	possibly damaging (0.905)	tolerated (0.06)	ENSP00000413025.1:p.Pro113Ser	EARLY
MI-F	RM2	7;50459516;A;G	0.59	missense variant	IKZF1	.	benign (0.004)	tolerated (0.11)	ENSP00000413025.1:p.Ser227Gly	EARLY
MI-F	RM2	7;50367333;C;T	0.35	missense variant	IKZF1	.	benign (0.005)	deleterious (0.04)	ENSP00000413025.1:p.Ser47Phe	EARLY
MI-F	DM	4;55955063;C;T	0.98	missense variant	KDR	.	probably damaging (0.997)	deleterious (0)	ENSP00000263923.4:p.Gly1161Glu	EARLY
MI-F	P	4;55955063;C;T	1.00	missense variant	KDR	.	probably damaging (0.997)	deleterious (0)	ENSP00000263923.4:p.Gly1161Glu	EARLY
MI-F	RM1	4;55955063;C;T	0.73	missense variant	KDR	.	probably damaging (0.997)	deleterious (0)	ENSP00000263923.4:p.Gly1161Glu	EARLY
MI-F	RM2	4;55955063;C;T	0.98	missense variant	KDR	.	probably damaging (0.997)	deleterious (0)	ENSP00000263923.4:p.Gly1161Glu	EARLY
MI-F	DM	11;118360549;G;A	0.28	missense variant	KMT2A	.	probably damaging (0.996)	tolerated (0.13)	ENSP00000376612.3:p.Glu220Lys	EARLY
MI-F	DM	11;118390431;C;T	0.32	missense variant	KMT2A	.	probably damaging (0.999)	deleterious (0)	ENSP00000374157.5:p.Arg3746Cys	EARLY

MI-F	P	11;118390431;C;T	0.34	missense variant	KMT2A	.	probably damaging (0.999)	deleterious (0)	ENSP00000374157.5:p.Arg3746Cys	EARLY
MI-F	P	11;118360549;G;A	0.31	missense variant	KMT2A	.	probably damaging (0.996)	tolerated (0.13)	ENSP00000376612.3:p.Glu220Lys	EARLY
MI-F	RM1	11;118390431;C;T	0.39	missense variant	KMT2A	.	probably damaging (0.999)	deleterious (0)	ENSP00000374157.5:p.Arg3746Cys	EARLY
MI-F	RM1	11;118360549;G;A	0.27	missense variant	KMT2A	.	probably damaging (0.996)	tolerated (0.13)	ENSP00000376612.3:p.Glu220Lys	EARLY
MI-F	RM2	11;118360549;G;A	0.29	missense variant	KMT2A	.	probably damaging (0.996)	tolerated (0.13)	ENSP00000376612.3:p.Glu220Lys	EARLY
MI-F	RM2	11;118390431;C;T	0.36	missense variant	KMT2A	.	probably damaging (0.999)	deleterious (0)	ENSP00000374157.5:p.Arg3746Cys	EARLY
MI-F	DM	12;49432428;G;A	0.31	missense variant	KMT2D	.	possibly damaging (0.468)	.	ENSP00000301067.7:p.Pro2904Leu	EARLY
MI-F	P	12;49432428;G;A	0.33	missense variant	KMT2D	.	possibly damaging (0.468)	.	ENSP00000301067.7:p.Pro2904Leu	EARLY
MI-F	RM1	12;49432428;G;A	0.29	missense variant	KMT2D	.	possibly damaging (0.468)	.	ENSP00000301067.7:p.Pro2904Leu	EARLY
MI-F	RM2	12;49432428;G;A	0.39	missense variant	KMT2D	.	possibly damaging (0.468)	.	ENSP00000301067.7:p.Pro2904Leu	EARLY
MI-F	DM	22;21344778;C;T	0.43	missense variant	LZTR1	.	probably damaging (0.982)	deleterious (0.02)	ENSP00000374006.3:p.Thr233Ile	EARLY
MI-F	P	22;21344778;C;T	0.44	missense variant	LZTR1	.	probably damaging (0.982)	deleterious (0.02)	ENSP00000374006.3:p.Thr233Ile	EARLY
MI-F	RM1	22;21344778;C;T	0.44	missense variant	LZTR1	.	probably damaging (0.982)	deleterious (0.02)	ENSP00000374006.3:p.Thr233Ile	EARLY
MI-F	RM2	22;21344778;C;T	0.37	missense variant	LZTR1	.	probably damaging (0.982)	deleterious (0.02)	ENSP00000374006.3:p.Thr233Ile	EARLY
MI-F	DM	12;124846825;C;A	0.50	stop gained	NCOR2	.	.	.	ENSP00000400281.2:p.Glu965Ter	EARLY
MI-F	P	12;124846825;C;A	0.39	stop gained	NCOR2	.	.	.	ENSP00000400281.2:p.Glu965Ter	EARLY
MI-F	RM1	12;124846825;C;A	0.29	stop gained	NCOR2	.	.	.	ENSP00000400281.2:p.Glu965Ter	EARLY
MI-F	RM2	12;124846825;C;A	0.47	stop gained	NCOR2	.	.	.	ENSP00000400281.2:p.Glu965Ter	EARLY
MI-F	DM	5;176721004;C;T	0.35	missense variant	NSD1	.	probably damaging (0.996)	deleterious (0)	ENSP00000343209.4:p.Pro1943Leu	EARLY
MI-F	P	5;176721004;C;T	0.31	missense variant	NSD1	.	probably damaging (0.996)	deleterious (0)	ENSP00000343209.4:p.Pro1943Leu	EARLY
MI-F	RM1	5;176721004;C;T	0.50	missense variant	NSD1	.	probably damaging (0.996)	deleterious (0)	ENSP00000343209.4:p.Pro1943Leu	EARLY
MI-F	RM2	5;176721004;C;T	0.34	missense variant	NSD1	.	probably damaging (0.996)	deleterious (0)	ENSP00000343209.4:p.Pro1943Leu	EARLY
MI-F	DM	15;88690570;C;T	0.15	missense variant	NTRK3	.	benign (0.297)	tolerated (0.16)	ENSP00000354207.2:p.Glu154Lys	EARLY
MI-F	P	15;88690570;C;T	0.50	missense variant	NTRK3	.	benign (0.297)	tolerated (0.16)	ENSP00000354207.2:p.Glu154Lys	EARLY
MI-F	RM1	15;88690570;C;T	0.44	missense variant	NTRK3	.	benign (0.297)	tolerated (0.16)	ENSP00000354207.2:p.Glu154Lys	EARLY
MI-F	RM2	15;88690570;C;T	0.32	missense variant	NTRK3	.	benign (0.297)	tolerated (0.16)	ENSP00000354207.2:p.Glu154Lys	EARLY
MI-F	DM	11;71732301;G;A	0.50	missense variant	NUMA1	.	benign (0.273)	deleterious (0.02)	ENSP00000444175.1:p.His137Tyr	EARLY
MI-F	P	11;71732301;G;A	0.42	missense variant	NUMA1	.	benign (0.273)	deleterious (0.02)	ENSP00000444175.1:p.His137Tyr	EARLY
MI-F	RM1	11;71732301;G;A	0.43	missense variant	NUMA1	.	benign (0.273)	deleterious (0.02)	ENSP00000444175.1:p.His137Tyr	EARLY
MI-F	RM2	11;71732301;G;A	0.50	missense variant	NUMA1	.	benign (0.273)	deleterious (0.02)	ENSP00000444175.1:p.His137Tyr	EARLY
MI-F	DM	6;106554931;G;T	0.34	missense variant	PRDM1	.	probably damaging (0.972)	tolerated (0.05)	ENSP00000358085.3:p.Arg549Leu	EARLY
MI-F	DM	6;106553656;C;T	0.50	missense variant	PRDM1	.	benign (0.004)	tolerated (0.68)	ENSP00000358085.3:p.Pro407Ser	EARLY
MI-F	P	6;106553656;C;T	0.45	missense variant	PRDM1	.	benign (0.004)	tolerated (0.68)	ENSP00000358085.3:p.Pro407Ser	EARLY
MI-F	P	6;106554931;G;T	0.44	missense variant	PRDM1	.	probably damaging (0.972)	tolerated (0.05)	ENSP00000358085.3:p.Arg549Leu	EARLY
MI-F	RM1	6;106554931;G;T	0.49	missense variant	PRDM1	.	probably damaging (0.972)	tolerated (0.05)	ENSP00000358085.3:p.Arg549Leu	EARLY
MI-F	RM1	6;106553656;C;T	0.49	missense variant	PRDM1	.	benign (0.004)	tolerated (0.68)	ENSP00000358085.3:p.Pro407Ser	EARLY
MI-F	RM2	6;106553656;C;T	0.49	missense variant	PRDM1	.	benign (0.004)	tolerated (0.68)	ENSP00000358085.3:p.Pro407Ser	EARLY

MI-F	RM2	6;106554931;G;T	0.50	missense variant	PRDM 1	.	probably damaging (0.972)	tolerated (0.05)	ENSP00000358085. 3:p.Arg549Leu	EARLY
MI-F	DM	12;70934719;A;T	0.32	missense variant	PTPRB	.	probably damaging (0.937)	deleterious (0.01)	ENSP00000447302. 1:p.Ile1530Asn	EARLY
MI-F	DM	12;71029678;G;A	0.61	missense variant	PTPRB	.	benign (0.004)	deleterious (0.02)	ENSP00000448349. 1:p.Ser74Phe	EARLY
MI-F	DM	12;70974905;G;A	0.67	missense variant	PTPRB	.	benign (0.017)	tolerated (0.19)	ENSP00000447302. 1:p.Ser522Phe	EARLY
MI-F	P	12;71029678;G;A	0.67	missense variant	PTPRB	.	benign (0.004)	deleterious (0.02)	ENSP00000448349. 1:p.Ser74Phe	EARLY
MI-F	P	12;70974905;G;A	0.67	missense variant	PTPRB	.	benign (0.017)	tolerated (0.19)	ENSP00000447302. 1:p.Ser522Phe	EARLY
MI-F	P	12;70934719;A;T	0.30	missense variant	PTPRB	.	probably damaging (0.937)	deleterious (0.01)	ENSP00000447302. 1:p.Ile1530Asn	EARLY
MI-F	RM1	12;71029678;G;A	0.59	missense variant	PTPRB	.	benign (0.004)	deleterious (0.02)	ENSP00000448349. 1:p.Ser74Phe	EARLY
MI-F	RM1	12;70974905;G;A	0.61	missense variant	PTPRB	.	benign (0.017)	tolerated (0.19)	ENSP00000447302. 1:p.Ser522Phe	EARLY
MI-F	RM1	12;70934719;A;T	0.23	missense variant	PTPRB	.	probably damaging (0.937)	deleterious (0.01)	ENSP00000447302. 1:p.Ile1530Asn	EARLY
MI-F	RM2	12;70974905;G;A	0.61	missense variant	PTPRB	.	benign (0.017)	tolerated (0.19)	ENSP00000447302. 1:p.Ser522Phe	EARLY
MI-F	RM2	12;70934719;A;T	0.37	missense variant	PTPRB	.	probably damaging (0.937)	deleterious (0.01)	ENSP00000447302. 1:p.Ile1530Asn	EARLY
MI-F	RM2	12;71029678;G;A	0.67	missense variant	PTPRB	.	benign (0.004)	deleterious (0.02)	ENSP00000448349. 1:p.Ser74Phe	EARLY
MI-F	DM	1;198721429;G;A	0.50	missense variant	PTPRC	.	probably damaging (0.954)	deleterious (0.04)	ENSP00000411355. 3:p.Glu1087Lys	EARLY
MI-F	P	1;198721429;G;A	0.44	missense variant	PTPRC	.	probably damaging (0.954)	deleterious (0.04)	ENSP00000411355. 3:p.Glu1087Lys	EARLY
MI-F	RM1	1;198721429;G;A	0.50	missense variant	PTPRC	.	probably damaging (0.954)	deleterious (0.04)	ENSP00000411355. 3:p.Glu1087Lys	EARLY
MI-F	RM2	1;198721429;G;A	0.50	missense variant	PTPRC	.	probably damaging (0.954)	deleterious (0.04)	ENSP00000411355. 3:p.Glu1087Lys	EARLY
MI-F	DM	6;117663609;T;A	0.45	missense variant	ROS1	.	benign (0.41)	tolerated (0.07)	ENSP00000357494. 3:p.Glu1541Asp	EARLY
MI-F	DM	6;117687278;G;A	0.50	stop gained	ROS1	.	.	.	ENSP00000357494. 3:p.Gln925Ter	EARLY
MI-F	DM	6;117708109;G;A	0.50	missense variant	ROS1	.	benign (0.295)	tolerated (0.45)	ENSP00000357494. 3:p.Pro690Ser	EARLY
MI-F	P	6;117663609;T;A	0.46	missense variant	ROS1	.	benign (0.41)	tolerated (0.07)	ENSP00000357494. 3:p.Glu1541Asp	EARLY
MI-F	P	6;117687278;G;A	0.43	stop gained	ROS1	.	.	.	ENSP00000357494. 3:p.Gln925Ter	EARLY
MI-F	P	6;117708109;G;A	0.47	missense variant	ROS1	.	benign (0.295)	tolerated (0.45)	ENSP00000357494. 3:p.Pro690Ser	EARLY
MI-F	RM1	6;117687278;G;A	0.50	stop gained	ROS1	.	.	.	ENSP00000357494. 3:p.Gln925Ter	EARLY
MI-F	RM1	6;117663609;T;A	0.48	missense variant	ROS1	.	benign (0.41)	tolerated (0.07)	ENSP00000357494. 3:p.Glu1541Asp	EARLY
MI-F	RM1	6;117708109;G;A	0.50	missense variant	ROS1	.	benign (0.295)	tolerated (0.45)	ENSP00000357494. 3:p.Pro690Ser	EARLY
MI-F	RM2	6;117687278;G;A	0.50	stop gained	ROS1	.	.	.	ENSP00000357494. 3:p.Gln925Ter	EARLY
MI-F	RM2	6;117663609;T;A	0.50	missense variant	ROS1	.	benign (0.41)	tolerated (0.07)	ENSP00000357494. 3:p.Glu1541Asp	EARLY
MI-F	RM2	6;117708109;G;A	0.50	missense variant	ROS1	.	benign (0.295)	tolerated (0.45)	ENSP00000357494. 3:p.Pro690Ser	EARLY
MI-F	DM	3;128348869;G;A	0.41	missense variant	RPN1	.	probably damaging (0.999)	deleterious (0)	ENSP00000417529. 1:p.Pro149Ser	EARLY
MI-F	P	3;128348869;G;A	0.50	missense variant	RPN1	.	probably damaging (0.999)	deleterious (0)	ENSP00000417529. 1:p.Pro149Ser	EARLY
MI-F	RM1	3;128348869;G;A	0.50	missense variant	RPN1	.	probably damaging (0.999)	deleterious (0)	ENSP00000417529. 1:p.Pro149Ser	EARLY
MI-F	RM2	3;128348869;G;A	0.45	missense variant	RPN1	.	probably damaging (0.999)	deleterious (0)	ENSP00000417529. 1:p.Pro149Ser	EARLY
MI-F	DM	3;47163802;A;C	0.28	missense variant	SETD2	1423580,1423579	benign (0.001)	deleterious (0.01)	ENSP00000416401. 1:p.Val731Gly	EARLY
MI-F	P	3;47163802;A;C	0.48	missense variant	SETD2	1423580,1423579	benign (0.001)	deleterious (0.01)	ENSP00000416401. 1:p.Val731Gly	EARLY
MI-F	RM1	3;47163802;A;C	0.50	missense variant	SETD2	1423580,1423579	benign (0.001)	deleterious (0.01)	ENSP00000416401. 1:p.Val731Gly	EARLY
MI-F	RM2	3;47163802;A;C	0.42	missense variant	SETD2	1423580,1423579	benign (0.001)	deleterious (0.01)	ENSP00000416401. 1:p.Val731Gly	EARLY
MI-F	DM	16;11348713;G;A	0.50	missense variant	SOCS1	.	probably damaging (1)	deleterious (0)	ENSP00000329418. 2:p.Pro208Leu	EARLY
MI-F	P	16;11348713;G;A	0.50	missense variant	SOCS1	.	probably damaging (1)	deleterious (0)	ENSP00000329418. 2:p.Pro208Leu	EARLY

MI-F	RM1	16;11348713;G;A	0.50	missense variant	SOCS1	.	probably damaging (1)	deleterious (0)	ENSP00000329418.2:p.Pro208Leu	EARLY
MI-F	RM2	16;11348713;G;A	0.50	missense variant	SOCS1	.	probably damaging (1)	deleterious (0)	ENSP00000329418.2:p.Pro208Leu	EARLY
MI-F	DM	4;106197332;C;T	0.85	missense variant	TET2	.	probably damaging (1)	deleterious (0)	ENSP00000369351.4:p.Pro1889Ser	EARLY
MI-F	P	4;106197332;C;T	1.00	missense variant	TET2	.	probably damaging (1)	deleterious (0)	ENSP00000369351.4:p.Pro1889Ser	EARLY
MI-F	RM1	4;106197332;C;T	0.86	missense variant	TET2	.	probably damaging (1)	deleterious (0)	ENSP00000369351.4:p.Pro1889Ser	EARLY
MI-F	RM2	4;106197332;C;T	0.95	missense variant	TET2	.	probably damaging (1)	deleterious (0)	ENSP00000369351.4:p.Pro1889Ser	EARLY
MI-F	DM	17;7578517;G;A	0.50	missense variant	TP53	43818,288785,288784, 288786,288787	possibly damaging (0.661)	deleterious (0.03)	ENSP00000398846.2:p.Ala138Val	EARLY
MI-F	P	17;7578517;G;A	0.47	missense variant	TP53	43818,288785,288784, 288786,288787	possibly damaging (0.661)	deleterious (0.03)	ENSP00000398846.2:p.Ala138Val	EARLY
MI-F	RM1	17;7578517;G;A	0.37	missense variant	TP53	43818,288785,288784, 288786,288787	possibly damaging (0.661)	deleterious (0.03)	ENSP00000398846.2:p.Ala138Val	EARLY
MI-F	RM2	17;7578517;G;A	0.39	missense variant	TP53	43818,288785,288784, 288786,288787	possibly damaging (0.661)	deleterious (0.03)	ENSP00000398846.2:p.Ala138Val	EARLY
MI-F	DM	1;186304261;C;T	0.29	missense variant	TPR	.	.	tolerated (0.36)	ENSP00000356448.3:p.Val1638Ile	EARLY
MI-F	DM	1;186304262;C;T	0.28	splice acceptor variant	TPR	.	.	.	.	EARLY
MI-F	P	1;186304261;C;T	0.33	missense variant	TPR	.	.	tolerated (0.36)	ENSP00000356448.3:p.Val1638Ile	EARLY
MI-F	P	1;186304262;C;T	0.35	splice acceptor variant	TPR	.	.	.	.	EARLY
MI-F	RM1	1;186304261;C;T	0.41	missense variant	TPR	.	.	tolerated (0.36)	ENSP00000356448.3:p.Val1638Ile	EARLY
MI-F	RM1	1;186304262;C;T	0.41	splice acceptor variant	TPR	.	.	.	.	EARLY
MI-F	RM2	1;186304261;C;T	0.38	missense variant	TPR	.	.	tolerated (0.36)	ENSP00000356448.3:p.Val1638Ile	EARLY
MI-F	RM2	1;186304262;C;T	0.39	splice acceptor variant	TPR	.	.	.	.	EARLY
MI-F	DM	8;103326042;G;A	0.25	missense variant	UBR5	.	probably damaging (0.991)	deleterious (0)	ENSP00000427819.1:p.Pro660Leu	EARLY
MI-F	P	8;103326042;G;A	0.42	missense variant	UBR5	.	probably damaging (0.991)	deleterious (0)	ENSP00000427819.1:p.Pro660Leu	EARLY
MI-F	RM1	8;103326042;G;A	0.44	missense variant	UBR5	.	probably damaging (0.991)	deleterious (0)	ENSP00000427819.1:p.Pro660Leu	EARLY
MI-F	RM2	8;103326042;G;A	0.45	missense variant	UBR5	.	probably damaging (0.991)	deleterious (0)	ENSP00000427819.1:p.Pro660Leu	EARLY
MI-F	DM	8;30921942;C;T	0.67	missense variant	WRN	.	probably damaging (0.921)	deleterious (0)	ENSP00000298139.5:p.Ser116Phe	EARLY
MI-F	P	8;30921942;C;T	0.50	missense variant	WRN	.	probably damaging (0.921)	deleterious (0)	ENSP00000298139.5:p.Ser116Phe	EARLY
MI-F	RM1	8;30921942;C;T	0.50	missense variant	WRN	.	probably damaging (0.921)	deleterious (0)	ENSP00000298139.5:p.Ser116Phe	EARLY
MI-F	RM2	8;30921942;C;T	0.44	missense variant	WRN	.	probably damaging (0.921)	deleterious (0)	ENSP00000298139.5:p.Ser116Phe	EARLY
MI-F	DM	16;72822618;G;A	0.49	missense variant	ZFHX3	.	unknown (0)	.	ENSP00000268489.5:p.Ala3186Val	EARLY
MI-F	P	16;72822618;G;A	0.40	missense variant	ZFHX3	.	unknown (0)	.	ENSP00000268489.5:p.Ala3186Val	EARLY
MI-F	RM1	16;72822618;G;A	0.50	missense variant	ZFHX3	.	unknown (0)	.	ENSP00000268489.5:p.Ala3186Val	EARLY
MI-F	RM2	16;72822618;G;A	0.50	missense variant	ZFHX3	.	unknown (0)	.	ENSP00000268489.5:p.Ala3186Val	EARLY
SK-G	DM	11;120346199;T;C	1.00	missense variant	ARHGEF12	.	probably damaging (0.993)	deleterious (0)	ENSP00000349056.3:p.Val1068Ala	EARLY
SK-G	P	11;120346199;T;C	0.99	missense variant	ARHGEF12	.	probably damaging (0.993)	deleterious (0)	ENSP00000349056.3:p.Val1068Ala	EARLY
SK-G	RM	11;120346199;T;C	1.00	missense variant	ARHGEF12	.	probably damaging (0.993)	deleterious (0)	ENSP00000349056.3:p.Val1068Ala	EARLY
SK-G	DM	12;46254605;C;T	0.27	missense variant	ARID2	.	benign (0.002)	tolerated (0.39)	ENSP00000388357.1:p.Pro207Ser	EARLY
SK-G	P	12;46254605;C;T	0.50	missense variant	ARID2	.	benign (0.002)	tolerated (0.39)	ENSP00000388357.1:p.Pro207Ser	EARLY
SK-G	RM	12;46254605;C;T	0.42	missense variant	ARID2	.	benign (0.002)	tolerated (0.39)	ENSP00000388357.1:p.Pro207Ser	EARLY
SK-G	P	X;76854915;C;T	0.50	missense variant	ATRX	.	benign (0.027)	tolerated (1)	ENSP00000362441.4:p.Gly1974Glu	EARLY
SK-G	RM	X;76854915;C;T	0.43	missense variant	ATRX	.	benign (0.027)	tolerated (1)	ENSP00000362441.4:p.Gly1974Glu	EARLY
SK-G	DM	4;187629712;C;A	0.50	stop gained	FAT1	.	.	.	ENSP00000406229.2:p.Glu424Ter	EARLY

SK-G	P	4;187629712;C;A	0.49	stop gained	FAT1	.	.	.	ENSP00000406229.2:p.Glu424Ter	EARLY
SK-G	RM	4;187629712;C;A	0.45	stop gained	FAT1	.	.	.	ENSP00000406229.2:p.Glu424Ter	EARLY
SK-G	DM	5;180057098;G;A	0.38	missense variant	FLT4	1293142,1293141	benign (0.002)	tolerated (0.32)	ENSP00000377016.3:p.Ser174Leu	EARLY
SK-G	P	5;180057098;G;A	0.27	missense variant	FLT4	1293142,1293141	benign (0.002)	tolerated (0.32)	ENSP00000377016.3:p.Ser174Leu	EARLY
SK-G	RM	5;180057098;G;A	0.28	missense variant	FLT4	1293142,1293141	benign (0.002)	tolerated (0.32)	ENSP00000377016.3:p.Ser174Leu	EARLY
SK-G	RM	7;151878296;G;A	0.42	missense variant	KMT2C	.	benign (0.019)	tolerated (0.11)	ENSP00000262189.6:p.Pro2217Ser	LATE
SK-G	DM	17;15968264;G;A	0.39	missense variant	NCOR1	.	probably damaging (0.999)	deleterious (0.01)	ENSP00000268712.2:p.Pro1674Leu	EARLY
SK-G	DM	17;15968220;G;A	0.55	missense variant	NCOR1	.	probably damaging (0.998)	tolerated (0.52)	ENSP00000268712.2:p.Pro1689Ser	EARLY
SK-G	P	17;15968220;G;A	0.50	missense variant	NCOR1	.	probably damaging (0.998)	tolerated (0.52)	ENSP00000268712.2:p.Pro1689Ser	EARLY
SK-G	P	17;15968264;G;A	0.40	missense variant	NCOR1	.	probably damaging (0.999)	deleterious (0.01)	ENSP00000268712.2:p.Pro1674Leu	EARLY
SK-G	RM	17;15968220;G;A	1.00	missense variant	NCOR1	.	probably damaging (0.998)	tolerated (0.52)	ENSP00000268712.2:p.Pro1689Ser	EARLY
SK-G	DM	1;115256530;G;T	0.50	missense variant	NRAS	580	benign (0.045)	deleterious (0.01)	ENSP00000358548.4:p.Gln61Lys	EARLY
SK-G	P	1;115256530;G;T	0.45	missense variant	NRAS	580	benign (0.045)	deleterious (0.01)	ENSP00000358548.4:p.Gln61Lys	EARLY
SK-G	RM	1;115256530;G;T	0.50	missense variant	NRAS	580	benign (0.045)	deleterious (0.01)	ENSP00000358548.4:p.Gln61Lys	EARLY
SK-G	DM	15;88522637;T;C	0.61	missense variant	NTRK3	.	benign (0.001)	deleterious (0)	ENSP00000444673.2:p.Asn593Ser	EARLY
SK-G	P	15;88522637;T;C	0.50	missense variant	NTRK3	.	benign (0.001)	deleterious (0)	ENSP00000444673.2:p.Asn593Ser	EARLY
SK-G	RM	15;88522637;T;C	0.45	missense variant	NTRK3	.	benign (0.001)	deleterious (0)	ENSP00000444673.2:p.Asn593Ser	EARLY
SK-G	RM	3;178921549;T;G	0.50	missense variant	PIK3CA	22540,258749	probably damaging (0.987)	deleterious (0)	ENSP00000263967.3:p.Val344Gly	LATE
SK-G	DM	3;128344836;G;A	0.37	missense variant	RPN1	.	possibly damaging (0.831)	deleterious (0.03)	ENSP00000417529.1:p.Pro214Ser	EARLY
SK-G	P	3;128344836;G;A	0.41	missense variant	RPN1	.	possibly damaging (0.831)	deleterious (0.03)	ENSP00000417529.1:p.Pro214Ser	EARLY
SK-G	RM	3;128344836;G;A	0.49	missense variant	RPN1	.	possibly damaging (0.831)	deleterious (0.03)	ENSP00000417529.1:p.Pro214Ser	EARLY
SK-G	RM	10;70404675;T;C	0.05	missense variant	TET1	.	benign (0.156)	tolerated (0.23)	ENSP00000362748.4:p.Val730Ala	LATE
SK-G	DM	16;2125841;G;A	0.60	missense variant	TSC2	.	probably damaging (0.994)	deleterious (0.02)	ENSP00000371978.6:p.Glu814Lys	EARLY
SK-G	P	16;2125841;G;A	0.47	missense variant	TSC2	.	probably damaging (0.994)	deleterious (0.02)	ENSP00000371978.6:p.Glu814Lys	EARLY
SK-G	RM	16;2125841;G;A	0.65	missense variant	TSC2	.	probably damaging (0.994)	deleterious (0.02)	ENSP00000371978.6:p.Glu814Lys	EARLY
SK-G	DM	17;5042622;G;A	0.67	missense variant	USP6	.	probably damaging (0.969)	deleterious (0)	ENSP00000328010.4:p.Gly384Glu	EARLY
SK-G	P	17;5042622;G;A	0.50	missense variant	USP6	.	probably damaging (0.969)	deleterious (0)	ENSP00000328010.4:p.Gly384Glu	EARLY
SK-G	DM	16;72992883;C;T	0.50	missense variant	ZFHX3	1609649	benign (0.346)	.	ENSP00000268489.5:p.Glu388Lys	EARLY
SK-G	P	16;72992883;C;T	0.50	missense variant	ZFHX3	1609649	benign (0.346)	.	ENSP00000268489.5:p.Glu388Lys	EARLY
SK-G	RM	16;72992883;C;T	0.50	missense variant	ZFHX3	1609649	benign (0.346)	.	ENSP00000268489.5:p.Glu388Lys	EARLY
SK-H	RM2	5;112179165;G;A	0.48	missense variant	APC	.	benign (0.001)	tolerated (0.52)	ENSP00000427089.2:p.Ser2625Asn	LATE
SK-H	RM2	11;120310893;G;A	0.50	missense variant	ARHGEF12	.	probably damaging (1)	deleterious (0)	ENSP00000349056.3:p.Gly333Glu	LATE
SK-H	RM1	1;27099054;G;A	0.37	missense variant	ARID1A	.	possibly damaging (0.511)	tolerated (0.09)	ENSP00000390317.2:p.Gly55Glu	LATE
SK-H	RM1	6;157099854;G;A	0.10	missense variant	ARID1B	.	unknown (0)	deleterious (0.01)	ENSP00000275248.4:p.Gly206Glu	LATE
SK-H	DM	12;46230691;C;T	0.90	missense variant	ARID2	278971	probably damaging (0.997)	deleterious (0)	ENSP00000415650.1:p.Arg165Cys	EARLY
SK-H	P	12;46230691;C;T	0.50	missense variant	ARID2	278971	probably damaging (0.997)	deleterious (0)	ENSP00000415650.1:p.Arg165Cys	EARLY
SK-H	P	12;46230743;G;A	0.39	missense variant	ARID2	.	probably damaging (0.999)	deleterious (0)	ENSP00000415650.1:p.Gly182Asp	EARLY
SK-H	RM1	12;46287425;G;A	0.29	missense variant	ARID2	.	probably damaging (1)	deleterious (0)	ENSP00000388357.1:p.Gly370Arg	LATE

SK-H	RM1	12;46230743;G;A	0.67	missense variant	ARID2	.	probably damaging (0.999)	deleterious (0)	ENSP00000415650.1:p.Gly182Asp	EARLY
SK-H	RM1	12;46230691;C;T	0.42	missense variant	ARID2	278971	probably damaging (0.997)	deleterious (0)	ENSP00000415650.1:p.Arg165Cys	EARLY
SK-H	RM2	12;46230691;C;T	0.30	missense variant	ARID2	278971	probably damaging (0.997)	deleterious (0)	ENSP00000415650.1:p.Arg165Cys	EARLY
SK-H	RM2	12;46230743;G;A	0.65	missense variant	ARID2	.	probably damaging (0.999)	deleterious (0)	ENSP00000415650.1:p.Gly182Asp	EARLY
SK-H	RM1	20;31022265;G;A	0.11	missense variant	ASXL1	.	probably damaging (0.998)	deleterious (0)	ENSP00000305119.5:p.Val579Met	LATE
SK-H	RM1	20;31024038;G;A	0.09	missense variant	ASXL1	.	benign (0.159)	deleterious (0.05)	ENSP00000305119.5:p.Glu1170Lys	LATE
SK-H	RM1	X;76854962;C;T	0.37	stop gained	ATRX	.	.	.	ENSP00000362441.4:p.Trp1958Ter	LATE
SK-H	RM1	22;19263266;C;T	0.21	missense variant	CLTCL1	.	benign (0.019)	tolerated (0.24)	ENSP00000439662.1:p.Val44Ile	LATE
SK-H	RM1	1;36932118;G;A	0.17	missense variant	CSF3R	.	probably damaging (0.978)	deleterious (0)	ENSP00000355406.4:p.Pro784Leu	LATE
SK-H	RM1	14;95570395;G;A	0.25	missense variant	DICER1	.	benign (0.004)	tolerated (0.42)	ENSP00000376783.1:p.Ser1113Phe	LATE
SK-H	RM2	14;95577694;C;T	0.23	missense variant	DICER1	.	probably damaging (0.99)	tolerated (0.19)	ENSP00000435681.1:p.Arg739Lys	LATE
SK-H	RM2	14;95557560;G;A	0.19	missense variant	DICER1	.	possibly damaging (0.507)	tolerated (0.22)	ENSP00000376783.1:p.Pro1836Leu	LATE
SK-H	DM	19;10922962;C;T	0.21	missense variant	DNM2	.	benign (0.191)	tolerated (0.07)	ENSP00000373905.3:p.Thr527Ile	LATE
SK-H	RM1	7;55260507;A;G	0.17	missense variant	EGFR	.	probably damaging (0.946)	deleterious (0)	ENSP00000415559.1:p.Thr847Ala	LATE
SK-H	RM1	4;187539291;A;G	0.20	missense variant	FAT1	.	benign (0.098)	tolerated (0.27)	ENSP00000406229.2:p.Phe2817Leu	LATE
SK-H	RM2	4;187541076;C;T	0.32	missense variant	FAT1	.	possibly damaging (0.597)	tolerated (0.22)	ENSP00000406229.2:p.Asp2222Asn	LATE
SK-H	RM1	4;126408731;C;T	0.33	missense variant	FAT4	.	benign (0.015)	tolerated (0.5)	ENSP00000335169.5:p.Pro2591Ser	LATE
SK-H	RM2	4;126328255;G;A	0.24	missense variant	FAT4	.	benign (0.005)	tolerated (0.71)	ENSP00000335169.5:p.Arg141Lys	LATE
SK-H	DM	7;50367354;G;A	0.11	splice donor variant	IKZF1	.	.	.	.	LATE
SK-H	RM2	12;49443821;C;T	0.32	missense variant	KMT2D	.	benign (0.444)	.	ENSP00000301067.7:p.Glu1184Lys	LATE
SK-H	RM2	22;21340186;G;A	0.32	missense variant	LZTR1	.	benign (0.327)	tolerated (0.1)	ENSP00000397247.1:p.Arg107Lys	LATE
SK-H	RM2	22;21346659;G;A	0.26	splice donor variant	LZTR1	1665940	.	.	.	LATE
SK-H	RM2	16;15870007;G;A	0.19	missense variant	MYH11	.	possibly damaging (0.689)	deleterious (0)	ENSP00000379616.3:p.Arg280Cys	LATE
SK-H	DM	12;124826588;G;A	0.36	missense variant	NCOR2	.	possibly damaging (0.862)	deleterious (0.03)	ENSP00000380513.1:p.Pro1648Ser	LATE
SK-H	RM1	12;124813662;G;A	0.23	missense variant	NCOR2	.	unknown (0)	deleterious (0)	ENSP00000398963.2:p.Pro125Ser	LATE
SK-H	RM2	12;124825216;C;T	0.39	missense variant	NCOR2	.	probably damaging (0.996)	deleterious (0)	ENSP00000380513.1:p.Arg1748His	LATE
SK-H	RM2	20;50179159;C;T	0.15	missense variant	NFATC2	.	benign (0.024)	.	ENSP00000396471.1:p.Glu4Lys	LATE
SK-H	DM	5;176636767;C;T	0.16	missense variant	NSD1	.	benign (0.008)	tolerated (0.18)	ENSP00000343209.4:p.Pro187Leu	LATE
SK-H	RM1	11;71726660;G;A	0.09	missense variant	NUMA1	.	possibly damaging (0.61)	tolerated (0.08)	ENSP00000442936.1:p.Ala630Val	LATE
SK-H	RM1	16;23634381;C;T	0.10	missense variant	PALB2	.	possibly damaging (0.825)	tolerated (0.06)	ENSP00000261584.4:p.Val969Met	LATE
SK-H	RM2	9;127920588;G;A	0.56	missense variant	PPP6C	.	possibly damaging (0.568)	deleterious (0)	ENSP00000392147.1:p.Pro141Leu	LATE
SK-H	RM2	6;106536225;C;A	0.20	missense variant	PRDM1	.	probably damaging (0.998)	deleterious (0)	ENSP00000358087.2:p.Asp28Glu	LATE
SK-H	DM	12;70986100;C;T	0.20	missense variant	PTPRB	.	probably damaging (1)	deleterious (0)	ENSP00000447302.1:p.Gly363Asp	LATE
SK-H	RM1	12;70915289;G;A	0.07	missense variant	PTPRB	.	benign (0.002)	tolerated (0.55)	ENSP00000447302.1:p.Pro1902Ser	LATE
SK-H	RM2	12;70915289;G;A	0.33	missense variant	PTPRB	.	benign (0.002)	tolerated (0.55)	ENSP00000447302.1:p.Pro1902Ser	LATE
SK-H	DM	1;198668722;C;T	0.19	missense variant	PTPRC	.	benign (0.165)	tolerated (0.39)	ENSP00000356337.5:p.Pro44Ser	LATE
SK-H	DM	6;117639351;C;T	0.16	missense variant	ROS1	.	probably damaging (0.968)	deleterious (0.01)	ENSP00000357494.3:p.Ser2002Asn	LATE
SK-H	RM1	6;117674161;G;A	0.20	missense variant	ROS1	.	benign (0.038)	deleterious (0)	ENSP00000357494.3:p.Pro1438Leu	LATE

SK-H	RM1	6;117718208;G;A	0.17	missense variant	ROS1	.	probably damaging (1)	deleterious (0.01)	ENSP00000357494.3:p.Pro217Ser	LATE
SK-H	RM2	6;117665390;C;T	0.18	missense variant	ROS1	.	benign (0.007)	tolerated (0.71)	ENSP00000357494.3:p.Glu1453Lys	LATE
SK-H	RM2	3;47103675;C;T	0.57	missense variant	SETD2	.	probably damaging (0.999)	deleterious (0)	ENSP00000386759.3:p.Gly2091Arg	LATE
SK-H	RM1	16;11349151;G;A	0.14	missense variant	SOCS1	.	benign (0.035)	tolerated (0.28)	ENSP00000329418.2:p.Ala62Val	LATE
SK-H	DM	2;214727252;G;C	0.13	missense variant	SPAG16	.	probably damaging (0.946)	deleterious (0.01)	ENSP00000332592.5:p.Glu372Gln	LATE
SK-H	RM1	2;214149224;G;A	0.13	missense variant	SPAG16	.	possibly damaging (0.518)	deleterious (0.01)	ENSP00000400847.1:p.Gly6Glu	LATE
SK-H	RM1	10;70332720;C;T	0.97	missense variant	TET1	.	possibly damaging (0.762)	tolerated (0.12)	ENSP00000362748.4:p.Pro209Ser	LATE
SK-H	RM2	10;70332720;C;T	0.43	missense variant	TET1	.	possibly damaging (0.762)	tolerated (0.12)	ENSP00000362748.4:p.Pro209Ser	LATE
SK-H	RM1	4;106196942;C;T	0.17	missense variant	TET2	.	benign (0.016)	tolerated (0.46)	ENSP00000369351.4:p.Pro1759Ser	LATE
SK-H	RM2	4;106164727;G;A	0.55	missense variant	TET2	.	probably damaging (0.999)	deleterious (0)	ENSP00000369351.4:p.Val1199Met	LATE
SK-H	RM2	17;7578278;G;A	0.29	missense variant	TP53	43702	possibly damaging (0.847)	deleterious (0.04)	ENSP00000398846.2:p.Pro191Ser	LATE
SK-H	P	1;186340104;G;A	0.67	stop gained	TPR	.	.	.	ENSP00000356448.3:p.Gln110Ter	EARLY
SK-H	RM2	1;186328986;T;C	0.27	missense variant	TPR	.	.	deleterious (0.01)	ENSP00000356448.3:p.Glu445Gly	LATE
SK-H	RM1	16;2134295;C;T	0.10	missense variant	TSC2	.	probably damaging (0.993)	deleterious (0)	ENSP00000371978.6:p.Pro1243Ser	LATE
SK-H	RM1	16;2130291;G;A	0.13	missense variant	TSC2	.	benign (0.002)	tolerated (0.58)	ENSP00000384468.2:p.Val1131Ile	LATE
SK-H	RM2	16;2114273;G;A	0.14	missense variant	TSC2	.	possibly damaging (0.622)	deleterious (0.04)	ENSP00000384468.2:p.Glu482Lys	LATE
SK-H	RM2	8;103340042;C;T	0.23	missense variant	UBR5	.	probably damaging (0.994)	tolerated (0.8)	ENSP00000427819.1:p.Gly464Glu	LATE
SK-H	DM	16;72993674;C;T	0.21	missense variant	ZFHX3	.	possibly damaging (0.827)	.	ENSP00000268489.5:p.Ser124Asn	LATE
SK-H	RM1	16;72993149;G;A	0.08	missense variant	ZFHX3	.	probably damaging (0.969)	.	ENSP00000268489.5:p.Thr299Ile	LATE

Supplementary Table 7. Hotspot Mutations.

Patient	Sample	Variant	MAF	Mutation Type	Gene	COSMIC	Polyphen2	SIFT	AA Change	Time Variant Acquisition
CAS-G	DM1	7;140453136;A;T	0.67	missense variant	BRAF	476	possibly damaging (0.792)	deleterious (0)	ENSP00000288602.6:p.Val600Glu	EARLY
CAS-G	DM2	7;140453136;A;T	0.59	missense variant	BRAF	476	possibly damaging (0.792)	deleterious (0)	ENSP00000288602.6:p.Val600Glu	EARLY
CAS-G	DM3	7;140453136;A;T	0.67	missense variant	BRAF	476	possibly damaging (0.792)	deleterious (0)	ENSP00000288602.6:p.Val600Glu	EARLY
CAS-G	DM4	7;140453136;A;T	0.67	missense variant	BRAF	476	possibly damaging (0.792)	deleterious (0)	ENSP00000288602.6:p.Val600Glu	EARLY
CAS-G	DM5	7;140453136;A;T	0.60	missense variant	BRAF	476	possibly damaging (0.792)	deleterious (0)	ENSP00000288602.6:p.Val600Glu	EARLY
CAS-G	DM6	7;140453136;A;T	0.63	missense variant	BRAF	476	possibly damaging (0.792)	deleterious (0)	ENSP00000288602.6:p.Val600Glu	EARLY
CAS-G	P	7;140453136;A;T	0.50	missense variant	BRAF	476	possibly damaging (0.792)	deleterious (0)	ENSP00000288602.6:p.Val600Glu	EARLY
CAS-G	RM	7;140453136;A;T	0.50	missense variant	BRAF	476	possibly damaging (0.792)	deleterious (0)	ENSP00000288602.6:p.Val600Glu	EARLY
CAS-G	DM6	5;167988433;T;A	0.08	missense variant	PANK3	225568	possibly damaging (0.833)	deleterious (0)	ENSP00000239231.6:p.Ile301Phe	LATE
CAS-G	DM4	3;178936091;G;A	0.08	missense variant	PIK3CA	763,125370	probably damaging (0.959)	deleterious (0.01)	ENSP00000263967.3:p.Glu545Lys	LATE
CAS-G	DM6	3;178936082;G;A	0.21	missense variant	PIK3CA	760,125369	probably damaging (0.96)	tolerated (0.11)	ENSP00000263967.3:p.Glu542Lys	LATE
CAS-D	DM1	7;140453136;A;T	0.58	missense variant	BRAF	476	possibly damaging (0.792)	deleterious (0)	ENSP00000288602.6:p.Val600Glu	EARLY
CAS-D	DM2	7;140453136;A;T	0.50	missense variant	BRAF	476	possibly damaging (0.792)	deleterious (0)	ENSP00000288602.6:p.Val600Glu	EARLY
CAS-D	DM3	7;140453136;A;T	0.47	missense variant	BRAF	476	possibly damaging (0.792)	deleterious (0)	ENSP00000288602.6:p.Val600Glu	EARLY
CAS-D	DM4	7;140453136;A;T	0.50	missense variant	BRAF	476	possibly damaging (0.792)	deleterious (0)	ENSP00000288602.6:p.Val600Glu	EARLY
CAS-D	DM5	7;140453136;A;T	0.50	missense variant	BRAF	476	possibly damaging (0.792)	deleterious (0)	ENSP00000288602.6:p.Val600Glu	EARLY
CAS-D	DM6	7;140453136;A;T	0.49	missense variant	BRAF	476	possibly damaging (0.792)	deleterious (0)	ENSP00000288602.6:p.Val600Glu	EARLY
CAS-D	P1	7;140453136;A;T	0.45	missense variant	BRAF	476	possibly damaging (0.792)	deleterious (0)	ENSP00000288602.6:p.Val600Glu	EARLY
CAS-D	P2	7;140453136;A;T	0.44	missense variant	BRAF	476	possibly damaging (0.792)	deleterious (0)	ENSP00000288602.6:p.Val600Glu	EARLY
CAS-D	P3	7;140453136;A;T	0.50	missense variant	BRAF	476	possibly damaging (0.792)	deleterious (0)	ENSP00000288602.6:p.Val600Glu	EARLY
CAS-D	RM	7;140453136;A;T	0.50	missense variant	BRAF	476	possibly damaging (0.792)	deleterious (0)	ENSP00000288602.6:p.Val600Glu	EARLY
CAS-D	DM1	17;7577539;G;A	1.00	missense variant	TP53	10656,120007,120005, 1640831,120006	probably damaging (1)	deleterious (0)	ENSP00000398846.2:p.Arg248Trp	EARLY
CAS-D	DM2	17;7577539;G;A	0.54	missense variant	TP53	10656,120007,120005, 1640831,120006	probably damaging (1)	deleterious (0)	ENSP00000398846.2:p.Arg248Trp	EARLY
CAS-D	DM3	17;7577539;G;A	0.89	missense variant	TP53	10656,120007,120005, 1640831,120006	probably damaging (1)	deleterious (0)	ENSP00000398846.2:p.Arg248Trp	EARLY
CAS-D	DM4	17;7577539;G;A	0.91	missense variant	TP53	10656,120007,120005, 1640831,120006	probably damaging (1)	deleterious (0)	ENSP00000398846.2:p.Arg248Trp	EARLY
CAS-D	DM5	17;7577539;G;A	1.00	missense variant	TP53	10656,120007,120005, 1640831,120006	probably damaging (1)	deleterious (0)	ENSP00000398846.2:p.Arg248Trp	EARLY
CAS-D	DM6	17;7577539;G;A	0.79	missense variant	TP53	10656,120007,120005, 1640831,120006	probably damaging (1)	deleterious (0)	ENSP00000398846.2:p.Arg248Trp	EARLY
CAS-D	P1	17;7577539;G;A	0.64	missense variant	TP53	10656,120007,120005, 1640831,120006	probably damaging (1)	deleterious (0)	ENSP00000398846.2:p.Arg248Trp	EARLY
CAS-D	P2	17;7577539;G;A	1.00	missense variant	TP53	10656,120007,120005, 1640831,120006	probably damaging (1)	deleterious (0)	ENSP00000398846.2:p.Arg248Trp	EARLY
CAS-D	P3	17;7577539;G;A	0.54	missense variant	TP53	10656,120007,120005, 1640831,120006	probably damaging (1)	deleterious (0)	ENSP00000398846.2:p.Arg248Trp	EARLY

CAS-D	RM	17;7577539;G;A	0.91	missense variant	TP53	10656,120007,120005,1640831,120006	probably damaging (1)	deleterious (0)	ENSP00000398846.2:p.Arg248Trp	EARLY
CAS-F	DM1	7;140453136;A;T	0.71	missense variant	BRAF	476	possibly damaging (0.792)	deleterious (0)	ENSP00000288602.6:p.Val600Glu	EARLY
CAS-F	DM2	7;140453136;A;T	0.80	missense variant	BRAF	476	possibly damaging (0.792)	deleterious (0)	ENSP00000288602.6:p.Val600Glu	EARLY
CAS-F	DM3	7;140453136;A;T	0.69	missense variant	BRAF	476	possibly damaging (0.792)	deleterious (0)	ENSP00000288602.6:p.Val600Glu	EARLY
CAS-F	RM1	7;140453136;A;T	0.72	missense variant	BRAF	476	possibly damaging (0.792)	deleterious (0)	ENSP00000288602.6:p.Val600Glu	EARLY
CAS-F	RM2	7;140453136;A;T	0.75	missense variant	BRAF	476	possibly damaging (0.792)	deleterious (0)	ENSP00000288602.6:p.Val600Glu	EARLY
CAS-F	RM3	7;140453136;A;T	0.61	missense variant	BRAF	476	possibly damaging (0.792)	deleterious (0)	ENSP00000288602.6:p.Val600Glu	EARLY
CAS-F	DM1	9;21971017;G;A	1.00	missense variant	CDKN2A	753743,753742,12476	probably damaging (1)	deleterious (0)	ENSP00000307101.5:p.Pro114Leu	EARLY
CAS-F	DM2	9;21971017;G;A	1.00	missense variant	CDKN2A	753743,753742,12476	probably damaging (1)	deleterious (0)	ENSP00000307101.5:p.Pro114Leu	EARLY
CAS-F	DM3	9;21971017;G;A	0.90	missense variant	CDKN2A	753743,753742,12476	probably damaging (1)	deleterious (0)	ENSP00000307101.5:p.Pro114Leu	EARLY
CAS-F	RM1	9;21971017;G;A	1.00	missense variant	CDKN2A	753743,753742,12476	probably damaging (1)	deleterious (0)	ENSP00000307101.5:p.Pro114Leu	EARLY
CAS-F	RM2	9;21971017;G;A	1.00	missense variant	CDKN2A	753743,753742,12476	probably damaging (1)	deleterious (0)	ENSP00000307101.5:p.Pro114Leu	EARLY
CAS-F	RM3	9;21971017;G;A	1.00	missense variant	CDKN2A	753743,753742,12476	probably damaging (1)	deleterious (0)	ENSP00000307101.5:p.Pro114Leu	EARLY
CAS-F	DM1	2;138373853;G;A	0.35	missense variant	THSD7B	.	unknown (0)	tolerated (1)	ENSP00000272643.3:p.Glu1181Lys	EARLY
CAS-F	DM2	2;138373853;G;A	0.50	missense variant	THSD7B	.	unknown (0)	tolerated (1)	ENSP00000272643.3:p.Glu1181Lys	EARLY
CAS-F	DM3	2;138373853;G;A	0.50	missense variant	THSD7B	.	unknown (0)	tolerated (1)	ENSP00000272643.3:p.Glu1181Lys	EARLY
CAS-F	RM1	2;138373853;G;A	0.36	missense variant	THSD7B	.	unknown (0)	tolerated (1)	ENSP00000272643.3:p.Glu1181Lys	EARLY
CAS-F	RM2	2;138373853;G;A	0.50	missense variant	THSD7B	.	unknown (0)	tolerated (1)	ENSP00000272643.3:p.Glu1181Lys	EARLY
CAS-F	RM3	2;138373853;G;A	0.29	missense variant	THSD7B	.	unknown (0)	tolerated (1)	ENSP00000272643.3:p.Glu1181Lys	EARLY
CAS-E	DM1	7;140453136;A;T	0.50	missense variant	BRAF	476	possibly damaging (0.792)	deleterious (0)	ENSP00000288602.6:p.Val600Glu	EARLY
CAS-E	DM2	7;140453136;A;T	0.36	missense variant	BRAF	476	possibly damaging (0.792)	deleterious (0)	ENSP00000288602.6:p.Val600Glu	EARLY
CAS-E	DM3	7;140453136;A;T	0.34	missense variant	BRAF	476	possibly damaging (0.792)	deleterious (0)	ENSP00000288602.6:p.Val600Glu	EARLY
CAS-E	DM4	7;140453136;A;T	0.44	missense variant	BRAF	476	possibly damaging (0.792)	deleterious (0)	ENSP00000288602.6:p.Val600Glu	EARLY
CAS-E	DM5	7;140453136;A;T	0.44	missense variant	BRAF	476	possibly damaging (0.792)	deleterious (0)	ENSP00000288602.6:p.Val600Glu	EARLY
CAS-E	P	7;140453136;A;T	0.41	missense variant	BRAF	476	possibly damaging (0.792)	deleterious (0)	ENSP00000288602.6:p.Val600Glu	EARLY
CAS-E	RM	7;140453136;A;T	0.50	missense variant	BRAF	476	possibly damaging (0.792)	deleterious (0)	ENSP00000288602.6:p.Val600Glu	EARLY
CAS-E	DM2	1;115256530;G;T	0.20	missense variant	NRAS	580	benign (0.045)	deleterious (0.01)	ENSP00000358548.4:p.Gln61Lys	LATE
CAS-E	DM3	1;115256530;G;T	0.13	missense variant	NRAS	580	benign (0.045)	deleterious (0.01)	ENSP00000358548.4:p.Gln61Lys	LATE
CAS-E	DM5	1;115256530;G;T	0.15	missense variant	NRAS	580	benign (0.045)	deleterious (0.01)	ENSP00000358548.4:p.Gln61Lys	LATE
CAS-E	DM1	7;6426892;C;T	1.00	missense variant	RAC1	125734,1167878	probably damaging (0.95)	deleterious (0.02)	ENSP00000258737.7:p.Pro29Ser	EARLY
CAS-E	DM2	7;6426892;C;T	0.94	missense variant	RAC1	125734,1167878	probably damaging (0.95)	deleterious (0.02)	ENSP00000258737.7:p.Pro29Ser	EARLY
CAS-E	DM3	7;6426892;C;T	1.00	missense variant	RAC1	125734,1167878	probably damaging (0.95)	deleterious (0.02)	ENSP00000258737.7:p.Pro29Ser	EARLY
CAS-E	DM4	7;6426892;C;T	1.00	missense variant	RAC1	125734,1167878	probably damaging (0.95)	deleterious (0.02)	ENSP00000258737.7:p.Pro29Ser	EARLY

CAS-E	DM5	7;6426892;C;T	0.98	missense variant	RAC1	125734,1167878	probably damaging (0.95)	deleterious (0.02)	ENSP00000258737.7:p.Pro29Ser	EARLY
CAS-E	P	7;6426892;C;T	1.00	missense variant	RAC1	125734,1167878	probably damaging (0.95)	deleterious (0.02)	ENSP00000258737.7:p.Pro29Ser	EARLY
CAS-E	RM	7;6426892;C;T	1.00	missense variant	RAC1	125734,1167878	probably damaging (0.95)	deleterious (0.02)	ENSP00000258737.7:p.Pro29Ser	EARLY
CAS-A	DM1	7;140453136;A;T	0.51	missense variant	BRAF	476	possibly damaging (0.792)	deleterious (0)	ENSP00000288602.6:p.Val600Glu	EARLY
CAS-A	DM2	7;140453136;A;T	0.67	missense variant	BRAF	476	possibly damaging (0.792)	deleterious (0)	ENSP00000288602.6:p.Val600Glu	EARLY
CAS-A	DM3	7;140453136;A;T	0.60	missense variant	BRAF	476	possibly damaging (0.792)	deleterious (0)	ENSP00000288602.6:p.Val600Glu	EARLY
CAS-A	DM4	7;140453136;A;T	0.67	missense variant	BRAF	476	possibly damaging (0.792)	deleterious (0)	ENSP00000288602.6:p.Val600Glu	EARLY
CAS-A	RM1	7;140453136;A;T	0.79	missense variant	BRAF	476	possibly damaging (0.792)	deleterious (0)	ENSP00000288602.6:p.Val600Glu	EARLY
CAS-A	RM2	7;140453136;A;T	0.73	missense variant	BRAF	476	possibly damaging (0.792)	deleterious (0)	ENSP00000288602.6:p.Val600Glu	EARLY
CAS-A	RM3	7;140453136;A;T	0.83	missense variant	BRAF	476	possibly damaging (0.792)	deleterious (0)	ENSP00000288602.6:p.Val600Glu	EARLY
CAS-A	DM1	1;115256530;G;T	0.81	missense variant	NRAS	580	benign (0.045)	deleterious (0.01)	ENSP00000358548.4:p.Gln61Lys	LATE
CAS-A	DM2	1;115256530;G;T	0.75	missense variant	NRAS	580	benign (0.045)	deleterious (0.01)	ENSP00000358548.4:p.Gln61Lys	LATE
CAS-A	DM3	1;115256530;G;T	0.23	missense variant	NRAS	580	benign (0.045)	deleterious (0.01)	ENSP00000358548.4:p.Gln61Lys	LATE
CAS-A	DM4	1;115256530;G;T	0.74	missense variant	NRAS	580	benign (0.045)	deleterious (0.01)	ENSP00000358548.4:p.Gln61Lys	LATE
CAS-A	DM1	3;178936094;C;A	0.52	missense variant	PIK3CA	766,255876	probably damaging (0.952)	tolerated (0.23)	ENSP00000263967.3:p.Gln546Lys	EARLY
CAS-A	DM2	3;178936094;C;A	0.19	missense variant	PIK3CA	766,255876	probably damaging (0.952)	tolerated (0.23)	ENSP00000263967.3:p.Gln546Lys	EARLY
CAS-A	DM3	3;178936094;C;A	0.16	missense variant	PIK3CA	766,255876	probably damaging (0.952)	tolerated (0.23)	ENSP00000263967.3:p.Gln546Lys	EARLY
CAS-A	DM4	3;178936094;C;A	0.44	missense variant	PIK3CA	766,255876	probably damaging (0.952)	tolerated (0.23)	ENSP00000263967.3:p.Gln546Lys	EARLY
CAS-A	RM2	3;178936094;C;A	0.19	missense variant	PIK3CA	766,255876	probably damaging (0.952)	tolerated (0.23)	ENSP00000263967.3:p.Gln546Lys	EARLY
CAS-B	DM1	7;140453136;A;T	0.67	missense variant	BRAF	476	possibly damaging (0.792)	deleterious (0)	ENSP00000288602.6:p.Val600Glu	EARLY
CAS-B	DM2	7;140453136;A;T	0.71	missense variant	BRAF	476	possibly damaging (0.792)	deleterious (0)	ENSP00000288602.6:p.Val600Glu	EARLY
CAS-B	DM3	7;140453136;A;T	0.61	missense variant	BRAF	476	possibly damaging (0.792)	deleterious (0)	ENSP00000288602.6:p.Val600Glu	EARLY
CAS-B	DM4	7;140453136;A;T	0.61	missense variant	BRAF	476	possibly damaging (0.792)	deleterious (0)	ENSP00000288602.6:p.Val600Glu	EARLY
CAS-B	DM5	7;140453136;A;T	0.68	missense variant	BRAF	476	possibly damaging (0.792)	deleterious (0)	ENSP00000288602.6:p.Val600Glu	EARLY
CAS-B	P	7;140453136;A;T	0.66	missense variant	BRAF	476	possibly damaging (0.792)	deleterious (0)	ENSP00000288602.6:p.Val600Glu	EARLY
CAS-B	RM1	7;140453136;A;T	0.67	missense variant	BRAF	476	possibly damaging (0.792)	deleterious (0)	ENSP00000288602.6:p.Val600Glu	EARLY
CAS-B	RM2	7;140453136;A;T	0.66	missense variant	BRAF	476	possibly damaging (0.792)	deleterious (0)	ENSP00000288602.6:p.Val600Glu	EARLY
CAS-B	RM3	7;140453136;A;T	0.66	missense variant	BRAF	476	possibly damaging (0.792)	deleterious (0)	ENSP00000288602.6:p.Val600Glu	EARLY
CAS-B	RM2	5;167988433;T;A	0.3	missense variant	PANK3	225568	possibly damaging (0.833)	deleterious (0)	ENSP00000239231.6:p.Ile301Phe	LATE
CAS-B	DM1	5;167988433;T;A	0.39	missense variant	PANK3	225568	possibly damaging (0.833)	deleterious (0)	ENSP00000239231.6:p.Ile301Phe	LATE
CAS-B	DM3	5;167988433;T;A	0.42	missense variant	PANK3	225568	possibly damaging (0.833)	deleterious (0)	ENSP00000239231.6:p.Ile301Phe	LATE
CAS-C	DM1	7;140453134;T;C	0.89	missense variant	BRAF	478	benign (0.195)	deleterious (0)	ENSP00000288602.6:p.Lys601Glu	EARLY
CAS-C	DM2	7;140453134;T;C	0.77	missense variant	BRAF	478	benign (0.195)	deleterious (0)	ENSP00000288602.6:p.Lys601Glu	EARLY

CAS-C	DM3	7;140453134;T;C	0.86	missense variant	BRAF	478	benign (0.195)	deleterious (0)	ENSP00000288602.6:p.Lys601Glu	EARLY
CAS-C	DM4	7;140453134;T;C	0.86	missense variant	BRAF	478	benign (0.195)	deleterious (0)	ENSP00000288602.6:p.Lys601Glu	EARLY
CAS-C	DM5	7;140453134;T;C	0.78	missense variant	BRAF	478	benign (0.195)	deleterious (0)	ENSP00000288602.6:p.Lys601Glu	EARLY
CAS-C	DM6	7;140453134;T;C	0.85	missense variant	BRAF	478	benign (0.195)	deleterious (0)	ENSP00000288602.6:p.Lys601Glu	EARLY
CAS-C	P	7;140453134;T;C	0.83	missense variant	BRAF	478	benign (0.195)	deleterious (0)	ENSP00000288602.6:p.Lys601Glu	EARLY
CAS-C	DM4	3;41266136;T;C	0.49	missense variant	CTNNB1	5663	probably damaging (0.939)	deleterious (0)	ENSP00000401599.1:p.Ser45Pro	LATE
CAS-C	DM5	3;41266136;T;C	0.46	missense variant	CTNNB1	5663	probably damaging (0.939)	deleterious (0)	ENSP00000401599.1:p.Ser45Pro	LATE
CAS-C	DM6	3;41266136;T;C	0.50	missense variant	CTNNB1	5663	probably damaging (0.939)	deleterious (0)	ENSP00000401599.1:p.Ser45Pro	LATE
ETH-E	DM1	7;140453136;A;T	0.50	missense variant	BRAF	476	possibly damaging (0.792)	deleterious (0)	ENSP00000288602.6:p.Val600Glu	EARLY
ETH-E	DM2	7;140453136;A;T	0.50	missense variant	BRAF	476	possibly damaging (0.792)	deleterious (0)	ENSP00000288602.6:p.Val600Glu	EARLY
ETH-E	DM3	7;140453136;A;T	0.50	missense variant	BRAF	476	possibly damaging (0.792)	deleterious (0)	ENSP00000288602.6:p.Val600Glu	EARLY
ETH-E	P	7;140453136;A;T	0.48	missense variant	BRAF	476	possibly damaging (0.792)	deleterious (0)	ENSP00000288602.6:p.Val600Glu	EARLY
ETH-E	RM	7;140453136;A;T	0.47	missense variant	BRAF	476	possibly damaging (0.792)	deleterious (0)	ENSP00000288602.6:p.Val600Glu	EARLY
ETH-J	DM1	1;115256529;T;C	0.92	missense variant	NRAS	584	benign (0.006)	deleterious (0.03)	ENSP00000358548.4:p.Gln61Arg	EARLY
ETH-J	DM2	1;115256529;T;C	0.98	missense variant	NRAS	584	benign (0.006)	deleterious (0.03)	ENSP00000358548.4:p.Gln61Arg	EARLY
ETH-J	DM3	1;115256529;T;C	0.96	missense variant	NRAS	584	benign (0.006)	deleterious (0.03)	ENSP00000358548.4:p.Gln61Arg	EARLY
ETH-J	P1	1;115256529;T;C	0.91	missense variant	NRAS	584	benign (0.006)	deleterious (0.03)	ENSP00000358548.4:p.Gln61Arg	EARLY
ETH-J	P2	1;115256529;T;C	1.00	missense variant	NRAS	584	benign (0.006)	deleterious (0.03)	ENSP00000358548.4:p.Gln61Arg	EARLY
ETH-J	RM	1;115256529;T;C	0.98	missense variant	NRAS	584	benign (0.006)	deleterious (0.03)	ENSP00000358548.4:p.Gln61Arg	EARLY
MI-F	DM	12;46230641;C;T	0.33	missense variant	ARID2	1167991	probably damaging (0.997)	deleterious (0)	ENSP00000335044.6:p.Ser297Phe	EARLY
MI-F	P	12;46230641;C;T	0.30	missense variant	ARID2	1167991	probably damaging (0.997)	deleterious (0)	ENSP00000335044.6:p.Ser297Phe	EARLY
MI-F	RM1	12;46230641;C;T	0.33	missense variant	ARID2	1167991	probably damaging (0.997)	deleterious (0)	ENSP00000335044.6:p.Ser297Phe	EARLY
MI-F	RM2	12;46230641;C;T	0.30	missense variant	ARID2	1167991	probably damaging (0.997)	deleterious (0)	ENSP00000335044.6:p.Ser297Phe	EARLY
MI-F	DM	12;21918682;C;T	0.83	missense variant	KCNJ8	.	possibly damaging (0.538)	deleterious (0)	ENSP00000240662.2:p.Gly417Glu	EARLY
MI-F	P	12;21918682;C;T	1.00	missense variant	KCNJ8	.	possibly damaging (0.538)	deleterious (0)	ENSP00000240662.2:p.Gly417Glu	EARLY
MI-F	RM1	12;21918682;C;T	1.00	missense variant	KCNJ8	.	possibly damaging (0.538)	deleterious (0)	ENSP00000240662.2:p.Gly417Glu	EARLY
MI-F	RM2	12;21918682;C;T	0.98	missense variant	KCNJ8	.	possibly damaging (0.538)	deleterious (0)	ENSP00000240662.2:p.Gly417Glu	EARLY
MI-F	DM	15;40675107;C;T	0.67	missense variant	KNSTRN	140056	probably damaging (0.983)	deleterious (0.01)	ENSP00000453213.1:p.Ser24Phe	EARLY
MI-F	P	15;40675107;C;T	0.19	missense variant	KNSTRN	140056	probably damaging (0.983)	deleterious (0.01)	ENSP00000453213.1:p.Ser24Phe	EARLY
MI-F	RM1	15;40675107;C;T	0.38	missense variant	KNSTRN	140056	probably damaging (0.983)	deleterious (0.01)	ENSP00000453213.1:p.Ser24Phe	EARLY
MI-F	RM2	15;40675107;C;T	0.50	missense variant	KNSTRN	140056	probably damaging (0.983)	deleterious (0.01)	ENSP00000453213.1:p.Ser24Phe	EARLY
MI-F	DM	12;25378647;T;A	1.00	missense variant	KRAS	28519,1562192	benign (0.185)	deleterious (0.01)	ENSP00000308495.3:p.Lys117Asn	EARLY
MI-F	P	12;25378647;T;A	1.00	missense variant	KRAS	28519,1562192	benign (0.185)	deleterious (0.01)	ENSP00000308495.3:p.Lys117Asn	EARLY

MI-F	RM1	12;25378647;T;A	1.00	missense variant	KRAS	28519,1562192	benign (0.185)	deleterious (0.01)	ENSP00000308495.3:p.Lys117Asn	EARLY
MI-F	RM2	12;25378647;T;A	1.00	missense variant	KRAS	28519,1562192	benign (0.185)	deleterious (0.01)	ENSP00000308495.3:p.Lys117Asn	EARLY
MI-F	DM	6;31940123;G;A	0.27	missense variant	STK19	21037,226579	benign (0.108)	deleterious (0.03)	ENSP00000364482.2:p.Asp89Asn	EARLY
MI-F	P	6;31940123;G;A	0.23	missense variant	STK19	21037,226579	benign (0.108)	deleterious (0.03)	ENSP00000364482.2:p.Asp89Asn	EARLY
MI-F	RM1	6;31940123;G;A	0.25	missense variant	STK19	21037,226579	benign (0.108)	deleterious (0.03)	ENSP00000364482.2:p.Asp89Asn	EARLY
MI-F	RM2	6;31940123;G;A	0.21	missense variant	STK19	21037,226579	benign (0.108)	deleterious (0.03)	ENSP00000364482.2:p.Asp89Asn	EARLY
SK-G	DM	1;115256530;G;T	0.50	missense variant	NRAS	580	benign (0.045)	deleterious (0.01)	ENSP00000358548.4:p.Gln61Lys	EARLY
SK-G	P	1;115256530;G;T	0.45	missense variant	NRAS	580	benign (0.045)	deleterious (0.01)	ENSP00000358548.4:p.Gln61Lys	EARLY
SK-G	RM	1;115256530;G;T	0.50	missense variant	NRAS	580	benign (0.045)	deleterious (0.01)	ENSP00000358548.4:p.Gln61Lys	EARLY
SK-G	RM	3;178921549;T;G	0.50	missense variant	PIK3CA	22540,258749	probably damaging (0.987)	deleterious (0)	ENSP00000263967.3:p.Val344Gly	LATE

Supplementary Table 8. Neoantigenic mutations lost

Patient	LOH Event	N Mutations Deleted	Mutations Deleted (Early Stage MAF)	Neoantigens Deleted
CAS-E	11q	10	11;101827497;C;T (0.32)<br>11;101981760;T;G (0.32)<br>11;101985040;T;G (0.47)<br>11;101985043;T;G (0.45)<br>11;102098225;A;G (0.39)<br>11;102098226;G;C (0.39)<br>11;64005009;C;T (0.37)<br>11;64665339;G;A (0.40)<br>11;65651878;G;A (0.31)<br>11;66392293;T;A (0.27)	KIAA1377;11-101827497;PVSSSFMIFSSFPPILH<br>YAP1;11-101981760;QIVHVRGDAETDLEALF<br>YAP1;11-101985040;PTAQHLRQAAFEIPDDV<br>YAP1;11-101985043;TAQHLRQAAFEIPDDVP<br>YAP1;11-102098225;GTYHSRDEATDSGLSMS<br>YAP1;11-102098226;GTYHSRDEATDSGLSMS<br>VEGFB;11-64005009;PDSPRPLCSRCTQHHQR<br>ATG2A;11-64665339;PGGGHSPSSPDQQPIYF<br>FIBP;11-65651878;NQYSASVHFLDGFR<br>FIBP;11-65651878;NQYSASVHFLDGFRHQA<br>RBM14;11-66392293;AQPSASALTSYGGQAAA
CAS-F	15	7	15;49085622;A;G (0.36)<br>15;71184539;T;C (0.34)<br>15;77320248;C;T (0.37)<br>15;79058378;A;G (0.19)<br>15;85400577;C;T (0.47)<br>15;91292754;G;A (0.14)<br>15;75982058;T;A (0.22)	CEP152;15-49085622;NMQIIQLQALNKAKERQ<br>THAP10;15-71184539;GKSLFRFPEDRAVRLLW<br>PSTPIP1;15-77320248;SKLSLYKKVMESKKTYE<br>ADAMTS7;15-79058378;WPTVGVASPLPPPIAPL<br>ALPK3;15-85400577;QEVPTMPSFPGTGLTAS<br>BLM;15-91292754;PNTTNQQRIKDFFKNAP<br>CSPG4; 15-75982058;GGTVWLEWWHVQPTLDL
SK-G	X	6	X;10102557;G;T (0.27)<br>X;101910581;C;G (0.50)<br>X;153069521;C;T (0.44)<br>X;18283733;C;T (0.40)<br>X;71821870;G;A (0.50)<br>X;79945559;T;C (0.50)	WWC3;X-10102557;ERPSRRARVSPFVRSGT<br>GPRASP1;X-101910581;PGAEEETILGSWFWAEN<br>PDZD4;X-153069521;AKFRSLSRNPEAGRRQH<br>SCML2;X-18283733;SSVKNITPKKKGPNSGK<br>PHKA1;X-71821870;SEIKQVEFCRLSISAES<br>BRWD3;X-79945559;SALMWEVRCIEHNARTF
CAS-F	8p	5	8;17486239;C;T (0.43)<br>8;38283717;G;A (0.59)<br>8;30553945;G;A (0.5)<br>8;30974004;G;A (0.5)<br>8;22069129;C;T (0.18)	PDGFRL;8-17486239;GVVYCRAEVGGRSQISV<br>FGFR1;8-38283717;IIMDSVVPFDKGNYTC<br>FGFR1;8-38283717;IIMDSVVPFDKGNYTCI<br>GSR;8-30553945;HCCGDGRDSVSPGF<br>WRN; 8-30974004;HAGMSFSTKKDIHHRFV<br>BMP1;8-22069129;GPPEEVYSVGDSVLVKF
CAS-D	1q	4	1;196716355;G;A (0.42)<br>1;196716356;G;T (0.42)<br>1;207753729;G;A (0.47)<br>1;207789988;C;T (0.50)	CFH;1-196716355;ESVEFVCKHGYRLSSRS<br>CFH;1-196716356;ESVEFVCKHGYRLSSRS<br>CR1;1-207753729;ASLHCTPQEDWSPEAPR<br>CR1;1-207789988;TGTPFGDISYGKEISYA
CAS-D	3q	4	3;157920867;G;T (0.50)<br>3;168806865;C;T (0.48)<br>3;170893054;T;G (0.50)<br>3;173998974;C;T (0.50)	RSRC1;3-157920867;SKSRTRRSSSRPRLRSH<br>MECOM;3-168806865;MEDNQYSETELSSFSTS<br>TNIK;3-170893054;PRNPAPRLQSKKWSKKF<br>NLGN1;3-173998974;TPNTITMISNTIPGIQP
CAS-D	5	3	5;1463843;G;A (0.50)<br>5;76003085;G;A (0.50)<br>5;87492143;A;T (0.50)	LPCAT1;5-1463843;AETSPAPISNGFCADFS<br>IQGAP2;5-76003085;MKMFDKVKMNVNLLIYL<br>TMEM161B;5-87492143;ALSSLKNIYTPLLFRGL
SK-H	12	3	12;42499802;C;A(0.4)<br>12;46230743;G;A(0.39)<br>12;94543124;G;A(0.04)	GXYLT1;12-42499802;ILFLRPVDYIWSLLKKF<br>ARID2;12-46230743;HFISLRQLDLDTLGNIA<br>PLXNC1;12-94543124;GLGGLLLTDWTFDRGAC
SK-G	8	2	8;108334329;C;T (0.50)<br>8;21768354;C;T (0.41)	ANGPT1;8-108334329;LLEHKILEIEGKHKEEL<br>DOK2;8-21768354;AVTVGPHKKFAVTMRPT
SK-G	17p	2	17;15968264;G;A (0.40)<br>17;7246855;G;A (0.50)	NCOR1;17-15968264;HPGGTSTPLMDRITYIP<br>ACAP1;17-7246855;RTARAGYRRRALDYA<br>ACAP1;17-7246855;RTARAGYRRRALDYALQ
CAS-D	10p	2	10;30318683;C;T (0.67)<br>10;34400116;C;T (0.66)	KIAA1462;10-30318683;RSQKPREHKNLEARGMA<br>PARD3;10-34400116;NRLQTPEKERPFYS
CAS-A	1p	2	1;109796000;T;C (0.35)<br>1;22985978;T;C (0.33)	CELSR2;1-109796000;RPLEAIMSALVSDGVHS<br>C1QB;1-22985978;MMKIPWGSTPVLMLLLL<br>C1QB;1-22985978;PMKIPWGSTPVLMLLLL<br>C1QB;1-22985978;SMKIPWGSTPVLMLLLL
CAS-E	18	2	18;12308722;C;T (0.47)<br>18;77470376;C;T (0.44)	TUBB6;18-12308722;ISDEHGIDSAGGYVGDS<br>CTDP1;18-77470376;LDPEKKLFFHRILSRDE
CAS-E	17	2	17;48627459;C;T (0.24)<br>17;7578472;G;A (0.37)	SPATA20;17-48627459;KMMANGGIWDHVGQGFH<br>SPATA20;17-48627459;KMMANGGIWDHVGQTAS<br>TP53;17-7578472;LWVDSTPPLGTRVRAMA
CAS-B	10	2	10;17195512;C;T (0.48)<br>10;50004362;G;A (0.50)	TRDMT1;10-17195512;ANLLGFPPKFGFPEKIT<br>TRDMT1;10-17195512;ANLLGFPPKFGMWDTCR<br>WDFY4;10-50004362;YQIMAFLLKKKASLLNH

CAS-C	12q21.2-12q24.31	2	12;90021464;G;A (0.15)<br>12;91498054;G;A (0.50)	ATP2B1;12-90021464;KQDGAIENCNKAKAQDG<br>LUM;12-91498054;FCKILGPLFYSKIKHLR
ETH-J	14	2	14;103371750;T;C (0.50)<br>14;23746261;G;A (0.50)	TRAF3;14-103371750;LSLYSQPFHTGYFGYKM<br>HOMEZ;14-23746261;LQLVWTQAVQTSELDSN
SK-G	1p36	2	1;11252401;G;A (0.43)<br>1;16577607;G;A (0.47)	ANGPTL7;1-11252401;GVYKLPPDNFLGSPELE<br>FBXO42;1-16577607;IKAMSSKGLSASAALSP
SK-H	6q	1	6;158296120;G;A (0.01)	SNX9;6-158296120;DGKDQFSCENSVADQAF
SK-H	8	3	8;145770957;C;T (0.25)<br>8;37698295;G;A(0.02)<br>8;22548509;C;T(0.004)	ARHGAP39;8-145770957;ESIKKPMIMTSDRHVKK<br>GPR124;8-37698295;MLRFYLIAEGIPLIICG<br>EGR3;8-22548509;IPEHKPFQDMDPIRVNP
SK-H	15q21.3-15q26	1	15;90969454;G;A (0.03)	IQGAP1;15-90969454;GNFFSPKVMSLKKIYDR
SK-H	17	1	17;56585561;G;A (0.50)	MTMR4;17-56585561;DKELENVAFFRSWKRIP
CAS-D	2	1	2;98421844;G;A (0.47)	TMEM131;2-98421844;CYVGLPFLFKSEPKVQP
CAS-D	4q	1	4;148546070;G;A (0.50)	TMEM184C;4-148546070;FLTNYLTNQYPNLVLIL
CAS-D	4p	1	4;42466932;G;A (0.44)	ATP8A1;4-42466932;NEGLQAANFSDYSIAQF
CAS-D	6q	1	6;152557362;G;A (0.50)	SYNE1;6-152557362;DDGKRLLIFISCSDLES
CAS-D	16q23-16q24	1	16;88503957;G;A (0.50)	ZNF469;16-88503957;LQRHLAVHNPQRVYLCP
CAS-F	4:190330186-191044268	1	4;190882998;A;G (0.19)	FRG1;4-190882998;TERNFRASRTTNLK
CAS-F	6p21.32	1	6;32552015;C;G(0.18)	HLA-DRB1;6-32552015;VGEFRAVTQLGRPDAEY
CAS-E	11p	1	11;1905806;A;C(0.42)	LSP1;11-1905806;WLEQYTQALETAGRTPK
CAS-E	16p	1	16;1614071;G;A(0.31)	IFT140;16-1614071;EPRLFVCEVVQETPRSQ
CAS-B	15	1	15;33359865;G;T(0.05)	FMN1;15-33359865;NPTSQQDDYSNGLDPQE
CAS-B	7p21	1	7;16460821;G;A (0.12)	ISPD;7-16460821;VAGTEPGRYPQAVAAVL
CAS-C	14q32.31-14q32.33	1	14;103372102;C;T(0.15)	TRAF3;14-103372102;FIKVIVDTLDLPDP
CAS-C	1p36.33-1p36.32	1	1;1374916;G;A(0.15)	VWA1;1-1374916;ALGSAAALSYHVQFGPL
CAS-C	1p31.3-1p31.2	1	1;64095682;G;A (0.23)	PGM1;1-64095682;KTIEEYAVYPDLKVDLG
CAS-C	20p	1	20;9368184;G;A (0.18)	PLCB4;20-9368184;IITHGKAMYTDILFKDV
ETH-J	11p	1	11;46921021;C;A (0.29)	LRP4;11-46921021;KCSDKEFRFSDGSCIAE
ETH-J	17q21.31-17q25.3	1	17;66920899;G;A (0.50)	ABCA8;17-66920899;EDEMDADPLFHDSFEQA

Supplementary Table 9. Number of regions in each patient with more/fewer non-synonymous mutations than expected based on the synonymous rate and codon usage bias and high/low neo-antigenic load (based on the median rate across the genome). One-sided tests of independence (mutual exclusivity) were performed using Barnard's test as implemented in the R package Exact.

Patient	NS:S above expected rate		NS:S below expected rate		Total per patient	P-value
	Neo-antigens below background	Neo-antigens above background	Neo-antigens below background	Neo-antigens above background		
**CAS-A**	2	0	2	2	6	1
**CAS-B**	7	0	0	6	13	1
**CAS-C**	1	1	1	2	5	0.7
**CAS-D**	9	22	13	9	53	0.02
**CAS-E**	11	4	1	3	19	0.9
**CAS-F**	0	1	0	1	2	NA
**CAS-G**	0	0	6	0	6	NA
**ETH-E**	0	2	4	7	13	0.2
**ETH-F**	0	0	1	0	1	NA
**ETH-J**	0	0	0	2	2	NA
**SK-G**	1	1	3	1	6	0.4
**SK-H**	4	2	0	3	9	1
**Total**	35	33	31	36	135	0.8

Supplementary Table 10. Mutations in genes that regulate chromosomal integrity.

Patient	Sample	Variant	MAF	Mutation Type	Gene	COSMIC	Polyphen2	SIFT	AA Change
CAS-G	DM4	1;186291703;G;A	0.03	stop_gained	TPR	.	.	.	ENSP00000356448.3:p.Arg2105Ter
CAS-G	DM5	11;108141866;C;T	0.17	missense_variant	ATM	.	benign(0.252)	deleterious(0.04)	ENSP00000388058.2:p.Pro972Ser
CAS-G	DM6	11;108178681;C;T	0.13	missense_variant	ATM	.	benign(0.052)	tolerated(0.25)	ENSP00000278616.4:p.Ala1911Val
CAS-G	DM6	11;71723981;C;T	0.06	missense_variant	NUMA1	.	probably_damaging(0.992)	tolerated(1)	ENSP00000446396.1:p.Arg127Lys
CAS-G	DM6	13;103515417;C;T	0.09	missense_variant	ERCC5	.	benign(0.063)	tolerated(0.14)	ENSP00000347978.4:p.Pro640Ser
CAS-G	DM3	16;23646286;A;C	0.51	missense_variant	PALB2	.	benign(0.005)	tolerated(0.13)	ENSP00000261584.4:p.Cys527Trp
CAS-G	DM4	16;23646286;A;C	0.66	missense_variant	PALB2	.	benign(0.005)	tolerated(0.13)	ENSP00000261584.4:p.Cys527Trp
CAS-G	DM1	16;23646286;A;C	0.67	missense_variant	PALB2	.	benign(0.005)	tolerated(0.13)	ENSP00000261584.4:p.Cys527Trp
CAS-G	DM2	16;23646286;A;C	0.52	missense_variant	PALB2	.	benign(0.005)	tolerated(0.13)	ENSP00000261584.4:p.Cys527Trp
CAS-G	DM5	16;23646286;A;C	0.50	missense_variant	PALB2	.	benign(0.005)	tolerated(0.13)	ENSP00000261584.4:p.Cys527Trp
CAS-G	DM6	16;23646286;A;C	0.51	missense_variant	PALB2	.	benign(0.005)	tolerated(0.13)	ENSP00000261584.4:p.Cys527Trp
CAS-G	P	16;23646286;A;C	0.31	missense_variant	PALB2	.	benign(0.005)	tolerated(0.13)	ENSP00000261584.4:p.Cys527Trp
CAS-G	RM	16;23646286;A;C	0.42	missense_variant	PALB2	.	benign(0.005)	tolerated(0.13)	ENSP00000261584.4:p.Cys527Trp
CAS-G	DM6	16;89815076;G;A	0.05	missense_variant	FANCA	.	unknown(0)	.	ENSP00000456588.1:p.Leu182Phe
CAS-G	DM4	16;89816195;C;T	0.11	missense_variant	FANCA	.	benign(0.023)	tolerated(1)	ENSP00000455974.1:p.Ser11Asn
CAS-G	DM3	16;89877451;C;T	0.10	missense_variant	FANCA	.	benign(0.001)	deleterious(0.03)	ENSP00000455946.1:p.Ala100Thr
CAS-G	DM5	17;41243719;C;T	0.21	missense_variant	BRCA1	.	benign(0.011)	tolerated(0.29)	ENSP00000417241.1:p.Ala42Thr
CAS-G	DM6	17;7579550;G;A	0.10	missense_variant	TP53	45013	benign(0.266)	tolerated(0.69)	ENSP00000398846.2:p.Ser46Phe
CAS-G	DM4	2;48018128;G;A	0.06	missense_variant	MSH6	.	probably_damaging(0.999)	deleterious(0)	ENSP00000475605.1:p.Cys9Tyr
CAS-G	DM5	2;48027175;G;A	0.24	missense_variant	MSH6	.	probably_damaging(1)	deleterious(0)	ENSP00000446475.1:p.Gly555Ser
CAS-G	DM6	2;48027431;G;A	0.20	missense_variant	MSH6	.	probably_damaging(1)	deleterious(0)	ENSP00000446475.1:p.Gly640Asp
CAS-G	DM3	2;48033352;C;T	0.15	missense_variant	MSH6	13892	probably_damaging(0.999)	deleterious(0)	ENSP00000446475.1:p.Thr1089Ile
CAS-G	DM4	2;48033352;C;T	0.13	missense_variant	MSH6	13892	probably_damaging(0.999)	deleterious(0)	ENSP00000446475.1:p.Thr1089Ile
CAS-G	DM1	2;48033352;C;T	0.12	missense_variant	MSH6	13892	probably_damaging(0.999)	deleterious(0)	ENSP00000446475.1:p.Thr1089Ile
CAS-G	DM6	2;48033352;C;T	0.27	missense_variant	MSH6	13892	probably_damaging(0.999)	deleterious(0)	ENSP00000446475.1:p.Thr1089Ile
CAS-G	DM5	2;48033791;G;A	0.13	splice_donor_variant	MSH6	.	.	.	.
CAS-G	DM6	22;41513691;G;A	0.18	missense_variant	EP300	.	unknown(0)	tolerated(0.07)	ENSP00000263253.7:p.Gly199Ser
CAS-G	DM5	3;37050383;G;A	0.05	missense_variant	MLH1	.	possibly_damaging(0.792)	deleterious(0.01)	ENSP00000402564.1:p.Glu80Lys
CAS-G	DM4	3;37089083;G;A	0.06	missense_variant	MLH1	.	probably_damaging(0.998)	deleterious(0)	ENSP00000443665.1:p.Gly361Asp
CAS-G	DM4	3;41266493;C;T	0.08	missense_variant	CTNNB1	.	benign(0.126)	tolerated(0.08)	ENSP00000401599.1:p.Ala97Val
CAS-G	DM4	9;110250310;G;A	0.05	missense_variant	KLF4	.	possibly_damaging(0.763)	tolerated(0.3)	ENSP00000363804.4:p.Ala122Val
CAS-G	DM3	9;110250325;G;A	0.04	missense_variant	KLF4	.	benign(0.036)	tolerated(0.09)	ENSP00000363804.4:p.Pro117Leu
CAS-D	DM1	17;57760301;C;T	1.00	missense_variant	CLTC	.	unknown(0)	deleterious(0.01)	ENSP00000462252.1:p.Arg204Cys
CAS-D	P1	17;57760301;C;T	1.00	missense_variant	CLTC	.	unknown(0)	deleterious(0.01)	ENSP00000462252.1:p.Arg204Cys
CAS-D	P2	17;57760301;C;T	0.87	missense_variant	CLTC	.	unknown(0)	deleterious(0.01)	ENSP00000462252.1:p.Arg204Cys
CAS-D	P3	17;57760301;C;T	0.97	missense_variant	CLTC	.	unknown(0)	deleterious(0.01)	ENSP00000462252.1:p.Arg204Cys

CAS-D	DM2	17;57760301;C;T	1.00	missense_variant	CLTC	.	unknown(0)	deleterious(0.01)	ENSP00000462252.1:p.Arg204Cys
CAS-D	DM3	17;57760301;C;T	1.00	missense_variant	CLTC	.	unknown(0)	deleterious(0.01)	ENSP00000462252.1:p.Arg204Cys
CAS-D	DM4	17;57760301;C;T	0.94	missense_variant	CLTC	.	unknown(0)	deleterious(0.01)	ENSP00000462252.1:p.Arg204Cys
CAS-D	DM5	17;57760301;C;T	1.00	missense_variant	CLTC	.	unknown(0)	deleterious(0.01)	ENSP00000462252.1:p.Arg204Cys
CAS-D	DM6	17;57760301;C;T	1.00	missense_variant	CLTC	.	unknown(0)	deleterious(0.01)	ENSP00000462252.1:p.Arg204Cys
CAS-D	RM	17;57760301;C;T	0.98	missense_variant	CLTC	.	unknown(0)	deleterious(0.01)	ENSP00000462252.1:p.Arg204Cys
CAS-D	DM1	17;7577539;G;A	1.00	missense_variant	TP53	106,561,200,071,200,000,000,000,000,000	probably_damaging(1)	deleterious(0)	ENSP00000398846.2:p.Arg248Trp
CAS-D	P1	17;7577539;G;A	0.64	missense_variant	TP53	106,561,200,071,200,000,000,000,000,000	probably_damaging(1)	deleterious(0)	ENSP00000398846.2:p.Arg248Trp
CAS-D	P2	17;7577539;G;A	1.00	missense_variant	TP53	106,561,200,071,200,000,000,000,000,000	probably_damaging(1)	deleterious(0)	ENSP00000398846.2:p.Arg248Trp
CAS-D	P3	17;7577539;G;A	0.54	missense_variant	TP53	106,561,200,071,200,000,000,000,000,000	probably_damaging(1)	deleterious(0)	ENSP00000398846.2:p.Arg248Trp
CAS-D	DM2	17;7577539;G;A	0.54	missense_variant	TP53	106,561,200,071,200,000,000,000,000,000	probably_damaging(1)	deleterious(0)	ENSP00000398846.2:p.Arg248Trp
CAS-D	DM3	17;7577539;G;A	0.89	missense_variant	TP53	106,561,200,071,200,000,000,000,000,000	probably_damaging(1)	deleterious(0)	ENSP00000398846.2:p.Arg248Trp
CAS-D	DM4	17;7577539;G;A	0.91	missense_variant	TP53	106,561,200,071,200,000,000,000,000,000	probably_damaging(1)	deleterious(0)	ENSP00000398846.2:p.Arg248Trp
CAS-D	DM5	17;7577539;G;A	1.00	missense_variant	TP53	106,561,200,071,200,000,000,000,000,000	probably_damaging(1)	deleterious(0)	ENSP00000398846.2:p.Arg248Trp
CAS-D	DM6	17;7577539;G;A	0.79	missense_variant	TP53	106,561,200,071,200,000,000,000,000,000	probably_damaging(1)	deleterious(0)	ENSP00000398846.2:p.Arg248Trp
CAS-D	RM	17;7577539;G;A	0.91	missense_variant	TP53	106,561,200,071,200,000,000,000,000,000	probably_damaging(1)	deleterious(0)	ENSP00000398846.2:p.Arg248Trp
CAS-D	DM1	6;167417807;C;CA	0.32	frameshift_variant	FGFR1OP	.	.	.	ENSP00000355812.3:p.Glu120ArgfsTer31
CAS-D	P1	6;167417807;C;CA	0.33	frameshift_variant	FGFR1OP	.	.	.	ENSP00000355812.3:p.Glu120ArgfsTer31
CAS-D	P2	6;167417807;C;CA	0.44	frameshift_variant	FGFR1OP	.	.	.	ENSP00000355812.3:p.Glu120ArgfsTer31
CAS-D	P3	6;167417807;C;CA	0.31	frameshift_variant	FGFR1OP	.	.	.	ENSP00000355812.3:p.Glu120ArgfsTer31
CAS-D	DM2	6;167417807;C;CA	0.47	frameshift_variant	FGFR1OP	.	.	.	ENSP00000355812.3:p.Glu120ArgfsTer31
CAS-D	DM3	6;167417807;C;CA	0.48	frameshift_variant	FGFR1OP	.	.	.	ENSP00000355812.3:p.Glu120ArgfsTer31
CAS-D	DM4	6;167417807;C;CA	1.00	frameshift_variant	FGFR1OP	.	.	.	ENSP00000355812.3:p.Glu120ArgfsTer31

CAS-D	DM5	6;167417807;C;CA	0.47	frameshift_variant	FGFR1OP	.	.	.	ENSP00000355812.3:p.Glu120ArgfsTer31
CAS-D	DM6	6;167417807;C;CA	0.40	frameshift_variant	FGFR1OP	.	.	.	ENSP00000355812.3:p.Glu120ArgfsTer31
CAS-D	RM	6;167417807;C;CA	0.63	frameshift_variant	FGFR1OP	.	.	.	ENSP00000355812.3:p.Glu120ArgfsTer31
CAS-F	RM2	11;3765753;G;A	0.96	missense_variant	NUP98	.	possibly_damaging(0.623)	tolerated(0.19)	ENSP00000352091.4:p.Pro465Leu
CAS-F	RM3	11;3765753;G;A	1.00	missense_variant	NUP98	.	possibly_damaging(0.623)	tolerated(0.19)	ENSP00000352091.4:p.Pro465Leu
CAS-F	RM1	11;3765753;G;A	0.57	missense_variant	NUP98	.	possibly_damaging(0.623)	tolerated(0.19)	ENSP00000352091.4:p.Pro465Leu
CAS-F	DM2	11;3765753;G;A	0.96	missense_variant	NUP98	.	possibly_damaging(0.623)	tolerated(0.19)	ENSP00000352091.4:p.Pro465Leu
CAS-F	DM3	11;3765753;G;A	1.00	missense_variant	NUP98	.	possibly_damaging(0.623)	tolerated(0.19)	ENSP00000352091.4:p.Pro465Leu
CAS-F	DM1	11;3765753;G;A	0.82	missense_variant	NUP98	.	possibly_damaging(0.623)	tolerated(0.19)	ENSP00000352091.4:p.Pro465Leu
CAS-F	RM2	15;91292754;G;A	0.14	missense_variant	BLM	.	benign(0)	tolerated(1)	ENSP00000347232.3:p.Val86Ile
CAS-F	RM3	15;91292754;G;A	0.12	missense_variant	BLM	.	benign(0)	tolerated(1)	ENSP00000347232.3:p.Val86Ile
CAS-F	RM1	15;91292754;G;A	0.14	missense_variant	BLM	.	benign(0)	tolerated(1)	ENSP00000347232.3:p.Val86Ile
CAS-F	DM2	15;91292754;G;A	0.18	missense_variant	BLM	.	benign(0)	tolerated(1)	ENSP00000347232.3:p.Val86Ile
CAS-F	DM1	15;91292754;G;A	0.25	missense_variant	BLM	.	benign(0)	tolerated(1)	ENSP00000347232.3:p.Val86Ile
CAS-F	RM2	8;30974004;G;A	0.50	missense_variant	WRN	.	probably_damaging(0.999)	deleterious(0)	ENSP00000298139.5:p.Arg803Lys
CAS-F	RM3	8;30974004;G;A	0.43	missense_variant	WRN	.	probably_damaging(0.999)	deleterious(0)	ENSP00000298139.5:p.Arg803Lys
CAS-F	RM1	8;30974004;G;A	0.14	missense_variant	WRN	.	probably_damaging(0.999)	deleterious(0)	ENSP00000298139.5:p.Arg803Lys
CAS-F	DM3	8;30974004;G;A	0.50	missense_variant	WRN	.	probably_damaging(0.999)	deleterious(0)	ENSP00000298139.5:p.Arg803Lys
CAS-F	DM1	8;30974004;G;A	0.29	missense_variant	WRN	.	probably_damaging(0.999)	deleterious(0)	ENSP00000298139.5:p.Arg803Lys
CAS-F	RM2	9;21971017;G;A	1.00	missense_variant	CDKN2A	75,374,375,374,212,400	probably_damaging(1)	deleterious(0)	ENSP00000307101.5:p.Pro114Leu
CAS-F	RM3	9;21971017;G;A	1.00	missense_variant	CDKN2A	75,374,375,374,212,400	probably_damaging(1)	deleterious(0)	ENSP00000307101.5:p.Pro114Leu
CAS-F	RM1	9;21971017;G;A	1.00	missense_variant	CDKN2A	75,374,375,374,212,400	probably_damaging(1)	deleterious(0)	ENSP00000307101.5:p.Pro114Leu
CAS-F	DM2	9;21971017;G;A	1.00	missense_variant	CDKN2A	75,374,375,374,212,400	probably_damaging(1)	deleterious(0)	ENSP00000307101.5:p.Pro114Leu
CAS-F	DM3	9;21971017;G;A	0.90	missense_variant	CDKN2A	75,374,375,374,212,400	probably_damaging(1)	deleterious(0)	ENSP00000307101.5:p.Pro114Leu
CAS-F	DM1	9;21971017;G;A	1.00	missense_variant	CDKN2A	75,374,375,374,212,400	probably_damaging(1)	deleterious(0)	ENSP00000307101.5:p.Pro114Leu
CAS-E	DM1	16;23641109;A;G	0.66	missense_variant	PALB2	.	benign(0.126)	deleterious(0.01)	ENSP00000261584.4:p.Leu789Pro
CAS-E	RM	16;23641109;A;G	0.50	missense_variant	PALB2	.	benign(0.126)	deleterious(0.01)	ENSP00000261584.4:p.Leu789Pro
CAS-E	DM2	16;23641109;A;G	0.98	missense_variant	PALB2	.	benign(0.126)	deleterious(0.01)	ENSP00000261584.4:p.Leu789Pro
CAS-E	DM3	16;23641109;A;G	0.67	missense_variant	PALB2	.	benign(0.126)	deleterious(0.01)	ENSP00000261584.4:p.Leu789Pro
CAS-E	DM4	16;23641109;A;G	0.50	missense_variant	PALB2	.	benign(0.126)	deleterious(0.01)	ENSP00000261584.4:p.Leu789Pro
CAS-E	DM5	16;23641109;A;G	0.50	missense_variant	PALB2	.	benign(0.126)	deleterious(0.01)	ENSP00000261584.4:p.Leu789Pro
CAS-E	P	16;23641109;A;G	0.50	missense_variant	PALB2	.	benign(0.126)	deleterious(0.01)	ENSP00000261584.4:p.Leu789Pro
CAS-E	DM1	17;7578472;G;A	0.28	missense_variant	TP53	44367	benign(0.014)	tolerated(0.21)	ENSP00000398846.2:p.Pro153Leu
CAS-E	RM	17;7578472;G;A	0.39	missense_variant	TP53	44367	benign(0.014)	tolerated(0.21)	ENSP00000398846.2:p.Pro153Leu
CAS-E	DM2	17;7578472;G;A	0.40	missense_variant	TP53	44367	benign(0.014)	tolerated(0.21)	ENSP00000398846.2:p.Pro153Leu
CAS-E	DM3	17;7578472;G;A	0.31	missense_variant	TP53	44367	benign(0.014)	tolerated(0.21)	ENSP00000398846.2:p.Pro153Leu
CAS-E	DM5	17;7578472;G;A	0.31	missense_variant	TP53	44367	benign(0.014)	tolerated(0.21)	ENSP00000398846.2:p.Pro153Leu

CAS-E	P	17;7578472;G;A	0.35	missense_variant	TP53	44367	benign(0.014)	tolerated(0.21)	ENSP00000398846.2:p.Pro153Leu
CAS-E	DM1	9;21974714;GGCAGCG CCCCCGCCTCCA;G	0.64	inframe_deletion	CDKN2A	24418	.	.	ENSP00000464202.1:p.Leu32_Leu37del
CAS-E	RM	9;21974714;GGCAGCG CCCCCGCCTCCA;G	0.69	inframe_deletion	CDKN2A	24418	.	.	ENSP00000464202.1:p.Leu32_Leu37del
CAS-E	DM2	9;21974714;GGCAGCG CCCCCGCCTCCA;G	0.62	inframe_deletion	CDKN2A	24418	.	.	ENSP00000464202.1:p.Leu32_Leu37del
CAS-E	DM3	9;21974714;GGCAGCG CCCCCGCCTCCA;G	0.55	inframe_deletion	CDKN2A	24418	.	.	ENSP00000464202.1:p.Leu32_Leu37del
CAS-E	DM4	9;21974714;GGCAGCG CCCCCGCCTCCA;G	0.66	inframe_deletion	CDKN2A	24418	.	.	ENSP00000464202.1:p.Leu32_Leu37del
CAS-E	DM5	9;21974714;GGCAGCG CCCCCGCCTCCA;G	0.57	inframe_deletion	CDKN2A	24418	.	.	ENSP00000464202.1:p.Leu32_Leu37del
CAS-E	P	9;21974714;GGCAGCG CCCCCGCCTCCA;G	0.66	inframe_deletion	CDKN2A	24418	.	.	ENSP00000464202.1:p.Leu32_Leu37del
CAS-A	DM3	12;133220089;G;C	0.26	missense_variant	POLE	.	benign(0.002)	tolerated(1)	ENSP00000445753.1:p.Gln1423Glu
CAS-A	DM1	12;133220089;G;C	0.16	missense_variant	POLE	.	benign(0.002)	tolerated(1)	ENSP00000445753.1:p.Gln1423Glu
CAS-A	DM2	12;133220089;G;C	0.23	missense_variant	POLE	.	benign(0.002)	tolerated(1)	ENSP00000445753.1:p.Gln1423Glu
CAS-A	DM4	12;133220089;G;C	0.21	missense_variant	POLE	.	benign(0.002)	tolerated(1)	ENSP00000445753.1:p.Gln1423Glu
CAS-A	DM3	5;112173567;C;T	0.75	missense_variant	APC	.	probably_damaging(0.999)	deleterious(0)	ENSP00000423828.2:p.Ala759Val
CAS-A	DM1	5;112173567;C;T	0.55	missense_variant	APC	.	probably_damaging(0.999)	deleterious(0)	ENSP00000423828.2:p.Ala759Val
CAS-A	RM2	5;112173567;C;T	0.34	missense_variant	APC	.	probably_damaging(0.999)	deleterious(0)	ENSP00000423828.2:p.Ala759Val
CAS-A	RM1	5;112173567;C;T	0.40	missense_variant	APC	.	probably_damaging(0.999)	deleterious(0)	ENSP00000423828.2:p.Ala759Val
CAS-A	RM3	5;112173567;C;T	0.47	missense_variant	APC	.	probably_damaging(0.999)	deleterious(0)	ENSP00000423828.2:p.Ala759Val
CAS-A	DM2	5;112173567;C;T	0.50	missense_variant	APC	.	probably_damaging(0.999)	deleterious(0)	ENSP00000423828.2:p.Ala759Val
CAS-A	DM4	5;112173567;C;T	0.45	missense_variant	APC	.	probably_damaging(0.999)	deleterious(0)	ENSP00000423828.2:p.Ala759Val
CAS-A	DM3	8;30982117;C;T	0.65	missense_variant	WRN	.	benign(0.003)	tolerated(0.17)	ENSP00000298139.5:p.His904Tyr
CAS-A	DM1	8;30982117;C;T	0.50	missense_variant	WRN	.	benign(0.003)	tolerated(0.17)	ENSP00000298139.5:p.His904Tyr
CAS-A	RM2	8;30982117;C;T	0.49	missense_variant	WRN	.	benign(0.003)	tolerated(0.17)	ENSP00000298139.5:p.His904Tyr
CAS-A	RM1	8;30982117;C;T	0.44	missense_variant	WRN	.	benign(0.003)	tolerated(0.17)	ENSP00000298139.5:p.His904Tyr
CAS-A	RM3	8;30982117;C;T	0.36	missense_variant	WRN	.	benign(0.003)	tolerated(0.17)	ENSP00000298139.5:p.His904Tyr
CAS-A	DM2	8;30982117;C;T	0.50	missense_variant	WRN	.	benign(0.003)	tolerated(0.17)	ENSP00000298139.5:p.His904Tyr
CAS-A	DM4	8;30982117;C;T	0.50	missense_variant	WRN	.	benign(0.003)	tolerated(0.17)	ENSP00000298139.5:p.His904Tyr
CAS-B	RM1	17;7574003;G;A	0.90	stop_gained	TP53	1,107,399,721	.	.	ENSP00000391478.2:p.Arg342Ter
CAS-B	RM2	17;7574003;G;A	0.71	stop_gained	TP53	1,107,399,721	.	.	ENSP00000391478.2:p.Arg342Ter
CAS-B	RM3	17;7574003;G;A	0.84	stop_gained	TP53	1,107,399,721	.	.	ENSP00000391478.2:p.Arg342Ter
CAS-B	DM2	17;7574003;G;A	1.00	stop_gained	TP53	1,107,399,721	.	.	ENSP00000391478.2:p.Arg342Ter
CAS-B	DM3	17;7574003;G;A	1.00	stop_gained	TP53	1,107,399,721	.	.	ENSP00000391478.2:p.Arg342Ter
CAS-B	DM1	17;7574003;G;A	0.99	stop_gained	TP53	1,107,399,721	.	.	ENSP00000391478.2:p.Arg342Ter
CAS-B	DM4	17;7574003;G;A	0.65	stop_gained	TP53	1,107,399,721	.	.	ENSP00000391478.2:p.Arg342Ter
CAS-B	DM5	17;7574003;G;A	0.89	stop_gained	TP53	1,107,399,721	.	.	ENSP00000391478.2:p.Arg342Ter
CAS-B	P	17;7574003;G;A	1.00	stop_gained	TP53	1,107,399,721	.	.	ENSP00000391478.2:p.Arg342Ter
CAS-C	DM3	1;186307212;G;A	0.14	missense_variant	TPR	.	.	deleterious(0.01)	ENSP00000356448.3:p.Arg1439Cys

CAS-C	DM1	2;109380084;C;T	0.50	missense_variant	RANBP2	.	benign(0.002)	tolerated(0.07)	ENSP00000283195.6:p.Pro1030Leu
CAS-C	DM3	2;109380084;C;T	0.49	missense_variant	RANBP2	.	benign(0.002)	tolerated(0.07)	ENSP00000283195.6:p.Pro1030Leu
CAS-C	DM5	2;109380084;C;T	0.50	missense_variant	RANBP2	.	benign(0.002)	tolerated(0.07)	ENSP00000283195.6:p.Pro1030Leu
CAS-C	DM2	2;109380084;C;T	0.50	missense_variant	RANBP2	.	benign(0.002)	tolerated(0.07)	ENSP00000283195.6:p.Pro1030Leu
CAS-C	DM4	2;109380084;C;T	0.49	missense_variant	RANBP2	.	benign(0.002)	tolerated(0.07)	ENSP00000283195.6:p.Pro1030Leu
CAS-C	DM6	2;109380084;C;T	0.38	missense_variant	RANBP2	.	benign(0.002)	tolerated(0.07)	ENSP00000283195.6:p.Pro1030Leu
CAS-C	P	2;109380084;C;T	0.48	missense_variant	RANBP2	.	benign(0.002)	tolerated(0.07)	ENSP00000283195.6:p.Pro1030Leu
CAS-C	DM5	3;41266133;C;G	0.45	missense_variant	CTNNB1	17661	possibly_damaging(0.792)	deleterious(0.02)	ENSP00000401599.1:p.Pro44Ala
CAS-C	DM4	3;41266133;C;G	0.47	missense_variant	CTNNB1	17661	possibly_damaging(0.792)	deleterious(0.02)	ENSP00000401599.1:p.Pro44Ala
CAS-C	DM6	3;41266133;C;G	0.49	missense_variant	CTNNB1	17661	possibly_damaging(0.792)	deleterious(0.02)	ENSP00000401599.1:p.Pro44Ala
CAS-C	DM5	3;41266136;T;C	0.46	missense_variant	CTNNB1	5663	probably_damaging(0.939)	deleterious(0)	ENSP00000401599.1:p.Ser45Pro
CAS-C	DM4	3;41266136;T;C	0.49	missense_variant	CTNNB1	5663	probably_damaging(0.939)	deleterious(0)	ENSP00000401599.1:p.Ser45Pro
CAS-C	DM6	3;41266136;T;C	0.50	missense_variant	CTNNB1	5663	probably_damaging(0.939)	deleterious(0)	ENSP00000401599.1:p.Ser45Pro
CAS-C	DM1	8;30998973;C;T	0.48	missense_variant	WRN	136658	probably_damaging(0.999)	deleterious(0)	ENSP00000298139.5:p.Arg999Cys
CAS-C	DM3	8;30998973;C;T	0.42	missense_variant	WRN	136658	probably_damaging(0.999)	deleterious(0)	ENSP00000298139.5:p.Arg999Cys
CAS-C	DM5	8;30998973;C;T	0.50	missense_variant	WRN	136658	probably_damaging(0.999)	deleterious(0)	ENSP00000298139.5:p.Arg999Cys
CAS-C	DM2	8;30998973;C;T	0.50	missense_variant	WRN	136658	probably_damaging(0.999)	deleterious(0)	ENSP00000298139.5:p.Arg999Cys
CAS-C	DM4	8;30998973;C;T	0.46	missense_variant	WRN	136658	probably_damaging(0.999)	deleterious(0)	ENSP00000298139.5:p.Arg999Cys
CAS-C	DM6	8;30998973;C;T	0.47	missense_variant	WRN	136658	probably_damaging(0.999)	deleterious(0)	ENSP00000298139.5:p.Arg999Cys
CAS-C	P	8;30998973;C;T	0.50	missense_variant	WRN	136658	probably_damaging(0.999)	deleterious(0)	ENSP00000298139.5:p.Arg999Cys
CAS-C	DM1	9;134073641;C;T	0.49	missense_variant	NUP214	.	benign(0.005)	deleterious(0.03)	ENSP00000396576.2:p.Ser1577Phe
CAS-C	DM3	9;134073641;C;T	0.50	missense_variant	NUP214	.	benign(0.005)	deleterious(0.03)	ENSP00000396576.2:p.Ser1577Phe
CAS-C	DM5	9;134073641;C;T	0.43	missense_variant	NUP214	.	benign(0.005)	deleterious(0.03)	ENSP00000396576.2:p.Ser1577Phe
CAS-C	DM2	9;134073641;C;T	0.47	missense_variant	NUP214	.	benign(0.005)	deleterious(0.03)	ENSP00000396576.2:p.Ser1577Phe
CAS-C	DM4	9;134073641;C;T	0.50	missense_variant	NUP214	.	benign(0.005)	deleterious(0.03)	ENSP00000396576.2:p.Ser1577Phe
CAS-C	DM6	9;134073641;C;T	0.50	missense_variant	NUP214	.	benign(0.005)	deleterious(0.03)	ENSP00000396576.2:p.Ser1577Phe
CAS-C	P	9;134073641;C;T	0.50	missense_variant	NUP214	.	benign(0.005)	deleterious(0.03)	ENSP00000396576.2:p.Ser1577Phe
ETH-E	RM	3;14209852;CA;C	0.87	frameshift_variant	XPC	.	.	.	ENSP00000423867.1:p.Val141GlyfsTer14
ETH-E	DM1	3;14209852;CA;C	0.91	frameshift_variant	XPC	.	.	.	ENSP00000423867.1:p.Val141GlyfsTer14
ETH-E	DM2	3;14209852;CA;C	0.84	frameshift_variant	XPC	.	.	.	ENSP00000423867.1:p.Val141GlyfsTer14
ETH-E	DM3	3;14209852;CA;C	0.91	frameshift_variant	XPC	.	.	.	ENSP00000423867.1:p.Val141GlyfsTer14
ETH-E	P	3;14209852;CA;C	0.08	frameshift_variant	XPC	.	.	.	ENSP00000423867.1:p.Val141GlyfsTer14
ETH-F	DM1	1;156106037;G;A	0.43	missense_variant	LMNA	.	benign(0.416)	tolerated(0.15)	ENSP00000357283.4:p.Arg397His
ETH-F	DM2	1;156106037;G;A	0.41	missense_variant	LMNA	.	benign(0.416)	tolerated(0.15)	ENSP00000357283.4:p.Arg397His
ETH-F	DM5	1;156106037;G;A	0.50	missense_variant	LMNA	.	benign(0.416)	tolerated(0.15)	ENSP00000357283.4:p.Arg397His
ETH-F	DM3	1;156106037;G;A	0.36	missense_variant	LMNA	.	benign(0.416)	tolerated(0.15)	ENSP00000357283.4:p.Arg397His
ETH-F	DM4	1;156106037;G;A	0.50	missense_variant	LMNA	.	benign(0.416)	tolerated(0.15)	ENSP00000357283.4:p.Arg397His
ETH-F	P1	1;156106037;G;A	0.67	missense_variant	LMNA	.	benign(0.416)	tolerated(0.15)	ENSP00000357283.4:p.Arg397His
ETH-F	P2	1;156106037;G;A	0.67	missense_variant	LMNA	.	benign(0.416)	tolerated(0.15)	ENSP00000357283.4:p.Arg397His
ETH-F	P3	1;156106037;G;A	0.67	missense_variant	LMNA	.	benign(0.416)	tolerated(0.15)	ENSP00000357283.4:p.Arg397His
MI-F	DM	1;186304261;C;T	0.29	missense_variant	TPR	.	.	tolerated(0.36)	ENSP00000356448.3:p.Val1638Ile
MI-F	RM1	1;186304261;C;T	0.41	missense_variant	TPR	.	.	tolerated(0.36)	ENSP00000356448.3:p.Val1638Ile
MI-F	RM2	1;186304261;C;T	0.38	missense_variant	TPR	.	.	tolerated(0.36)	ENSP00000356448.3:p.Val1638Ile

MI-F	P	1;186304261;C;T	0.33	missense_variant	TPR	.	.	tolerated(0.36)	ENSP00000356448.3:p.Val1638Ile
MI-F	DM	1;186304262;C;T	0.28	splice_acceptor_variant	TPR	.	.	.	.
MI-F	RM1	1;186304262;C;T	0.41	splice_acceptor_variant	TPR	.	.	.	.
MI-F	RM2	1;186304262;C;T	0.39	splice_acceptor_variant	TPR	.	.	.	.
MI-F	P	1;186304262;C;T	0.35	splice_acceptor_variant	TPR	.	.	.	.
MI-F	DM	11;71732301;G;A	0.50	missense_variant	NUMA1	.	benign(0.273)	deleterious(0.02)	ENSP00000444175.1:p.His137Tyr
MI-F	RM1	11;71732301;G;A	0.43	missense_variant	NUMA1	.	benign(0.273)	deleterious(0.02)	ENSP00000444175.1:p.His137Tyr
MI-F	RM2	11;71732301;G;A	0.50	missense_variant	NUMA1	.	benign(0.273)	deleterious(0.02)	ENSP00000444175.1:p.His137Tyr
MI-F	P	11;71732301;G;A	0.42	missense_variant	NUMA1	.	benign(0.273)	deleterious(0.02)	ENSP00000444175.1:p.His137Tyr
MI-F	DM	15;40675107;C;T	0.67	missense_variant	KNSTRN	140056	probably_damaging(0.983)	deleterious(0.01)	ENSP00000453213.1:p.Ser24Phe
MI-F	RM1	15;40675107;C;T	0.38	missense_variant	KNSTRN	140056	probably_damaging(0.983)	deleterious(0.01)	ENSP00000453213.1:p.Ser24Phe
MI-F	RM2	15;40675107;C;T	0.50	missense_variant	KNSTRN	140056	probably_damaging(0.983)	deleterious(0.01)	ENSP00000453213.1:p.Ser24Phe
MI-F	P	15;40675107;C;T	0.19	missense_variant	KNSTRN	140056	probably_damaging(0.983)	deleterious(0.01)	ENSP00000453213.1:p.Ser24Phe
MI-F	DM	16;67694198;G;A	0.50	missense_variant	ACD	135719	possibly_damaging(0.86)	tolerated(0.25)	ENSP00000377496.4:p.Leu62Phe
MI-F	RM1	16;67694198;G;A	0.44	missense_variant	ACD	135719	possibly_damaging(0.86)	tolerated(0.25)	ENSP00000377496.4:p.Leu62Phe
MI-F	RM2	16;67694198;G;A	0.49	missense_variant	ACD	135719	possibly_damaging(0.86)	tolerated(0.25)	ENSP00000377496.4:p.Leu62Phe
MI-F	P	16;67694198;G;A	0.50	missense_variant	ACD	135719	possibly_damaging(0.86)	tolerated(0.25)	ENSP00000377496.4:p.Leu62Phe
MI-F	DM	16;67694228;G;A	0.50	missense_variant	ACD	.	possibly_damaging(0.587)	tolerated(0.3)	ENSP00000377496.4:p.Leu52Phe
MI-F	RM1	16;67694228;G;A	0.42	missense_variant	ACD	.	possibly_damaging(0.587)	tolerated(0.3)	ENSP00000377496.4:p.Leu52Phe
MI-F	RM2	16;67694228;G;A	0.50	missense_variant	ACD	.	possibly_damaging(0.587)	tolerated(0.3)	ENSP00000377496.4:p.Leu52Phe
MI-F	P	16;67694228;G;A	0.47	missense_variant	ACD	.	possibly_damaging(0.587)	tolerated(0.3)	ENSP00000377496.4:p.Leu52Phe
MI-F	DM	16;89825073;G;A	0.48	missense_variant	FANCA	.	probably_damaging(0.984)	tolerated(0.27)	ENSP00000454217.1:p.Pro78Ser
MI-F	RM1	16;89825073;G;A	0.40	missense_variant	FANCA	.	probably_damaging(0.984)	tolerated(0.27)	ENSP00000454217.1:p.Pro78Ser
MI-F	RM2	16;89825073;G;A	0.38	missense_variant	FANCA	.	probably_damaging(0.984)	tolerated(0.27)	ENSP00000454217.1:p.Pro78Ser
MI-F	P	16;89825073;G;A	0.34	missense_variant	FANCA	.	probably_damaging(0.984)	tolerated(0.27)	ENSP00000454217.1:p.Pro78Ser
MI-F	DM	17;59858236;G;A	0.48	missense_variant	BRIP1	.	benign(0.005)	tolerated(1)	ENSP00000464654.1:p.His587Tyr
MI-F	RM1	17;59858236;G;A	0.50	missense_variant	BRIP1	.	benign(0.005)	tolerated(1)	ENSP00000464654.1:p.His587Tyr
MI-F	RM2	17;59858236;G;A	0.49	missense_variant	BRIP1	.	benign(0.005)	tolerated(1)	ENSP00000464654.1:p.His587Tyr
MI-F	P	17;59858236;G;A	0.50	missense_variant	BRIP1	.	benign(0.005)	tolerated(1)	ENSP00000464654.1:p.His587Tyr
MI-F	DM	17;59870969;G;A	0.50	missense_variant	BRIP1	.	benign(0.117)	tolerated(0.25)	ENSP00000464654.1:p.Pro488Ser
MI-F	RM1	17;59870969;G;A	0.50	missense_variant	BRIP1	.	benign(0.117)	tolerated(0.25)	ENSP00000464654.1:p.Pro488Ser
MI-F	RM2	17;59870969;G;A	0.50	missense_variant	BRIP1	.	benign(0.117)	tolerated(0.25)	ENSP00000464654.1:p.Pro488Ser
MI-F	P	17;59870969;G;A	0.45	missense_variant	BRIP1	.	benign(0.117)	tolerated(0.25)	ENSP00000464654.1:p.Pro488Ser
MI-F	DM	17;7578517;G;A	0.50	missense_variant	TP53	43,818,288,785,288,700,000,000,000,000	possibly_damaging(0.661)	deleterious(0.03)	ENSP00000398846.2:p.Ala138Val
MI-F	RM1	17;7578517;G;A	0.37	missense_variant	TP53	43,818,288,785,288,700,000,000,000,000	possibly_damaging(0.661)	deleterious(0.03)	ENSP00000398846.2:p.Ala138Val
MI-F	RM2	17;7578517;G;A	0.39	missense_variant	TP53	43,818,288,785,288,700,000,000,000,000	possibly_damaging(0.661)	deleterious(0.03)	ENSP00000398846.2:p.Ala138Val

MI-F	P	17;7578517;G;A	0.47	missense_variant	TP53	43,818,288,785,288,700,000,000,000,000	possibly_damaging(0.661)	deleterious(0.03)	ENSP00000398846.2:p.Ala138Val
MI-F	DM	5;112154970;G;T	0.39	missense_variant	APC	.	possibly_damaging(0.762)	deleterious(0.02)	ENSP00000423828.2:p.Arg414Leu
MI-F	RM1	5;112154970;G;T	0.48	missense_variant	APC	.	possibly_damaging(0.762)	deleterious(0.02)	ENSP00000423828.2:p.Arg414Leu
MI-F	RM2	5;112154970;G;T	0.35	missense_variant	APC	.	possibly_damaging(0.762)	deleterious(0.02)	ENSP00000423828.2:p.Arg414Leu
MI-F	P	5;112154970;G;T	0.33	missense_variant	APC	.	possibly_damaging(0.762)	deleterious(0.02)	ENSP00000423828.2:p.Arg414Leu
MI-F	DM	5;112170771;C;T	0.33	missense_variant	APC	.	possibly_damaging(0.874)	deleterious(0.01)	ENSP00000473355.1:p.Arg186Trp
MI-F	RM1	5;112170771;C;T	0.43	missense_variant	APC	.	possibly_damaging(0.874)	deleterious(0.01)	ENSP00000473355.1:p.Arg186Trp
MI-F	RM2	5;112170771;C;T	0.47	missense_variant	APC	.	possibly_damaging(0.874)	deleterious(0.01)	ENSP00000473355.1:p.Arg186Trp
MI-F	P	5;112170771;C;T	0.50	missense_variant	APC	.	possibly_damaging(0.874)	deleterious(0.01)	ENSP00000473355.1:p.Arg186Trp
MI-F	DM	6;35427133;C;T	0.11	missense_variant	FANCE	.	probably_damaging(0.999)	deleterious(0)	ENSP00000229769.2:p.Ser380Phe
MI-F	RM1	6;35427133;C;T	0.25	missense_variant	FANCE	.	probably_damaging(0.999)	deleterious(0)	ENSP00000229769.2:p.Ser380Phe
MI-F	RM2	6;35427133;C;T	0.27	missense_variant	FANCE	.	probably_damaging(0.999)	deleterious(0)	ENSP00000229769.2:p.Ser380Phe
MI-F	P	6;35427133;C;T	0.22	missense_variant	FANCE	.	probably_damaging(0.999)	deleterious(0)	ENSP00000229769.2:p.Ser380Phe
MI-F	DM	7;91570420;G;A	0.46	missense_variant	AKAP9	.	probably_damaging(0.999)	deleterious(0)	ENSP00000350813.2:p.Asp3Asn
MI-F	RM1	7;91570420;G;A	0.33	missense_variant	AKAP9	.	probably_damaging(0.999)	deleterious(0)	ENSP00000350813.2:p.Asp3Asn
MI-F	RM2	7;91570420;G;A	0.44	missense_variant	AKAP9	.	probably_damaging(0.999)	deleterious(0)	ENSP00000350813.2:p.Asp3Asn
MI-F	P	7;91570420;G;A	0.50	missense_variant	AKAP9	.	probably_damaging(0.999)	deleterious(0)	ENSP00000350813.2:p.Asp3Asn
MI-F	DM	8;30921942;C;T	0.67	missense_variant	WRN	.	probably_damaging(0.921)	deleterious(0)	ENSP00000298139.5:p.Ser116Phe
MI-F	RM1	8;30921942;C;T	0.50	missense_variant	WRN	.	probably_damaging(0.921)	deleterious(0)	ENSP00000298139.5:p.Ser116Phe
MI-F	RM2	8;30921942;C;T	0.44	missense_variant	WRN	.	probably_damaging(0.921)	deleterious(0)	ENSP00000298139.5:p.Ser116Phe
MI-F	P	8;30921942;C;T	0.50	missense_variant	WRN	.	probably_damaging(0.921)	deleterious(0)	ENSP00000298139.5:p.Ser116Phe
MI-F	DM	9;123874812;A;G	0.41	missense_variant	CNTRL	.	probably_damaging(0.998)	deleterious(0)	ENSP00000238341.5:p.Lys360Glu
MI-F	RM1	9;123874812;A;G	0.45	missense_variant	CNTRL	.	probably_damaging(0.998)	deleterious(0)	ENSP00000238341.5:p.Lys360Glu
MI-F	RM2	9;123874812;A;G	0.41	missense_variant	CNTRL	.	probably_damaging(0.998)	deleterious(0)	ENSP00000238341.5:p.Lys360Glu
MI-F	P	9;123874812;A;G	0.50	missense_variant	CNTRL	.	probably_damaging(0.998)	deleterious(0)	ENSP00000238341.5:p.Lys360Glu
SK-G	RM	15;40505576;T;G	0.49	missense_variant	BUB1B	.	possibly_damaging(0.742)	deleterious(0.01)	ENSP00000287598.6:p.Val860Gly
SK-G	DM	15;40505576;T;G	0.65	missense_variant	BUB1B	.	possibly_damaging(0.742)	deleterious(0.01)	ENSP00000287598.6:p.Val860Gly
SK-G	P	15;40505576;T;G	0.42	missense_variant	BUB1B	.	possibly_damaging(0.742)	deleterious(0.01)	ENSP00000287598.6:p.Val860Gly
SK-G	RM	17;57739005;G;A	0.29	splice_donor_variant	CLTC	.	.	.	.
SK-G	RM	17;59924545;T;A	0.62	missense_variant	BRIP1	.	benign(0.001)	tolerated(0.72)	ENSP00000464654.1:p.Asn182Tyr
SK-G	DM	17;59924545;T;A	0.30	missense_variant	BRIP1	.	benign(0.001)	tolerated(0.72)	ENSP00000464654.1:p.Asn182Tyr
SK-G	P	17;59924545;T;A	0.48	missense_variant	BRIP1	.	benign(0.001)	tolerated(0.72)	ENSP00000464654.1:p.Asn182Tyr
SK-G	RM	17;59924546;G;T	0.61	missense_variant	BRIP1	.	benign(0.002)	tolerated(0.52)	ENSP00000464654.1:p.His181Gln
SK-G	DM	17;59924546;G;T	0.31	missense_variant	BRIP1	.	benign(0.002)	tolerated(0.52)	ENSP00000464654.1:p.His181Gln
SK-G	P	17;59924546;G;T	0.48	missense_variant	BRIP1	.	benign(0.002)	tolerated(0.52)	ENSP00000464654.1:p.His181Gln
SK-H	RM1	1;156085052;G;A	0.10	missense_variant	LMNA	.	benign(0.395)	tolerated(0.11)	ENSP00000357283.4:p.Glu115Lys
SK-H	DM	1;156105807;G;A	0.29	missense_variant	LMNA	.	benign(0.036)	tolerated(0.45)	ENSP00000357283.4:p.Arg351Lys
SK-H	RM2	1;186328986;T;C	0.27	missense_variant	TPR	.	.	deleterious(0.01)	ENSP00000356448.3:p.Glu445Gly
SK-H	P	1;186340104;G;A	0.67	stop_gained	TPR	.	.	.	ENSP00000356448.3:p.Gln110Ter
SK-H	RM1	11;108158333;C;T	0.26	missense_variant	ATM	.	benign(0.169)	tolerated(0.09)	ENSP00000434327.2:p.His3Tyr
SK-H	DM	11;108165675;G;A	0.34	missense_variant	ATM	.	benign(0.059)	tolerated(0.51)	ENSP00000278616.4:p.Val1600Ile

SK-H	RM1	11;71726660;G;A	0.09	missense_variant	NUMA1	.	possibly_damaging(0.61)	tolerated(0.08)	ENSP00000442936.1:p.Ala630Val
SK-H	DM	12;133210778;C;T	0.31	missense_variant	POLE	11,800,141,180,015	benign(0.005)	tolerated(1)	ENSP00000445753.1:p.Val1973Ile
SK-H	DM	13;103527861;A;G	0.42	missense_variant	ERCC5	.	benign(0.293)	tolerated(0.17)	ENSP00000365121.1:p.Lys290Glu
SK-H	DM	15;40488976;G;A	0.38	splice_donor_variant	BUB1B	.	.	.	.
SK-H	RM1	16;23634381;C;T	0.10	missense_variant	PALB2	.	possibly_damaging(0.825)	tolerated(0.06)	ENSP00000261584.4:p.Val969Met
SK-H	RM2	17;41244330;C;T	0.44	missense_variant	BRCA1	.	benign(0.159)	tolerated(0.18)	ENSP00000418960.2:p.Gly1073Asp
SK-H	RM2	17;7578278;G;A	0.29	missense_variant	TP53	43702	possibly_damaging(0.847)	deleterious(0.04)	ENSP00000398846.2:p.Pro191Ser
SK-H	RM2	2;109381175;G;A	0.23	missense_variant	RANBP2	.	benign(0.039)	tolerated(0.21)	ENSP00000283195.6:p.Val1394Ile
SK-H	RM2	2;48030675;C;T	0.42	missense_variant	MSH6	.	probably_damaging(1)	deleterious(0)	ENSP00000446475.1:p.Pro967Ser
SK-H	RM1	2;48030802;G;A	0.28	missense_variant	MSH6	.	probably_damaging(0.998)	deleterious(0)	ENSP00000446475.1:p.Gly1009Asp
SK-H	RM1	22;41574425;G;A	0.09	missense_variant	EP300	.	possibly_damaging(0.886)	tolerated(0.07)	ENSP00000263253.7:p.Gly2237Glu
SK-H	RM1	3;142231107;G;T	0.50	stop_gained	ATR	.	.	.	ENSP00000343741.4:p.Ser1616Ter
SK-H	RM2	3;142231107;G;T	0.59	stop_gained	ATR	.	.	.	ENSP00000343741.4:p.Ser1616Ter
SK-H	RM2	5;112179165;G;A	0.48	missense_variant	APC	.	benign(0.001)	tolerated(0.52)	ENSP00000427089.2:p.Ser2625Asn
SK-H	RM1	5;1268736;C;T	0.05	missense_variant	TERT	.	possibly_damaging(0.618)	deleterious(0)	ENSP00000425003.1:p.Val755Met
SK-H	RM2	5;1293427;C;T	0.08	splice_donor_variant	TERT	.	.	.	.
SK-H	DM	5;1293592;C;T	0.05	missense_variant	TERT	.	possibly_damaging(0.603)	tolerated(0.3)	ENSP00000425003.1:p.Arg470His
SK-H	RM1	7;91712601;G;A	0.10	missense_variant	AKAP9	.	benign(0.047)	tolerated(0.21)	ENSP00000350813.2:p.Glu2772Lys
SK-H	DM	7;91714177;C;T	0.13	stop_gained	AKAP9	.	.	.	ENSP00000378042.2:p.Gln765Ter
SK-H	RM2	7;91718700;G;A	0.11	missense_variant	AKAP9	.	benign(0.234)	tolerated(1)	ENSP00000350813.2:p.Gly3022Asp

Supplementary Table 11. Mutations gained or increased in allelic dose with a genome doubling event for patients CAS-G, CAS-D, CAS-C and ETH-E.

Patient	Sample	Variant	MAF	Mutation Type	Gene	COSMIC	Polyphen2	SIFT	AA Change
CAS-G	DM1	12;40076528;C;T	0.51	missense variant	C12orf40	.	possibly damaging (0.878)	deleterious (0.03)	ENSP00000473371.1:p.His191Tyr
CAS-G	DM2	12;40076528;C;T	0.67	missense variant	C12orf40	.	possibly damaging (0.878)	deleterious (0.03)	ENSP00000473371.1:p.His191Tyr
CAS-G	DM3	12;40076528;C;T	0.57	missense variant	C12orf40	.	possibly damaging (0.878)	deleterious (0.03)	ENSP00000473371.1:p.His191Tyr
CAS-G	DM4	12;40076528;C;T	0.63	missense variant	C12orf40	.	possibly damaging (0.878)	deleterious (0.03)	ENSP00000473371.1:p.His191Tyr
CAS-G	DM5	12;40076528;C;T	0.50	missense variant	C12orf40	.	possibly damaging (0.878)	deleterious (0.03)	ENSP00000473371.1:p.His191Tyr
CAS-G	DM6	12;40076528;C;T	0.67	missense variant	C12orf40	.	possibly damaging (0.878)	deleterious (0.03)	ENSP00000473371.1:p.His191Tyr
CAS-G	P	12;40076528;C;T	0.02	missense variant	C12orf40	.	possibly damaging (0.878)	deleterious (0.03)	ENSP00000473371.1:p.His191Tyr
CAS-G	RM	12;40076528;C;T	0.005	missense variant	C12orf40	.	possibly damaging (0.878)	deleterious (0.03)	ENSP00000473371.1:p.His191Tyr
CAS-G	DM1	1;75097441;G;A	0.61	missense variant	C1orf173	912103	benign (0.296)	deleterious (0)	ENSP00000473404.1:p.Arg62Cys
CAS-G	DM2	1;75097441;G;A	0.59	missense variant	C1orf173	912103	benign (0.296)	deleterious (0)	ENSP00000473404.1:p.Arg62Cys
CAS-G	DM3	1;75097441;G;A	0.61	missense variant	C1orf173	912103	benign (0.296)	deleterious (0)	ENSP00000473404.1:p.Arg62Cys
CAS-G	DM4	1;75097441;G;A	0.67	missense variant	C1orf173	912103	benign (0.296)	deleterious (0)	ENSP00000473404.1:p.Arg62Cys
CAS-G	DM5	1;75097441;G;A	0.67	missense variant	C1orf173	912103	benign (0.296)	deleterious (0)	ENSP00000473404.1:p.Arg62Cys
CAS-G	DM6	1;75097441;G;A	0.60	missense variant	C1orf173	912103	benign (0.296)	deleterious (0)	ENSP00000473404.1:p.Arg62Cys
CAS-G	P	1;75097441;G;A	0.34	missense variant	C1orf173	912103	benign (0.296)	deleterious (0)	ENSP00000473404.1:p.Arg62Cys
CAS-G	RM	1;75097441;G;A	0.28	missense variant	C1orf173	912103	benign (0.296)	deleterious (0)	ENSP00000473404.1:p.Arg62Cys
CAS-G	DM1	1;155013029;C;T	0.50	stop gained	DCST1	.	.	.	ENSP00000295542.1:p.Gln154Ter
CAS-G	DM2	1;155013029;C;T	0.50	stop gained	DCST1	.	.	.	ENSP00000295542.1:p.Gln154Ter
CAS-G	DM3	1;155013029;C;T	0.50	stop gained	DCST1	.	.	.	ENSP00000295542.1:p.Gln154Ter
CAS-G	DM4	1;155013029;C;T	0.42	stop gained	DCST1	.	.	.	ENSP00000295542.1:p.Gln154Ter
CAS-G	DM5	1;155013029;C;T	0.50	stop gained	DCST1	.	.	.	ENSP00000295542.1:p.Gln154Ter
CAS-G	DM6	1;155013029;C;T	0.50	stop gained	DCST1	.	.	.	ENSP00000295542.1:p.Gln154Ter
CAS-G	DM1	11;6561242;C;A	0.28	missense variant	DNHD1	.	possibly damaging (0.804)	deleterious (0.01)	ENSP00000254579.6:p.Ser1186Tyr

CAS-G	DM2	11;6561242;C;A	0.28	missense variant	DNHD1	.	possibly damaging (0.804)	deleterious (0.01)	ENSP00000254579.6:p.Ser1186Tyr
CAS-G	DM3	11;6561242;C;A	0.24	missense variant	DNHD1	.	possibly damaging (0.804)	deleterious (0.01)	ENSP00000254579.6:p.Ser1186Tyr
CAS-G	DM4	11;6561242;C;A	0.31	missense variant	DNHD1	.	possibly damaging (0.804)	deleterious (0.01)	ENSP00000254579.6:p.Ser1186Tyr
CAS-G	DM5	11;6561242;C;A	0.26	missense variant	DNHD1	.	possibly damaging (0.804)	deleterious (0.01)	ENSP00000254579.6:p.Ser1186Tyr
CAS-G	DM6	11;6561242;C;A	0.32	missense variant	DNHD1	.	possibly damaging (0.804)	deleterious (0.01)	ENSP00000254579.6:p.Ser1186Tyr
CAS-G	DM1	12;132445369;G;T	0.49	missense variant	EP400	.	unknown (0)	deleterious (0)	ENSP00000330620.6:p.Gly69Trp
CAS-G	DM2	12;132445369;G;T	0.44	missense variant	EP400	.	unknown (0)	deleterious (0)	ENSP00000330620.6:p.Gly69Trp
CAS-G	DM3	12;132445369;G;T	0.36	missense variant	EP400	.	unknown (0)	deleterious (0)	ENSP00000330620.6:p.Gly69Trp
CAS-G	DM4	12;132445369;G;T	0.28	missense variant	EP400	.	unknown (0)	deleterious (0)	ENSP00000330620.6:p.Gly69Trp
CAS-G	DM5	12;132445369;G;T	0.44	missense variant	EP400	.	unknown (0)	deleterious (0)	ENSP00000330620.6:p.Gly69Trp
CAS-G	DM6	12;132445369;G;T	0.44	missense variant	EP400	.	unknown (0)	deleterious (0)	ENSP00000330620.6:p.Gly69Trp
CAS-G	DM1	8;124998329;G;A	0.60	missense variant	FER1L6	.	benign (0.184)	deleterious (0.01)	ENSP00000381982.1:p.Glu478Lys
CAS-G	DM2	8;124998329;G;A	0.54	missense variant	FER1L6	.	benign (0.184)	deleterious (0.01)	ENSP00000381982.1:p.Glu478Lys
CAS-G	DM3	8;124998329;G;A	0.47	missense variant	FER1L6	.	benign (0.184)	deleterious (0.01)	ENSP00000381982.1:p.Glu478Lys
CAS-G	DM4	8;124998329;G;A	0.53	missense variant	FER1L6	.	benign (0.184)	deleterious (0.01)	ENSP00000381982.1:p.Glu478Lys
CAS-G	DM5	8;124998329;G;A	0.49	missense variant	FER1L6	.	benign (0.184)	deleterious (0.01)	ENSP00000381982.1:p.Glu478Lys
CAS-G	DM6	8;124998329;G;A	0.55	missense variant	FER1L6	.	benign (0.184)	deleterious (0.01)	ENSP00000381982.1:p.Glu478Lys
CAS-G	P	8;124998329;G;A	0.28	missense variant	FER1L6	.	benign (0.184)	deleterious (0.01)	ENSP00000381982.1:p.Glu478Lys
CAS-G	RM	8;124998329;G;A	0.30	missense variant	FER1L6	.	benign (0.184)	deleterious (0.01)	ENSP00000381982.1:p.Glu478Lys
CAS-G	DM1	13;32841312;G;A	0.28	missense variant	FRY	.	benign (0.017)	tolerated (0.69)	ENSP00000369600.3:p.Gly2651Glu
CAS-G	DM2	13;32841312;G;A	0.30	missense variant	FRY	.	benign (0.017)	tolerated (0.69)	ENSP00000369600.3:p.Gly2651Glu
CAS-G	DM3	13;32841312;G;A	0.25	missense variant	FRY	.	benign (0.017)	tolerated (0.69)	ENSP00000369600.3:p.Gly2651Glu
CAS-G	DM4	13;32841312;G;A	0.32	missense variant	FRY	.	benign (0.017)	tolerated (0.69)	ENSP00000369600.3:p.Gly2651Glu
CAS-G	DM5	13;32841312;G;A	0.41	missense variant	FRY	.	benign (0.017)	tolerated (0.69)	ENSP00000369600.3:p.Gly2651Glu
CAS-G	DM6	13;32841312;G;A	0.34	missense variant	FRY	.	benign (0.017)	tolerated (0.69)	ENSP00000369600.3:p.Gly2651Glu
CAS-G	DM1	7;150439446;G;A	0.61	stop gained	GIMAP5	.	.	.	ENSP00000351473.3:p.Trp73Ter
CAS-G	DM2	7;150439446;G;A	0.63	stop gained	GIMAP5	.	.	.	ENSP00000351473.3:p.Trp73Ter
CAS-G	DM3	7;150439446;G;A	0.63	stop gained	GIMAP5	.	.	.	ENSP00000351473.3:p.Trp73Ter

CAS-G	DM4	7;150439446;G;A	0.63	stop gained	GIMAP5	.	.	.	ENSP00000351473.3:p.Trp73Ter
CAS-G	DM5	7;150439446;G;A	0.59	stop gained	GIMAP5	.	.	.	ENSP00000351473.3:p.Trp73Ter
CAS-G	DM6	7;150439446;G;A	0.57	stop gained	GIMAP5	.	.	.	ENSP00000351473.3:p.Trp73Ter
CAS-G	P	7;150439446;G;A	0.44	stop gained	GIMAP5	.	.	.	ENSP00000351473.3:p.Trp73Ter
CAS-G	RM	7;150439446;G;A	0.44	stop gained	GIMAP5	.	.	.	ENSP00000351473.3:p.Trp73Ter
CAS-G	DM1	5;35876232;G;T	0.34	missense variant	IL7R	.	probably damaging (0.965)	deleterious (0)	ENSP00000306157.3:p.Gly342Trp
CAS-G	DM2	5;35876232;G;T	0.32	missense variant	IL7R	.	probably damaging (0.965)	deleterious (0)	ENSP00000306157.3:p.Gly342Trp
CAS-G	DM3	5;35876232;G;T	0.32	missense variant	IL7R	.	probably damaging (0.965)	deleterious (0)	ENSP00000306157.3:p.Gly342Trp
CAS-G	DM4	5;35876232;G;T	0.33	missense variant	IL7R	.	probably damaging (0.965)	deleterious (0)	ENSP00000306157.3:p.Gly342Trp
CAS-G	DM5	5;35876232;G;T	0.37	missense variant	IL7R	.	probably damaging (0.965)	deleterious (0)	ENSP00000306157.3:p.Gly342Trp
CAS-G	DM6	5;35876232;G;T	0.33	missense variant	IL7R	.	probably damaging (0.965)	deleterious (0)	ENSP00000306157.3:p.Gly342Trp
CAS-G	DM1	4;6107610;G;A	0.26	missense variant	JAKMIP1	.	probably damaging (0.993)	deleterious (0.03)	ENSP00000282924.5:p.Leu72Phe
CAS-G	DM2	4;6107610;G;A	0.36	missense variant	JAKMIP1	.	probably damaging (0.993)	deleterious (0.03)	ENSP00000282924.5:p.Leu72Phe
CAS-G	DM3	4;6107610;G;A	0.26	missense variant	JAKMIP1	.	probably damaging (0.993)	deleterious (0.03)	ENSP00000282924.5:p.Leu72Phe
CAS-G	DM4	4;6107610;G;A	0.22	missense variant	JAKMIP1	.	probably damaging (0.993)	deleterious (0.03)	ENSP00000282924.5:p.Leu72Phe
CAS-G	DM5	4;6107610;G;A	0.22	missense variant	JAKMIP1	.	probably damaging (0.993)	deleterious (0.03)	ENSP00000282924.5:p.Leu72Phe
CAS-G	DM6	4;6107610;G;A	0.26	missense variant	JAKMIP1	.	probably damaging (0.993)	deleterious (0.03)	ENSP00000282924.5:p.Leu72Phe
CAS-G	DM1	X;8502447;C;T	0.67	missense variant	KAL1	.	possibly damaging (0.609)	tolerated (0.05)	ENSP00000262648.3:p.Glu633Lys
CAS-G	DM2	X;8502447;C;T	0.67	missense variant	KAL1	.	possibly damaging (0.609)	tolerated (0.05)	ENSP00000262648.3:p.Glu633Lys
CAS-G	DM3	X;8502447;C;T	0.61	missense variant	KAL1	.	possibly damaging (0.609)	tolerated (0.05)	ENSP00000262648.3:p.Glu633Lys
CAS-G	DM4	X;8502447;C;T	0.62	missense variant	KAL1	.	possibly damaging (0.609)	tolerated (0.05)	ENSP00000262648.3:p.Glu633Lys
CAS-G	DM5	X;8502447;C;T	0.59	missense variant	KAL1	.	possibly damaging (0.609)	tolerated (0.05)	ENSP00000262648.3:p.Glu633Lys
CAS-G	DM6	X;8502447;C;T	0.66	missense variant	KAL1	.	possibly damaging (0.609)	tolerated (0.05)	ENSP00000262648.3:p.Glu633Lys
CAS-G	P	X;8502447;C;T	0.45	missense variant	KAL1	.	possibly damaging (0.609)	tolerated (0.05)	ENSP00000262648.3:p.Glu633Lys
CAS-G	RM	X;8502447;C;T	0.46	missense variant	KAL1	.	possibly damaging (0.609)	tolerated (0.05)	ENSP00000262648.3:p.Glu633Lys
CAS-G	DM1	5;137753192;A;T	0.32	missense variant	KDM3B	.	benign (0.056)	tolerated (0.3)	ENSP00000326563.5:p.Met1110Leu
CAS-G	DM2	5;137753192;A;T	0.27	missense variant	KDM3B	.	benign (0.056)	tolerated (0.3)	ENSP00000326563.5:p.Met1110Leu
CAS-G	DM3	5;137753192;A;T	0.27	missense variant	KDM3B	.	benign (0.056)	tolerated (0.3)	ENSP00000326563.5:p.Met1110Leu

CAS-G	DM4	5;137753192;A;T	0.27	missense variant	KDM3B	.	benign (0.056)	tolerated (0.3)	ENSP00000326563.5:p.Met1110Leu
CAS-G	DM5	5;137753192;A;T	0.26	missense variant	KDM3B	.	benign (0.056)	tolerated (0.3)	ENSP00000326563.5:p.Met1110Leu
CAS-G	DM6	5;137753192;A;T	0.28	missense variant	KDM3B	.	benign (0.056)	tolerated (0.3)	ENSP00000326563.5:p.Met1110Leu
CAS-G	DM1	19;54760019;G;T	0.17	missense variant	LILRB5	.	possibly damaging (0.497)	deleterious (0.05)	ENSP00000320390.5:p.Pro181His
CAS-G	DM2	19;54760019;G;T	0.21	missense variant	LILRB5	.	possibly damaging (0.497)	deleterious (0.05)	ENSP00000320390.5:p.Pro181His
CAS-G	DM3	19;54760019;G;T	0.25	missense variant	LILRB5	.	possibly damaging (0.497)	deleterious (0.05)	ENSP00000320390.5:p.Pro181His
CAS-G	DM4	19;54760019;G;T	0.30	missense variant	LILRB5	.	possibly damaging (0.497)	deleterious (0.05)	ENSP00000320390.5:p.Pro181His
CAS-G	DM5	19;54760019;G;T	0.25	missense variant	LILRB5	.	possibly damaging (0.497)	deleterious (0.05)	ENSP00000320390.5:p.Pro181His
CAS-G	DM6	19;54760019;G;T	0.17	missense variant	LILRB5	.	possibly damaging (0.497)	deleterious (0.05)	ENSP00000320390.5:p.Pro181His
CAS-G	DM1	6;105474276;G;A	0.67	missense variant	LIN28B	.	probably damaging (0.977)	deleterious (0.01)	ENSP00000344401.4:p.Gly101Glu
CAS-G	DM2	6;105474276;G;A	0.63	missense variant	LIN28B	.	probably damaging (0.977)	deleterious (0.01)	ENSP00000344401.4:p.Gly101Glu
CAS-G	DM3	6;105474276;G;A	0.63	missense variant	LIN28B	.	probably damaging (0.977)	deleterious (0.01)	ENSP00000344401.4:p.Gly101Glu
CAS-G	DM4	6;105474276;G;A	0.67	missense variant	LIN28B	.	probably damaging (0.977)	deleterious (0.01)	ENSP00000344401.4:p.Gly101Glu
CAS-G	DM5	6;105474276;G;A	0.67	missense variant	LIN28B	.	probably damaging (0.977)	deleterious (0.01)	ENSP00000344401.4:p.Gly101Glu
CAS-G	DM6	6;105474276;G;A	0.64	missense variant	LIN28B	.	probably damaging (0.977)	deleterious (0.01)	ENSP00000344401.4:p.Gly101Glu
CAS-G	P	6;105474276;G;A	0.50	missense variant	LIN28B	.	probably damaging (0.977)	deleterious (0.01)	ENSP00000344401.4:p.Gly101Glu
CAS-G	RM	6;105474276;G;A	0.41	missense variant	LIN28B	.	probably damaging (0.977)	deleterious (0.01)	ENSP00000344401.4:p.Gly101Glu
CAS-G	DM1	7;141736632;C;T	0.67	missense variant	MGAM	.	probably damaging (0.997)	deleterious (0)	ENSP00000447378.2:p.Pro696Ser
CAS-G	DM2	7;141736632;C;T	0.67	missense variant	MGAM	.	probably damaging (0.997)	deleterious (0)	ENSP00000447378.2:p.Pro696Ser
CAS-G	DM3	7;141736632;C;T	0.63	missense variant	MGAM	.	probably damaging (0.997)	deleterious (0)	ENSP00000447378.2:p.Pro696Ser
CAS-G	DM4	7;141736632;C;T	0.61	missense variant	MGAM	.	probably damaging (0.997)	deleterious (0)	ENSP00000447378.2:p.Pro696Ser
CAS-G	DM5	7;141736632;C;T	0.60	missense variant	MGAM	.	probably damaging (0.997)	deleterious (0)	ENSP00000447378.2:p.Pro696Ser
CAS-G	DM6	7;141736632;C;T	0.67	missense variant	MGAM	.	probably damaging (0.997)	deleterious (0)	ENSP00000447378.2:p.Pro696Ser
CAS-G	P	7;141736632;C;T	0.49	missense variant	MGAM	.	probably damaging (0.997)	deleterious (0)	ENSP00000447378.2:p.Pro696Ser
CAS-G	RM	7;141736632;C;T	0.38	missense variant	MGAM	.	probably damaging (0.997)	deleterious (0)	ENSP00000447378.2:p.Pro696Ser
CAS-G	DM1	8;16885128;C;T	0.67	missense variant	MICU3	.	benign (0.249)	deleterious (0)	ENSP00000321455.5:p.Arg114Trp
CAS-G	DM2	8;16885128;C;T	0.67	missense variant	MICU3	.	benign (0.249)	deleterious (0)	ENSP00000321455.5:p.Arg114Trp
CAS-G	DM3	8;16885128;C;T	0.67	missense variant	MICU3	.	benign (0.249)	deleterious (0)	ENSP00000321455.5:p.Arg114Trp

CAS-G	DM4	8;16885128;C;T	0.67	missense variant	MICU3	.	benign (0.249)	deleterious (0)	ENSP00000321455.5:p.Arg114Trp
CAS-G	DM5	8;16885128;C;T	0.67	missense variant	MICU3	.	benign (0.249)	deleterious (0)	ENSP00000321455.5:p.Arg114Trp
CAS-G	DM6	8;16885128;C;T	0.67	missense variant	MICU3	.	benign (0.249)	deleterious (0)	ENSP00000321455.5:p.Arg114Trp
CAS-G	P	8;16885128;C;T	0.47	missense variant	MICU3	.	benign (0.249)	deleterious (0)	ENSP00000321455.5:p.Arg114Trp
CAS-G	RM	8;16885128;C;T	0.49	missense variant	MICU3	.	benign (0.249)	deleterious (0)	ENSP00000321455.5:p.Arg114Trp
CAS-G	DM1	2;242794820;G;A	0.53	missense variant	PDCD1	.	possibly damaging (0.851)	deleterious (0.01)	ENSP00000340808.3:p.Pro74Leu
CAS-G	DM2	2;242794820;G;A	0.50	missense variant	PDCD1	.	possibly damaging (0.851)	deleterious (0.01)	ENSP00000340808.3:p.Pro74Leu
CAS-G	DM3	2;242794820;G;A	0.49	missense variant	PDCD1	.	possibly damaging (0.851)	deleterious (0.01)	ENSP00000340808.3:p.Pro74Leu
CAS-G	DM4	2;242794820;G;A	0.58	missense variant	PDCD1	.	possibly damaging (0.851)	deleterious (0.01)	ENSP00000340808.3:p.Pro74Leu
CAS-G	DM5	2;242794820;G;A	0.50	missense variant	PDCD1	.	possibly damaging (0.851)	deleterious (0.01)	ENSP00000340808.3:p.Pro74Leu
CAS-G	DM6	2;242794820;G;A	0.48	missense variant	PDCD1	.	possibly damaging (0.851)	deleterious (0.01)	ENSP00000340808.3:p.Pro74Leu
CAS-G	P	2;242794820;G;A	0.32	missense variant	PDCD1	.	possibly damaging (0.851)	deleterious (0.01)	ENSP00000340808.3:p.Pro74Leu
CAS-G	RM	2;242794820;G;A	0.32	missense variant	PDCD1	.	possibly damaging (0.851)	deleterious (0.01)	ENSP00000340808.3:p.Pro74Leu
CAS-G	DM1	7;103290772;G;A	0.40	missense variant	RELN	.	probably damaging (0.999)	deleterious (0)	ENSP00000345694.5:p.Arg651Cys
CAS-G	DM2	7;103290772;G;A	0.36	missense variant	RELN	.	probably damaging (0.999)	deleterious (0)	ENSP00000345694.5:p.Arg651Cys
CAS-G	DM3	7;103290772;G;A	0.33	missense variant	RELN	.	probably damaging (0.999)	deleterious (0)	ENSP00000345694.5:p.Arg651Cys
CAS-G	DM4	7;103290772;G;A	0.31	missense variant	RELN	.	probably damaging (0.999)	deleterious (0)	ENSP00000345694.5:p.Arg651Cys
CAS-G	DM5	7;103290772;G;A	0.42	missense variant	RELN	.	probably damaging (0.999)	deleterious (0)	ENSP00000345694.5:p.Arg651Cys
CAS-G	DM6	7;103290772;G;A	0.38	missense variant	RELN	.	probably damaging (0.999)	deleterious (0)	ENSP00000345694.5:p.Arg651Cys
CAS-G	P	7;103290772;G;A	0.08	missense variant	RELN	.	probably damaging (0.999)	deleterious (0)	ENSP00000345694.5:p.Arg651Cys
CAS-G	P	7;103290772;G;A	0.005	missense variant	RELN	.	probably damaging (0.999)	deleterious (0)	ENSP00000345694.5:p.Arg651Cys
CAS-G	DM6	16;82034421;C;T	0.23	missense variant	SDR42E1	.	probably damaging (1)	deleterious (0)	ENSP00000332407.4:p.Gly15Arg
CAS-G	P	16;82034421;C;T	0.11	missense variant	SDR42E1	.	probably damaging (1)	deleterious (0)	ENSP00000332407.4:p.Gly15Arg
CAS-G	DM1	19;37005354;G;C	0.20	missense variant	ZNF260	.	probably damaging (0.998)	deleterious (0)	ENSP00000465834.1:p.Leu263Val
CAS-G	DM2	19;37005354;G;C	0.25	missense variant	ZNF260	.	probably damaging (0.998)	deleterious (0)	ENSP00000465834.1:p.Leu263Val
CAS-G	DM3	19;37005354;G;C	0.16	missense variant	ZNF260	.	probably damaging (0.998)	deleterious (0)	ENSP00000465834.1:p.Leu263Val
CAS-G	DM4	19;37005354;G;C	0.26	missense variant	ZNF260	.	probably damaging (0.998)	deleterious (0)	ENSP00000465834.1:p.Leu263Val
CAS-G	DM5	19;37005354;G;C	0.21	missense variant	ZNF260	.	probably damaging (0.998)	deleterious (0)	ENSP00000465834.1:p.Leu263Val

CAS-G	DM6	19;37005354;G;C	0.16	missense variant	ZNF260	.	probably damaging (0.998)	deleterious (0)	ENSP00000465834.1:p.Leu263Val
CAS-D	DM1	17;35518730;G;A	1.00	missense variant	ACACA	.	benign (0.155)	deleterious (0.04)	ENSP00000377928.2:p.His1735Tyr
CAS-D	DM2	17;35518730;G;A	1.00	missense variant	ACACA	.	benign (0.155)	deleterious (0.04)	ENSP00000377928.2:p.His1735Tyr
CAS-D	DM3	17;35518730;G;A	1.00	missense variant	ACACA	.	benign (0.155)	deleterious (0.04)	ENSP00000377928.2:p.His1735Tyr
CAS-D	DM4	17;35518730;G;A	1.00	missense variant	ACACA	.	benign (0.155)	deleterious (0.04)	ENSP00000377928.2:p.His1735Tyr
CAS-D	DM5	17;35518730;G;A	0.91	missense variant	ACACA	.	benign (0.155)	deleterious (0.04)	ENSP00000377928.2:p.His1735Tyr
CAS-D	DM6	17;35518730;G;A	1.00	missense variant	ACACA	.	benign (0.155)	deleterious (0.04)	ENSP00000377928.2:p.His1735Tyr
CAS-D	P1	17;35518730;G;A	0.60	missense variant	ACACA	.	benign (0.155)	deleterious (0.04)	ENSP00000377928.2:p.His1735Tyr
CAS-D	P2	17;35518730;G;A	1.00	missense variant	ACACA	.	benign (0.155)	deleterious (0.04)	ENSP00000377928.2:p.His1735Tyr
CAS-D	P3	17;35518730;G;A	0.67	missense variant	ACACA	.	benign (0.155)	deleterious (0.04)	ENSP00000377928.2:p.His1735Tyr
CAS-D	RM	17;35518730;G;A	1.00	missense variant	ACACA	.	benign (0.155)	deleterious (0.04)	ENSP00000377928.2:p.His1735Tyr
CAS-D	DM1	2;27805006;C;T	1.00	missense variant	C2orf16	.	unknown (0)	deleterious (0.03)	ENSP00000386190.2:p.Pro1856Leu
CAS-D	DM2	2;27805006;C;T	1.00	missense variant	C2orf16	.	unknown (0)	deleterious (0.03)	ENSP00000386190.2:p.Pro1856Leu
CAS-D	DM3	2;27805006;C;T	1.00	missense variant	C2orf16	.	unknown (0)	deleterious (0.03)	ENSP00000386190.2:p.Pro1856Leu
CAS-D	DM4	2;27805006;C;T	0.92	missense variant	C2orf16	.	unknown (0)	deleterious (0.03)	ENSP00000386190.2:p.Pro1856Leu
CAS-D	DM5	2;27805006;C;T	0.93	missense variant	C2orf16	.	unknown (0)	deleterious (0.03)	ENSP00000386190.2:p.Pro1856Leu
CAS-D	DM6	2;27805006;C;T	1.00	missense variant	C2orf16	.	unknown (0)	deleterious (0.03)	ENSP00000386190.2:p.Pro1856Leu
CAS-D	P1	2;27805006;C;T	0.78	missense variant	C2orf16	.	unknown (0)	deleterious (0.03)	ENSP00000386190.2:p.Pro1856Leu
CAS-D	P2	2;27805006;C;T	1.00	missense variant	C2orf16	.	unknown (0)	deleterious (0.03)	ENSP00000386190.2:p.Pro1856Leu
CAS-D	P3	2;27805006;C;T	0.78	missense variant	C2orf16	.	unknown (0)	deleterious (0.03)	ENSP00000386190.2:p.Pro1856Leu
CAS-D	RM	2;27805006;C;T	1.00	missense variant	C2orf16	.	unknown (0)	deleterious (0.03)	ENSP00000386190.2:p.Pro1856Leu
CAS-D	DM1	3;121994763;G;T	0.94	missense variant	CASR	.	probably damaging (1)	deleterious (0)	ENSP00000418685.1:p.Trp494Cys
CAS-D	DM2	3;121994763;G;T	0.98	missense variant	CASR	.	probably damaging (1)	deleterious (0)	ENSP00000418685.1:p.Trp494Cys
CAS-D	DM3	3;121994763;G;T	0.94	missense variant	CASR	.	probably damaging (1)	deleterious (0)	ENSP00000418685.1:p.Trp494Cys
CAS-D	DM4	3;121994763;G;T	1.00	missense variant	CASR	.	probably damaging (1)	deleterious (0)	ENSP00000418685.1:p.Trp494Cys
CAS-D	DM5	3;121994763;G;T	0.88	missense variant	CASR	.	probably damaging (1)	deleterious (0)	ENSP00000418685.1:p.Trp494Cys
CAS-D	DM6	3;121994763;G;T	1.00	missense variant	CASR	.	probably damaging (1)	deleterious (0)	ENSP00000418685.1:p.Trp494Cys
CAS-D	P1	3;121994763;G;T	0.44	missense variant	CASR	.	probably damaging (1)	deleterious (0)	ENSP00000418685.1:p.Trp494Cys

CAS-D	P2	3;121994763;G;T	0.67	missense variant	CASR	.	probably damaging (1)	deleterious (0)	ENSP00000418685.1:p.Trp494Cys
CAS-D	P3	3;121994763;G;T	0.22	missense variant	CASR	.	probably damaging (1)	deleterious (0)	ENSP00000418685.1:p.Trp494Cys
CAS-D	RM	3;121994763;G;T	0.78	missense variant	CASR	.	probably damaging (1)	deleterious (0)	ENSP00000418685.1:p.Trp494Cys
CAS-D	DM1	8;642583;G;C	1.00	missense variant	ERICH1	.	benign (0.437)	deleterious (0.01)	ENSP00000262109.7:p.Arg67Gly
CAS-D	DM2	8;642583;G;C	0.79	missense variant	ERICH1	.	benign (0.437)	deleterious (0.01)	ENSP00000262109.7:p.Arg67Gly
CAS-D	DM3	8;642583;G;C	1.00	missense variant	ERICH1	.	benign (0.437)	deleterious (0.01)	ENSP00000262109.7:p.Arg67Gly
CAS-D	DM4	8;642583;G;C	1.00	missense variant	ERICH1	.	benign (0.437)	deleterious (0.01)	ENSP00000262109.7:p.Arg67Gly
CAS-D	DM5	8;642583;G;C	0.70	missense variant	ERICH1	.	benign (0.437)	deleterious (0.01)	ENSP00000262109.7:p.Arg67Gly
CAS-D	DM6	8;642583;G;C	1.00	missense variant	ERICH1	.	benign (0.437)	deleterious (0.01)	ENSP00000262109.7:p.Arg67Gly
CAS-D	P1	8;642583;G;C	0.50	missense variant	ERICH1	.	benign (0.437)	deleterious (0.01)	ENSP00000262109.7:p.Arg67Gly
CAS-D	P2	8;642583;G;C	0.84	missense variant	ERICH1	.	benign (0.437)	deleterious (0.01)	ENSP00000262109.7:p.Arg67Gly
CAS-D	P3	8;642583;G;C	0.35	missense variant	ERICH1	.	benign (0.437)	deleterious (0.01)	ENSP00000262109.7:p.Arg67Gly
CAS-D	RM	8;642583;G;C	0.89	missense variant	ERICH1	.	benign (0.437)	deleterious (0.01)	ENSP00000262109.7:p.Arg67Gly
CAS-D	DM1	3;120360531;G;A	0.79	missense variant	HGD	.	probably damaging (0.974)	deleterious (0)	ENSP00000283871.5:p.Pro262Ser
CAS-D	DM2	3;120360531;G;A	1.00	missense variant	HGD	.	probably damaging (0.974)	deleterious (0)	ENSP00000283871.5:p.Pro262Ser
CAS-D	DM3	3;120360531;G;A	1.00	missense variant	HGD	.	probably damaging (0.974)	deleterious (0)	ENSP00000283871.5:p.Pro262Ser
CAS-D	DM4	3;120360531;G;A	1.00	missense variant	HGD	.	probably damaging (0.974)	deleterious (0)	ENSP00000283871.5:p.Pro262Ser
CAS-D	DM5	3;120360531;G;A	1.00	missense variant	HGD	.	probably damaging (0.974)	deleterious (0)	ENSP00000283871.5:p.Pro262Ser
CAS-D	DM6	3;120360531;G;A	0.98	missense variant	HGD	.	probably damaging (0.974)	deleterious (0)	ENSP00000283871.5:p.Pro262Ser
CAS-D	P1	3;120360531;G;A	0.38	missense variant	HGD	.	probably damaging (0.974)	deleterious (0)	ENSP00000283871.5:p.Pro262Ser
CAS-D	P2	3;120360531;G;A	0.49	missense variant	HGD	.	probably damaging (0.974)	deleterious (0)	ENSP00000283871.5:p.Pro262Ser
CAS-D	P3	3;120360531;G;A	0.34	missense variant	HGD	.	probably damaging (0.974)	deleterious (0)	ENSP00000283871.5:p.Pro262Ser
CAS-D	RM	3;120360531;G;A	0.89	missense variant	HGD	.	probably damaging (0.974)	deleterious (0)	ENSP00000283871.5:p.Pro262Ser
CAS-D	DM1	X;3242180;G;A	0.99	missense variant	MXRA5	.	probably damaging (1)	deleterious (0.01)	ENSP00000217939.5:p.Pro516Ser
CAS-D	DM2	X;3242180;G;A	1.00	missense variant	MXRA5	.	probably damaging (1)	deleterious (0.01)	ENSP00000217939.5:p.Pro516Ser
CAS-D	DM3	X;3242180;G;A	0.99	missense variant	MXRA5	.	probably damaging (1)	deleterious (0.01)	ENSP00000217939.5:p.Pro516Ser
CAS-D	DM4	X;3242180;G;A	0.95	missense variant	MXRA5	.	probably damaging (1)	deleterious (0.01)	ENSP00000217939.5:p.Pro516Ser
CAS-D	DM5	X;3242180;G;A	0.88	missense variant	MXRA5	.	probably damaging (1)	deleterious (0.01)	ENSP00000217939.5:p.Pro516Ser

CAS-D	DM6	X;3242180;G;A	1.00	missense variant	MXRA5	.	probably damaging (1)	deleterious (0.01)	ENSP00000217939.5:p.Pro516Ser
CAS-D	P1	X;3242180;G;A	0.76	missense variant	MXRA5	.	probably damaging (1)	deleterious (0.01)	ENSP00000217939.5:p.Pro516Ser
CAS-D	P2	X;3242180;G;A	0.96	missense variant	MXRA5	.	probably damaging (1)	deleterious (0.01)	ENSP00000217939.5:p.Pro516Ser
CAS-D	P3	X;3242180;G;A	0.74	missense variant	MXRA5	.	probably damaging (1)	deleterious (0.01)	ENSP00000217939.5:p.Pro516Ser
CAS-D	RM	X;3242180;G;A	1.00	missense variant	MXRA5	.	probably damaging (1)	deleterious (0.01)	ENSP00000217939.5:p.Pro516Ser
CAS-D	DM1	1;204197277;C;T	1.00	missense variant	PLEKHA6	.	probably damaging (0.998)	deleterious (0.01)	ENSP00000272203.2:p.Glu989Lys
CAS-D	DM2	1;204197277;C;T	0.66	missense variant	PLEKHA6	.	probably damaging (0.998)	deleterious (0.01)	ENSP00000272203.2:p.Glu989Lys
CAS-D	DM3	1;204197277;C;T	0.91	missense variant	PLEKHA6	.	probably damaging (0.998)	deleterious (0.01)	ENSP00000272203.2:p.Glu989Lys
CAS-D	DM4	1;204197277;C;T	0.50	missense variant	PLEKHA6	.	probably damaging (0.998)	deleterious (0.01)	ENSP00000272203.2:p.Glu989Lys
CAS-D	DM5	1;204197277;C;T	1.00	missense variant	PLEKHA6	.	probably damaging (0.998)	deleterious (0.01)	ENSP00000272203.2:p.Glu989Lys
CAS-D	DM6	1;204197277;C;T	0.95	missense variant	PLEKHA6	.	probably damaging (0.998)	deleterious (0.01)	ENSP00000272203.2:p.Glu989Lys
CAS-D	P1	1;204197277;C;T	0.35	missense variant	PLEKHA6	.	probably damaging (0.998)	deleterious (0.01)	ENSP00000272203.2:p.Glu989Lys
CAS-D	P2	1;204197277;C;T	0.50	missense variant	PLEKHA6	.	probably damaging (0.998)	deleterious (0.01)	ENSP00000272203.2:p.Glu989Lys
CAS-D	P3	1;204197277;C;T	0.38	missense variant	PLEKHA6	.	probably damaging (0.998)	deleterious (0.01)	ENSP00000272203.2:p.Glu989Lys
CAS-D	RM	1;204197277;C;T	0.81	missense variant	PLEKHA6	.	probably damaging (0.998)	deleterious (0.01)	ENSP00000272203.2:p.Glu989Lys
CAS-D	DM1	8;18729361;G;A	0.74	missense variant	PSD3	.	benign (0.014)	tolerated (0.09)	ENSP00000401704.2:p.Ser338Phe
CAS-D	DM2	8;18729361;G;A	1.00	missense variant	PSD3	.	benign (0.014)	tolerated (0.09)	ENSP00000401704.2:p.Ser338Phe
CAS-D	DM3	8;18729361;G;A	1.00	missense variant	PSD3	.	benign (0.014)	tolerated (0.09)	ENSP00000401704.2:p.Ser338Phe
CAS-D	DM4	8;18729361;G;A	0.98	missense variant	PSD3	.	benign (0.014)	tolerated (0.09)	ENSP00000401704.2:p.Ser338Phe
CAS-D	DM5	8;18729361;G;A	0.96	missense variant	PSD3	.	benign (0.014)	tolerated (0.09)	ENSP00000401704.2:p.Ser338Phe
CAS-D	DM6	8;18729361;G;A	1.00	missense variant	PSD3	.	benign (0.014)	tolerated (0.09)	ENSP00000401704.2:p.Ser338Phe
CAS-D	P1	8;18729361;G;A	0.50	missense variant	PSD3	.	benign (0.014)	tolerated (0.09)	ENSP00000401704.2:p.Ser338Phe
CAS-D	P2	8;18729361;G;A	1.00	missense variant	PSD3	.	benign (0.014)	tolerated (0.09)	ENSP00000401704.2:p.Ser338Phe
CAS-D	P3	8;18729361;G;A	0.47	missense variant	PSD3	.	benign (0.014)	tolerated (0.09)	ENSP00000401704.2:p.Ser338Phe
CAS-D	RM	8;18729361;G;A	1.00	missense variant	PSD3	.	benign (0.014)	tolerated (0.09)	ENSP00000401704.2:p.Ser338Phe
CAS-D	DM1	16;82034421;C;T	0.44	missense variant	SDR42E1	.	probably damaging (1)	deleterious (0)	ENSP00000332407.4:p.Gly15Arg
CAS-D	DM2	16;82034421;C;T	0.50	missense variant	SDR42E1	.	probably damaging (1)	deleterious (0)	ENSP00000332407.4:p.Gly15Arg
CAS-D	DM3	16;82034421;C;T	1.00	missense variant	SDR42E1	.	probably damaging (1)	deleterious (0)	ENSP00000332407.4:p.Gly15Arg

CAS-D	DM4	16;82034421;C;T	0.41	missense variant	SDR42E1	.	probably damaging (1)	deleterious (0)	ENSP00000332407.4:p.Gly15Arg
CAS-D	DM5	16;82034421;C;T	0.51	missense variant	SDR42E1	.	probably damaging (1)	deleterious (0)	ENSP00000332407.4:p.Gly15Arg
CAS-D	DM6	16;82034421;C;T	0.67	missense variant	SDR42E1	.	probably damaging (1)	deleterious (0)	ENSP00000332407.4:p.Gly15Arg
CAS-D	P1	16;82034421;C;T	0.20	missense variant	SDR42E1	.	probably damaging (1)	deleterious (0)	ENSP00000332407.4:p.Gly15Arg
CAS-D	P2	16;82034421;C;T	0.42	missense variant	SDR42E1	.	probably damaging (1)	deleterious (0)	ENSP00000332407.4:p.Gly15Arg
CAS-D	P3	16;82034421;C;T	0.22	missense variant	SDR42E1	.	probably damaging (1)	deleterious (0)	ENSP00000332407.4:p.Gly15Arg
CAS-D	RM	16;82034421;C;T	0.39	missense variant	SDR42E1	.	probably damaging (1)	deleterious (0)	ENSP00000332407.4:p.Gly15Arg
CAS-D	DM1	5;121759212;C;G	0.91	missense variant	SNCAIP	.	benign (0.012)	tolerated (0.07)	ENSP00000261367.7:p.Asn307Lys
CAS-D	DM1	5;121759229;C;T	0.90	missense variant	SNCAIP	.	benign (0.018)	deleterious (0)	ENSP00000261367.7:p.Pro313Leu
CAS-D	DM2	5;121759229;C;T	1.00	missense variant	SNCAIP	.	benign (0.018)	deleterious (0)	ENSP00000261367.7:p.Pro313Leu
CAS-D	DM2	5;121759212;C;G	1.00	missense variant	SNCAIP	.	benign (0.012)	tolerated (0.07)	ENSP00000261367.7:p.Asn307Lys
CAS-D	DM3	5;121759212;C;G	1.00	missense variant	SNCAIP	.	benign (0.012)	tolerated (0.07)	ENSP00000261367.7:p.Asn307Lys
CAS-D	DM3	5;121759229;C;T	1.00	missense variant	SNCAIP	.	benign (0.018)	deleterious (0)	ENSP00000261367.7:p.Pro313Leu
CAS-D	DM4	5;121759229;C;T	1.00	missense variant	SNCAIP	.	benign (0.018)	deleterious (0)	ENSP00000261367.7:p.Pro313Leu
CAS-D	DM4	5;121759212;C;G	1.00	missense variant	SNCAIP	.	benign (0.012)	tolerated (0.07)	ENSP00000261367.7:p.Asn307Lys
CAS-D	DM5	5;121759229;C;T	0.97	missense variant	SNCAIP	.	benign (0.018)	deleterious (0)	ENSP00000261367.7:p.Pro313Leu
CAS-D	DM5	5;121759212;C;G	0.97	missense variant	SNCAIP	.	benign (0.012)	tolerated (0.07)	ENSP00000261367.7:p.Asn307Lys
CAS-D	DM6	5;121759229;C;T	0.90	missense variant	SNCAIP	.	benign (0.018)	deleterious (0)	ENSP00000261367.7:p.Pro313Leu
CAS-D	DM6	5;121759212;C;G	0.89	missense variant	SNCAIP	.	benign (0.012)	tolerated (0.07)	ENSP00000261367.7:p.Asn307Lys
CAS-D	P1	5;121759229;C;T	0.36	missense variant	SNCAIP	.	benign (0.018)	deleterious (0)	ENSP00000261367.7:p.Pro313Leu
CAS-D	P1	5;121759212;C;G	0.30	missense variant	SNCAIP	.	benign (0.012)	tolerated (0.07)	ENSP00000261367.7:p.Asn307Lys
CAS-D	P2	5;121759212;C;G	0.50	missense variant	SNCAIP	.	benign (0.012)	tolerated (0.07)	ENSP00000261367.7:p.Asn307Lys
CAS-D	P2	5;121759229;C;T	0.49	missense variant	SNCAIP	.	benign (0.018)	deleterious (0)	ENSP00000261367.7:p.Pro313Leu
CAS-D	P3	5;121759229;C;T	0.35	missense variant	SNCAIP	.	benign (0.018)	deleterious (0)	ENSP00000261367.7:p.Pro313Leu
CAS-D	P3	5;121759212;C;G	0.28	missense variant	SNCAIP	.	benign (0.012)	tolerated (0.07)	ENSP00000261367.7:p.Asn307Lys
CAS-D	RM	5;121759229;C;T	0.93	missense variant	SNCAIP	.	benign (0.018)	deleterious (0)	ENSP00000261367.7:p.Pro313Leu
CAS-D	RM	5;121759212;C;G	0.73	missense variant	SNCAIP	.	benign (0.012)	tolerated (0.07)	ENSP00000261367.7:p.Asn307Lys
CAS-D	DM1	3;113176065;A;T	0.91	missense variant	SPICE1	.	benign (0.034)	tolerated (1)	ENSP00000295872.4:p.Phe525Leu

CAS-D	DM2	3;113176065;A;T	1.00	missense variant	SPICE1	.	benign (0.034)	tolerated (1)	ENSP00000295872.4:p.Phe525Leu
CAS-D	DM3	3;113176065;A;T	1.00	missense variant	SPICE1	.	benign (0.034)	tolerated (1)	ENSP00000295872.4:p.Phe525Leu
CAS-D	DM4	3;113176065;A;T	1.00	missense variant	SPICE1	.	benign (0.034)	tolerated (1)	ENSP00000295872.4:p.Phe525Leu
CAS-D	DM5	3;113176065;A;T	1.00	missense variant	SPICE1	.	benign (0.034)	tolerated (1)	ENSP00000295872.4:p.Phe525Leu
CAS-D	DM6	3;113176065;A;T	1.00	missense variant	SPICE1	.	benign (0.034)	tolerated (1)	ENSP00000295872.4:p.Phe525Leu
CAS-D	P1	3;113176065;A;T	0.35	missense variant	SPICE1	.	benign (0.034)	tolerated (1)	ENSP00000295872.4:p.Phe525Leu
CAS-D	P2	3;113176065;A;T	0.66	missense variant	SPICE1	.	benign (0.034)	tolerated (1)	ENSP00000295872.4:p.Phe525Leu
CAS-D	P3	3;113176065;A;T	0.33	missense variant	SPICE1	.	benign (0.034)	tolerated (1)	ENSP00000295872.4:p.Phe525Leu
CAS-D	RM	3;113176065;A;T	1.00	missense variant	SPICE1	.	benign (0.034)	tolerated (1)	ENSP00000295872.4:p.Phe525Leu
CAS-C	DM6	17;27893804;C;T	0.21	missense variant	ABHD15	.	benign (0.007)	tolerated (0.47)	ENSP00000302657.3:p.Asp61Asn
CAS-C	DM1	16;77401383;G;A	0.50	missense variant	ADAMTS18	.	benign (0.01)	tolerated (0.52)	ENSP00000392540.2:p.His245Tyr
CAS-C	DM2	16;77401383;G;A	0.50	missense variant	ADAMTS18	.	benign (0.01)	tolerated (0.52)	ENSP00000392540.2:p.His245Tyr
CAS-C	DM3	16;77401383;G;A	0.47	missense variant	ADAMTS18	.	benign (0.01)	tolerated (0.52)	ENSP00000392540.2:p.His245Tyr
CAS-C	DM4	16;77401383;G;A	0.42	missense variant	ADAMTS18	.	benign (0.01)	tolerated (0.52)	ENSP00000392540.2:p.His245Tyr
CAS-C	DM5	16;77401383;G;A	0.38	missense variant	ADAMTS18	.	benign (0.01)	tolerated (0.52)	ENSP00000392540.2:p.His245Tyr
CAS-C	DM6	16;77401383;G;A	0.67	missense variant	ADAMTS18	.	benign (0.01)	tolerated (0.52)	ENSP00000392540.2:p.His245Tyr
CAS-C	P	16;77401383;G;A	0.32	missense variant	ADAMTS18	.	benign (0.01)	tolerated (0.52)	ENSP00000392540.2:p.His245Tyr
CAS-C	DM1	11;10825714;C;A	0.50	stop gained	EIF4G2	.	.	.	ENSP00000435523.1:p.Glu174Ter
CAS-C	DM2	11;10825714;C;A	0.50	stop gained	EIF4G2	.	.	.	ENSP00000435523.1:p.Glu174Ter
CAS-C	DM3	11;10825714;C;A	0.50	stop gained	EIF4G2	.	.	.	ENSP00000435523.1:p.Glu174Ter
CAS-C	DM4	11;10825714;C;A	0.50	stop gained	EIF4G2	.	.	.	ENSP00000435523.1:p.Glu174Ter
CAS-C	DM5	11;10825714;C;A	0.40	stop gained	EIF4G2	.	.	.	ENSP00000435523.1:p.Glu174Ter
CAS-C	DM6	11;10825714;C;A	0.67	stop gained	EIF4G2	.	.	.	ENSP00000435523.1:p.Glu174Ter
CAS-C	P	11;10825714;C;A	0.50	stop gained	EIF4G2	.	.	.	ENSP00000435523.1:p.Glu174Ter
CAS-C	DM1	15;72642972;G;T	0.44	missense variant	HEXA	.	benign (0.033)	tolerated (0.22)	ENSP00000457125.1:p.Thr231Asn
CAS-C	DM2	15;72642972;G;T	0.50	missense variant	HEXA	.	benign (0.033)	tolerated (0.22)	ENSP00000457125.1:p.Thr231Asn
CAS-C	DM3	15;72642972;G;T	0.41	missense variant	HEXA	.	benign (0.033)	tolerated (0.22)	ENSP00000457125.1:p.Thr231Asn
CAS-C	DM4	15;72642972;G;T	0.50	missense variant	HEXA	.	benign (0.033)	tolerated (0.22)	ENSP00000457125.1:p.Thr231Asn

CAS-C	DM5	15;72642972;G;T	0.50	missense variant	HEXA	.	benign (0.033)	tolerated (0.22)	ENSP00000457125.1:p.Thr231Asn
CAS-C	DM6	15;72642972;G;T	0.67	missense variant	HEXA	.	benign (0.033)	tolerated (0.22)	ENSP00000457125.1:p.Thr231Asn
CAS-C	P	15;72642972;G;T	0.50	missense variant	HEXA	.	benign (0.033)	tolerated (0.22)	ENSP00000457125.1:p.Thr231Asn
CAS-C	DM1	14;77945023;G;A	0.39	missense variant	ISM2	.	probably damaging (0.96)	tolerated (0.05)	ENSP00000341490.4:p.Pro337Ser
CAS-C	DM2	14;77945023;G;A	0.48	missense variant	ISM2	.	probably damaging (0.96)	tolerated (0.05)	ENSP00000341490.4:p.Pro337Ser
CAS-C	DM3	14;77945023;G;A	0.41	missense variant	ISM2	.	probably damaging (0.96)	tolerated (0.05)	ENSP00000341490.4:p.Pro337Ser
CAS-C	DM4	14;77945023;G;A	0.36	missense variant	ISM2	.	probably damaging (0.96)	tolerated (0.05)	ENSP00000341490.4:p.Pro337Ser
CAS-C	DM5	14;77945023;G;A	0.50	missense variant	ISM2	.	probably damaging (0.96)	tolerated (0.05)	ENSP00000341490.4:p.Pro337Ser
CAS-C	DM6	14;77945023;G;A	0.67	missense variant	ISM2	.	probably damaging (0.96)	tolerated (0.05)	ENSP00000341490.4:p.Pro337Ser
CAS-C	P	14;77945023;G;A	0.23	missense variant	ISM2	.	probably damaging (0.96)	tolerated (0.05)	ENSP00000341490.4:p.Pro337Ser
CAS-C	DM1	11;128781776;C;A	0.50	missense variant	KCNJ5	.	probably damaging (1)	deleterious (0)	ENSP00000433295.1:p.Ser203Tyr
CAS-C	DM2	11;128781776;C;A	0.46	missense variant	KCNJ5	.	probably damaging (1)	deleterious (0)	ENSP00000433295.1:p.Ser203Tyr
CAS-C	DM3	11;128781776;C;A	0.39	missense variant	KCNJ5	.	probably damaging (1)	deleterious (0)	ENSP00000433295.1:p.Ser203Tyr
CAS-C	DM4	11;128781776;C;A	0.50	missense variant	KCNJ5	.	probably damaging (1)	deleterious (0)	ENSP00000433295.1:p.Ser203Tyr
CAS-C	DM5	11;128781776;C;A	0.41	missense variant	KCNJ5	.	probably damaging (1)	deleterious (0)	ENSP00000433295.1:p.Ser203Tyr
CAS-C	DM6	11;128781776;C;A	0.67	missense variant	KCNJ5	.	probably damaging (1)	deleterious (0)	ENSP00000433295.1:p.Ser203Tyr
CAS-C	P	11;128781776;C;A	0.37	missense variant	KCNJ5	.	probably damaging (1)	deleterious (0)	ENSP00000433295.1:p.Ser203Tyr
CAS-C	DM1	11;101832797;A;G	0.50	missense variant	KIAA1377	.	benign (0.046)	tolerated (0.28)	ENSP00000263468.8:p.Asn344Ser
CAS-C	DM2	11;101832797;A;G	0.42	missense variant	KIAA1377	.	benign (0.046)	tolerated (0.28)	ENSP00000263468.8:p.Asn344Ser
CAS-C	DM3	11;101832797;A;G	0.50	missense variant	KIAA1377	.	benign (0.046)	tolerated (0.28)	ENSP00000263468.8:p.Asn344Ser
CAS-C	DM4	11;101832797;A;G	0.50	missense variant	KIAA1377	.	benign (0.046)	tolerated (0.28)	ENSP00000263468.8:p.Asn344Ser
CAS-C	DM5	11;101832797;A;G	0.50	missense variant	KIAA1377	.	benign (0.046)	tolerated (0.28)	ENSP00000263468.8:p.Asn344Ser
CAS-C	DM6	11;101832797;A;G	0.67	missense variant	KIAA1377	.	benign (0.046)	tolerated (0.28)	ENSP00000263468.8:p.Asn344Ser
CAS-C	DM1	14;50769638;C;T	0.49	missense variant	L2HGDH	.	probably damaging (0.998)	deleterious (0)	ENSP00000450494.1:p.Glu80Lys
CAS-C	DM2	14;50769638;C;T	0.50	missense variant	L2HGDH	.	probably damaging (0.998)	deleterious (0)	ENSP00000450494.1:p.Glu80Lys
CAS-C	DM3	14;50769638;C;T	0.42	missense variant	L2HGDH	.	probably damaging (0.998)	deleterious (0)	ENSP00000450494.1:p.Glu80Lys
CAS-C	DM4	14;50769638;C;T	0.40	missense variant	L2HGDH	.	probably damaging (0.998)	deleterious (0)	ENSP00000450494.1:p.Glu80Lys
CAS-C	DM5	14;50769638;C;T	0.29	missense variant	L2HGDH	.	probably damaging (0.998)	deleterious (0)	ENSP00000450494.1:p.Glu80Lys

CAS-C	DM6	14;50769638;C;T	0.65	missense variant	L2HGDH	.	probably damaging (0.998)	deleterious (0)	ENSP00000450494.1:p.Glu80Lys
CAS-C	P	14;50769638;C;T	0.50	missense variant	L2HGDH	.	probably damaging (0.998)	deleterious (0)	ENSP00000450494.1:p.Glu80Lys
CAS-C	DM6	1;52863511;C;T	0.20	missense variant	ORC1	.	benign (0.004)	tolerated (0.55)	ENSP00000360623.3:p.Arg83His
CAS-C	DM5	1;52863511;C;T	0.01	missense variant	ORC1	.	benign (0.004)	tolerated (0.55)	ENSP00000360623.3:p.Arg83His
CAS-C	P	1;52863511;C;T	0.005	missense variant	ORC1	.	benign (0.004)	tolerated (0.55)	ENSP00000360623.3:p.Arg83His
CAS-C	DM1	11;62382289;A;C	0.41	missense variant	ROM1	.	benign (0.074)	tolerated (0.07)	ENSP00000278833.3:p.Lys345Thr
CAS-C	DM2	11;62382289;A;C	0.50	missense variant	ROM1	.	benign (0.074)	tolerated (0.07)	ENSP00000278833.3:p.Lys345Thr
CAS-C	DM3	11;62382289;A;C	0.50	missense variant	ROM1	.	benign (0.074)	tolerated (0.07)	ENSP00000278833.3:p.Lys345Thr
CAS-C	DM4	11;62382289;A;C	0.50	missense variant	ROM1	.	benign (0.074)	tolerated (0.07)	ENSP00000278833.3:p.Lys345Thr
CAS-C	DM5	11;62382289;A;C	0.39	missense variant	ROM1	.	benign (0.074)	tolerated (0.07)	ENSP00000278833.3:p.Lys345Thr
CAS-C	DM6	11;62382289;A;C	0.67	missense variant	ROM1	.	benign (0.074)	tolerated (0.07)	ENSP00000278833.3:p.Lys345Thr
CAS-C	P	11;62382289;A;C	0.50	missense variant	ROM1	.	benign (0.074)	tolerated (0.07)	ENSP00000278833.3:p.Lys345Thr
CAS-C	DM1	14;95029946;G;A	0.50	missense variant	SERPINA4	.	possibly damaging (0.454)	tolerated (0.34)	ENSP00000450838.1:p.Gly43Ser
CAS-C	DM2	14;95029946;G;A	0.44	missense variant	SERPINA4	.	possibly damaging (0.454)	tolerated (0.34)	ENSP00000450838.1:p.Gly43Ser
CAS-C	DM3	14;95029946;G;A	0.50	missense variant	SERPINA4	.	possibly damaging (0.454)	tolerated (0.34)	ENSP00000450838.1:p.Gly43Ser
CAS-C	DM4	14;95029946;G;A	0.39	missense variant	SERPINA4	.	possibly damaging (0.454)	tolerated (0.34)	ENSP00000450838.1:p.Gly43Ser
CAS-C	DM5	14;95029946;G;A	0.40	missense variant	SERPINA4	.	possibly damaging (0.454)	tolerated (0.34)	ENSP00000450838.1:p.Gly43Ser
CAS-C	DM6	14;95029946;G;A	0.64	missense variant	SERPINA4	.	possibly damaging (0.454)	tolerated (0.34)	ENSP00000450838.1:p.Gly43Ser
CAS-C	P	14;95029946;G;A	0.39	missense variant	SERPINA4	.	possibly damaging (0.454)	tolerated (0.34)	ENSP00000450838.1:p.Gly43Ser
CAS-C	DM1	14;94933501;C;T	0.34	missense variant	SERPINA9	.	probably damaging (0.999)	deleterious (0)	ENSP00000337133.5:p.Glu301Lys
CAS-C	DM2	14;94933501;C;T	0.25	missense variant	SERPINA9	.	probably damaging (0.999)	deleterious (0)	ENSP00000337133.5:p.Glu301Lys
CAS-C	DM3	14;94933501;C;T	0.50	missense variant	SERPINA9	.	probably damaging (0.999)	deleterious (0)	ENSP00000337133.5:p.Glu301Lys
CAS-C	DM4	14;94933501;C;T	0.50	missense variant	SERPINA9	.	probably damaging (0.999)	deleterious (0)	ENSP00000337133.5:p.Glu301Lys
CAS-C	DM5	14;94933501;C;T	0.47	missense variant	SERPINA9	.	probably damaging (0.999)	deleterious (0)	ENSP00000337133.5:p.Glu301Lys
CAS-C	DM6	14;94933501;C;T	0.67	missense variant	SERPINA9	.	probably damaging (0.999)	deleterious (0)	ENSP00000337133.5:p.Glu301Lys
CAS-C	P	14;94933501;C;T	0.50	missense variant	SERPINA9	.	probably damaging (0.999)	deleterious (0)	ENSP00000337133.5:p.Glu301Lys
CAS-C	DM1	11;65417650;A;T	0.43	stop gained	SIPA1	.	.	.	ENSP00000436269.1:p.Lys964Ter
CAS-C	DM2	11;65417650;A;T	0.48	stop gained	SIPA1	.	.	.	ENSP00000436269.1:p.Lys964Ter

CAS-C	DM3	11;65417650;A;T	0.50	stop gained	SIPA1	.	.	.	ENSP00000436269.1:p.Lys964Ter
CAS-C	DM4	11;65417650;A;T	0.50	stop gained	SIPA1	.	.	.	ENSP00000436269.1:p.Lys964Ter
CAS-C	DM5	11;65417650;A;T	0.50	stop gained	SIPA1	.	.	.	ENSP00000436269.1:p.Lys964Ter
CAS-C	DM6	11;65417650;A;T	0.67	stop gained	SIPA1	.	.	.	ENSP00000436269.1:p.Lys964Ter
CAS-C	P	11;65417650;A;T	0.47	stop gained	SIPA1	.	.	.	ENSP00000436269.1:p.Lys964Ter
CAS-C	DM1	11;62575106;G;C	0.50	missense variant	STX5	.	benign (0.003)	.	ENSP00000367129.4:p.Ser314Cys
CAS-C	DM2	11;62575106;G;C	0.43	missense variant	STX5	.	benign (0.003)	.	ENSP00000367129.4:p.Ser314Cys
CAS-C	DM3	11;62575106;G;C	0.28	missense variant	STX5	.	benign (0.003)	.	ENSP00000367129.4:p.Ser314Cys
CAS-C	DM4	11;62575106;G;C	0.38	missense variant	STX5	.	benign (0.003)	.	ENSP00000367129.4:p.Ser314Cys
CAS-C	DM5	11;62575106;G;C	0.33	missense variant	STX5	.	benign (0.003)	.	ENSP00000367129.4:p.Ser314Cys
CAS-C	DM6	11;62575106;G;C	0.61	missense variant	STX5	.	benign (0.003)	.	ENSP00000367129.4:p.Ser314Cys
CAS-C	P	11;62575106;G;C	0.38	missense variant	STX5	.	benign (0.003)	.	ENSP00000367129.4:p.Ser314Cys
CAS-C	DM6	11;101353764;T;C	0.57	missense variant	TRPC6	.	benign (0.039)	tolerated (0.43)	ENSP00000353687.4:p.Ser421Gly
CAS-C	DM1	6;33283340;C;T	0.34	missense variant	ZBTB22	.	probably damaging (0.999)	deleterious (0.01)	ENSP00000404403.1:p.Gly452Arg
CAS-C	DM2	6;33283340;C;T	0.33	missense variant	ZBTB22	.	probably damaging (0.999)	deleterious (0.01)	ENSP00000404403.1:p.Gly452Arg
CAS-C	DM3	6;33283340;C;T	0.38	missense variant	ZBTB22	.	probably damaging (0.999)	deleterious (0.01)	ENSP00000404403.1:p.Gly452Arg
CAS-C	DM4	6;33283340;C;T	0.27	missense variant	ZBTB22	.	probably damaging (0.999)	deleterious (0.01)	ENSP00000404403.1:p.Gly452Arg
CAS-C	DM5	6;33283340;C;T	0.25	missense variant	ZBTB22	.	probably damaging (0.999)	deleterious (0.01)	ENSP00000404403.1:p.Gly452Arg
CAS-C	DM6	6;33283340;C;T	0.50	missense variant	ZBTB22	.	probably damaging (0.999)	deleterious (0.01)	ENSP00000404403.1:p.Gly452Arg
CAS-C	P	6;33283340;C;T	0.27	missense variant	ZBTB22	.	probably damaging (0.999)	deleterious (0.01)	ENSP00000404403.1:p.Gly452Arg
ETH-E	DM1	7;48318608;C;A	0.38	missense variant	ABCA13	.	benign (0.234)	deleterious (0)	ENSP00000411096.1:p.Pro2606His
ETH-E	DM2	7;48318608;C;A	0.25	missense variant	ABCA13	.	benign (0.234)	deleterious (0)	ENSP00000411096.1:p.Pro2606His
ETH-E	DM3	7;48318608;C;A	0.24	missense variant	ABCA13	.	benign (0.234)	deleterious (0)	ENSP00000411096.1:p.Pro2606His
ETH-E	DM1	10;28151532;C;A	0.79	missense variant	ARMC4	.	possibly damaging (0.58)	deleterious (0.05)	ENSP00000443208.1:p.Arg569Leu
ETH-E	DM2	10;28151532;C;A	0.78	missense variant	ARMC4	.	possibly damaging (0.58)	deleterious (0.05)	ENSP00000443208.1:p.Arg569Leu
ETH-E	DM3	10;28151532;C;A	1.00	missense variant	ARMC4	.	possibly damaging (0.58)	deleterious (0.05)	ENSP00000443208.1:p.Arg569Leu
ETH-E	DM1	11;76158048;A;T	1.00	missense variant	C11orf30	.	possibly damaging (0.758)	deleterious (0.01)	ENSP00000432327.1:p.Lys22Asn
ETH-E	DM2	11;76158048;A;T	0.99	missense variant	C11orf30	.	possibly damaging (0.758)	deleterious (0.01)	ENSP00000432327.1:p.Lys22Asn

ETH-E	DM3	11;76158048;A;T	1.00	missense variant	C11orf30	.	possibly damaging (0.758)	deleterious (0.01)	ENSP00000432327.1:p.Lys22Asn
ETH-E	DM1	3;40433630;C;T	1.00	missense variant	ENTPD3	.	probably damaging (0.995)	tolerated (0.11)	ENSP00000301825.3:p.Pro53Ser
ETH-E	DM2	3;40433630;C;T	0.91	missense variant	ENTPD3	.	probably damaging (0.995)	tolerated (0.11)	ENSP00000301825.3:p.Pro53Ser
ETH-E	DM3	3;40433630;C;T	0.88	missense variant	ENTPD3	.	probably damaging (0.995)	tolerated (0.11)	ENSP00000301825.3:p.Pro53Ser
ETH-E	RM	3;40433630;C;T	0.66	missense variant	ENTPD3	.	probably damaging (0.995)	tolerated (0.11)	ENSP00000301825.3:p.Pro53Ser
ETH-E	DM1	2;14789439;G;A	0.67	splice acceptor variant	FAM84A	.	.	.	.
ETH-E	DM2	2;14789439;G;A	0.64	splice acceptor variant	FAM84A	.	.	.	.
ETH-E	DM3	2;14789439;G;A	0.57	splice acceptor variant	FAM84A	.	.	.	.
ETH-E	P	2;14789439;G;A	0.40	splice acceptor variant	FAM84A	.	.	.	.
ETH-E	RM	2;14789439;G;A	0.45	splice acceptor variant	FAM84A	.	.	.	.
ETH-E	DM1	16;71220694;C;A	0.20	missense variant	HYDIN	.	possibly damaging (0.887)	deleterious (0)	ENSP00000314736.5:p.Lys35Asn
ETH-E	DM2	16;71220694;C;A	0.50	missense variant	HYDIN	.	possibly damaging (0.887)	deleterious (0)	ENSP00000314736.5:p.Lys35Asn
ETH-E	DM3	16;71220694;C;A	0.28	missense variant	HYDIN	.	possibly damaging (0.887)	deleterious (0)	ENSP00000314736.5:p.Lys35Asn
ETH-E	DM1	22;21348438;G;A	0.96	missense variant	LZTR1	.	benign (0.308)	tolerated (0.13)	ENSP00000374006.3:p.Val480Met
ETH-E	DM2	22;21348438;G;A	1.00	missense variant	LZTR1	.	benign (0.308)	tolerated (0.13)	ENSP00000374006.3:p.Val480Met
ETH-E	DM3	22;21348438;G;A	1.00	missense variant	LZTR1	.	benign (0.308)	tolerated (0.13)	ENSP00000374006.3:p.Val480Met
ETH-E	P	22;21348438;G;A	0.16	missense variant	LZTR1	.	benign (0.308)	tolerated (0.13)	ENSP00000374006.3:p.Val480Met
ETH-E	RM	22;21348438;G;A	0.39	missense variant	LZTR1	.	benign (0.308)	tolerated (0.13)	ENSP00000374006.3:p.Val480Met
ETH-E	DM1	5;126783275;A;T	0.96	missense variant	MEGF10	.	possibly damaging (0.832)	tolerated (0.06)	ENSP00000423354.2:p.Asn919Tyr
ETH-E	DM2	5;126783275;A;T	1.00	missense variant	MEGF10	.	possibly damaging (0.832)	tolerated (0.06)	ENSP00000423354.2:p.Asn919Tyr
ETH-E	DM3	5;126783275;A;T	1.00	missense variant	MEGF10	.	possibly damaging (0.832)	tolerated (0.06)	ENSP00000423354.2:p.Asn919Tyr
ETH-E	P	5;126783275;A;T	0.40	missense variant	MEGF10	.	possibly damaging (0.832)	tolerated (0.06)	ENSP00000423354.2:p.Asn919Tyr
ETH-E	RM	5;126783275;A;T	0.85	missense variant	MEGF10	.	possibly damaging (0.832)	tolerated (0.06)	ENSP00000423354.2:p.Asn919Tyr
ETH-E	DM1	16;50745225;G;A	0.28	missense variant	NOD2	.	possibly damaging (0.901)	tolerated (0.08)	ENSP00000300589.2:p.Arg468His
ETH-E	DM2	16;50745225;G;A	0.48	missense variant	NOD2	.	possibly damaging (0.901)	tolerated (0.08)	ENSP00000300589.2:p.Arg468His
ETH-E	DM3	16;50745225;G;A	0.39	missense variant	NOD2	.	possibly damaging (0.901)	tolerated (0.08)	ENSP00000300589.2:p.Arg468His
ETH-E	DM1	8;18729956;C;T	1.00	missense variant	PSD3	.	possibly damaging (0.635)	deleterious (0)	ENSP00000401704.2:p.Ala140Thr
ETH-E	DM2	8;18729956;C;T	1.00	missense variant	PSD3	.	possibly damaging (0.635)	deleterious (0)	ENSP00000401704.2:p.Ala140Thr

ETH-E	DM3	8;18729956;C;T	1.00	missense variant	PSD3	.	possibly damaging (0.635)	deleterious (0)	ENSP00000401704.2:p.Ala140Thr
ETH-E	P	8;18729956;C;T	0.44	missense variant	PSD3	.	possibly damaging (0.635)	deleterious (0)	ENSP00000401704.2:p.Ala140Thr
ETH-E	RM	8;18729956;C;T	0.40	missense variant	PSD3	.	possibly damaging (0.635)	deleterious (0)	ENSP00000401704.2:p.Ala140Thr
ETH-E	DM1	8;14022112;C;A	1.00	missense variant	SGCZ	.	possibly damaging (0.538)	deleterious (0)	ENSP00000405224.2:p.Gly128Val
ETH-E	DM2	8;14022112;C;A	0.92	missense variant	SGCZ	.	possibly damaging (0.538)	deleterious (0)	ENSP00000405224.2:p.Gly128Val
ETH-E	DM3	8;14022112;C;A	0.96	missense variant	SGCZ	.	possibly damaging (0.538)	deleterious (0)	ENSP00000405224.2:p.Gly128Val
ETH-E	P	8;14022112;C;A	0.02	missense variant	SGCZ	.	possibly damaging (0.538)	deleterious (0)	ENSP00000405224.2:p.Gly128Val
ETH-E	RM	8;14022112;C;A	0.43	missense variant	SGCZ	.	possibly damaging (0.538)	deleterious (0)	ENSP00000405224.2:p.Gly128Val
ETH-E	DM1	11;65414015;C;T	1.00	missense variant	SIPA1	.	probably damaging (1)	deleterious (0)	ENSP00000436269.1:p.Arg504Trp
ETH-E	DM2	11;65414015;C;T	1.00	missense variant	SIPA1	.	probably damaging (1)	deleterious (0)	ENSP00000436269.1:p.Arg504Trp
ETH-E	DM3	11;65414015;C;T	0.86	missense variant	SIPA1	.	probably damaging (1)	deleterious (0)	ENSP00000436269.1:p.Arg504Trp
ETH-E	P	11;65414015;C;T	0.36	missense variant	SIPA1	.	probably damaging (1)	deleterious (0)	ENSP00000436269.1:p.Arg504Trp
ETH-E	RM	11;65414015;C;T	0.50	missense variant	SIPA1	.	probably damaging (1)	deleterious (0)	ENSP00000436269.1:p.Arg504Trp
ETH-E	DM1	6;84270606;G;T	0.66	missense variant	SNAP91	.	possibly damaging (0.824)	tolerated (0.33)	ENSP00000428215.1:p.Gln835Lys
ETH-E	DM2	6;84270606;G;T	0.36	missense variant	SNAP91	.	possibly damaging (0.824)	tolerated (0.33)	ENSP00000428215.1:p.Gln835Lys
ETH-E	DM3	6;84270606;G;T	0.37	missense variant	SNAP91	.	possibly damaging (0.824)	tolerated (0.33)	ENSP00000428215.1:p.Gln835Lys
ETH-E	DM1	2;54858731;G;T	0.41	missense variant	SPTBN1	.	benign (0.34)	tolerated (0.29)	ENSP00000349259.4:p.Ala1183Ser
ETH-E	DM2	2;54858731;G;T	0.37	missense variant	SPTBN1	.	benign (0.34)	tolerated (0.29)	ENSP00000349259.4:p.Ala1183Ser
ETH-E	DM3	2;54858731;G;T	0.38	missense variant	SPTBN1	.	benign (0.34)	tolerated (0.29)	ENSP00000349259.4:p.Ala1183Ser
ETH-E	DM1	12;10777303;C;G	0.67	missense variant	STYK1	.	probably damaging (1)	deleterious (0)	ENSP00000075503.3:p.Lys291Asn
ETH-E	DM2	12;10777303;C;G	0.67	missense variant	STYK1	.	probably damaging (1)	deleterious (0)	ENSP00000075503.3:p.Lys291Asn
ETH-E	DM3	12;10777303;C;G	0.65	missense variant	STYK1	.	probably damaging (1)	deleterious (0)	ENSP00000075503.3:p.Lys291Asn
ETH-E	P	12;10777303;C;G	0.02	missense variant	STYK1	.	probably damaging (1)	deleterious (0)	ENSP00000075503.3:p.Lys291Asn
ETH-E	RM	12;10777303;C;G	0.42	missense variant	STYK1	.	probably damaging (1)	deleterious (0)	ENSP00000075503.3:p.Lys291Asn
ETH-E	DM1	8;38678116;T;G	0.50	missense variant	TACC1	.	probably damaging (0.95)	tolerated (0.38)	ENSP00000428253.1:p.Leu227Val
ETH-E	DM2	8;38678116;T;G	0.50	missense variant	TACC1	.	probably damaging (0.95)	tolerated (0.38)	ENSP00000428253.1:p.Leu227Val
ETH-E	DM3	8;38678116;T;G	0.50	missense variant	TACC1	.	probably damaging (0.95)	tolerated (0.38)	ENSP00000428253.1:p.Leu227Val
ETH-E	DM1	16;49672325;C;A	0.50	missense variant	ZNF423	.	possibly damaging (0.843)	deleterious (0.01)	ENSP00000457928.1:p.Lys186Asn

ETH-E	DM2	16;49672325;C;A	0.49	missense variant	ZNF423	.	possibly damaging (0.843)	deleterious (0.01)	ENSP00000457928.1:p.Lys186Asn
ETH-E	DM3	16;49672325;C;A	0.58	missense variant	ZNF423	.	possibly damaging (0.843)	deleterious (0.01)	ENSP00000457928.1:p.Lys186Asn
ETH-E	DM1	2;185802564;G;A	0.47	missense variant	ZNF804A	.	probably damaging (0.953)	deleterious (0.01)	ENSP00000303252.6:p.Arg814Gln
ETH-E	DM2	2;185802564;G;A	0.65	missense variant	ZNF804A	.	probably damaging (0.953)	deleterious (0.01)	ENSP00000303252.6:p.Arg814Gln
ETH-E	DM3	2;185802564;G;A	0.57	missense variant	ZNF804A	.	probably damaging (0.953)	deleterious (0.01)	ENSP00000303252.6:p.Arg814Gln
ETH-E	P	2;185802564;G;A	0.31	missense variant	ZNF804A	.	probably damaging (0.953)	deleterious (0.01)	ENSP00000303252.6:p.Arg814Gln
ETH-E	RM	2;185802564;G;A	0.34	missense variant	ZNF804A	.	probably damaging (0.953)	deleterious (0.01)	ENSP00000303252.6:p.Arg814Gln

Supplementary Table 12. GO Slim terms associated with mutations gained or increased in allelic dose with increased ploidy in patients CAS-D, CAS-G, CAS-C and ETH-E.

GO Slim Annotation	Number of Genes	Genes
intracellular	28	ACACA,C11orf30,DNHD1,EIF4G2,EP400,FRY,GIMAP5,HGD,ISM2,JAKMIP1,KDM3B,LIN28B,MICU3,NOD2,ORC1,PSD3,RELN, SERPINA9,SIPA1,SNAP91,SNCAIP,SPICE1,SPTBN1,STX5,TACC1,ZBTB22,ZNF260,ZNF423
organelle	25	ACACA,C11orf30,DNHD1,EP400,FRY,GIMAP5,HYDIN,ISM2,JAKMIP1,KDM3B,LIN28B,MICU3,NOD2,ORC1,PSD3,ROM1, SIPA1,SNCAIP,SPICE1,SPTBN1,STX5,TACC1,ZBTB22,ZNF260,ZNF423
ion binding	24	ABCA13,ACACA,ADAMTS18,DCST1,ENTPD3,EP400,FER1L6,GIMAP5,HGD,KAL1,KDM3B,LIN28B,MICU3,NOD2,ORC1,RELN,SNAP 91, SPTBN1,STYK1,TRPC6,ZBTB22,ZNF260,ZNF423,ZNF804A
cytoplasm	20	ACACA,EIF4G2,FRY,GIMAP5,HGD,JAKMIP1,LIN28B,MICU3,NOD2,ORC1,RELN,SERPINA9,SIPA1,SNAP91,SNCAIP,SPICE1,SPTBN1 ,STX5, TACC1,ZNF260
plasma membrane	18	CASR,ENTPD3,IL7R,KAL1,KCNJ5,MEGF10,MGAM,NOD2,ORC1,PDCD1,PSD3,ROM1,SGCZ,SNAP91,SNCAIP,SPTBN1,STYK1,TRPC6
transport	16	ABCA13,ACACA,CASR,DNHD1,FER1L6,GIMAP5,JAKMIP1,KCNJ5,MEGF10,NOD2,RELN,SNAP91,SNCAIP,SPTBN1,STX5,TRPC6
cellular nitrogen compound metabolic process	15	ACACA,C11orf30,ENTPD3,GIMAP5,HGD,IL7R,KDM3B,LIN28B,NOD2,ORC1,RELN,SIPA1,ZBTB22,ZNF260,ZNF423
nucleus	15	C11orf30,EP400,ISM2,KDM3B,LIN28B,NOD2,ORC1,SIPA1,SNCAIP,SPTBN1,STX5,TACC1,ZBTB22,ZNF260,ZNF423
biosynthetic process	12	ACACA,C11orf30,GIMAP5,KDM3B,LIN28B,NOD2,ORC1,RELN,SDR42E1,ZBTB22,ZNF260,ZNF423
signal transduction	11	ADAMTS18,CASR,IL7R,LILRB5,NOD2,PDCD1,PSD3,RELN,SIPA1,SPTBN1,ZNF423
anatomical structure development	11	CASR,GIMAP5,IL7R,KAL1,MEGF10,PSD3,RELN,SPTBN1,TACC1,TRPC6,ZNF423
protein complex	11	DNHD1,EP400,FRY,JAKMIP1,KCNJ5,NOD2,ORC1,SGCZ,SNAP91,SPICE1,SPTBN1
cell differentiation	10	GIMAP5,IL7R,KAL1,MEGF10,PSD3,RELN,SPTBN1,TACC1,TRPC6,ZNF423
cellular component assembly	8	ACACA,DNHD1,NOD2,RELN,SNAP91,SNCAIP,SPICE1,SPTBN1
cytoskeleton	8	DNHD1,FRY,JAKMIP1,NOD2,PSD3,SPICE1,SPTBN1,TACC1
extracellular region	8	ABHD15,IL7R,ISM2,KAL1,MXRA5,RELN,SERPINA4,SERPINA9
DNA binding	6	EP400,LIN28B,ORC1,ZBTB22,ZNF260,ZNF423
catabolic process	6	ENTPD3,GIMAP5,HGD,LIN28B,MGAM,SIPA1
cytosol	6	ACACA,HGD,NOD2,ORC1,SIPA1,SPTBN1
locomotion	6	DNHD1,KAL1,NOD2,RELN,SPTBN1,TRPC6
response to stress	6	C11orf30,GIMAP5,LILRB5,NOD2,RELN,TRPC6
vesicle-mediated transport	6	DNHD1,MEGF10,NOD2,SNAP91,SPTBN1,STX5
cell adhesion	5	KAL1,MEGF10,RELN,ROM1,SIPA1
cell cycle	5	ORC1,SIPA1,SPICE1,SPTBN1,TACC1
cell morphogenesis	5	IL7R,KAL1,RELN,SPTBN1,TRPC6
cell proliferation	5	IL7R,MEGF10,NOD2,SIPA1,TACC1
cellular protein modification process	5	EP400,NOD2,RELN,SPTBN1,STYK1
enzyme regulator activity	5	KAL1,MEGF10,SERPINA4,SERPINA9,SIPA1
homeostatic process	5	ACACA,CASR,GIMAP5,IL7R,TRPC6
macromolecular complex assembly	5	ACACA,NOD2,SNAP91,SPICE1,SPTBN1
protein complex assembly	5	ACACA,NOD2,SNAP91,SPICE1,SPTBN1
signal transducer activity	4	CASR,IL7R,LILRB5,PDCD1
small molecule metabolic process	5	ACACA,ENTPD3,HGD,MGAM,SIPA1
transmembrane transport	5	ACACA,CASR,FER1L6,RELN,TRPC6
cell death	4	GIMAP5,NOD2,PDCD1,SNCAIP
cytoskeleton organization	4	SIPA1,SPICE1,SPTBN1,TACC1
extracellular space	4	KAL1,RELN,SERPINA4,SERPINA9

immune system process	4	GIMAP5,IL7R,NOD2,PDCD1
lipid metabolic process	4	ACACA,GIMAP5,NOD2,SDR42E1
membrane organization	4	GIMAP5,RELN,SNAP91,SPTBN1
mitochondrion	4	ACACA,GIMAP5,LIN28B,MICU3
neurological system process	4	KCNJ5,RELN,ROM1,SNCAIP
oxidoreductase activity	4	HGD,KDM3B,L2HGDH,SDR42E1
DNA metabolic process	3	C11orf30,IL7R,ORC1
RNA binding	3	EIF4G2,JAKMIP1,LIN28B
carbohydrate metabolic process	3	HEXA,MGAM,SDR42E1
cell division	3	SPICE1,SPTBN1,TACC1
cell motility	3	DNHD1,NOD2,RELN
cell-cell signaling	3	KCNJ5,RELN,SNCAIP
chromosome organization	3	C11orf30,EP400,KDM3B
enzyme binding	3	JAKMIP1,NOD2,SNCAIP
growth	3	IL7R,NOD2,SIPA1
microtubule organizing center	3	FRY,SPICE1,TACC1
nucleobase-containing compound catabolic process	3	ENTPD3,LIN28B,SIPA1
nucleolus	3	ORC1,SNCAIP,SPTBN1
transmembrane transporter activity	3	FER1L6,KCNJ5,TRPC6
ATPase activity	2	ABCA13,FER1L6
cilium	2	HYDIN,ROM1
cytoskeletal protein binding	2	JAKMIP1,SPTBN1
hydrolase activity, acting on glycosyl bonds	2	HEXA,MGAM
kinase activity	2	RELN,STYK1
lipid binding	2	SNAP91,SPTBN1
nucleoplasm	2	EP400,ORC1
peptidase activity	2	ADAMTS18,RELN
proteinaceous extracellular matrix	2	KAL1,RELN
structural molecule activity	2	KAL1,SPTBN1
Golgi apparatus	1	STX5
cellular amino acid metabolic process	1	HGD
chromosome	1	ORC1
chromosome segregation	1	SPICE1
cofactor metabolic process	1	ACACA
developmental maturation	1	RELN
generation of precursor metabolites and energy	1	ACACA
helicase activity	1	EP400
ligase activity	1	ACACA
lysosome	1	GIMAP5
mitochondrion organization	1	GIMAP5
mitosis	1	SPICE1
nuclear chromosome	1	ORC1
nucleocytoplasmic transport	1	SPTBN1

plasma membrane organization	1	SPTBN1
protein targeting	1	SPTBN1
sulfur compound metabolic process	1	ACACA
symbiosis, encompassing mutualism through parasitism	1	NOD2
vacuole	1	GIMAP5
