# Supplementary material for: Evolution of late-stage metastatic melanoma is dominated by aneuploidy and whole genome doubling
Source: Nat Commun. 2021 Mar 4;12:1434. doi: 10.1038/s41467-021-21576-8 (PMC7933255; doi:10.1038/s41467-021-21576-8)
Supplement: Supplementary file 2 — Reporting Summary [file 41467_2021_21576_MOESM2_ESM.pdf]

nature research

Corresponding author(s): Anthony T Papenfuss

Last updated by author(s): Sep 22, 2020

Reporting Summary

Nature Research wishes to improve the reproducibility of the work that we publish. This form provides structure for consistency and transparency in reporting. For further information on Nature Research policies, see Authors & Referees and the Editorial Policy Checklist.

Statistics

For all statistical analyses, confirm that the following items are present in the figure legend, table legend, main text, or Methods section.

n/a	Confirmed	
☐	☒	The exact sample size (*n*) for each experimental group/condition, given as a discrete number and unit of measurement
☐	☒	A statement on whether measurements were taken from distinct samples or whether the same sample was measured repeatedly
☐	☒	The statistical test(s) used AND whether they are one- or two-sided<br>*Only common tests should be described solely by name; describe more complex techniques in the Methods section.*
☒	☐	A description of all covariates tested
☒	☐	A description of any assumptions or corrections, such as tests of normality and adjustment for multiple comparisons
☐	☒	A full description of the statistical parameters including central tendency (e.g. means) or other basic estimates (e.g. regression coefficient) AND variation (e.g. standard deviation) or associated estimates of uncertainty (e.g. confidence intervals)
☒	☐	For null hypothesis testing, the test statistic (e.g. *F*, *t*, *r*) with confidence intervals, effect sizes, degrees of freedom and *P* value noted<br>*Give P values as exact values whenever suitable.*
☒	☐	For Bayesian analysis, information on the choice of priors and Markov chain Monte Carlo settings
☒	☐	For hierarchical and complex designs, identification of the appropriate level for tests and full reporting of outcomes
☒	☐	Estimates of effect sizes (e.g. Cohen's *d*, Pearson's *r*), indicating how they were calculated

Our web collection on statistics for biologists contains articles on many of the points above.

Software and code

Policy information about availability of computer code

Data collection	No software was used for data collection.
Data analysis	The SNV calling pipeline is available here: https://github.com/PapenfussLab/CascadePipe.<br>The genome doubling test is described in the methods and R code is available at https://github.com/PapenfussLab/Genome_doubling_test.<br>The neoantigen prediction pipeline is available at https://github.com/PapenfussLab/CascadeNeoPipe.<br>Open source bioinformatics analysis tools are described in detail in the methods and comprise cutadapt (v1.7.1), bwa (v0.7.12), picard (v1.128), GATK (v3.4.0), multiSNV (v2.3), VarScan (v.2.3), muTect (v3.1.0), IndelGenotyper (v1.04905), deconstructSigs (v1.9.0), TNT (v1.5) & GRIDSS (v1.3.2), Sequenza (v2.1.0), MEDICC (downloaded July 2014)

For manuscripts utilizing custom algorithms or software that are central to the research but not yet described in published literature, software must be made available to editors/reviewers. We strongly encourage code deposition in a community repository (e.g. GitHub). See the Nature Research guidelines for submitting code & software for further information.

Data

Policy information about availability of data

All manuscripts must include a data availability statement. This statement should provide the following information, where applicable:

- Accession codes, unique identifiers, or web links for publicly available datasets
- A list of figures that have associated raw data
- A description of any restrictions on data availability

All sequencing data has been deposited in the European Genome Phenome Archive (EGA accession EGAS00001004950).

Field-specific reporting

Please select the one below that is the best fit for your research. If you are not sure, read the appropriate sections before making your selection.

☒ Life sciences ☐ Behavioural & social sciences ☐ Ecological, evolutionary & environmental sciences

For a reference copy of the document with all sections, see nature.com/documents/nr-reporting-summary-flat.pdf

Life sciences study design

All studies must disclose on these points even when the disclosure is negative.

Sample size	The sample size was determined by the number of patients willing to be involved and the capacity of clinicians to recruit, as well as the number of metastases collected at autopsy, availability of primary and regional metastases.
Data exclusions	No data was excluded from the study.
Replication	13 patients were included in the study with 3-11 samples per patient.<br>SNVs were called using a consensus approach and a subset validated experimentally. Multiple samples per patient offered a form of pseudo replication, taken advantage of using MultiSNV and in copy number analysis. CNV calling was validated using FISH, replicated across cells.<br>Analyses were repeated multiple times using different tools or newer versions of tools.<br>No other experiments were performed.
Randomization	The study is retrospective in nature and the precious samples involved are identified and collected opportunistically at autopsy. Thus randomization is not possible or relevant.
Blinding	The cohort is comprised of a single group only, thus no blinding was necessary.

Reporting for specific materials, systems and methods

We require information from authors about some types of materials, experimental systems and methods used in many studies. Here, indicate whether each material, system or method listed is relevant to your study. If you are not sure if a list item applies to your research, read the appropriate section before selecting a response.

Materials & experimental systems

n/a	Involved in the study	
☒	☐	Antibodies
☒	☐	Eukaryotic cell lines
☒	☐	Palaeontology
☒	☐	Animals and other organisms
☐	☒	Human research participants
☒	☐	Clinical data

Methods

n/a	Involved in the study	
☒	☐	ChIP-seq
☒	☐	Flow cytometry
☒	☐	MRI-based neuroimaging

Human research participants

Policy information about studies involving human research participants

Population characteristics	6 male and 7 female patients aged 31-83 (median 48) years with advanced melanoma with varying stages at time of diagnosis.
Recruitment	Recruitment to the CASCADE rapid autopsy programme at the Peter MacCallum Cancer Centre was undertaken according to approved protocols (PMCC Human Research Ethics Committee approval numbers 11/102). Informed consent was obtained from all patients and families. Recruitment for the CASCADE Rapid Autopsy Program was described in detail in the cited reference Alsop, K. et al. A community-based model of rapid autopsy in end-stage cancer patients. Nat Biotechnol 34, 1010-1014, doi:10.1038/nbt.3674 (2016). All patients selected as part of CASCADE had BRAF mutations and brain mets.
Ethics oversight	Peter MacCallum Cancer Centre Human Research Ethics Committee. Informed consent was obtained from all patients and families.

Note that full information on the approval of the study protocol must also be provided in the manuscript.
